# An Operationally Unsaturated Iridium-Pincer Complex That C–H Activates Methane and Ethane in the Crystalline Solid-State

**DOI:** 10.1021/jacs.4c18122

**Published:** 2025-02-25

**Authors:** Matthew R. Gyton, M. Arif Sajjad, Daniel J. Storm, Kristof M. Altus, Joe C. Goodall, Chloe L. Johnson, Samuel J. Page, Alison J. Edwards, Ross O. Piltz, Simon B. Duckett, Stuart A. Macgregor, Andrew S. Weller

**Affiliations:** † Department of Chemistry, University of York, Heslington, York YO10 5DD, U.K.; ‡ EaStCHEM School of Chemistry, University of St Andrews, North Haugh, St Andrews KY16 9ST, U.K.; § Department of Chemistry, University of Durham, Durham DH1 3LE, U.K.; ∥ Australian Centre for Neutron Scattering, Australian Nuclear Science and Technology Organisation, Lucas Heights, New South Wales 2234, Australia

## Abstract

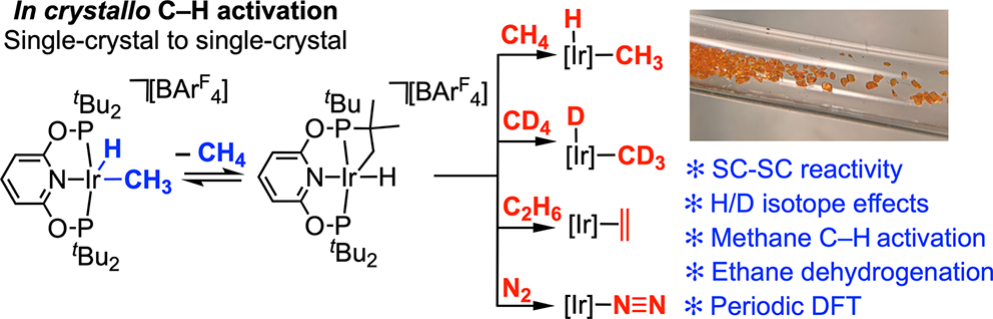

The known complex [Ir(^*t*^Bu-PONOP)MeH][BAr^F^_4_], **1[BAr**^**F**^_**4**_**]** [^*t*^Bu-PONOP **=** κ^3^-2,6-(^*t*^Bu_2_PO)_2_C_5_H_3_N); Ar^F^ = 3,5-(CF_3_)_2_(C_6_H_3_); *J. Am. Chem. Soc.***2009,** 131, 8603], is a robust precursor for *in crystallo* single-crystal to single-crystal (SC-SC) C–H activation of methane and ethane at 80 °C. This contrasts with the reported solution (CD_2_Cl_2_) behavior, where **1[BAr**^**F**^_**4**_**]** decomposes by methane loss. Crystalline **1[BAr**^**F**^_**4**_**]** is accessed as a single polymorph on a gram scale. A single-crystal neutron diffraction study locates the hydride. ^13^C{^1^H} SSNMR experiments on **1[BAr**^**F**^_**4**_**]**, and its isotopologue [Ir(^*t*^Bu-PONOP)(CD_3_)D][BAr^F^_4_], **d**_**4**_**-1[BAr**^**F**^_**4**_**]**, suggest a rapid and reversible endergonic reductive bond formation is occurring *in crystallo* to access an Ir(I) σ-methane complex. Heating **1[BAr**^**F**^_**4**_**]** to 80 °C under high vacuum results in loss of methane and intramolecular C–H activation to form cyclometalated [Ir(cyclo-^*t*^Bu-PONOP′)H][BAr^F^_4_], **2[BAr**^**F**^_**4**_**]**, in a SC-SC reaction. This is reversible, and the addition of CH_4_ or CD_4_ to **2[BAr**^**F**^_**4**_**]** at 80 °C results in an equilibrium with **1[BAr**^**F**^_**4**_**]** or **d**_**4**_**-1[BAr**^**F**^_**4**_**]**, respectively. Complex **2[BAr**^**F**^_**4**_**]** is thus an operationally unsaturated source of 14-electron [Ir(^t^Bu-PONOP)][BAr^F^_4_], **III**, that undergoes C–H activation with methane. Periodic DFT studies, alongside isotope labeling experiments, link **1[BAr**^**F**^_**4**_**]** and **2[BAr**^**F**^_**4**_**]**/CH_4_ via a reductive elimination/oxidative addition pathway. Heating **2[BAr**^**F**^_**4**_**]** to 80 °C under N_2_ forms [Ir(^*t*^Bu-PONOP)(κ^1^-N_2_)][BAr^F^_4_], in a SC-SC transformation. Reaction with CO forms [Ir(^*t*^Bu-PONOP)(CO)][BAr^F^_4_] at room temperature. Calculations suggest reaction with N_2_ occurs via an associative process or competitively through **III**, while with CO only an associative process operates. Heating **2[BAr**^**F**^_**4**_**]** to 80 °C under an ethane atmosphere results in alkane dehydrogenation, via a SC–SC reaction, forming a ∼1:1 mixture of [Ir(^*t*^Bu-PONOP)(η^2^-H_2_C=CH_2_)][BAr^F^_4_], and [Ir(^*t*^Bu-PONOP)H_2_][BAr^F^_4_].

## Introduction

The coordination and activation of methane in molecular metal/ligand complexes has been a grand-challenge in organometallic chemistry^1−7^ since the report by Shilov in the early 1970s of homogeneous platinum-based systems that convert methane to methanol.^8^ The interest in methane activation comes from the abundance of this C_1_ feedstock and opportunities for upgrading into more valuable chemicals or energy vectors.^9,10^ While heterogeneous catalysis offers promise for methane valorization on an industrial scale;^11,12^ doing so selectively, efficiently and under relatively mild conditions represents a significant challenge, as the C–H bond in methane is strong (homolytic bond strength = 105 kcal/mol,^13^ nonpolar and sterically hindered (sp^3^ carbon). Nevertheless elegant examples of catalytic methane activation using homogeneous molecular organometallic complexes have been reported.^1,14−21^ Several mechanisms for the C–H bond activation of methane have been established, and include outer-sphere processes (e.g., in metalloenzymes or iron-oxo complexes^22^) or σ-bond metathesis.^23^ Mechanisms based on inner-sphere coordination of the C–H bond to form an intermediate σ-methane complex,^24^ with a 3c–2e M···H–C bond,^25^ result in activation by oxidative cleavage or σ-complex assisted metathesis.^26,27^ The role of reactive unsaturated intermediates that are primed for coordination of methane to form intermediate σ-complexes are thus important in its C–H activation.

**Figure 1:**
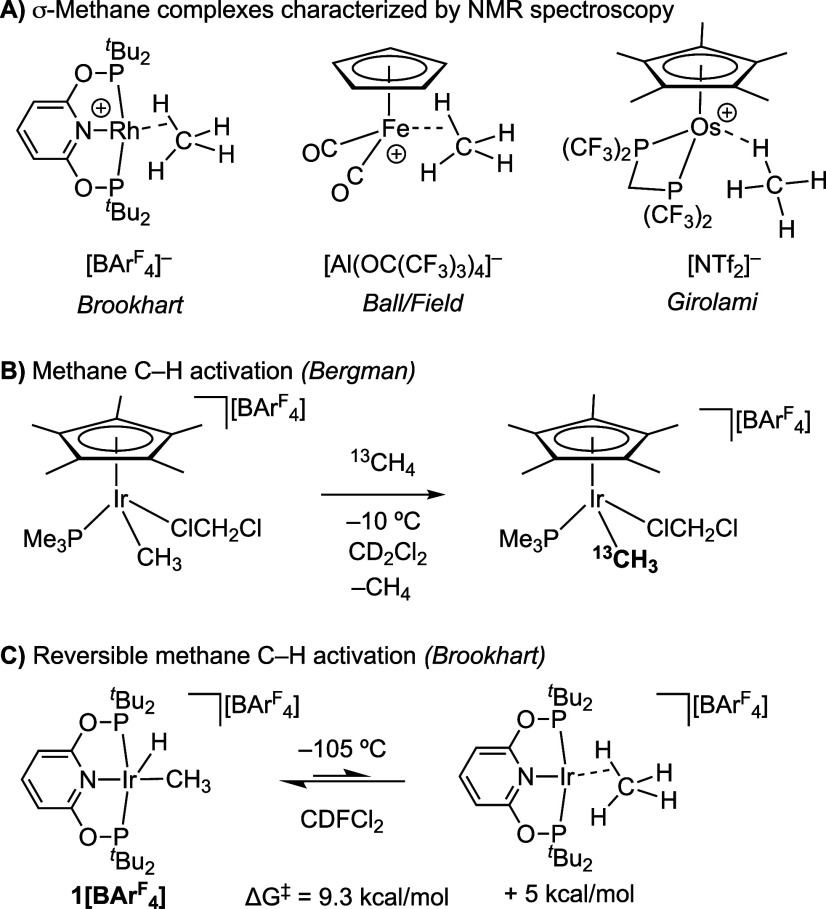
Methane σ-complexes and C–H activation.

Despite this considerable interest, the observation of precursor methane σ-complexes,^7,28−32^Figure 1A, or the direct products of methane C–H activation,^2,33−40^ exemplified in Figure 1B,^41^ are still relatively rare, as the weak binding of methane, and alkanes more generally^6,7,24,27^, to metal centers results in competition with solvent or a displaced ligand, such as photochemically generated CO. Consequently, σ-methane complexes are short-lived and have only been characterized *in situ* at low temperatures by matrix isolation methods, or in solutions of weakly binding solvents, using NMR or time-resolved infrared spectroscopy. These conditions also mean that a structural analysis of a σ-methane complex using single-crystal X-ray diffraction has not yet been realized, although *in situ* neutron powder diffraction experiments on methane uptake in MOF-type materials show metal···H_4_C interactions, albeit weak.^42,43^ In addition, as the thermodynamics of methane C–H activation are finely balanced, the position of the dynamic equilibrium between a σ-complex and its tautomeric methyl hydride can be influenced by the identity of the metal center and ligand choice.^28,44−47^ Of direct relevance to this work is the example from Brookhart and coworkers of an Ir(III) methyl hydride complex [Ir(^*t*^Bu-PONOP)MeH][BAr^F^_4_], **1[BAr**^**F**^_**4**_**]**,^48^ that is in rapid exchange with the corresponding, higher energy, Ir(I) σ-methane complex [Ir(^*t*^Bu-PONOP)(H_4_C)][BAr^F^_4_] at −105 °C in CDCl_2_F solution, Figure 1C [^*t*^Bu-PONOP **=** κ^3^-2,6-(^*t*^Bu_2_PO)_2_C_5_H_3_N; Ar^F^ = 3,5-(CF_3_)_2_C_6_H_3_].

**Figure 2:**
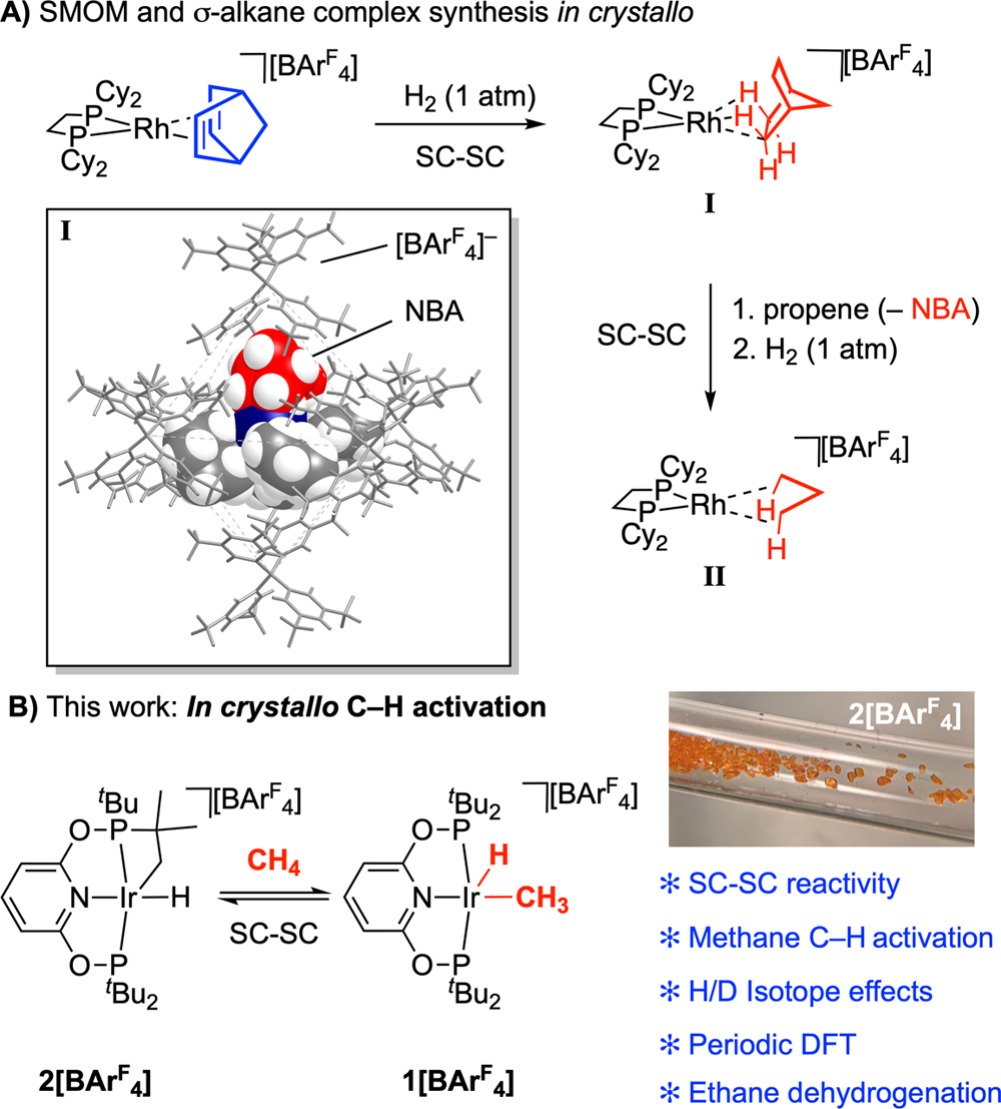
(A) *In crystallo* SMOM and σ-alkane complexes. Cation shown at van der Waals radii. (B) This work. SC-SC = single-crystal to single-crystal.

We have been developing solid/gas single-crystal to single-crystal^49−51^ (SC-SC) *in crystallo*(52) reactivity to synthesize remarkably stable, compared with *in situ* solution methods, σ-alkane complexes by addition of excess H_2_ to precursor alkene complexes.^53^ We refer to this approach as solid-state molecular organometallic chemistry, SMOM,^54^ that is related to, but distinct from, surface organometallic chemistry, SOMC,^55^ where highly active organometallic complexes are grafted onto a platform support. The prototypical SMOM example is the addition of H_2_ to single-crystals of the cationic Rh(I) complex [Rh(Cy_2_PCH_2_CH_2_PCy_2_)(NBD)][BAr^F^_4_] (NBD = norbornadiene). This results in rapid double-bond hydrogenation and formation of the corresponding room temperature stable σ-alkane complex, [Rh(Cy_2_PCH_2_CH_2_PCy_2_)(NBA)][BAr^F^_4_] **I** (NBA = norbornane), that can be characterized by single-crystal X-ray and neutron diffraction, solid-state NMR spectroscopy and periodic DFT calculations, Figure 2A.^56−58^ With solvent absent, and any competitive pre-equilibria for alkane binding removed, such σ-alkane complexes have been shown to undergo room temperature intramolecular C–H activation *in crystallo*, for example selective H/D exchange at bound NBA or cyclohexane with D_2_^58,59^ or alkane dehydrogenation of bound cyclohexane.^59^ The stability in the solid-state comes from the absence of solvent combined with the secondary microenvironment provided by a cage of [BAr^F^_4_]^−^ anions that encapsulate the cationic σ-alkane complex, in which noncovalent interactions with the alkane (directional C–F···H–C and more diffuse C–H···π) play an important role.^57,60−62^ These anions, with their CF_3_ groups, also allow for substrate ingress and product egress through the nonporous crystalline lattice.^63^ For example, **I** reacts with propene to displace the bound NBA ligand in a SC-SC transformation to form the corresponding propene complex,^54^ which can then undergo further SC-SC reaction with H_2_ to form the corresponding (albeit short-lived) crystallographically characterized σ-propane complex, **II**.^60^

The intermolecular coordination and activation of methane is, however, challenging using this SMOM ligand exchange approach. First, the pseudodegenerate exchange of a relatively strongly^57,60^ encapsulated alkane ligand (i.e., η^2^η^2^-NBA) for methane is likely to be both thermodynamically disfavored and kinetically slow. Second, while *in crystallo* hydrogenolysis of a suitable precursor, such as a methylidene complex,^64^ would form methane, this would rapidly be displaced by H_2_ under the conditions of excess H_2_,^45,65^ and is further complicated by the local concentration of H_2_ in a single-crystal being modified by spatial and temporal constraints.^66−69^ We now report an alternative approach for the straightforward coordination and activation of methane in a SC-SC process using a molecular organometallic complex, Figure 2B. The *in crystallo* synthesis of an “operationally unsaturated”^70−72^ cyclometalated Ir(III)-hydride pincer complex, **2[BAr**^**F**^_**4**_**]**, allows for C–H activation of methane at a reactive Ir(I) center to form the corresponding methyl hydride, **1[BAr**^**F**^_**4**_**]**,^48^ under mild conditions (80 °C, up to 8 bar CH_4_). This reaction is reversible while retaining single crystallinity, and light alkane activation can be extended to the dehydrogenation of ethane. The generation of unsaturated 14-electron Ir(I)-pincer complexes that undergo C–H activation is of direct relevance to alkane-dehydrogenation catalysis where such intermediates are postulated.^73,74^

## Results and Discussion

### Isolation of Room Temperature Stable [Ir(^*t*^Bu-PONOP)MeH][BAr^F^_4_], 1[BAr^F^_4_], as Single Crystals on Gram Scale: Polymorphs and Structural Characterization Using Single-Crystal Neutron Diffraction

To generate an operationally unsaturated complex using *in crystallo* methods we chose Brookhart’s [Ir(^*t*^Bu-PONOP)MeH][BAr^F^_4_], **1[BAr**^**F**^_**4**_**]**, as its low temperature recrystallization (−35 °C) on the 20–30 mg scale, and structural characterization using single-crystal X-ray diffraction, have been reported.^48^**1[BAr**^**F**^_**4**_**]** is unstable in solution, losing methane via reductive elimination at 29 °C [*t*_1/2_= 3 h]. The organometallic products of methane loss were not described, although for related systems binding of solvent has been proposed^46^ or observed.^29,75^ We speculated that *in crystallo* loss of methane from **1[BAr**^**F**^_**4**_**]**, in the absence of solvent, would result in a reactive low coordinate species that could then undergo further C–H activation processes with gaseous alkanes. In support of this approach, we and others, have shown that pincer-ligand motifs can support single-crystal to single-crystal solid/gas reactivity.^64,76−79^

**Figure 3:**
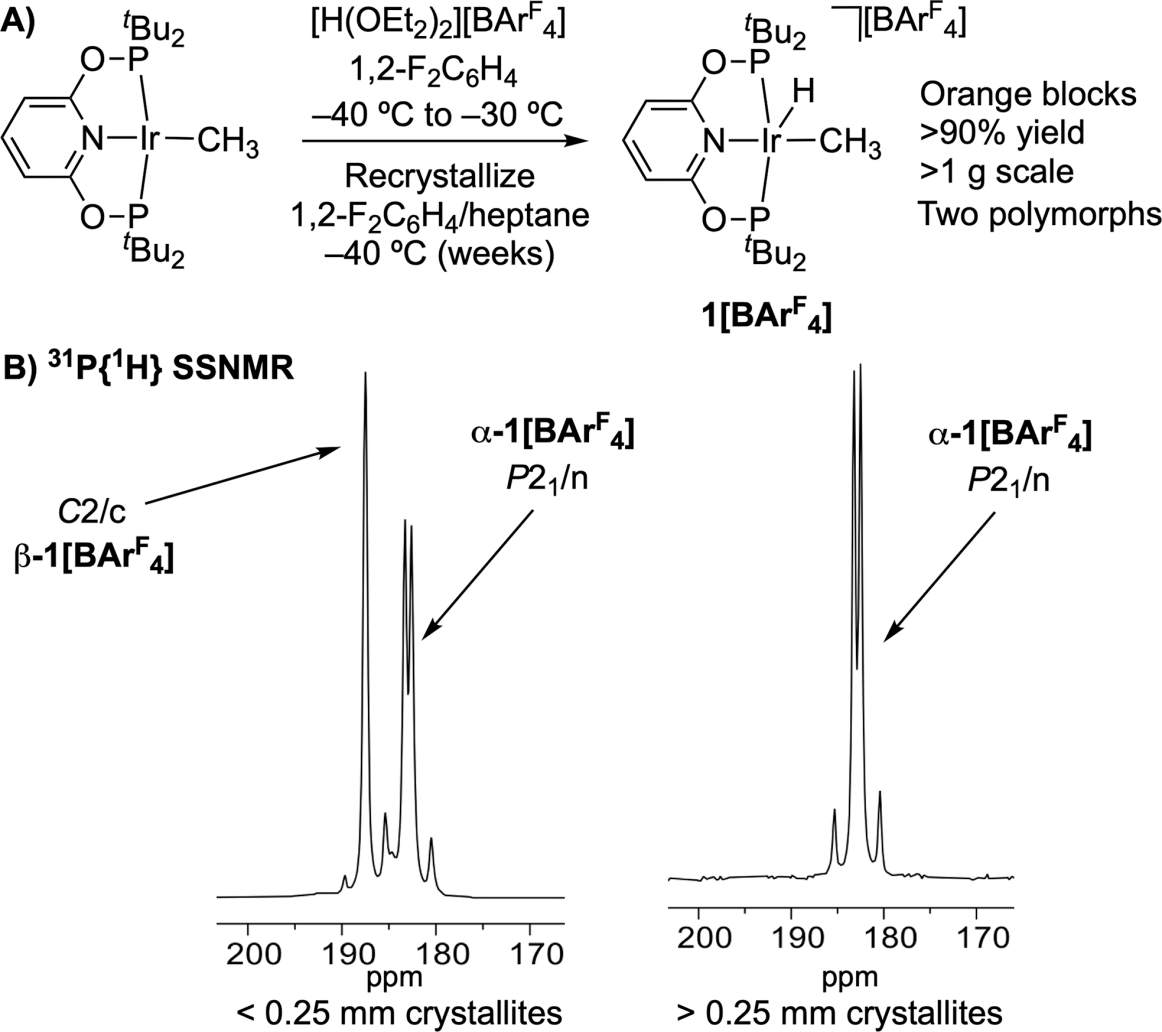
(A) Synthesis and isolation of **1[BAr**^**F**^_**4**_**]**. (B) ^31^P{^1^H} SSNMR (25 °C) of the sieved and graded crystalline polymorphs of **α-1[BAr**^**F**^_**4**_**]** and **β-1[BAr**^**F**^_**4**_**]**.

Using a slightly revised preparation, **1[BAr**^**F**^_**4**_**]** was synthesized and isolated by slow (weeks) low temperature recrystallization at −40 °C (1,2-F_2_C_6_H_4_/heptane) to repeatably afford orange blocks in greater than 90% isolated yield on a 1.1–1.4 g scale, Figure 3A. In contrast to its instability in solution, under an argon atmosphere at ambient temperatures crystalline **1[BAr^F^_4_]** is stable for months, as shown by low temperature (−70 °C) solution NMR, ambient temperature solid-state NMR (SSNMR) spectroscopy and single-crystal diffraction. Remarkably, crystalline **1[BAr^F^_4_]** is also tolerant to degassed D_2_O at room temperature as measured by NMR spectroscopy after 1 week (Figures S13–S16). Solution NMR data (−70 °C, CD_2_Cl_2_) are in full agreement with the reported literature values.^48^ In the ^1^H NMR spectrum the Ir–hydride is observed at δ −41.85 and Ir–methyl at δ 1.85. A single environment is observed in the ^31^P{^1^H} NMR spectrum at δ 184.3. In contrast, ^31^P{^1^H} SSNMR spectroscopy revealed that this initially isolated crystalline material is dimorphic. This is shown by the observation of two distinct sets of tightly coupled AB doublets, centered at δ 187.5, **β-1[BAr**^**F**^_**4**_**]**, and δ 182.9, **α-1[BAr**^**F**^_**4**_**]**. Each shows *trans*^31^P–^31^P coupling [*J*(PP) ∼ 340 Hz], suggesting crystallographically inequivalent phosphine groups for each polymorph. While these polymorphs could not be distinguished visually, they could be reliably separated by graded sieving at ambient temperature (Figure S1). Crystallites of greater than 0.25 mm consist of the major, pure, polymorph, **α-1[BAr**^**F**^_**4**_**]**, and could be isolated in ∼90% overall yield on the ∼1 g scale. Crystallites of less than 0.25 mm are a mixture of α-and β-forms, Figure 3B. As different morphologies are likely to display different stabilities and reactivity profiles in single-crystal solid/gas reactions,^49,54,79−83^ the isolation of a single polymorph, **α-1[BAr**^**F**^_**4**_**]**, provides a consistent framework for subsequent reactivity studies.

The previously reported solid-state structure of **1[BAr**^**F**^_**4**_**]**,^48^ as determined from crystals recrystallized at low temperature from CH_2_Cl_2_/diethyl ether, contains a molecule of solvent (CH_2_Cl_2_) in the lattice while the Ir–hydride was not located. We have recollected the structure (at −163 °C) using material of the major polymorph that was crystallized at −40 °C using 1,2-F_2_C_6_H_4_/heptane followed by graded sieving (crystals of greater than 0.25 mm). The structure shows (Figure S91) that single-crystals of phase-pure **α-1[BAr**^**F**^_**4**_**]** (*P*2_1_/n space group) contain no lattice solvent, which removes potential complications in subsequent SC-SC reactivity that are associated with loss of solvent.^82^ There is no crystallographically imposed symmetry, consistent with the inequivalent ^31^P environments observed in the SSNMR (Figure 3B). However, despite the structural refinement being good [*R*_int_ = 3.04%; *R*(2σ) = 3.24%] the hydride ligand could not be located. The position of the hydride above or below the Ir(PONOP)CH_3_-plane is nondegenerate in the solid-state. While there was no indirect evidence for Ir–H disorder from ^31^P{^1^H} or ^13^C{^1^H} SSNMR spectroscopy (Figures 3B, S6 and S9) the location of the hydride was thus unresolved.

**Figure 4:**
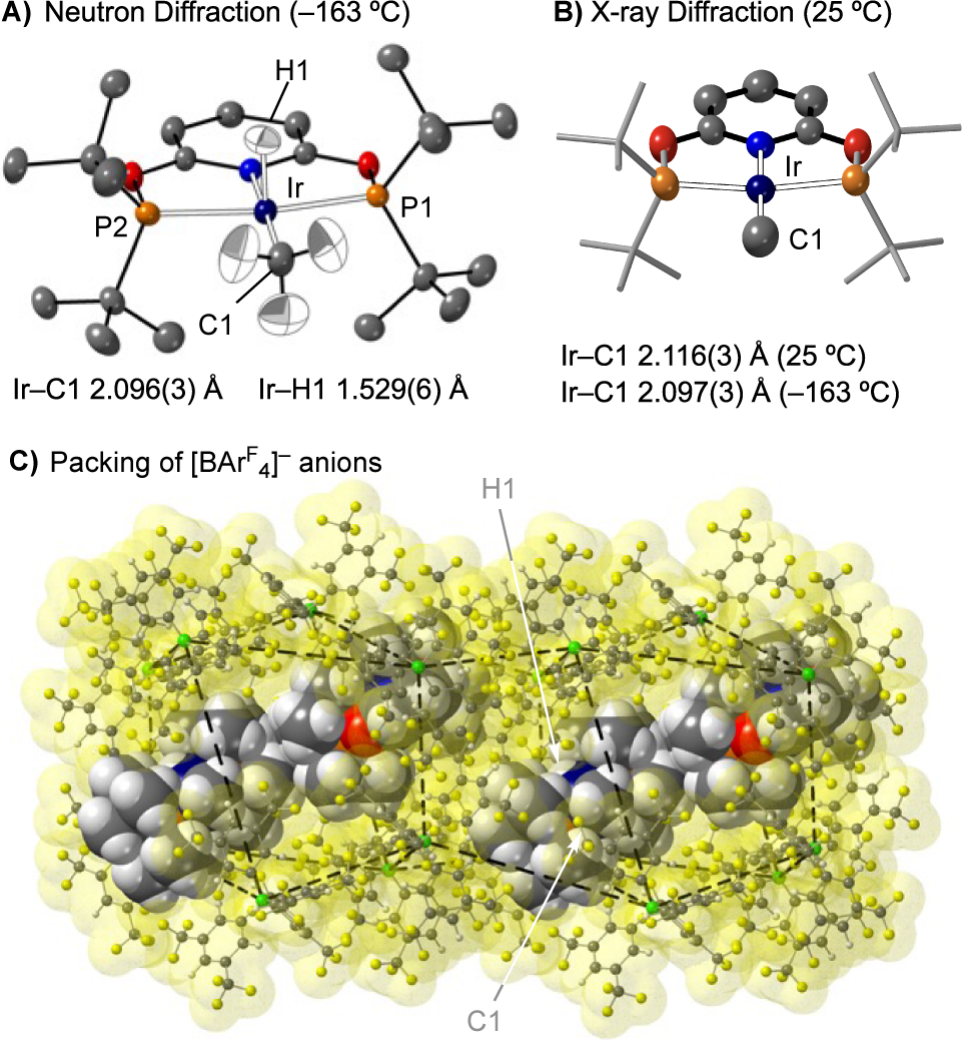
Structure of the cation in **α-1[BAr^F^_4_]** as determined by single-crystal neutron diffraction at −163 °C (A) and X-ray diffraction at 25 °C (B). Displacement ellipsoids at the 50% probability level, selected H atoms shown. (C) Packing arrangement of [BAr^F^_4_]^−^ anions in **α-1[BAr^F^_4_]**, with H1 and C1 highlighted. Cation atoms and anion molecular surfaces shown at van der Waals radii.

Precise determination of the position of the hydride ligand came from a single-crystal neutron diffraction study of **α-1[BAr**^**F**^_**4**_**]**. Single crystals (1.0 × 1.2 × 1.8 mm), that had been stored at ambient temperature under argon, were studied using Laue neutron diffraction at −163 °C, Figure 4A. This revealed the same overall structure as from the single-crystal X-ray experiment, with the hydride and methyl group hydrogens additionally directly located and freely refined with no evidence for disorder [Ir–H1, 1.529(6) Å]. The Ir–C1 distance, 2.096(3) Å, is consistent with an Ir(III)–CH_3_ group^45,84^ and not Ir(I)···H_4_C, which would be expected to have a longer Ir···C distance of at least 2.3 Å.^46^

The anions surrounding the cation in **α-1[BAr**^**F**^_**4**_**]** form a twisted, pseudo cubic arrangement, Figure 4C, with each cation sitting in an irregular quadrilateral face of [BAr^F^_4_]^−^ anions. While this pattern is unusual in SMOM, with ∼octahedral,^54,56,62^ and ∼bicapped square prismatic,^76,77,82^ more common, similar arrangements have been noted previously.^85^ While the other polymorph **β**-**1[BAr**^**F**^_**4**_**]** (using graded crystallites of less than 0.25 mm) could not be isolated in compositionally pure form separated from **α-1[BAr**^**F**^_**4**_**]**, screening of individual single crystals allowed for its structure to be determined (Figures S94 and S95). This showed a bicapped square prismatic arrangement of [BAr^F^_4_]^−^ anions around two crystallographically equivalent cations,^64,76,77^ in the *C*2/c space group. The Ir(I)-cation is essentially the same in both polymorphs.

### Variable Temperature X-ray Diffraction and SSNMR, Evidence for a Dynamic Equilibrium That Accesses an Ir(I)σ-Methane Complex

**Figure 5:**
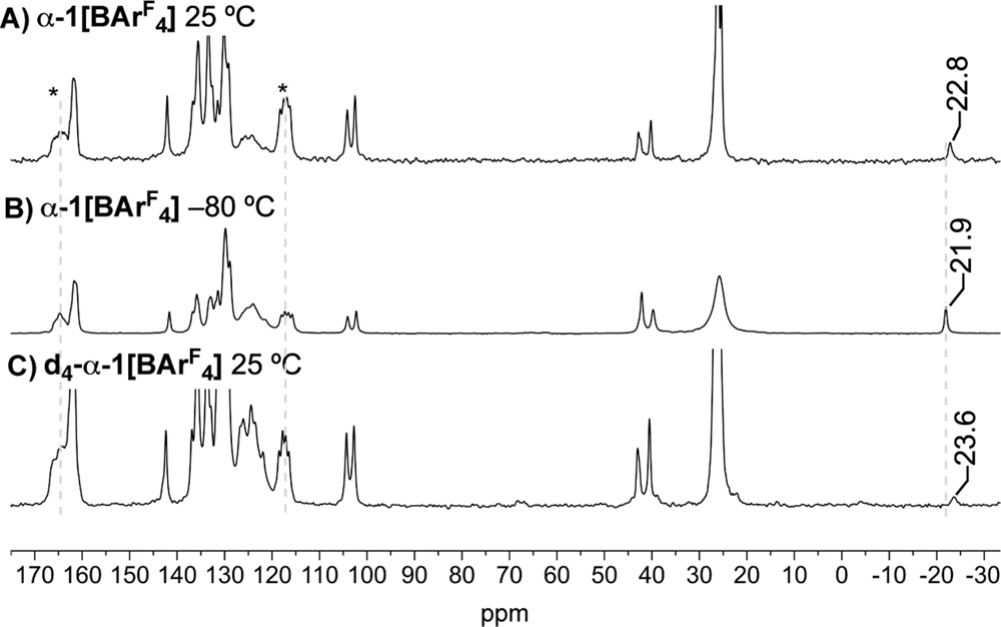
Variable temperature ^13^C{^1^H} CPTOSS (total suppression of side-bands) SSNMR spectra of **α-1[BAr^F^_4_]** and **d_4_α-1[BAr^F^_4_]**. *Selected peaks assigned to [BAr^F^_4_]. Dotted lines to guide the eye. Referenced to adamantane (external).

In CDCl_2_F solution it has been determined, using quantitative EXSY NMR experiments, that rapid site exchange between Ir–H and Ir–Me occurs at −105 °C for **1[BAr**^**F**^_**4**_**]** with a barrier of 9.3(4) kcal/mol, via an Ir(I) σ-methane intermediate.^48^ While this σ-methane complex is ∼5 kcal/mol higher in energy (gas-phase calculations on a truncated model^46^), we were interested to see if an ambient temperature single-crystal X-ray diffraction experiment revealed that this exchange process also occurred *in crystallo*. Such an exchange would be signaled by a lengthening of the Ir···C distance at higher temperatures, as a larger proportion of the σ-methane complex would be present in any dynamic admixture. Aside from the expected small increase in unit cell volume (4%), the structure collected at +25 °C shows a statistically significant (3σ) but very small change in the Ir–C bond metrics: Ir–C1 = 2.116 (3) Å, Figure 4B. While very small, this nevertheless supports a dynamic reductive coupling to form a σ-methane complex is occurring in the solid-state. However, if such a process is occurring then the equilibrium position at 298 K must still favor the methyl-hydride structural extreme. As NMR spectroscopy is potentially more sensitive to small changes in the weighted-average equilibrium position of the Ir–CH_3_ group,^86^ the ^13^C{^1^H} SSNMR spectrum of **α-1[BAr**^**F**^_**4**_**]** was recorded at 25 °C and −80 °C, Figures 5A,B. These data demonstrate a small, but significant, upfield chemical shift change of the Ir–CH_3_ signal on warming, moving from δ −21.9 to δ −22.8. As an Ir(I) σ-methane complex is expected to have a ^13^C chemical shift of ∼δ −40 to −50,^28,29,31^ and a cationic Ir(III)–CH_3_ would be observed at lower field ∼δ −20,^84^ this suggests a small shift in the weighted-average position of a dynamic equilibrium toward the σ-methane complex at room temperature. The signals due to the [BAr^F^_4_]^−^ anions do not shift significantly.

These observations are consistent with a kinetically accessible σ-methane complex being in rapid equilibrium with a thermodynamically favored ground–state methyl-hydride in the solid-state. As shown next, isotopic labeling studies, reactivity profiles and periodic DFT calculations support this hypothesis.

### Synthesis of **d_4_-α-1[BAr^F^_4_]**. Isotopic Perturbation of Equilibrium in the Solid-State

**Scheme 1:**
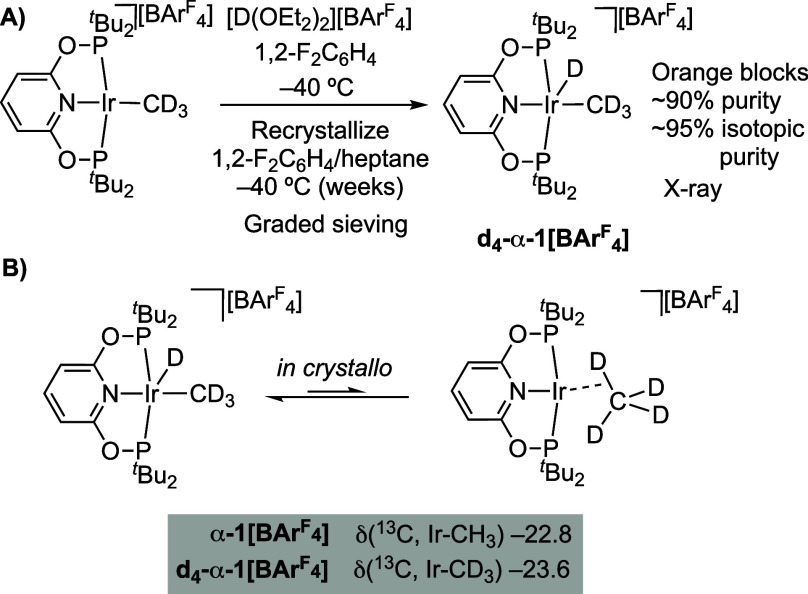
(A) Synthesis of **d_4_-α-1[BAr^F^_4_]**; (B) Proposed Isotopic Perturbation of Equilibrium at 25 °C

The *d*_4_-isotopologue of **1[BAr**^**F**^_**4**_**]** was prepared by addition of [D(OEt_2_)_2_][BAr^F^_4_]^87^ to Ir(^*t*^Bu-PONOP)(CD_3_)^48^ in 1,2–F_2_C_6_H_4_ solvent at −40 °C (Scheme 1A), and isolated as a crystalline solid from a low-temperature recrystallization, followed by graded sieving (crystals greater than 0.25 mm). Solution NMR data (CD_2_Cl_2_, −95 °C) show that [Ir(^*t*^Bu-PONOP)(CD_3_)D][BAr^F^_4_], **d**_**4**_**-1[BAr**^**F**^_**4**_**]**, is formed in ∼90% chemical and ∼95% isotopic purity, alongside minor impurities, including cyclometalated **2[BAr**^**F**^_**4**_**]** (∼2%).^88^^31^P{^1^H} SSNMR spectroscopy of crystalline material (Figures S32) shows that the α-polymorph is formed, **d**_**4**_**-**α**-1[BAr**^**F**^_**4**_**]**.

If a rapid, and reversible equilibrium was occurring between an Ir(III)-methyl-hydride and an Ir(I) σ-methane complex, as, at a first approximation,^89^ D prefers to reside in the higher bond-strength oscillator (i.e., C–D rather than Ir–D) an isotopic perturbation of equilibrium may be expected for **d**_**4**_**-**α**-1[BAr**^**F**^_**4**_**]**,^89−91^ that would bias toward the σ-methane complex. SSNMR spectroscopy supports such a perturbation, as the room temperature ^13^C{^1^H} SSNMR spectrum shows the Ir–CD_3_ signal shifted to higher field compared with the protio-analog (δ −23.6 versus δ −22.8 respectively), Figure 5C and Scheme 1B.

Chemical shift perturbations, with and without isotopic labeling, have been noted as being diagnostic of the existence of rapid oxidative cleavage/reductive bond formation equilibria occurring in solution, for example in (Cp^R^)_2_Nb(H_2_B(O_2_C_6_H_3_R′)) (Cp^R^ = C_5_H_5_, C_5_Me_5_, R′ = H, ^*t*^Bu) between Nb(V)-dihydride boryl and Nb(III)-hydrido borate structures.^86,92^ Reversible reductive elimination has recently been reported for [Ir(^*i*^Pr-PNP)(SiR_3_)(CH_3_)][BAr^F^_4_] pincer complexes, closely related to **1[BAr**^**F**^_**4**_**]**.^84^ While the chemical shift changes observed here are small, that they can be observed at all at room temperature is due to the stabilizing effect of the solid-state crystalline environment, which means that **1[BAr**^**F**^_**4**_**]** or **d**_**4**_**-1[BAr**^**F**^_**4**_**]** do not lose methane readily at room temperature *in crystallo*. However, on relatively mild heating methane is reversibly lost in a SC-SC transformation to form the operationally unsaturated cyclometalated complex, **2[BAr**^**F**^_**4**_**]**, as described next.

### Methane Reductive Elimination and Cyclometalation *In Crystallo*. Synthesis and Characterization of [Ir(cyclo-^*t*^Bu-PONOP′)H][BAr^F^_4_]

**Figure 6:**
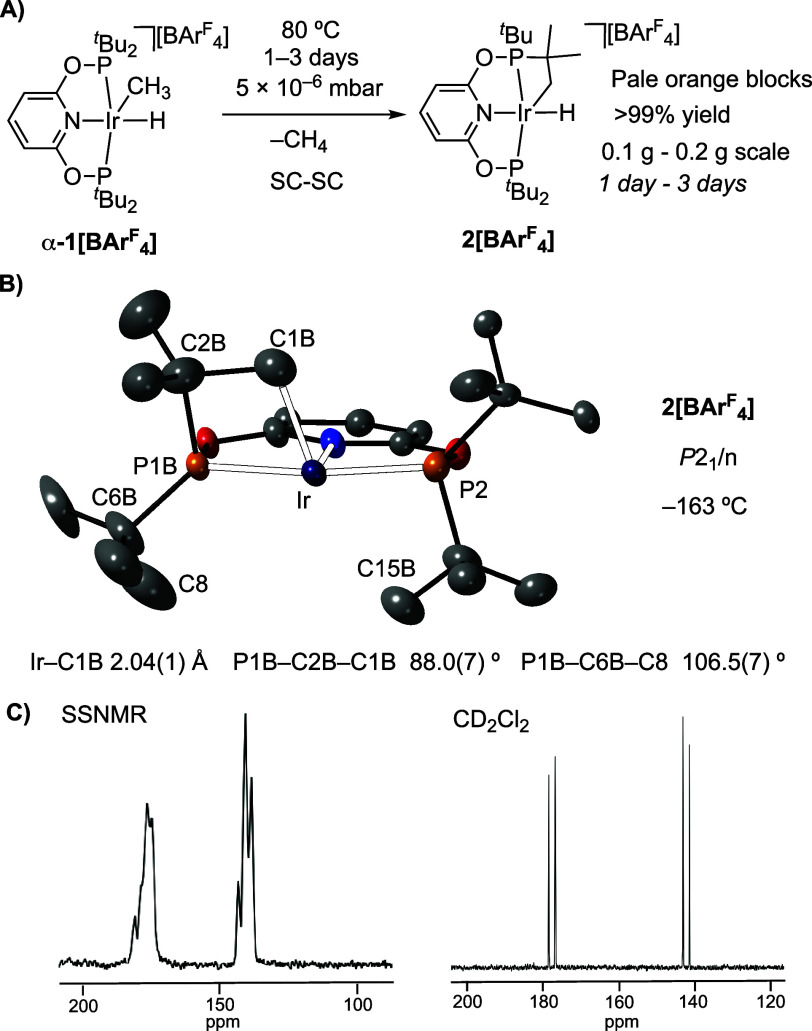
(A) Synthesis of **2[BAr**^**F**^_**4**_**]**. (B) Structure of the cation in one of the disorder components of **2[BAr**^**F**^_**4**_**]** from single crystal X-ray diffraction. Displacement ellipsoids shown at the 30% probability levels. H-atoms omitted for clarity. (C) ^31^P{^1^H} SSNMR (left) and solution NMR (right) spectra at 25 °C of **2[BAr**^**F**^_**4**_**]**.

Heating single crystals of **α-1[BAr**^**F**^_**4**_**]** to 80 °C under a dynamic high vacuum (5 × 10^–6^ mbar) for 3 days (200 mg scale) resulted in the formation of Ir(III)-cyclometalated [Ir(cyclo-^*t*^Bu-PONOP′)H][BAr^F^_4_], **2[BAr**^**F**^_**4**_**]**, in 99% recovered yield as single-crystalline pale orange blocks, [cyclo-^*t*^Bu-PONOP′ = κ^4^-2-(^*t*^Bu(H_2_CCMe_2_)-PO)-6-(^*t*^Bu_2_PO)C_5_H_3_N)].^93^ This SC-SC procedure is repeatable (over 10 times) and has been performed on sample sizes of 0.1–0.2 g (1 to 3 days respectively). Figure 6 shows the solid-state structure determined for the cation in **2[BAr**^**F**^_**4**_**]**.

The single crystal X-ray diffraction analysis of **2[BAr**^**F**^_**4**_**]** (−163 °C) shows that the α-crystal form from the starting material is retained (Figure S97), and the unit cell volume is essentially unchanged (Δ*V* = −0.8%). Loss of methane has occurred, and one of the ^*t*^Bu groups has undergone a cyclometalation^94^ to form the corresponding Ir(III)–alkyl hydride. The solid-state structure shows a superposition of chemically equivalent isomers, arising from C–H activation of distinct ^*t*^Bu groups. The best model [*R*_int_ = 3.1%; *R*(2σ) = 3.84%] converges to a two site disorder in which ^*t*^Bu groups from the same phosphine (P1) have undergone C–H activation and cyclometalation in the apical positions of the complex, i.e., above/below the PONOP–Ir plane. The hydride was not located, but is likely situated *trans* to the pyridyl nitrogen, consistent with its rather low field chemical shift and *J*(PH) coupling constants (*vide infra*). The Ir–C1B distance [2.039(13) Å] and compressed P1B–C2B–C1B angle [88.0(7)°] are consistent with cyclometalation. There is a relatively close approach of another ^*t*^Bu methyl group to the vacant site *trans* to C1B [Ir···C15B = 2.884(16) Å] which may suggest an agostic interaction.^95^ Although there is no evidence from solution NMR spectroscopy for this, a weak agostic interaction is computed in the static structure (see below). Closely related cyclometalated Ir(III) pincer complexes show similar structures in terms of apical C–H activation, and Ir–C distances.^96−101^ While this superposition of disordered components means the detailed structural metrics should be interpreted with caution, the SC-SC reactivity is unambiguous: on heating **α-1[BAr**^**F**^_**4**_**]** to 80 °C under high vacuum methane loss results in conversion to cyclometalated **2[BAr**^**F**^_**4**_**]**. Further confirmation of the cyclometalated motif came from dissolving crystalline **2[BAr**^**F**^_**4**_**]** in MeCN and the isolation of, structurally characterized, **2-MeCN[BAr**^**F**^_**4**_**],** [Ir(cyclo-^*t*^Bu-PONOP′)H(NCMe)][BAr^F^_4_], Figure S98, as the only product.^102^

Room temperature solid-state and solution NMR spectroscopies show that this conversion to **2[BAr**^**F**^_**4**_**]** is quantitative, and data are similar to those for other cyclometalated Ir(III) pincer complexes.^96−101^ In the ^31^P{^1^H} SSNMR spectrum multiple overlapping environments are observed, centered at δ 175 and 140. The solution ^31^P{^1^H} NMR spectrum is much simpler, with signals observed at δ 177.7 and 141.8 that show *trans*^31^P–^31^P coupling [*J*(PP) = 345 Hz], Figure 6C. These data are consistent with the disorder associated with cyclometalation in the solid-state, which is resolved in solution as the different components become degenerate. The cyclometalated methylene is observed at δ −3.7 (br) in the ^13^C{^1^H} SSNMR spectrum and δ −2.5 [dd, *J*(CP) = 26, 2 Hz] in solution. In the ^1^H NMR spectrum the Ir–H hydride is observed as an apparent triplet δ −9.24 [dd, *J*(HP) = 11.3 Hz], and the diastereotopic methylene group at δ 3.09 and 0.79.

Combined, these data also show that there is no fast exchange between the cyclometalated and other ^*t*^Bu groups on the NMR time scale at room temperature *in crystallo* or in solution, unlike for some other operationally unsaturated ^*t*^Bu-cyclometalated complexes that undergo fast exchange in solution.^72,103,104^ As such a reversible exchange likely operates via low coordinate, low oxidation state, intermediates, we hypothesized that such an intermediate may be accessible from **2[BAr**^**F**^_**4**_**]** on heating *in crystallo* – informed by the reactivity of **1[BAr**^**F**^_**4**_**]**, and the reactivity of related cyclo-metalated complexes in solution.^72,104^ This was initially probed by reactivity with N_2_ and CO, coordination of which would trap out such an intermediate.

### *In Crystallo* Reactivity of [Ir(cyclo-^*t*^Bu-PONOP′)H][BAr^F^_4_] with CO, N_2_

**Figure 7:**
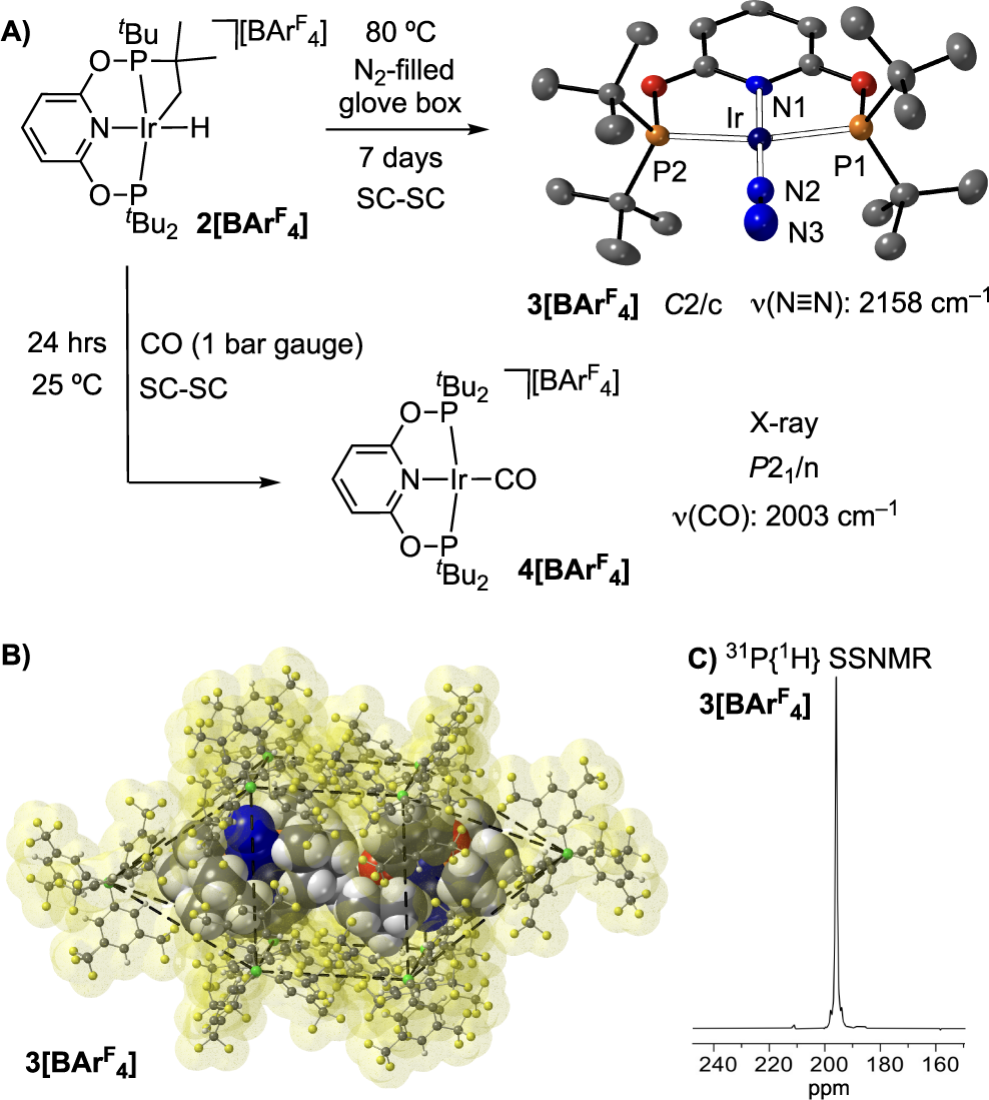
(A) SC-SC reactivity of **2[BAr**^**F**^_**4**_**]** with CO and N_2_. Solid-state structure of the cation of **3[BAr**^**F**^_**4**_**]**. Displacement ellipsoids are shown at the 50% level. (B) Packing diagram of **3[BAr**^**F**^_**4**_**].** Cation atoms and anion molecular surface shown at van der Waals radii. (C) ^31^P{^1^H} SSNMR of **3[BAr**^**F**^_**4**_**].**

The reaction of crystalline **2[BAr**^**F**^_**4**_**]** with N_2_ (80 °C, 7 days, flask open to an N_2_-filled glovebox) is a SC-SC transformation, that forms [Ir(^*t*^Bu-PONOP)(κ^1^-N_2_)][BAr^F^_4_], **3[BAr**^**F**^_**4**_**]**. No reaction occurs at ambient temperature. A single-crystal X-ray diffraction analysis, Figure 7A, shows a κ^1^-N_2_ ligand [N2–N3 = 1.120(12) Å]; while in the infrared spectrum the N≡N stretch is observed at ν_(N≡N)_ 2158 cm^–1^. The N≡N stretch in related pincer complexes [Rh(^*t*^Bu-PONOP)(κ^1^-N_2_)][BAr^F^_4_] [ν_(N≡N)_ 2202 cm^–1^]^75^ and Ir(^*t*^Bu-PCP)(κ^1^-N_2_) [ν_(N≡N)_ 2076 cm^–1^]^105^ reflect the back-bonding differences between 4*d*/5*d* and cationic/neutral metal centers. Interestingly, there is a space group change on going from **2[BAr**^**F**^_**4**_**]** to **3[BAr**^**F**^_**4**_**]**, from *P*2_1_/n to *C*2/c, the latter the same as **β-1[BAr**^**F**^_**4**_**]**. This manifests by a change in the anion-packing to a bicapped square prism and a characteristic ^31^P{^1^H} SSNMR spectrum Figure 7C (cf. Figure 3B). This SC-SC structural adaptivity^80,83^ on addition of an external gaseous reagent has been noted before in SMOM systems,^77,106^ as is facilitated by the −CF_3_ groups on the [BAr^F^_4_]^−^ anion that allow for significant plasticity in the single-crystal. There is, however, no significant change in the unit cell volume (*V*/*Z* = 1496 Å^3^, **3[BAr**^**F**^_**4**_**]**; 1502 Å^3^, **2[BAr**^**F**^_**4**_**]**).

This reactivity is consistent with **2[BAr**^**F**^_**4**_**]** acting as a source of a transient 14-electron Ir(I) [Ir(^t^Bu-PONOP)][BAr^F^_4_], **III**, by reductive elimination of the cyclometalated group at 80 °C, and that lattice-available N_2_ traps this reactive species by coordination. However, we cannot discount a mechanism in which initial binding of N_2_ to **2[BAr**^**F**^_**4**_**]** promotes reductive elimination (see Computational Studies Section). In support of this alternative mechanism being plausible, the reaction of CO with **2[BAr**^**F**^_**4**_**]** does not require heating and occurs over a much shorter time scale (24 h).

Addition of CO (1 bar gauge) to single-crystals of **2[BAr**^**F**^_**4**_**]** for 24 h at 25 °C (unoptimized) results in the quantitative formation of the known^45^ complex [Ir(^*t*^Bu-PONOP)(CO)][BAr^F^_4_], **4[BAr**^**F**^_**4**_**]**, Figure 7A. This is a SC-SC transformation that retains the same anion-packing and space group (*P*2_1_/n) of the starting material, as shown by the solid-state structure (Figures S100 and S102). Solution and SSNMR data confirm that C–H reductive elimination has occurred from **2[BAr**^**F**^_**4**_**]**. This presumably occurs via coordination of CO to form a six-coordinate Ir(III)-cyclometalated complex,^100^ that then undergoes reductive elimination.^107^ While it is generally accepted that reductive elimination in d^6^ complexes is favored from 5-coordinate species,^108^ ligand-assisted reductive elimination from 6-coordinate complexes can occur by addition of ligands such as alkenes or H_2_.^109,110^ Addition of CO has also been shown to promote reductive elimination in a closely related neopentyl cyclometalated Ir-pincer-hydride complex.^99,100^

While CO and N_2_ are similar sizes, studies on microporous MOF systems show that CO may diffuse more slowly than N_2_ through a relatively confined lattice.^111,112^ While this suggests that the relative rates of reaction observed here are not diffusion limited,^67^ the effects of local concentration and the space-group change associated with **3[BAr**^**F**^_**4**_**]** cannot be discounted and are currently unresolved.

### *In Crystallo* Reactivity of [Ir(cyclo-^*t*^Bu-PONOP′)H][BAr^F^_4_] with CH_4_. Reversible Methane Activation in a SC-SC Transformation

**Figure 8:**
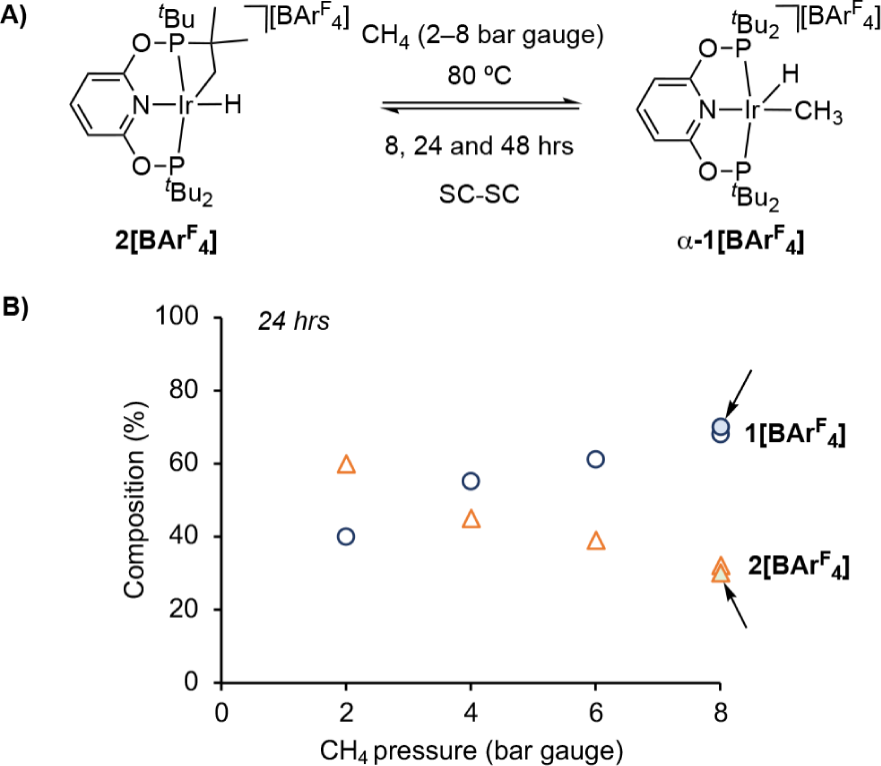
(A) Addition of CH_4_ to **2[BAr**^**F**^_**4**_**]** in the crystalline solid-state. (B) Plot of relative proportions of **α-1[BAr**^**F**^_**4**_**]** and **2[BAr**^**F**^_**4**_**]** as measured by ^1^H NMR spectroscopy (−90 °C, CD_2_Cl_2_). Solid markers, highlighted with arrows, indicate the reverse reaction (i.e., starting from **α-1[BAr**^**F**^_**4**_**]**) under the same conditions of temperature, pressure (8 bar gauge) and time.

To further test the hypothesis that reductive elimination of the cyclometalated ^t^Bu group can access a transient 14-electron [Ir(^t^Bu-PONOP)][BAr^F^_4_], **III**, intermediate *in crystallo*, finely crushed **2[BAr**^**F**^_**4**_**]** (∼5 mg) was heated to 80 °C under an atmosphere of methane (2 to 8 bar gauge) for 24 h in heavy walled/high pressure NMR tubes, Figure 8A. It was anticipated that, if formed, **III** would react rapidly with methane present in the lattice to reform **1[BAr**^**F**^_**4**_**]**. After 24 h the resulting solids were analyzed by low temperature solution NMR spectroscopy (CD_2_Cl_2_, −90 °C). This showed that **1[BAr**^**F**^_**4**_**]** is indeed formed cleanly, and that higher methane pressures result in increasing proportions of **1[BAr**^**F**^_**4**_**]** compared with **2[BAr**^**F**^_**4**_**]**: from a ratio of 40:60 at 2 bar gauge, to 68:32 at 8 bar gauge, Figure 8B. A single crystal X-ray diffraction experiment (8 bar gauge CH_4_, Figure S60) solved as a mixture of **α-1[BAr**^**F**^_**4**_**]:2[BAr**^**F**^_**4**_**]** with the methyl group (C1) refining for 55% occupancy – in good agreement with the NMR data. Shorter reaction times (8 h) resulted in reduced conversion to **1[BAr**^**F**^_**4**_**]** while longer times (48 h) were no different, showing the data at 24 h represents the equilibrium position. There was no reaction at ambient temperature.

**Figure 9:**
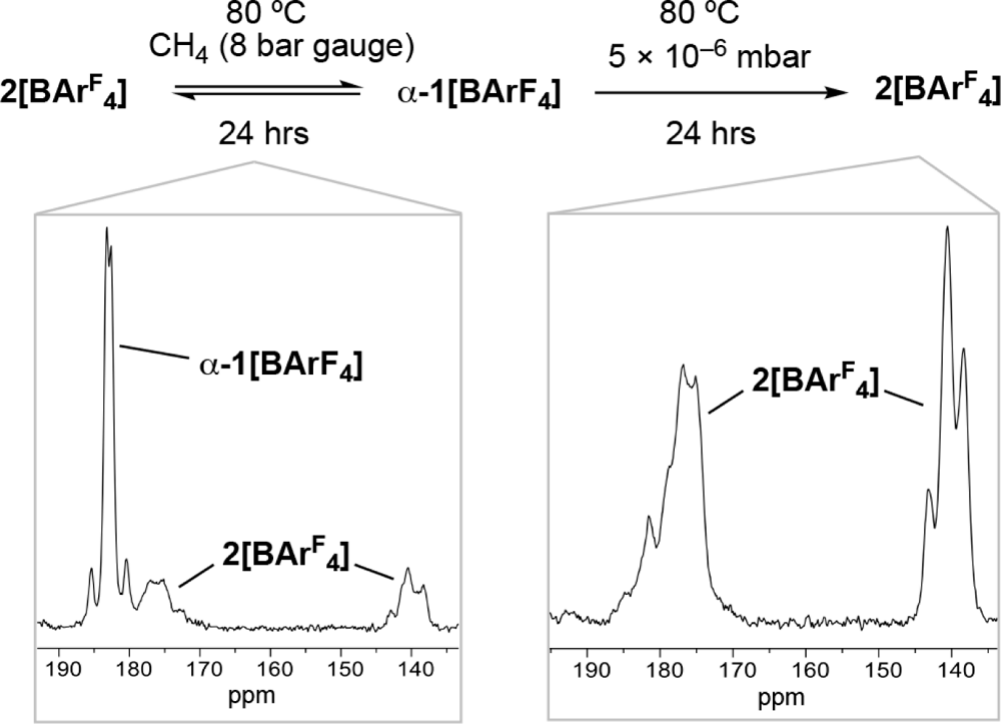
Sequential reaction of crystalline **2[BAr**^**F**^_**4**_**]** with CH_4_ (8 bar gauge) and then application of a high vacuum. Insets show ^31^P{^1^H} SSNMR spectra of each reaction.

These results suggest a dynamic, pressure-dependent, equilibrium exists between **1[BAr**^**F**^_**4**_**]** and **2[BAr**^**F**^_**4**_**]**/CH_4_ at 80 °C *in crystallo*. This hypothesis is supported by two additional observations. First, addition of 8 bar gauge CH_4_ to single crystals of **α-1[BAr**^**F**^_**4**_**]** (∼6 mg) at 80 °C results in essentially the same ratio as observed when starting with **2[BAr**^**F**^_**4**_**]** under the same conditions (Figure 8B, solid-markers). Second, addition of CH_4_ to **2[BAr**^**F**^_**4**_**]** (∼20 mg) to form an equilibrium mixture with **α-1[BAr**^**F**^_**4**_**]**, followed by application of high vacuum returns **2[BAr**^**F**^_**4**_**]**, as shown by ^31^P{^1^H} SSNMR spectra of crystalline materials after each step (Figure 9).

Reaction between CD_4_ (8 bar gauge, 24 h, 80 °C) and **2[BAr**^**F**^_**4**_**]** resulted in a ratio of **d**_**4**_**-1[BAr**^**F**^_**4**_**]:2[BAr**^**F**^_**4**_**]** that was relatively enriched in **2[BAr**^**F**^_**4**_**]** compared to the reaction with CH_4_ (56:44 versus 68:32 respectively) as measured by low temperature solution NMR spectroscopy (−90 °C, CD_2_Cl_2_) of the dissolved solids. While this is consistent with an equilibrium isotope effect operating, with D preferring to be in the C–D (i.e., CD_4_) rather than Ir–D position,^90,113^ the presence of N_2_ in the, as received, CD_4_ resulted in the formation of significant amounts of the dinitrogen adduct [Ir(^*t*^Bu-PONOP)(κ^1^-N_2_)][BAr^F^_4_] **3[BAr**^**F**^_**4**_**]** (∼10%), presumably by intercepting **III** prior to C–H (or C–D) activation.

There was no D incorporation into the ^*t*^Bu groups in the product (^2^H NMR spectroscopy, CD_2_Cl_2_) under these conditions. This was best measured by subsequent solid/gas addition of CO to the D-labeled equilibrium mixture to form a mixture of̀ the room temperature stable complexes **4[BAr**^**F**^_**4**_**]** (Figure 7) and [Ir(^*t*^Bu-PONOP)(CO)(CD_3_)D][BAr^F^_4_], the isotopologue of previously reported [Ir(^*t*^Bu-PONOP)(CO)(CH_3_)H][BAr^F^_4_].^45^ The lack of measurable H/D exchange into the ^*t*^Bu groups supports oxidative addition of methane to a 14-electron [Ir(^*t*^Bu-PONOP)][BAr^F^_4_] intermediate, rather than a σ-CAM (σ-complex assisted metathesis) mechanism, in which H/D scrambling into the ^*t*^Bu groups might be expected.^27^

### Computational Studies

These experimental observations were explored further with periodic DFT calculations. The computed structures of both **α-1[BAr**^**F**^_**4**_**]** (neutron) and **2[BAr**^**F**^_**4**_**]** show good agreement with experiment.^102^ For the **2**^**+**^ cation the location of the hydride *trans* to pyridine is confirmed and while some discrepancy is seen in the Ir–C1B distance (calc: 2.10 Å; exp: 2.04(1) Å), this likely reflects the disorder noted in the crystal structure. A weak agostic interaction is computed *trans* to the Ir–C15B bond in **2**^**+**^ (C1B–H = 1.14 Å; Ir···H = 2.07 Å), but no agostic interaction is seen in **1**^**+**^ (shortest Ir···H^*t*^Bu contact = 2.86 Å).

**Figure 10:**
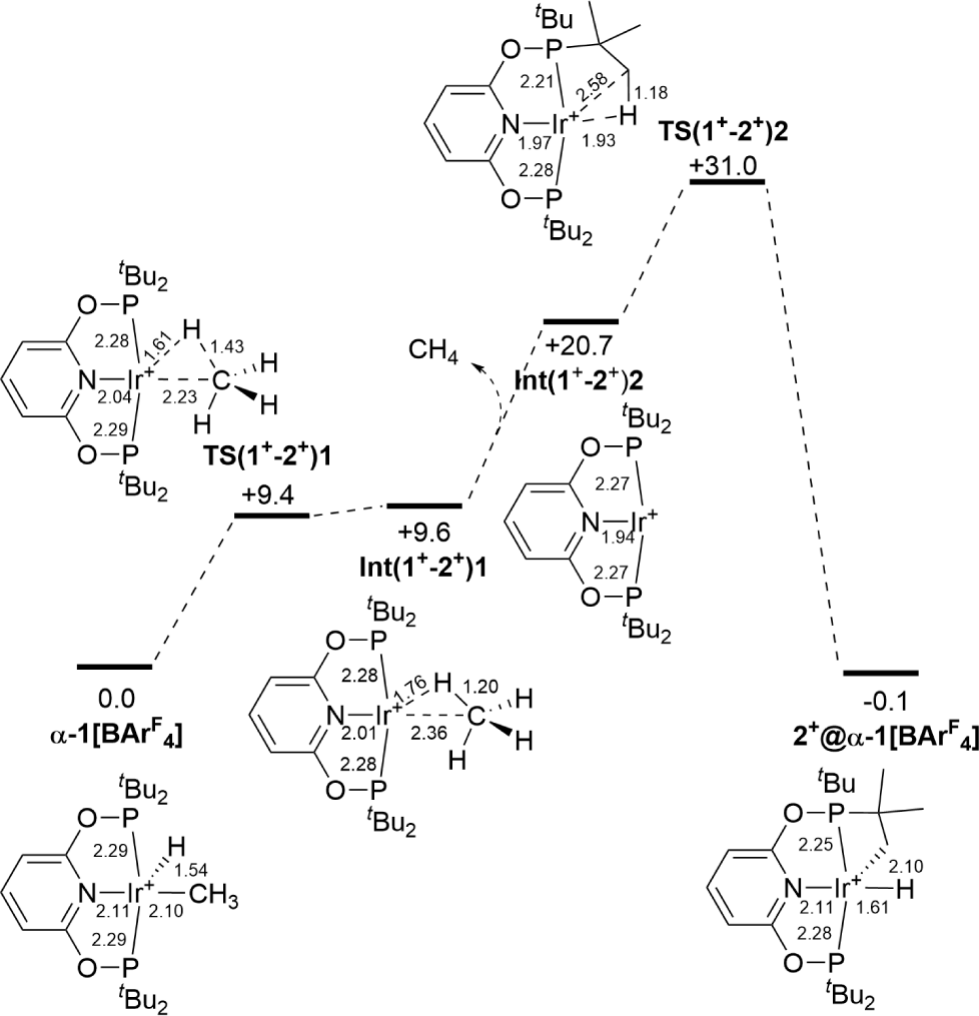
Periodic DFT free energy profile (kcal/mol) computed at 80 °C for methane loss from **α-1[BAr**^**F**^_**4**_**]** to give **2**^**+**^**@α-1[BAr**^**F**^_**4**_**].** Structures show key distances (Å) in the reacting cation; intermediate and transition state labels omit the unit cell designation for simplicity. Method: PBE-D3/DZVP-MOLOPT-SR-GTH/GTH-PBE/700 Ry cutoff.

Reactivity in the solid-state is modeled at one cation in the unit cell of interest, e.g., CH_4_ loss from **α-1[BAr**^**F**^_**4**_**]** forms one **2**^**+**^ cation within the **α-1[BAr**^**F**^_**4**_**]** unit cell (denoted **2**^**+**^@**α-1[BAr**^**F**^_**4**_**]**, see Figure 10).^114^ The most accessible pathway proceeds via reductive coupling to a CH_4_ σ-complex, **Int(1**^**+**^**–2**^**+**^**)1** at +9.6 kcal/mol followed by CH_4_ dissociation to 14e **Int(1**^**+**^**–2**^**+**^**)2** at 20.7 kcal/mol (intermediate and transition state labels omit the unit cell designation for simplicity). Cyclometalation via **TS(1**^**+**^**–2**^**+**^**)2** at 31.0 kcal/mol leads to **2**^**+**^@**α-1[BAr**^**F**^_**4**_**]** with an overall free energy change, Δ*G*, of −0.1 kcal/mol. A transition state for cyclometalation occurring directly at **Int(1**^**+**^**–2**^**+**^**)1** in which CH_4_ maintains an interaction with the Ir center was higher in energy (+38.6 kcal/mol) and other alternative pathways (via an Ir(V) intermediate, an Ir(III) σ-CH_4_ complex or concerted cyclometalation/CH_4_ loss) were also ruled out (Figures S107–S109). The naked [Ir(PONOP)]^+^ species **Int(1**^**+**^**–2**^**+**^**)2** (equivalent to 14 electron **III**) is therefore a key intermediate in the reaction from which rate-limiting C–H activation is accessed via **TS(1**^**+**^**–2**^**+**^**)2** with an overall energy span, Δ*G*^‡^, of 31.0 kcal/mol. **TS(1**^**+**^**–2**^**+**^**)2** is an early transition state with relatively little C–H bond elongation (1.18 Å) and an Ir···H distance of 1.93 Å. More significant changes involve the Ir–P–C(*^t^*Bu) angles, which evolve from 114.8°/120.0° in **Int(1**^**+**^**–2**^**+**^**)2** through 93.9°/128.9° in **TS(1**^**+**^**–2**^**+**^**)2** to 90.1°/137.1° in **2**^**+**^. The calculations also confirm that the **1**^**+**^ cation in **α**-**1[BAr**^**F**^_**4**_**]** can readily access a σ-CH_4_ complex in a rapid and reversible equilibrium that lies toward the Ir hydrido methyl form, as suggested by experiment (Figure 5).

Modeling the reverse reaction, the addition of CH_4_ (1 atm) to one of the cations in **2[BAr**^**F**^_**4**_**]** to give **1**^**+**^**@2[BAr**^**F**^_**4**_**]**, confirms the mechanism in Figure 10: initial C–H coupling forms the equivalent 14-electron **Int(2**^**+**^**–1**^**+**^**)1** followed by oxidative addition of CH_4_ (Figure S112). With this model the overall energy span, Δ*G*^‡^ (relative to **2[BAr**^**F**^_**4**_**]**), is 30.5 kcal/mol and Δ*G* = +0.8 kcal/mol. Both models therefore provide barriers commensurate with slow reactivity at 80 °C while the small Δ*G* values reflect the finely balanced equilibria evidenced in Figure 8. Alternative mechanisms, including σ-CAM processes, are again higher in energy; the proposed mechanism is therefore consistent with the exclusive formation of [Ir(^*t*^Bu-PONOP)(CD_3_)D][BAr^F^_4_] seen experimentally.

**Figure 11:**
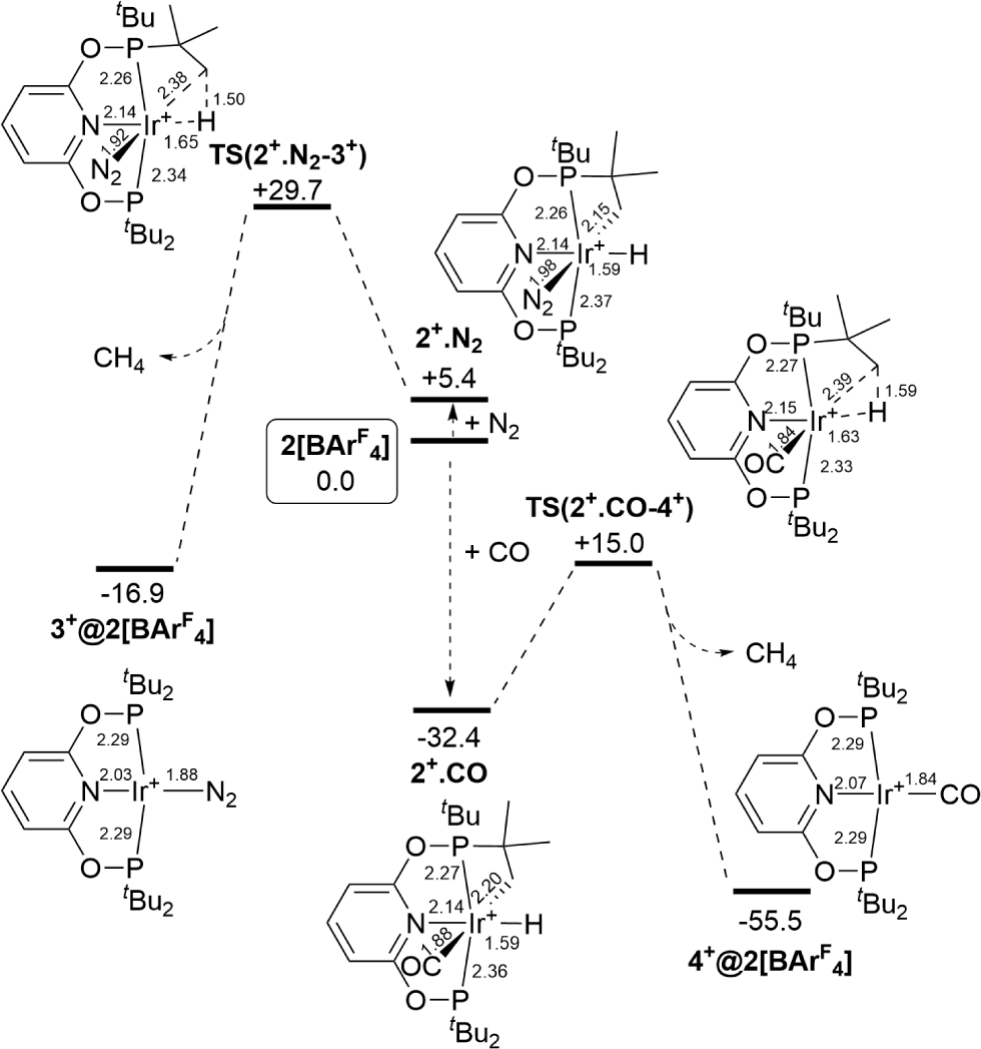
Periodic DFT free energy profile (kcal/mol) computed for the reactions of **2[BAr**^**F**^_**4**_**]** with N_2_ (1 atm at 80 °C) and CO (1 atm at 25 °C) to form **3**^**+**^**@2[BAr**^**F**^_**4**_**]** and **4**^**+**^**@2[BAr**^**F**^_**4**_**]**, respectively. Structures show key distances (Å) in the reacting cation; intermediate and transition state labels omit the unit cell designation for clarity. Method: PBE-D3/DZVP-MOLOPT-SR-GTH/GTH-PBE/700 Ry cutoff.

Computed profiles for the reactions of **2[BAr**^**F**^_**4**_**]** with N_2_ and CO are shown in Figure 11. With N_2_ a 6-coordinate adduct is formed at +5.4 kcal/mol and C–H coupling proceeds through **TS(2**^**+**^**·N**_**2**_**–3**^**+**^**)** with an overall barrier of 29.7 kcal/mol. This associative transition state has a somewhat later transition state geometry than **TS(1**^**+**^**–2**^**+**^**)2** computed in the unassisted pathway (Figure 10). Both processes have similar overall barriers (within 1 kcal/mol) suggesting the two mechanisms will be competitive. This is consistent with the similar time scales for the reactions of **2[BAr**^**F**^_**4**_**]** with CH_4_ and N_2_ observed experimentally. In contrast, CO addition to one of the cations in **2[BAr**^**F**^_**4**_**]** is strongly exergonic and first forms **2**^**+**^**·CO** at −32.4 kcal/mol. The subsequent C–H coupling proceeds with a barrier of only 17.4 kcal/mol and forms **4**^**+**^**@2[BAr**^**F**^_**4**_**]** at −55.5 kcal/mol. CO therefore plays a significant role in promoting the C–H coupling process. While N_2_ also promotes this step (the barrier from **2**^**+**^**·N**_**2**_ is 24.3 kcal/mol) its effect on the overall energy span is marginal.

### Equilibrium Isotope Effects for Reversible Methane Loss in [Ir(^*t*^Bu-PONOP)MeH][BAr^F^_4_]. Support for Rapid and Reversible Reductive Bond Formation to Form a σ-Methane Complex, Followed by a Slower Dissociative Methane Loss *In Crystallo*

**Figure 12:**
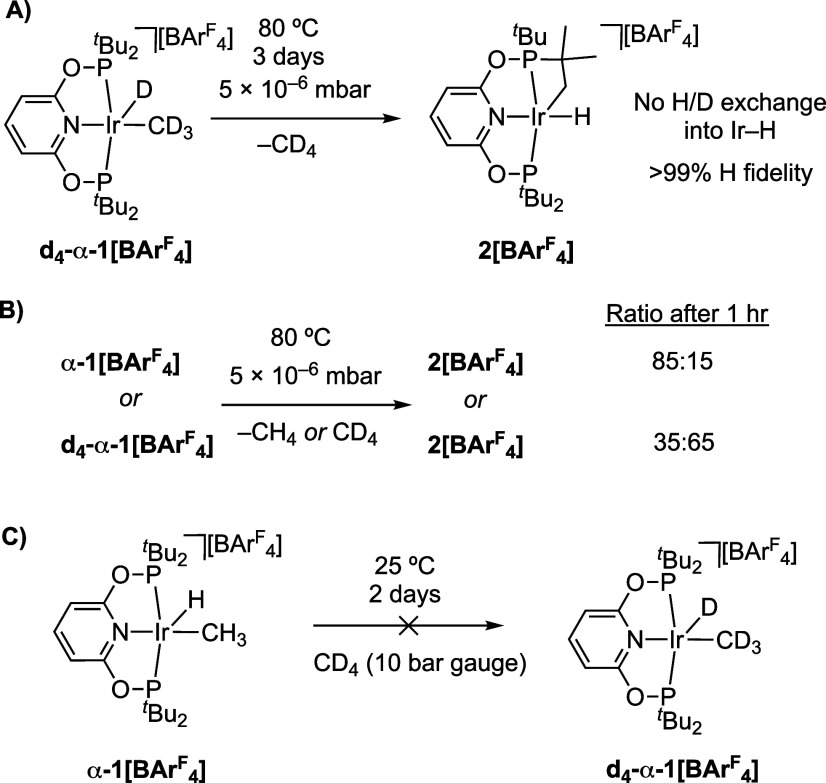
Reactivity of isotopologues of **α-1[BAr**^**F**^_**4**_**]**.

**Scheme 2:**
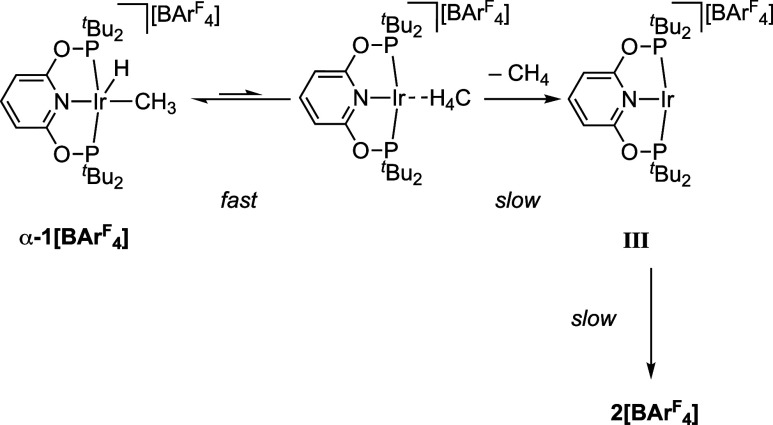
Pre-Equilibrium before CH_4_ Loss That Results in the Observed EIE (Conditions 80 °C, High Vacuum)

Experiment and periodic DFT calculations suggest the reversible, and low energy, formation of an intermediate Ir(I) σ-methane complex from **1[BAr**^**F**^_**4**_**]**, prior to a higher energy dissociative methane loss to form 14-electron [Ir(^t^Bu-PONOP)][BAr^F^_4_], **III**. To support this hypothesis experiments were performed using the isotopologue **d**_**4**_**-α-1[BAr**^**F**^_**4**_**]**, Figure 12. First, heating crystalline **d**_**4**_**-α-1[BAr**^**F**^_**4**_**]** under high vacuum at 80 °C for 3 days produces isotopically pure **2[BAr**^**F**^_**4**_**]**, with no H/D exchange into the Ir–H unit observed to the detection limit of ^1^H NMR, Figure 12A. This supports reductive elimination of CD_4_ occurring prior to cyclometalation. An alternative σ-CAM mechanism, or an Ir(V) intermediate, would be expected to result in an Ir–D bond being formed.^27^ Second, the rapid and reversible formation of an Ir(I) σ-methane complex would be expected to be signaled by an inverse isotope effect being observed for overall methane reductive elimination, i.e., the overall reaction proceeds faster with the deuterated isotopologue.^89−91^ This is because subsequent, rate-determining, dissociative loss of methane to form **III** would be expected to show only a small, if any, isotope dependence, when using **d**_**4**_**-α-1[BAr**^**F**^_**4**_**]**. An equilibrium isotope effect (EIE) arising from preceding reversible reductive bond formation from **1[BAr**^**F**^_**4**_**]** would, however, bias the pre-equilibrium toward an Ir(I) σ-methane complex for the deuterated analog, Scheme 2; as discussed for the ^13^C{^1^H} SSNMR data of **α-1[BAr**^**F**^_**4**_**]** and **d**_**4**_-**α-1[BAr**^**F**^_**4**_**]**. Heating finely crushed samples (∼14 mg) of **α-1[BAr**^**F**^_**4**_**]** and **d**_**4**_-**α-1[BAr**^**F**^_**4**_**]** side-by-side at 80 °C under high vacuum for 1 h, quenching by cooling to ambient temperature and analysis by ^1^H NMR spectroscopy (–90 °C, CD_2_Cl_2_) showed considerably more methane loss from **d**_**4**_-**α-1[BAr**^**F**^_**4**_**]** to form **2[BAr**^**F**^_**4**_**]** compared to **α-1[BAr**^**F**^_**4**_**]**, Figure 12B (65% versus 15% respectively). Related inverse isotope effects have previously been measured for the SC-SC hydrogenation, or deuteration, of [Rh(Cy_2_PCH_2_CH_2_CH_2_PCy_2_)(cyclooctadiene)][BArF_4_] to form the corresponding σ-alkane (cyclooctane) complex.^115^ Finally, addition of CD_4_ (10 bar gauge) to crystalline **α-1[BArF**_**4**_**]** at ambient temperature resulted in no exchange to form **d_4_-α-1[BArF_4_]** (or any other d-isotopologue), Figure 12C, consistent with a high barrier to dissociative loss of methane from the Ir(I) σ-methane complex.

### Stoichiometric *In Crystallo* Ethane Dehydrogenation with [Ir(cyclo-^*t*^Bu-PONOP′)H][BAr^F^_4_]

**Figure 13:**
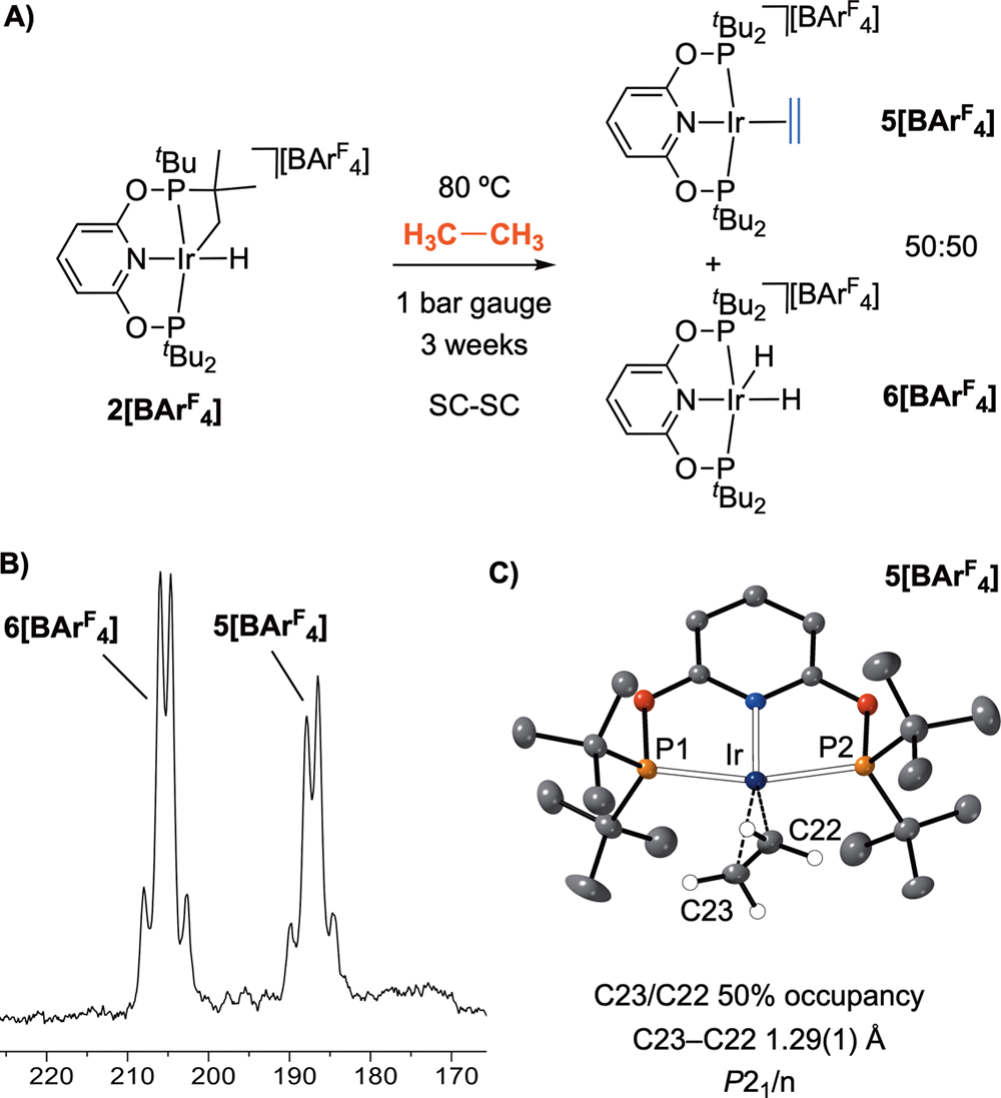
(A) Reaction of **2[BAr**^**F**^_**4**_**]** with ethane to form **5[BAr**^**F**^_**4**_**]** and **6[BAr**^**F**^_**4**_**]**. (B) ^31^P{^1^H} SSNMR of the crystalline materials post reaction. (C) Solid-state structure of the cations of the admixture of **5[BAr**^**F**^_**4**_**]** and **6[BAr**^**F**^_**4**_**]**, with the ethene ligand refined at 50% occupancy.

The *in crystallo* reaction between **2[BAr**^**F**^_**4**_**]** and CH_4_ to form the methyl hydride **α-1[BAr**^**F**^_**4**_**]** has been shown to be a finely balanced equilibrium, operating via a 14-electron intermediate, **III**. Interested in exploring alkane C–H activation with another simple substrate, the reaction of ethane with crystalline **2[BAr**^**F**^_**4**_**]** was studied, under similar conditions to those used for methane activation. Heating **2[BAr**^**F**^_**4**_**]** for 3 weeks under 1 bar gauge of ethane did not, however, result in the isolation of the corresponding ethyl hydride complex. Instead the known^116^ ethene and dihydride complexes, [Ir(^*t*^Bu-PONOP)(η^2^-H_2_C=CH_2_)][BAr^F^_4_] **5[BAr**^**F**^_**4**_**]** and [Ir(^*t*^Bu-PONOP)H_2_][BAr^F^_4_] **6[BAr**^**F**^_**4**_**]** are formed, Figure 13A.

Solution NMR data for this mixture are the same as previously reported for the individual components,^116^ and show **5[BAr**^**F**^_**4**_**]** and **6[BAr**^**F**^_**4**_**]** are formed in a ∼50:50 ratio. In the ^31^P{^1^H} SSNMR NMR spectrum the two complexes are observed in approximately equal amounts, each as a tightly coupled AB doublet, characteristic of the α-crystal form, Figure 13B. A single-crystal X-ray diffraction study shows that this transformation is SC-SC, solving as a 50:50 superposition of the two cations in the characteristic *P*2_1_/*n* arrangement of [BAr^F^_4_]^−^ anions, Figure 13C. The ethene ligand in **5[BAr**^**F**^_**4**_**]** refines at 50% occupancy, while the hydride ligands in **6[BAr**^**F**^_**4**_**]** were not located.

**Scheme 3:**
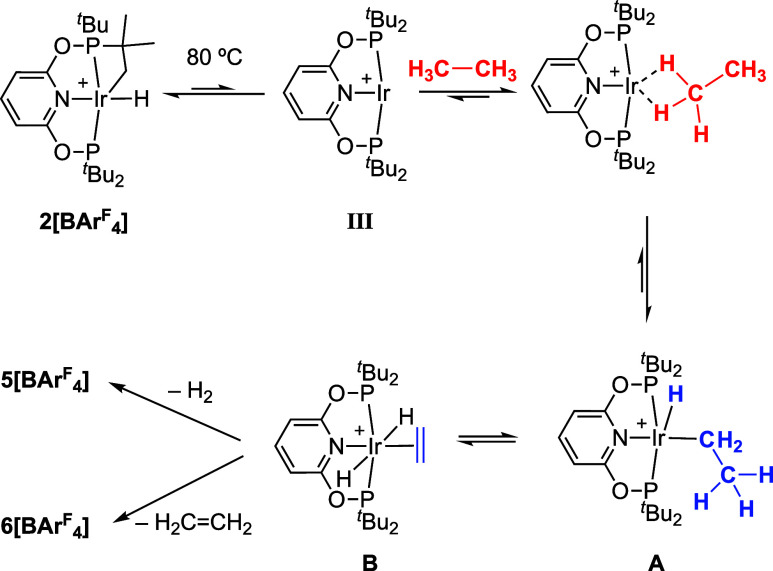
Proposed Mechanism for the Dehydrogenation of Ethane *In Crystallo*; [BAr^F^_4_]^−^ Anions Are Not Shown

The proposed mechanism of formation of this mixture is shown in Scheme 3. Reductive elimination in the cyclometalated ligand at 80 °C reversibly forms the high energy intermediate **III** that reacts with lattice-available ethane through C–H activation to initially form an Ir(III) ethyl hydride, **A**. β-hydrogen transfer then forms a dihydride ethene complex, **B**. If the barriers to loss of ethene or H_2_ (via an isomerization in **B** to place the hydrides *cis* to one another) were competitive in the single crystal this would lead to the admixture of **5[BAr**^**F**^_**4**_**]** and **6[BAr**^**F**^_**4**_**]**. *In crystallo* H_2_ loss from a complex closely related to **B**, [Ir(^i^Pr-PONOP)(η^2^-H_2_C=CHMe)H_2_][BAr^F^_4_], has recently been reported, alongside competitive loss of H_2_ or alkene in solution.^76^ Further support for this mechanism is provided by the *in situ* observation of the ethyl hydride complex [Ir(^*t*^Bu-PONOP)(H)(CH_2_CH_3_)][NTf_2_] (i.e., the cation in **A**) in solution at very low temperature (−100 °C) using NMR spectroscopy,^46^ which undergoes rapid site exchange between bound C–atoms. DFT calculations suggest this occurs via a β-hydrogen transfer process operating via **B**.

The reaction of **2[BAr**^**F**^_**4**_**]** with ethane thus represents an *in crystallo* stoichiometric alkane dehydrogenation to form ethene and H_2_, both of which are trapped in the resulting crystalline products, **5[BAr**^**F**^_**4**_**]** and **6[BAr**^**F**^_**4**_**]** respectively. This result adds further support for the formation of a transient 14-electron intermediate, **III**, from reductive elimination from operationally unsaturated **2[BAr**^**F**^_**4**_**]**, that is irreversibly trapped by reaction with ethane. The slower reaction with ethane compared with methane (3 weeks versus 24 h) is likely a consequence of decreased lattice mobility of the larger ethane, similar to the selectivity observed for the *in crystallo* hydrogenation of ethene over propene using Ir(^*t*^Bu-POCOP)(κ^1^-N_2_).^78^

## Conclusions

The coordination and C–H activation of alkanes at transition metal centers relies on the generation of reactive, operationally unsaturated, intermediates in solution. However, such intermediates are in competition with solvent binding, the vast excess of which means that coordination and activation of simple alkanes is challenging. By working *in crystallo*, solvent is removed from the equation, and impediments to alkane binding, C–H activation or overall decomposition are thus attenuated. By generating the operationally unsaturated complexes, **1[BAr**^**F**^_**4**_**]** and cyclometalated **2[BAr**^**F**^_**4**_**]**, that function with the retention of crystallinity, methane and ethane activation at a 14-electron Ir(I) center occurs under relatively mild conditions. Such reactivity demonstrates the advantages that synthesis and reactivity in the molecular solid-state provides, especially for the generation of highly reactive intermediates. Extending this to suitable single-crystalline systems that can catalytically activate alkanes is a clear next challenge.

## Supporting Information Available

NMR spectra, experimental and characterization data for the compounds reported including single-crystal X-ray diffraction studies, and coordinates and energies of computed stationary points (PDF)
Computed geometries (.xyz) as a combined file (ZIP)
