# Supplementary material for: An Operationally Unsaturated Iridium-Pincer Complex That C–H Activates Methane and Ethane in the Crystalline Solid-State
Source: J Am Chem Soc. 2025 Feb 25;147(10):8706–19. doi: 10.1021/jacs.4c18122 (PMC11912492; doi:10.1021/jacs.4c18122)
Supplement: Supplementary file 1 — ja4c18122_si_001.pdf [file ja4c18122_si_001.pdf]

Supporting Information for

An Operationally Unsaturated Iridium-Pincer Complex that C–H Activates Methane and Ethane in the Crystalline Solid-State

Matthew R. Gyton,[a] M. Arif Sajjad,[b] Daniel J. Storm,[b] Kristof M. Altus,[a] Joe C. Goodall,[a] Chloe L. Johnson,[a] Samuel J. Page,[c] Alison J. Edwards,[d] Ross O. Piltz,[d] Simon B. Duckett,[a*] Stuart A. Macgregor,[b*] Andrew S. Weller[a*]

[a] Department of Chemistry, University of York, Heslington, York, YO10 5DD, UK

[b] EaSTCHEM School of Chemistry, University of St Andrews, North Haugh, St Andrews, KY16 9ST, UK

[c] Department of Chemistry, University of Durham, Durham, DH1 3LE, UK

[d]Australian Centre for Neutron Scattering, Australian Nuclear Science and Technology Organisation, Lucas Heights, NSW, 2234, Australia.

Table of Contents

General Procedures ... 3
Synthesis ... 5
Preparation of [Ir(^{t}Bu-PONOP)MeH][BAr$^F_4$] 1[BAr$^F_4$] ... 5
Data for [Ir(^{t}Bu-PONOP)MeH][BAr$^F_4$] ... 5
Solid-State Stability of [Ir(^{t}Bu-PONOP)MeH][BAr$^F_4$] ... 9
Stability of [Ir(^{t}Bu-PONOP)MeH][BAr$^F_4$] stored under D_2O ... 11
Solution-State Stability of [Ir(^{t}Bu-PONOP)HMe][BAr$^F_4$] ... 13
Preparation of [Ir(^{t}Bu-PONOP)CD_3] ... 15
Data for [Ir(^{t}Bu-PONOP)CD_3] ... 15
Preparation of Solutions of Anhydrous Deuterium Chloride ... 17
Titration of Deuterium Chloride Solution ... 18
Preparation of [D(OEt_2)$_2$][BAr$^F_4$] ... 18
Data for [D(OEt_2)$_2$][BAr$^F_4$] ... 19
Preparation of [Ir(^{t}Bu-PONOP)(CD_3)D][BAr$^F_4$] ... 20
Data for [Ir(^{t}Bu-PONOP)(CD_3)D][BAr$^F_4$] ... 20
Preparation of [Ir(cyclo-^{t}Bu-PONOP')H][BAr$^F_4$] ... 23
Data for [Ir(cyclo-^{t}Bu-PONOP')H][BAr$^F_4$] ... 23
Characterisation of [Ir(cyclo-^{t}Bu-PONOP')H(MeCN)][BAr$^F_4$] ... 27
Data for [Ir(cyclo-^{t}Bu-PONOP')H(MeCN)][BAr$^F_4$] ... 27
Preparation of [Ir(PONOP^{t}Bu)(N_2)][BAr$^F_4$] ... 28
Data for [Ir(^{t}Bu-PONOP)(κ^1-N_2)][BAr$^F_4$] ... 29
Solid/Gas Reaction of [Ir(cyclo-^{t}Bu-PONOP')H][BAr$^F_4$] with Carbon Monoxide ... 32
Variable Pressure Solid/Gas Reactions of [Ir(cyclo-^{t}Bu-PONOP')H][BAr$^F_4$] with CH_4 ... 35
Spectroscopic Data After 8 Hours ... 36
Spectroscopic Data After 24 Hours ... 37
Solid/Gas Reaction of [Ir(^{t}Bu-PONOP)MeH][BAr$^F_4$] with CH_4 ... 40
Reversibility of Methane Oxidative Addition and Reductive Elimination ... 41
Solid/Gas Reaction of [Ir(cyclo-^{t}Bu-PONOP')H][BAr$^F_4$] with CD_4 ... 43
Preparation of [Ir(cyclo-^{t}Bu-PONOP')H][BAr$^F_4$] from [Ir(^{t}Bu-PONOP)(CD_3)D][BAr$^F_4$] ... 47
Investigation of Equilibrium Isotope Effects through NMR Scale Vacuum Thermolysis ... 48
Attempted Solid/Gas CH_4/CD_4 Exchange at Room Temperature ... 51
Solid/Gas Reaction of [Ir(cyclo-^{t}Bu-PONOP')H][BAr$^F_4$] with C_2H_6 ... 53
Single Crystal Diffraction ... 56
X-ray Diffraction ... 56
Neutron Diffraction ... 56
Computational Details ... 67
Solid-State Calculations ... 67
Molecular Calculations ... 67
Methane Loss from α-1[BAr$^F_4$] ... 69
Isolated Cation Model ... 69
Solid-State Model ... 71
Methane Loss from α-1[BAr$^F_4$] to form 2^+@α-1[BAr$^F_4$] ... 71
Reaction of 2[BAr$^F_4$] with CH_4 to form 1^+@2[BAr$^F_4$]. ... 77
Reactions of 2[BAr$^F_4$] with N_2 and CO. ... 81
Isolated Cation Model ... 81
Solid-State Model ... 82
References ... 84

General Procedures

All manipulations, unless otherwise stated, were performed under an inert atmosphere (argon or nitrogen, BOC, N4.8 purity) using standard Schlenk line and glovebox (<0.1 ppm H_2O/O_2) techniques. Glassware was oven-dried at 150 °C overnight and flame dried under vacuum prior to use. All solvents were degassed by three successive freeze-pump-thaw cycles and stored over activated 3 Å molecular sieves under inert gas in resealable glass ampoules fitted with PTFE high vacuum stopcocks (Kontes Hi-Vac, J. Young or Rotaflo HP). Dichloromethane, pentane and hexane were dried using commercially available solvent drying systems (Innovative Technologies/Inert or MBraun) by passage through stainless-steel columns packed with activated alumina.[1] Heptane was purchased anhydrous from Sigma-Aldrich and decanted by cannula into resealable glass ampoules, degassed by three successive freeze-pump-thaw cycles and stored over activated 3 Å molecular sieves. 1,2-$C_6H_4F_2$ (pre-dried by stirring over activated alumina) was distilled from CaH_2 under dinitrogen followed by storage as above. CD_2Cl_2 was stored over two successive batches of activated molecular sieves.

^{t}Bu-PONOP,[1] [Ir(^{t}Bu-PONOP)Cl],[2] [Ir(^{t}Bu-PONOP)Me],[2] Na[BAr$^F_4$][3] and [H(OEt$_2$)$_2$][BAr$^F_4$][4] were prepared by literature methods. Carbon monoxide (CK Gas Products, N3.7), CH_4 (CK Gas Products, N5.5) and C_2H_6 (Air Liquide, N2.5) were used as received. CD_4 (Cambridge Isotope Laboratories, N2.5, 99% D) was purified by passage through a short ¼" OD stainless-steel column packed with activated 3 Å molecular sieves and Pd/C (5% w/w, outgassed under high vacuum) to remove H_2O and H_2/D_2 respectively. High pressure experiments were conducted in 5 mm OD heavy-walled glass NMR tubes (Quick Pressure Valve, Wilmad Labglass) connected by a 10-32 threaded connection (Swagelok SS-200-1-0157) to a stainless-steel vacuum/inert manifold. Glass under pressure was only handled behind a polycarbonate safety shield with appropriate PPE (face shield, safety glasses, Kevlar gloves and hearing protection).

All solution phase NMR samples were prepared on a greaseless high vacuum line (<1 x 10^{-5} mbar) by condensation of the solvent under static vacuum onto solid samples in 5 mm thin-wall NMR tubes fitted with high vacuum PTFE (J. Young) valves. Solution phase NMR spectra were recorded on Bruker Avance III HD 500 MHz spectrometers using a TBO probe (^{1}H = 500.22 MHz, ^{2}H = 76.79 MHz ^{13}C = 125.80 MHz, ^{31}P = 202.50 MHz), or BBI probe (^{1}H = 500.12 MHz, ^{31}P = 202.47 MHz), Bruker Avance III HD 600 MHz spectrometer using a BBO probe (^{1}H = 600.09 MHz, ^{13}C = 150.91 MHz, ^{31}P = 242.93 MHz), a Bruker Avance III 400 MHz spectrometer using a BBI probe (^{1}H = 400.12 MHz, ^{31}P = 162.00 MHz) or Bruker Avance NEO (^{1}H = 400.11 MHz, ^{2}H = 61.42 MHz, ^{31}P = 162.00 MHz) at 298 K unless otherwise specified. Residual proteo solvent was used as the reference for ^{1}H and ^{13}C{^{1}H} spectra in deuterated solvent samples.[5] ^{2}H NMR are referenced using the standard frequency ratio for the ^{2}H nucleus (Ξ = 15.350609%)[6] relative to a ^{1}H NMR spectrum recorded concomitantly and referenced as above. All chemical shifts (δ) are quoted in ppm and coupling constants (J) in Hz. NMR assignments were aided by ^{1}H{^{31}P} and 2D experiments (^{1}H-^{1}H-COSY, ^{1}H-^{13}C-HSQC, ^{1}H-^{13}C-HMBC) where required.

Gas phase NMR spectra were recorded on a Bruker Avance III HD 500 MHz spectrometer using a TBO probe (^{1}H = 500.22 MHz) at 298 K in 5 mm thin-wall NMR tubes fitted with high vacuum PTFE (J. Young) valves.

Solid state NMR spectra were recorded on a Bruker wide-bore Avance III HD spectrometer (^{13}C = 100.63 MHz; ^{31}P = 162.00 MHz) in 4.0 mm zirconia rotors with a MAS rate of 10 kHz or a Bruker wide-bore Avance III HD spectrometer (^{13}C = 100.66 MHz; ^{31}P = 162.06 MHz) in 2.5 mm zirconia rotors with a MAS rate of 20 kHz. Rotors were packed and sealed with Kel-F caps (4.0 mm) or Vespel (4.0 mm variable temperature, 2.5 mm ambient temperature) caps in an argon filled glovebox. Spectra are referenced externally to $SiMe_4$ or H_3PO_4 using the secondary references adamantane (^{13}C δ = 29.5 for the shielded methylene resonance)[7] or triphenylphosphine (^{31}P, δ = −9.3).[8]

FT-IR spectra were recorded on a Bruker Alpha spectrometer operating with the Platinum ATR accessory in an argon glovebox. Elemental microanalyses were performed by Orla McCullough at London Metropolitan University using a Thermo Scientific FLASH 2000 CHN-O Analyzer. Electrospray Ionisation Mass Spectrometry (ESI-MS) was carried out using a Bruker compact Time-of-Flight mass spectrometer by Mr Karl Heaton at the University of York.

Synthesis

Preparation of [Ir(^{t}Bu-PONOP)MeH][BAr$^{F}_{4}$] 1[BAr$^{F}_{4}$]

Adapted from the published literature procedure[2] by variation of the solvent from CH_2Cl_2 to 1,2-difluorobenzene. This affords solvent-free crystalline material in bulk as opposed to the published method which affords [Ir(^{t}Bu-PONOP)MeH][BAr$^{F}_{4}$]·CH_2Cl_2.

A freshly prepared, cold (~−40 °C) solution of [H(OEt_2)$_2$][BAr$^{F}_{4}$] (1.005 g, 992.6 µmol) in 1,2-difluorobenzene (~25 mL) was added to a cold (~−40 °C) deep-red solution of [Ir(^{t}Bu-PONOP)Me] (603.4 mg, 993.0 µmol), also in 1,2-difluorobenzene (~25 mL) and stirred at −30 °C for 30 minutes. Volatiles from the resulting orange solution were removed *in vacuo*, the solid material redissolved in fresh, cold (~−30 °C) 1,2-difluorobenzene, layered with excess heptane (~250 mL) and stored at −40 °C for several weeks to allow crystallisation by slow diffusion. The supernatant was decanted away, the isolated crystals were washed with hexane and dried *in vacuo* to afford [Ir(^{t}Bu-PONOP)MeH][BAr$^{F}_{4}$] **1[BAr$^{F}_{4}$]** as two polymorphs of orange blocks **α-1[BAr$^{F}_{4}$]** and orange rods **β-1[BAr$^{F}_{4}$]** (1.387 g, 943.0 µmol, 95%).

Phase purity was ensured by sieving the dried crystals through stainless-steel sieves with defined pore sizes (Endecotts Ltd, 1.0 mm, 0.5 mm and 0.25 mm; Figure S1) in an argon glovebox, retaining crystalline material >0.25 mm as **α-1[BAr$^{F}_{4}$]** exclusively (1.334 g, 906.8 µmol, 91%).

All crystal-to-crystal transformations from **1[BAr$^{F}_{4}$]** used material 0.25-0.5 mm.

Data for [Ir(^{t}Bu-PONOP)MeH][BAr$^{F}_{4}$]

^{1}H NMR (500.23 MHz, CD_2Cl_2, 203 K) δ 7.82 (t, $^{3}J_{HH}$ = 8, 1H, py) 7.71 (s, 8H, Ar$^{F}_{4}$), 7.53 (s, 4H, Ar$^{F}_{4}$), 6.98 (d $^{3}J_{HH}$ = 8, 2H, py), 1.85 (s br, 3H, IrMe), 1.23 (vt, J = 7, P^{t}Bu$_2$), −41.85 (br, 1H, IrH).

^{31}P{^{1}H} NMR (202.50 MHz, CD_2Cl_2, 203 K) δ 184.3.

^{13}C{^{1}H} CP MAS NMR (10 kHz spin rate, 100.63 MHz, 298 K) δ 165.9-163.8 (Ar^{F}), 162.1-161.6 (py *C*),142.2 (py *C*H), 136.7-116.4 (Ar^{F}), 104.3-102.7 (py *C*H), 43.0-40.4 (P*C*(CH_3)$_3$), 27.0-25.5 (PC(*C*H_3)$_3$), −22.8 (Ir*C*H_3).

^{31}P{^{1}H} CP MAS NMR (20 kHz spin rate, 162.04 MHz, 298 K) δ 184.2 (d, J_{PP} = 330, P^{t}Bu$_2$), 181.5 (d, J_{PP} = 342, P^{t}Bu$_2$).

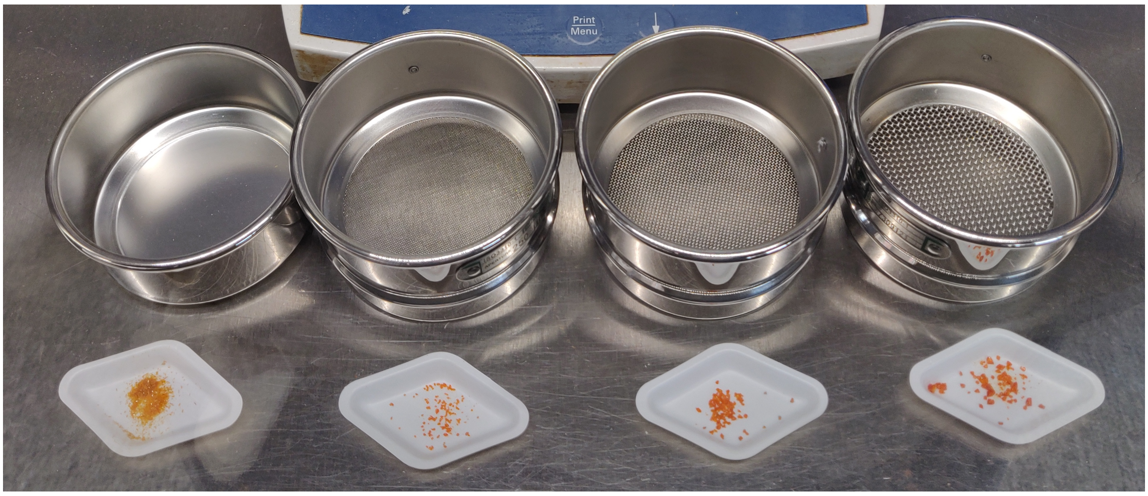

Figure S1. *Photograph showing the crystal size distributions obtained after sieving recrystallised [Ir(^{t}Bu-PONOP)MeH][BAr$^{F}_{4}$]* ***1[BAr$^{F}_{4}$]*** *through (from right to left) 1, 0.5 and 0.25 mm sieves.*

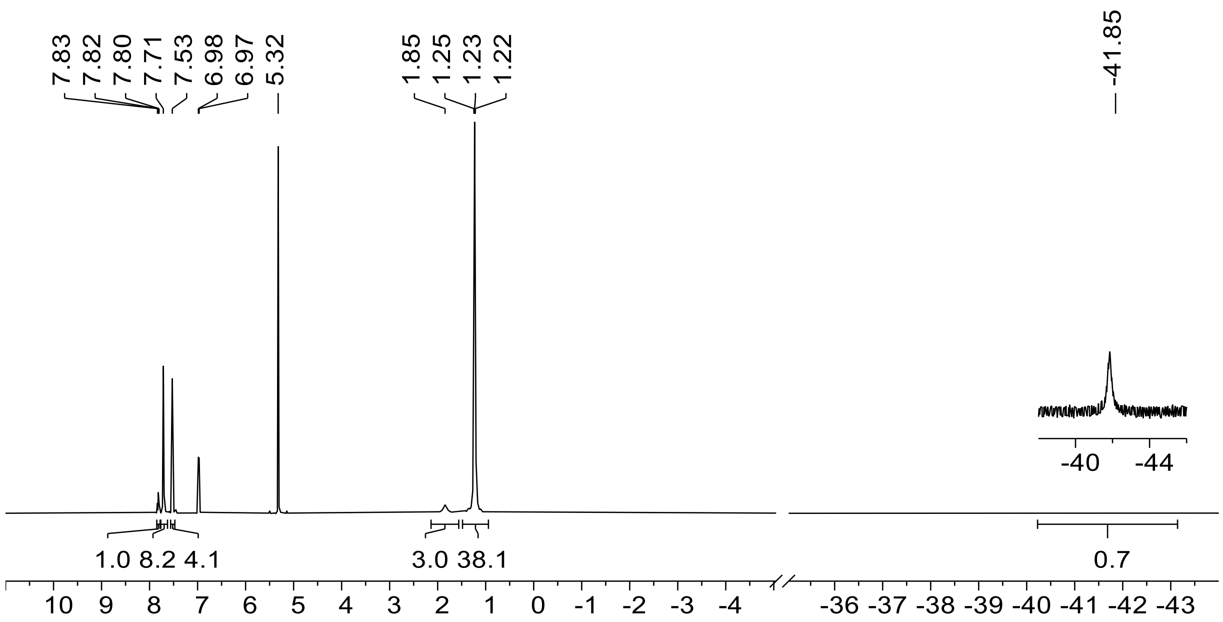

Figure S2. *^{1}H NMR Spectrum of [Ir(^{t}Bu-PONOP)MeH][BAr$^F_4$]* ***1[BAr$^F_4$]*** *(500.22 MHz, CD_2Cl_2, 203 K).*

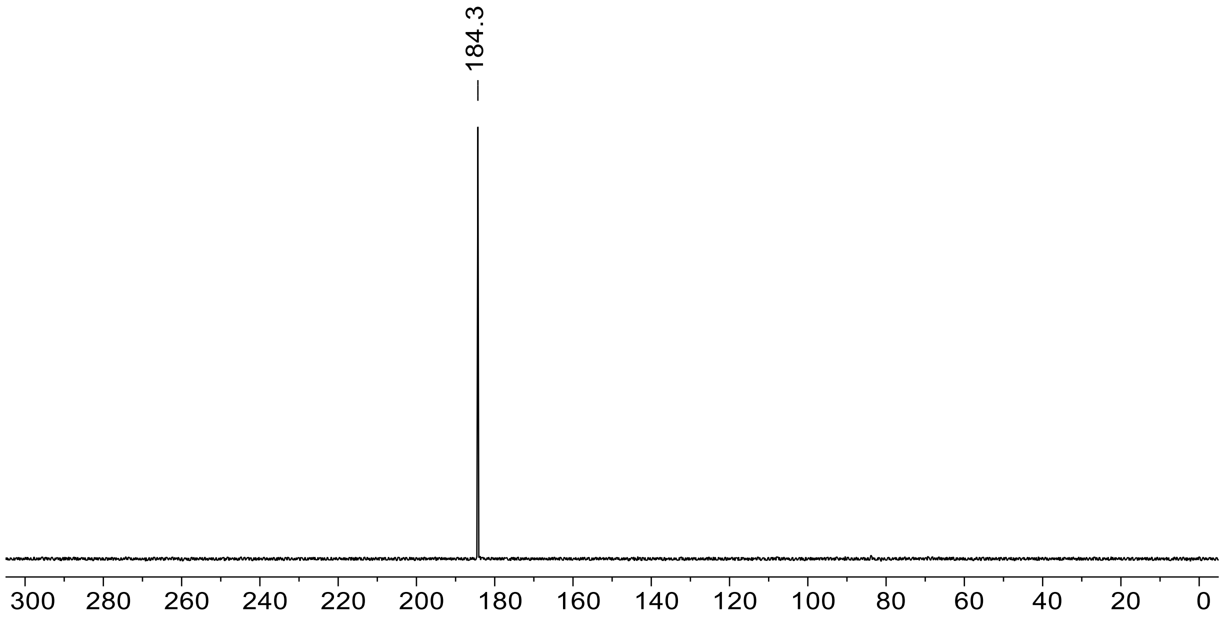

Figure S3. *^{31}P{^{1}H} NMR Spectrum of [Ir(^{t}Bu-PONOP)MeH][BAr$^F_4$]* ***1[BAr$^F_4$]*** *(202.53 MHz, CD_2Cl_2, 203 K).*

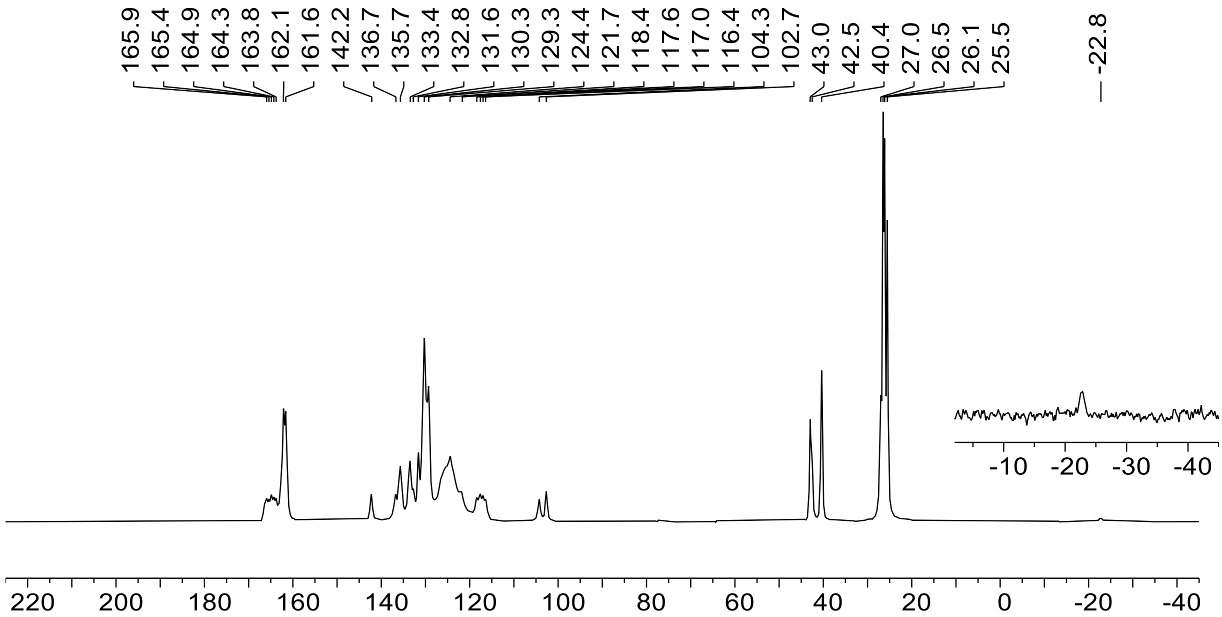

Figure S4. *$^{13}C\{^{1}H\}$ CPTOSS MAS NMR spectrum of [Ir(^{t}Bu-PONOP)MeH][BAr$^{F}_{4}$]* ***1[BAr$^{F}_{4}$]*** *crystals >0.25 mm containing* ***α-1[BAr$^{F}_{4}$]*** *only (10 kHz spin rate, 100.63 MHz, 298 K).*

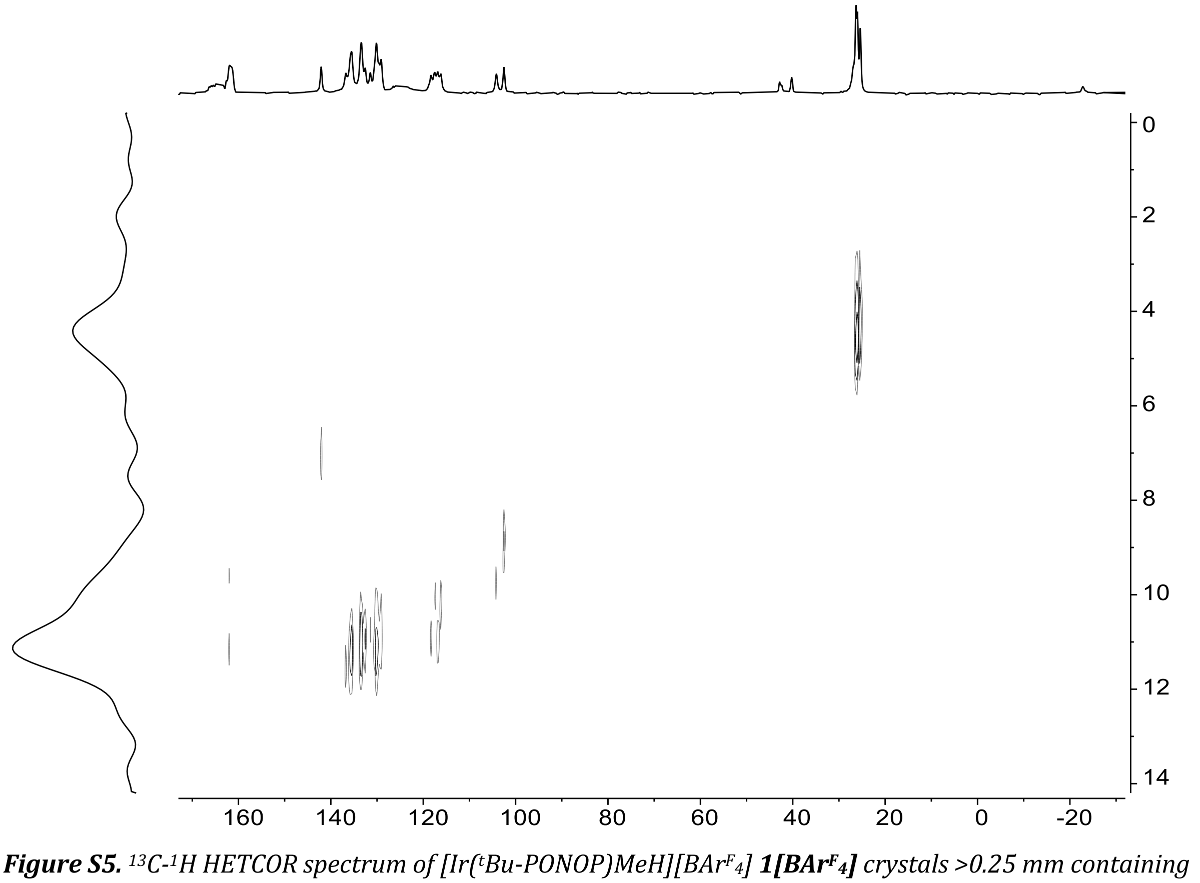

Figure S5. *^{13}C-^{1}H HETCOR spectrum of [Ir(^{t}Bu-PONOP)MeH][BAr$^{F}_{4}$]* ***1[BAr$^{F}_{4}$]*** *crystals >0.25 mm containing* ***α-1[BAr$^{F}_{4}$]*** *only (10 kHz spin rate, 100.63 MHz, 298 K).*

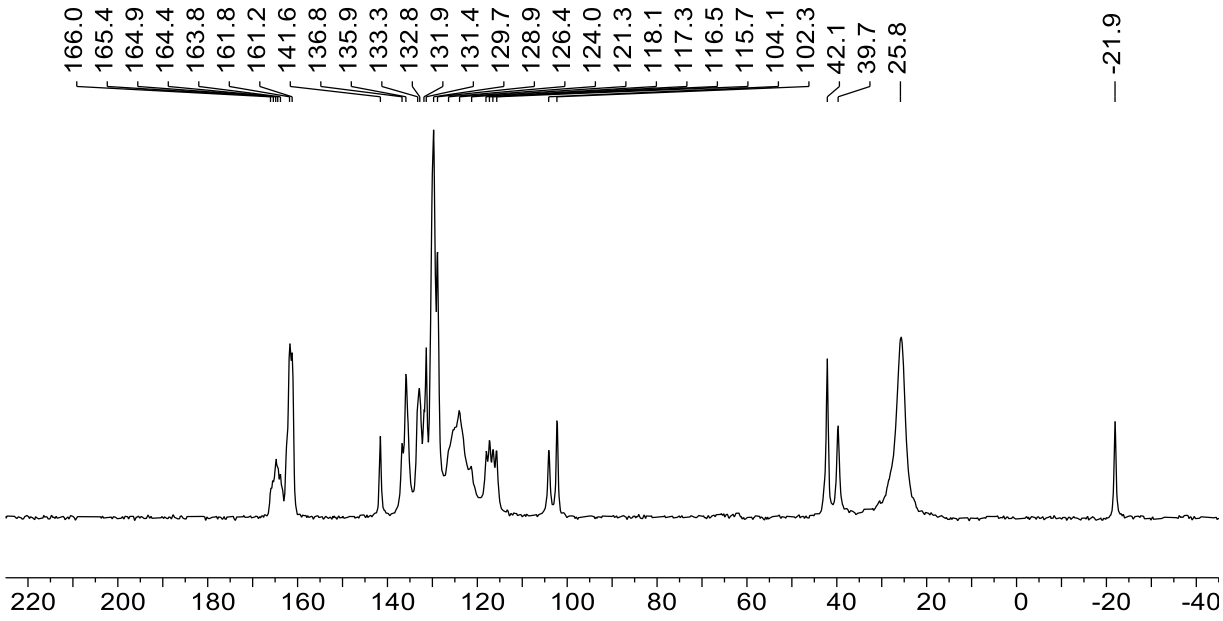

Figure S6. *$^{13}C\{^{1}H\}$ CPTOSS MAS NMR spectrum of [Ir(^{t}Bu-PONOP)MeH][BAr$^{F}{}_{4}$]* ***1[BAr$^{F}{}_{4}$]*** *crystals >0.25 mm containing* ***α-1[BAr$^{F}{}_{4}$]*** *only (10 kHz spin rate, 100.63 MHz, 193 K).*

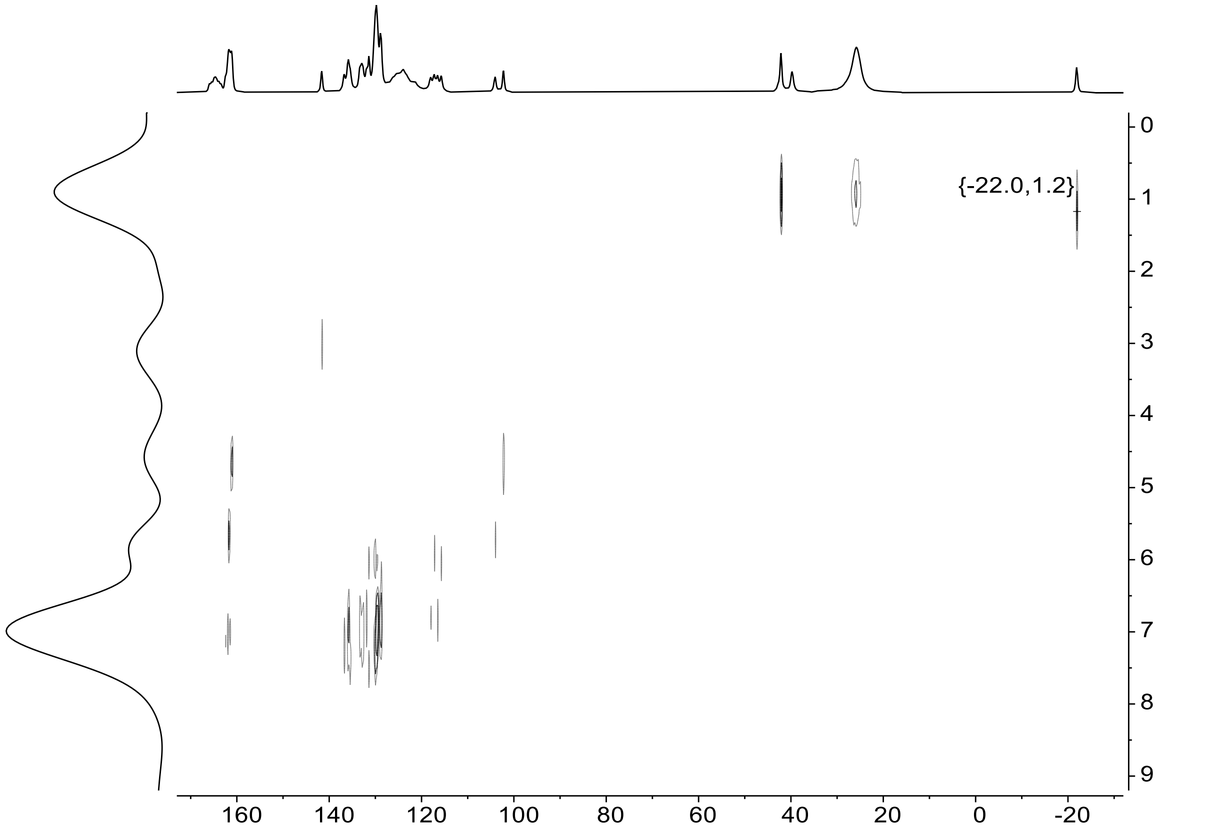

Figure S7. *^{13}C-^{1}H HETCOR spectrum of [Ir(^{t}Bu-PONOP)MeH][BAr$^{F}{}_{4}$]* ***1[BAr$^{F}{}_{4}$]*** *crystals >0.25 mm containing* ***α-1[BAr$^{F}{}_{4}$]*** *only (10 kHz spin rate, 100.63 MHz, 193 K).*

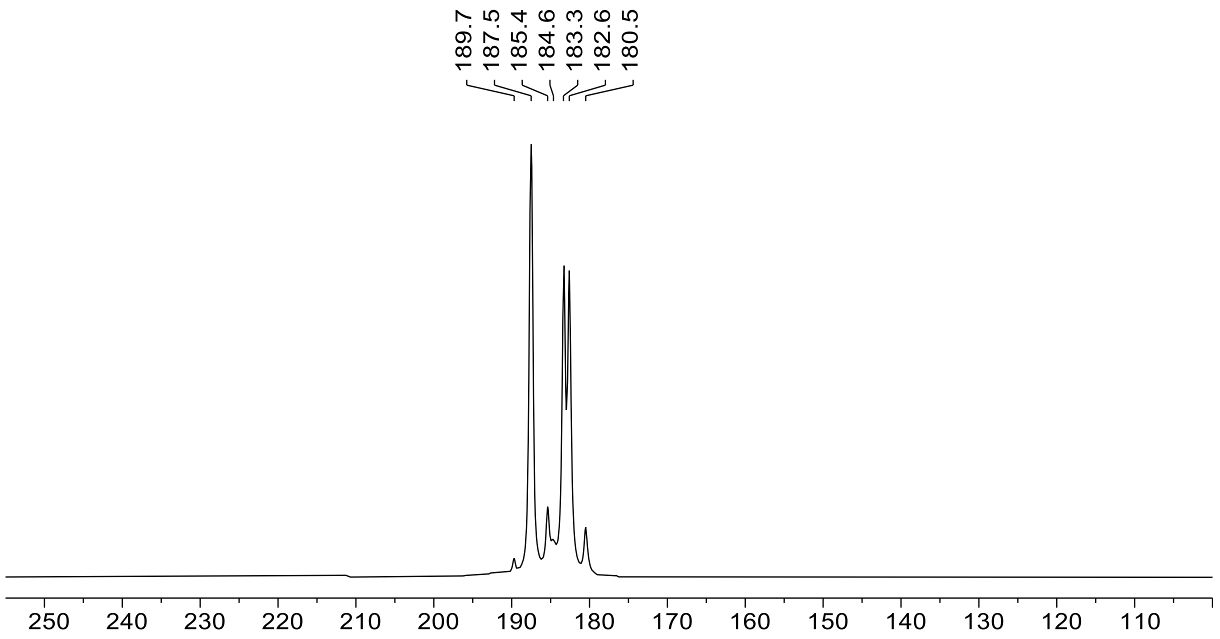

Figure S8. *$^{31}P\{^{1}H\}$ HPDEC MAS NMR spectrum of [Ir(^{t}Bu-PONOP)MeH][BAr$^{F}_{4}$]* ***1[BAr$^{F}_{4}$]*** *crystals <0.25 mm containing both* ***α-1[BAr$^{F}_{4}$]*** *and* ***β-1[BAr$^{F}_{4}$]*** *(20 kHz spin rate, 162.06 MHz, 298 K).*

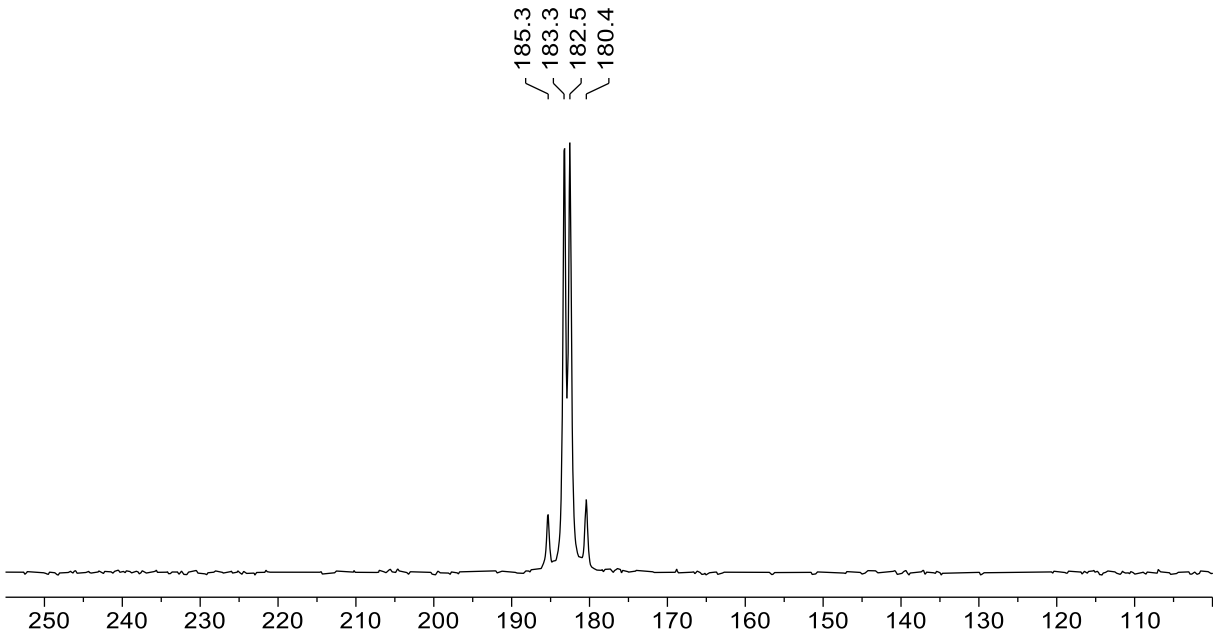

Figure S9. *$^{31}P\{^{1}H\}$ CP MAS NMR spectrum of [Ir(^{t}Bu-PONOP)MeH][BAr$^{F}_{4}$]* ***1[BAr$^{F}_{4}$]*** *crystals >0.25 mm containing* ***α-1[BAr$^{F}_{4}$]*** *only (20 kHz spin rate, 162.06 MHz, 298 K).*

Solid-State Stability of [Ir(^{t}Bu-PONOP)MeH][BAr$^{F}_{4}$]

Single crystals of [Ir(^{t}Bu-PONOP)MeH][BAr$^{F}_{4}$] **α-1[BAr$^{F}_{4}$]** (6.7 mg, 4.6 µmol) in a 5 mm thin-wall valved NMR tube under argon were stored in a temperature-controlled water-bath at 25-26 °C for 7 days. The headspace was monitored by gas-phase ^{1}H NMR and was featureless to the detection limit of the spectrometer. The crystals were then dissolved by condensation of CD_2Cl_2 *in vacuo* and assayed by ^{1}H and $^{31}P\{^{1}H\}$ NMR spectroscopy at 193 K. Spectra were consistent with unchanged **1[BAr$^{F}_{4}$]**

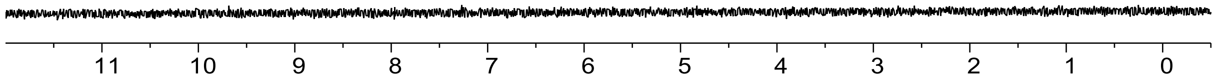

Figure S10. *^{1}H NMR spectrum of the headspace above single crystals of [Ir(^{t}Bu-PONOP)MeH][BAr$^{F}_{4}$]* ***1[BAr$^{F}_{4}$]*** *stored at ambient temperature for seven days (500.22 MHz, gas phase, 298 K).*

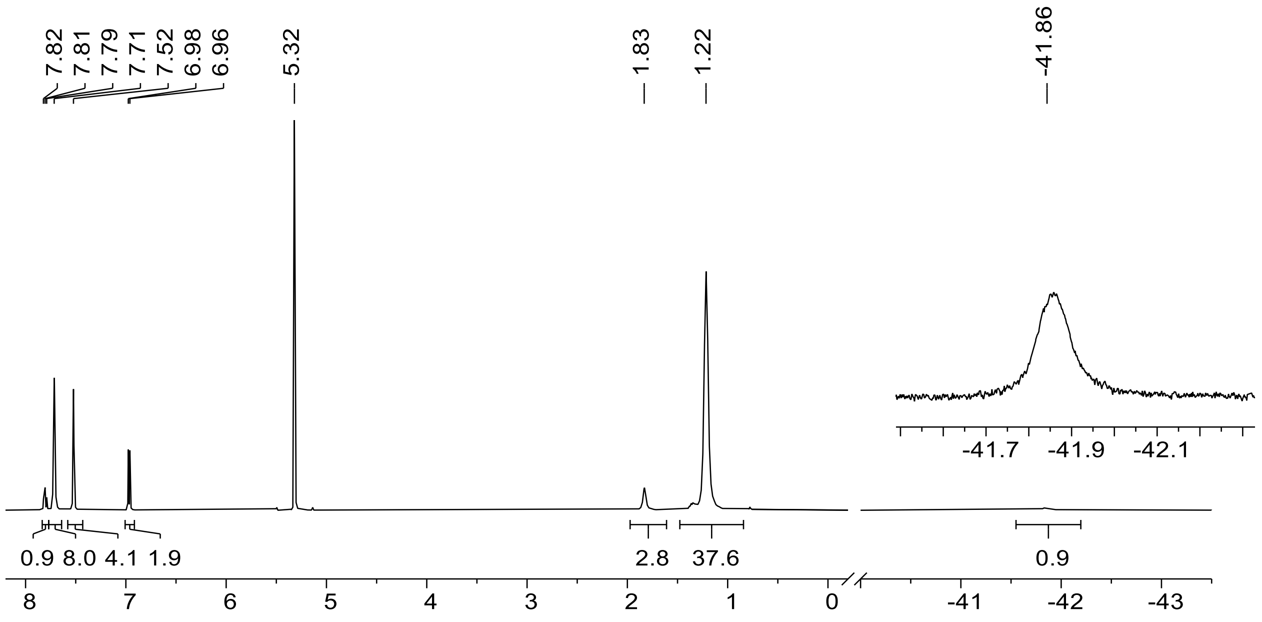

Figure S11. *^{1}H NMR spectrum of single crystals of [Ir(^{t}Bu-PONOP)MeH][BAr$^{F}_{4}$]* ***1[BAr$^{F}_{4}$]*** *stored at ambient temperature for seven days (500.22 MHz, CD_2Cl_2, 193 K).*

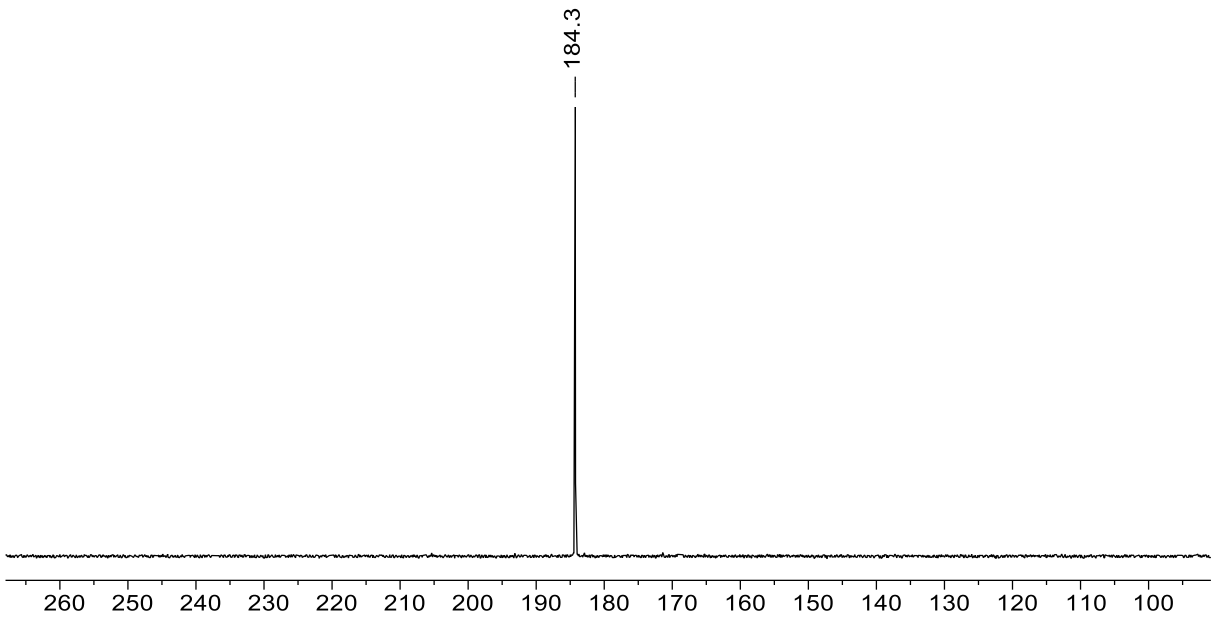

Figure S12. *$^{31}P\{^{1}H\}$ NMR spectrum of single crystals of [Ir(^{t}Bu-PONOP)MeH][BAr$^{F}_{4}$]* ***1[BAr$^{F}_{4}$]*** *stored at ambient temperature for seven days (202.50 MHz, CD_2Cl_2, 193 K).*

Stability of [Ir(^{t}Bu-PONOP)MeH][BAr$^{F}_{4}$] stored under D_2O

A suspension of single crystals of [Ir(^{t}Bu-PONOP)MeH][BAr$^{F}_{4}$] **1[BAr$^{F}_{4}$]** (15.6 mg, 10.6 μmol) in degassed D_2O (0.5 mL) under dinitrogen was stored at ambient temperature for seven days without mixing. The supernatant was decanted away under argon and the crystals dried initially under a current of argon for one hour followed by *in vacuo* for a further hour. The crystals were assayed initially by $^{31}P\{^{1}H\}$ SS NMR spectroscopy under argon (Figure S14); a minor impurity (<5%) was observed at δ 176.5. The crystals were then dissolved by condensation of CD_2Cl_2 (*ca.* 0.5 mL) on the high vacuum line and the contents assayed by NMR spectroscopy at 183 K (Figure S15 and Figure S16). Overall purity of >99% of **1[BAr$^{F}_{4}$]** was maintained as determined by solution phase ^{1}H and $^{31}P\{^{1}H\}$ NMR.

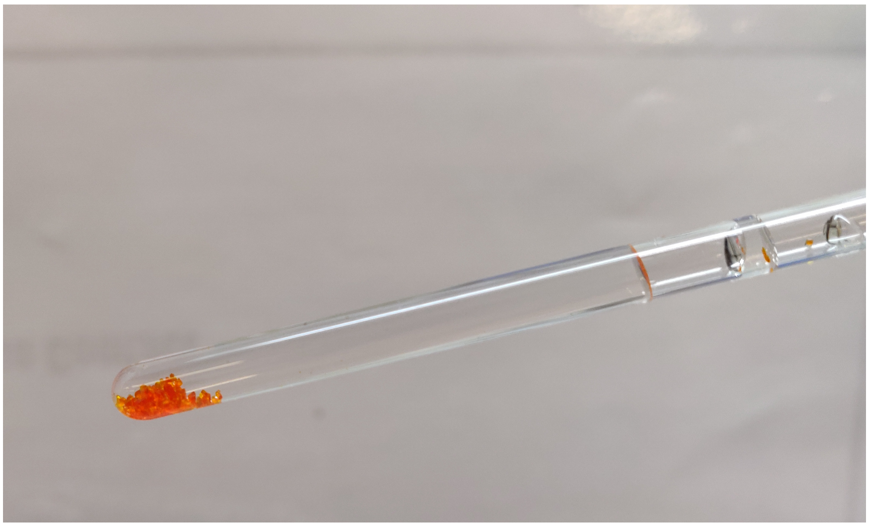

Figure S13. *Photograph of crystals of* ***1[BAr$^{F}_{4}$]*** *suspended in D_2O under dinitrogen.*

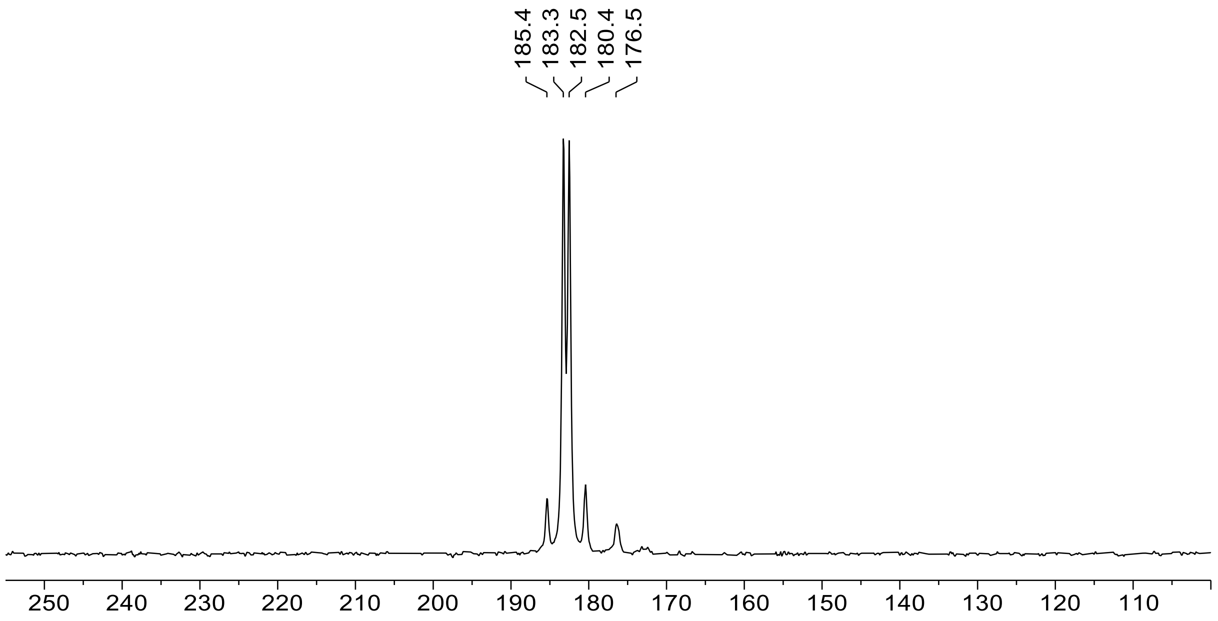

Figure S14. *$^{31}P\{^{1}H\}$ CP MAS NMR spectrum of single crystals of [Ir(^{t}Bu-PONOP)MeH][BAr$^{F}_{4}$]* ***1[BAr$^{F}_{4}$]*** *stored under D_2O for seven days (20 kHz spin rate, 162.06 MHz, 298 K).*

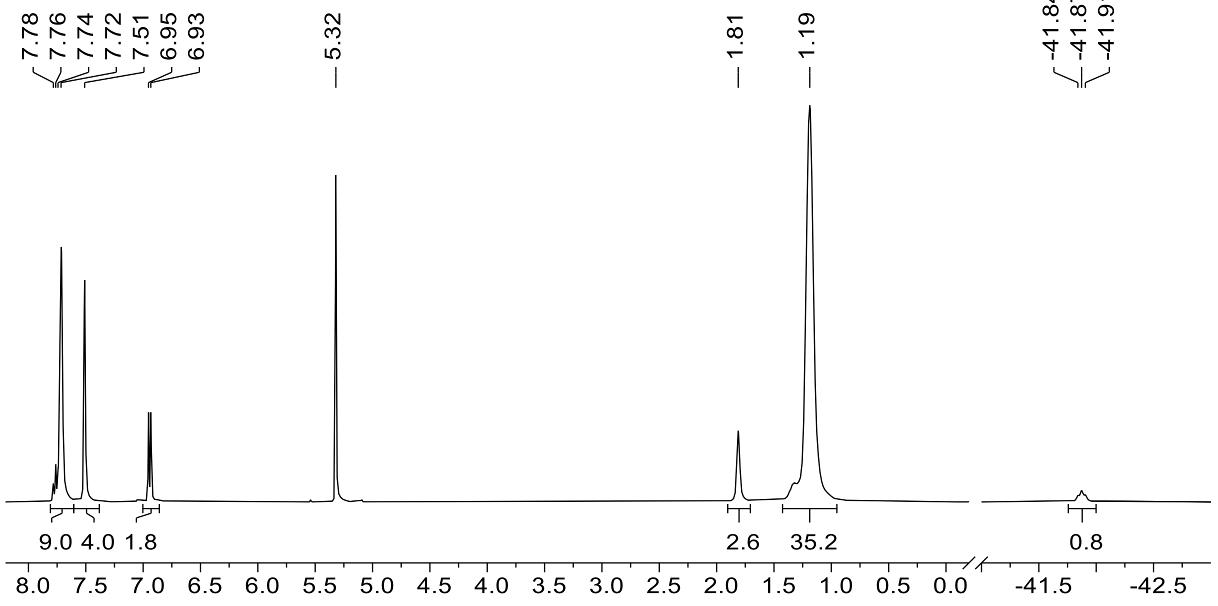

Figure S15. *^{1}H NMR spectrum of single crystals of [Ir(^{t}Bu-PONOP)MeH][BAr$^{F}_{4}$]* ***1[BAr$^{F}_{4}$]*** *stored under D_2O for seven days (400.11 MHz, CD_2Cl_2, 183 K).*

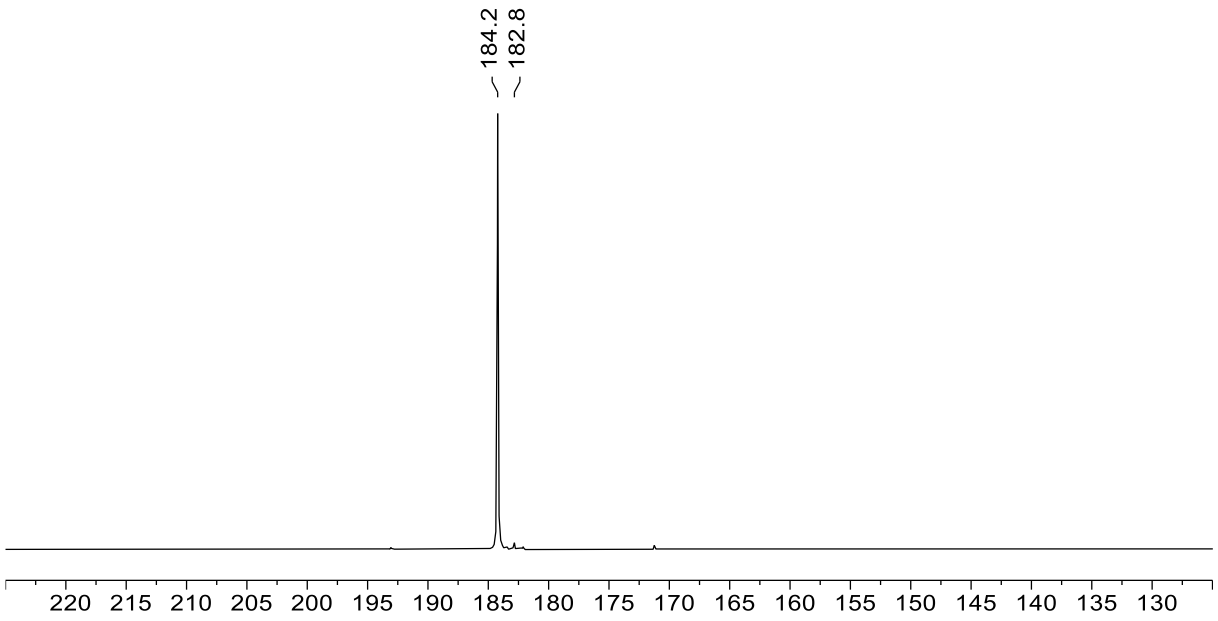

Figure S16. *^{31}P{^{1}H} NMR spectrum of single crystals of [Ir(^{t}Bu-PONOP)MeH][BAr$^F_4$]* ***1[BAr$^F_4$]*** *stored under D_2O for seven days (161.99 MHz, CD_2Cl_2, 183 K).*

Solution-State Stability of [Ir(^{t}Bu-PONOP)HMe][BAr$^F_4$]

A solution of [Ir(^{t}Bu-PONOP)MeH][BAr$^F_4$] **1[BAr$^F_4$]** (10.1 mg, 6.87 μmol) prepared by condensation of CD_2Cl_2 (*ca.* 0.5 mL) at liquid nitrogen temperature, then mixing at −78 °C to dissolve the single crystals, was monitored by ^{1}H and ^{31}P{^{1}H} NMR spectroscopy over 18 hours at 298 K. Complete decomposition of **1[BAr$^F_4$]** to a mixture of products was observed within 5 hours, with no component of this mixture consistent with data for **2[BAr$^F_4$]**. The broad signals observed (δ 19.1, 9.15) are likely paramagnetic iridium species and the identity of these compounds is currently under investigation.

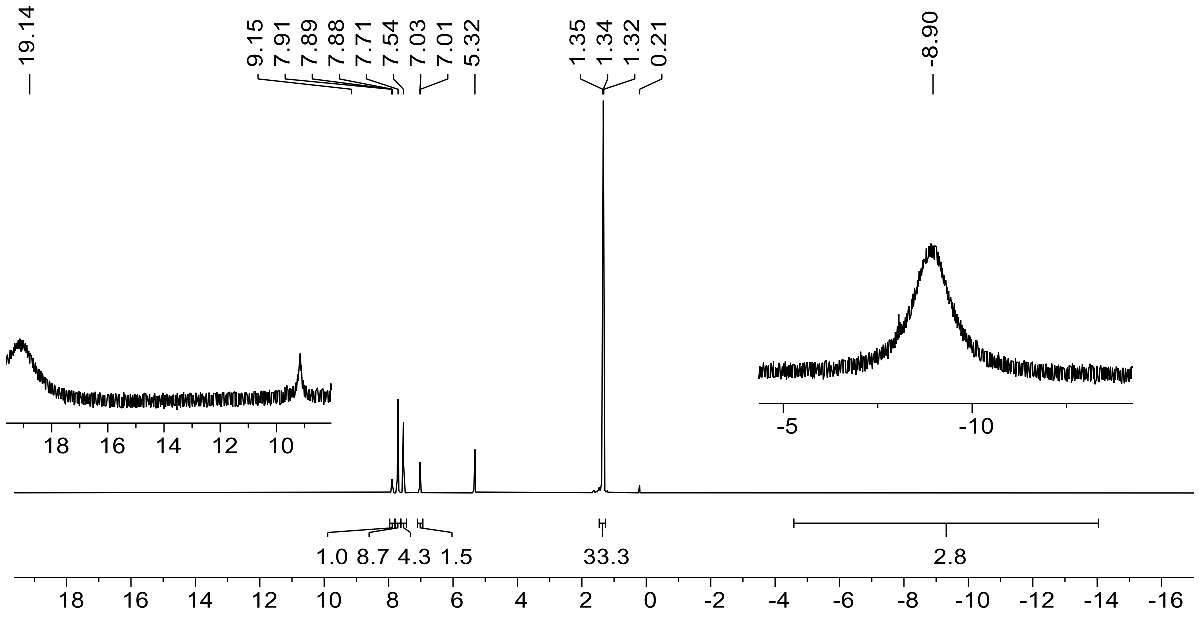

Figure S17. *^{1}H NMR spectrum of [Ir(^{t}Bu-PONOP)MeH][BAr$^F_4$]* ***1[BAr$^F_4$]*** *at ambient temperature immediately after mixing (500.12 MHz, CD_2Cl_2, 298 K).*

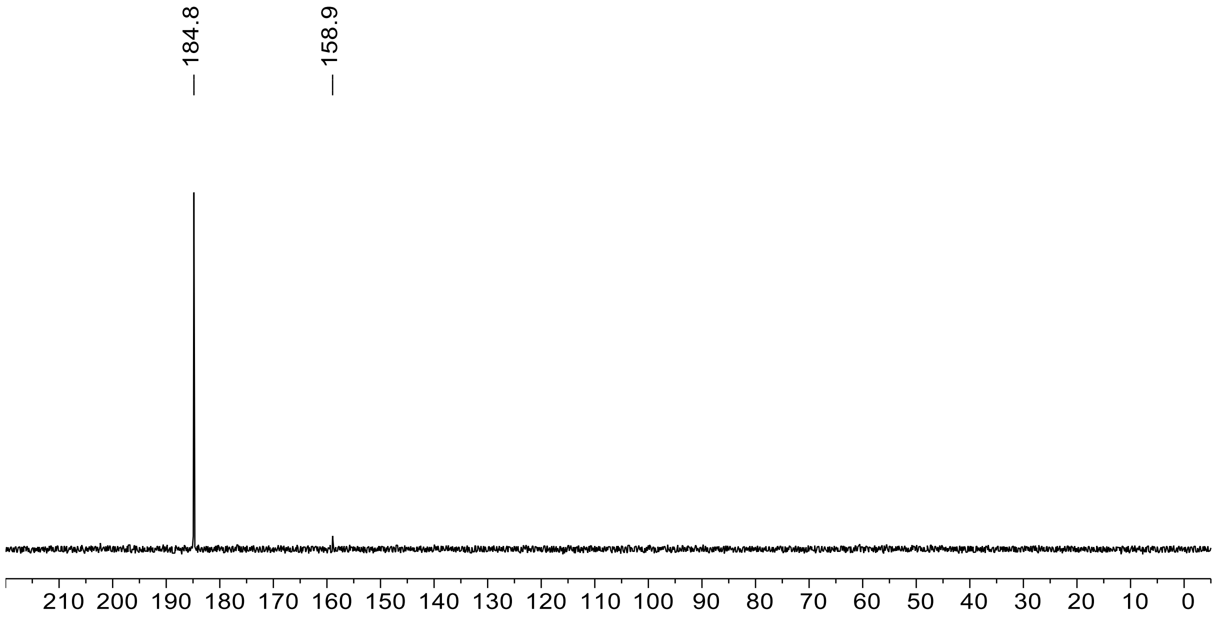

Figure S18. *$^{31}P\{^{1}H\}$ NMR spectrum of [Ir(^{t}Bu-PONOP)MeH][BAr$^{F}{}_{4}$]* ***1[BAr$^{F}{}_{4}$]*** *at ambient temperature immediately after mixing (202.47 MHz, CD_2Cl_2, 298 K).*

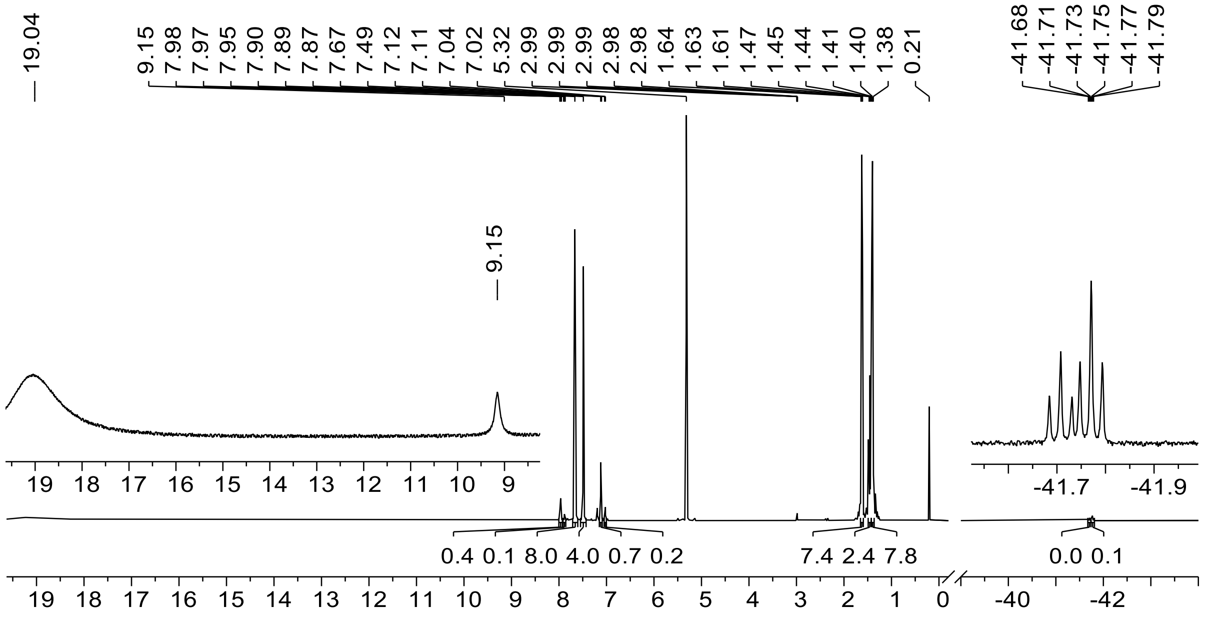

Figure S19. *^{1}H NMR spectrum of [Ir(^{t}Bu-PONOP)MeH][BAr$^{F}{}_{4}$]* ***1[BAr$^{F}{}_{4}$]*** *after storage at ambient temperature for five hours in solution (500.12 MHz, CD_2Cl_2, 298 K).*

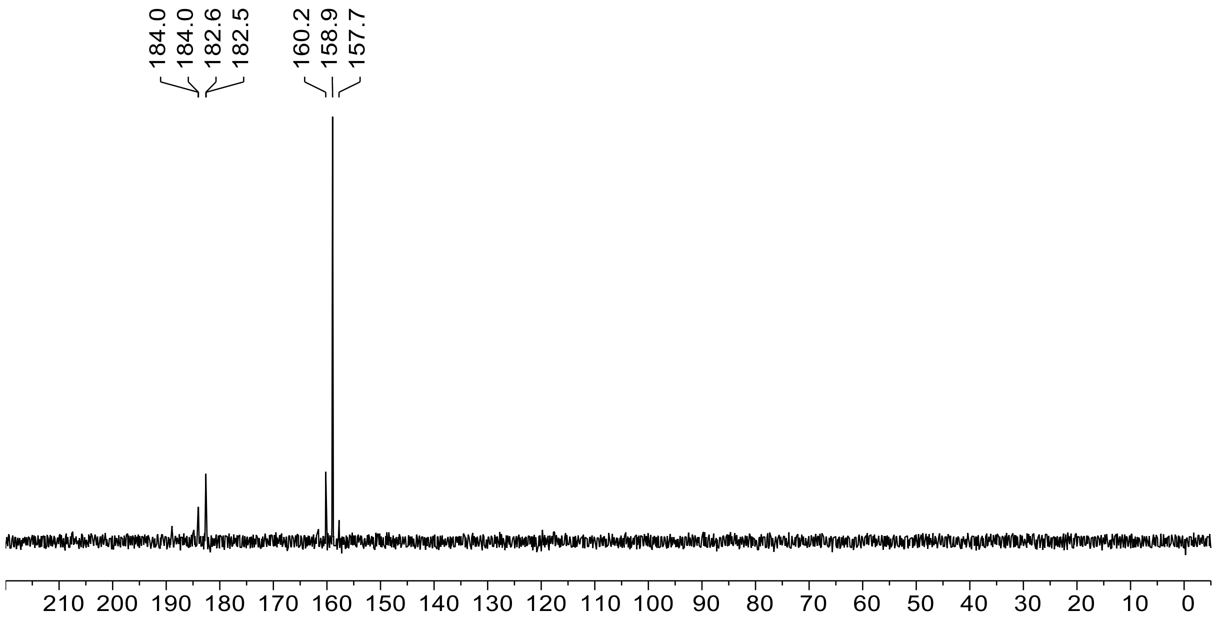

Figure S20. *$^{31}P\{^1H\}$ NMR spectrum of [Ir(^{t}Bu-PONOP)MeH][BAr$^F{}_4$]* ***1[BAr$^F{}_4$]*** *after storage at ambient temperature for five hours in solution (202.47 MHz, CD_2Cl_2, 298 K).*

Preparation of [Ir(^{t}Bu-PONOP)CD_3]

This compound has previously been observed[2] during the course of investigations into H/D exchange processes at [Ir(^{t}Bu-PONOP)Me] but only limited data was reported.

A solution of $LiCD_3$·LiI (0.47 mL, 0.24 mmol, 0.50 M in Et_2O) was added directly via syringe into a red solution of [Ir(^{t}Bu-PONOP)Cl] (90.0 mg, 0.144 mmol) in toluene (~7 mL). The solution was heated at 90 °C for 48 hours, cooled to ambient temperature and volatiles removed *in vacuo*. The product was extracted into hexane (2 x 10 mL), filtered, volatiles again removed *in vacuo* and the solids dried at ambient temperature (2 x 10^{-2} mbar) to afford [Ir(^{t}Bu-PONOP)CD_3] as a dark red powder (63.0 mg, 103 µmol, 72%). Data are consistent with the selected data ($^{31}P\{^1H\}$ NMR δ 186.7) previously reported for this compound.

Data for [Ir(^{t}Bu-PONOP)CD_3]

^{1}H NMR (400.12 MHz, C_6D_6, 298 K) δ 7.43 (tt, $^3J_{HH}$ = 7.9, $^5J_{HP}$ = 0.9, 1H, py), 6.13 (d, $^3J_{HH}$ = 7.9 , 2H, py), 2.11-2.05 (m, 0.1H, $IrCD_nH_{3-n}$), 1.42 (vt, J_{HP} = 13.7, 36H, P^tBu_2).

^{2}H NMR (61.42 MHz, C_6D_6, 298 K) δ 2.03 (s, $IrCD_nH_{3-n}$).

$^{13}C\{^1H\}$ NMR (151 MHz, C_6D_6, 298 K) δ 163.6 (vt, J = 8, py C), 130.4 (py CH), 101.6 (vt, J = 8, py CH), 40.5 (vt, J = 17, P$C(CH_3)_3$), 27.9 (vt, J = 8, P$C(CH_3)_3$), -26.1 (br m, $IrCD_nH_{3-n}$).

$^{31}P\{^1H\}$ NMR (243 MHz, C_6D_6, 298 K) δ 186.5 ($IrCD_3$), 186.4 ($IrCD_2H$), 186.3 ($IrCDH_2$).

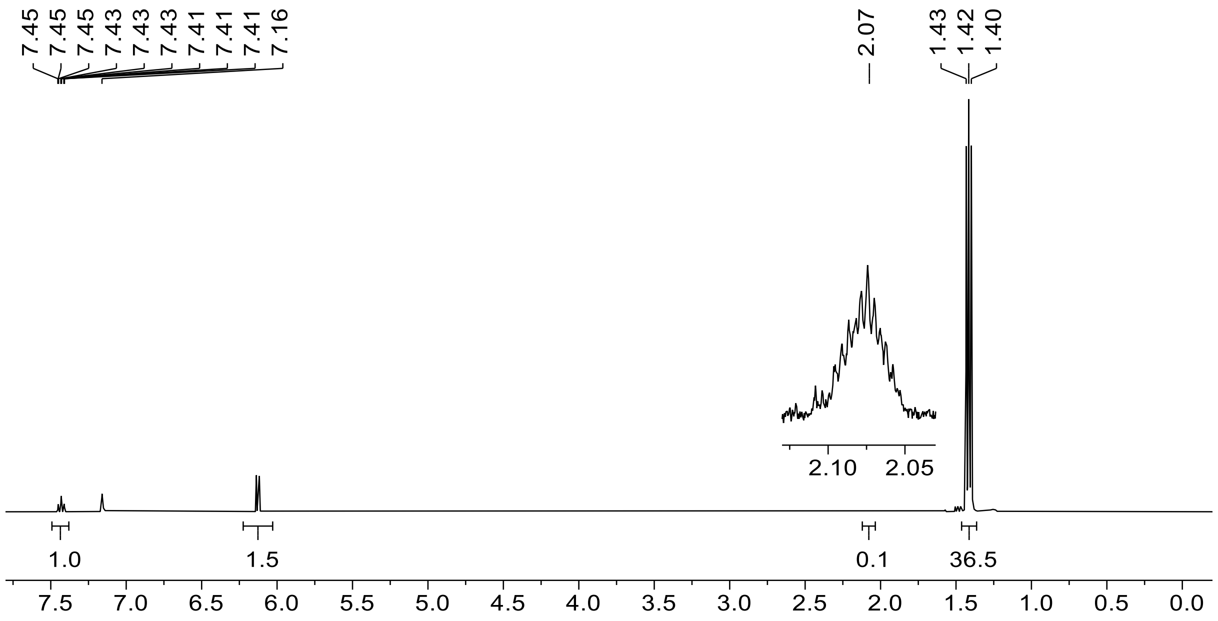

Figure S21. *^{1}H NMR Spectrum of [Ir(^{t}Bu-PONOP)CD_3] (400.12 MHz, C_6D_6, 298 K).*

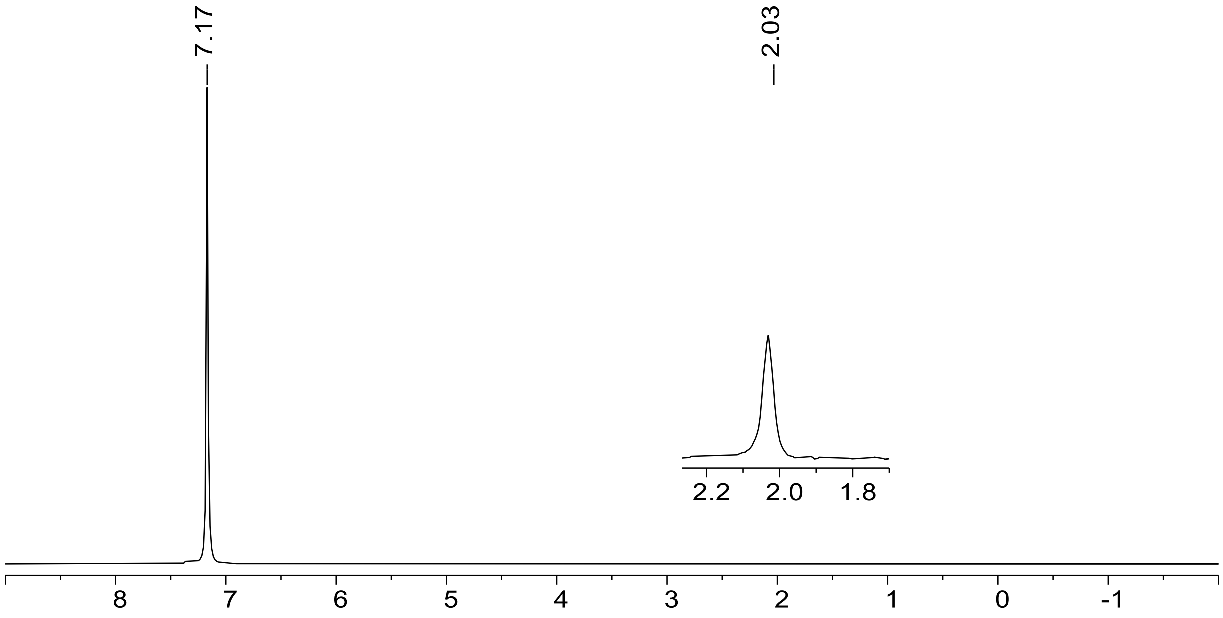

Figure S22. *^{2}H NMR spectrum of [Ir(^{t}Bu-PONOP)CD_3] (61.42 MHz, C_6D_6, 298 K).*

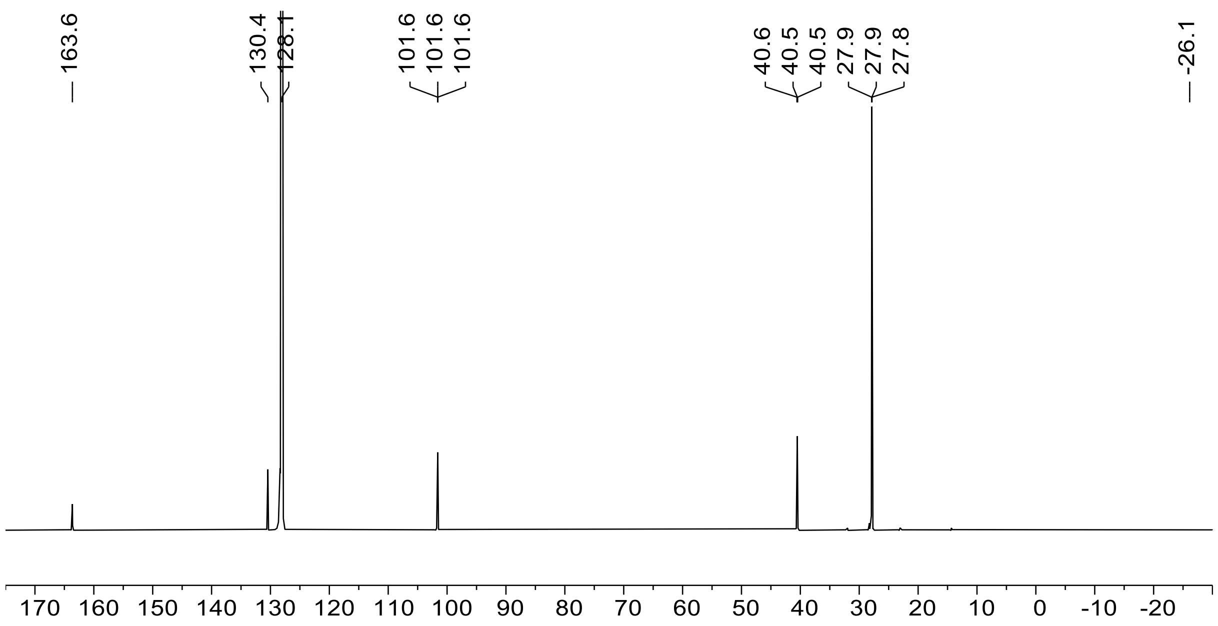

Figure S23. *$^{13}C\{^1H\}$ NMR spectrum of [Ir(^{t}Bu-PONOP)CD$_3$] (150.91 MHz, C_6D_6, 298 K).*

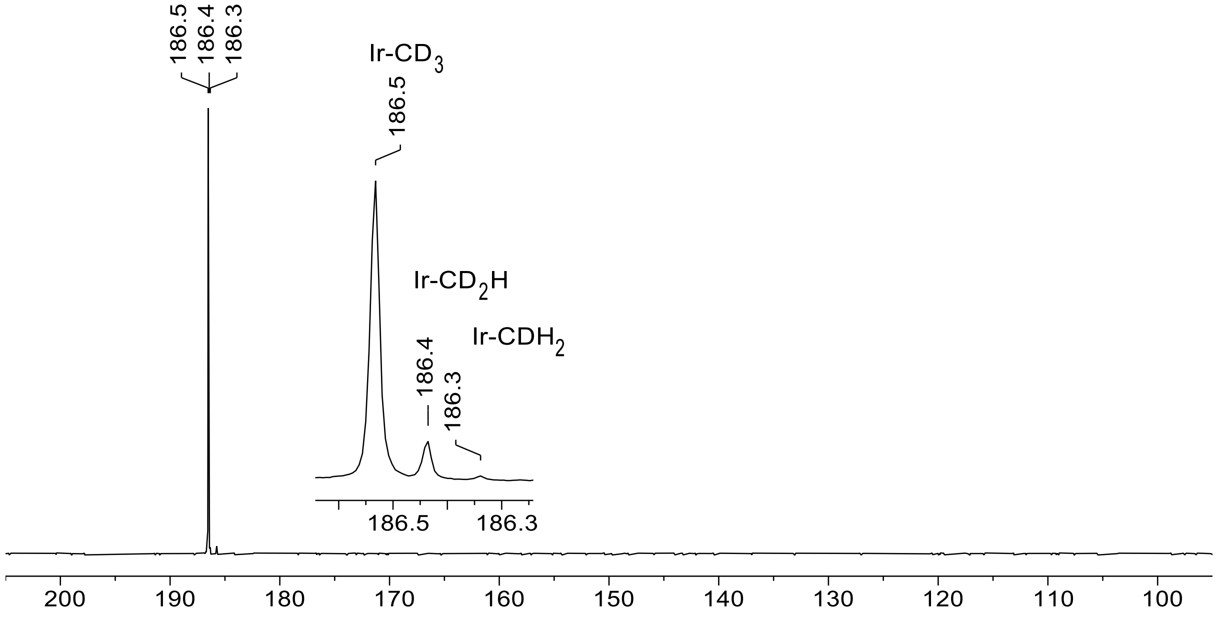

Figure S24. *$^{31}P\{^1H\}$ NMR spectrum of [Ir(^{t}Bu-PONOP)CD$_3$] (242.95 MHz, C_6D_6, 298 K). The detail inset shows the peaks assigned to $^1H/^2H$ isotopologues.*

Preparation of Solutions of Anhydrous Deuterium Chloride

Deuterium chloride gas that is currently commercially available was found to be insufficiently anhydrous for the organometallic syntheses reported herein and lead to ^1H incorporation or hydrolysis products. The following procedure provides manageable quantities of sufficiently anhydrous gaseous deuterium chloride from commercially available materials.

An 0.445 L ampoule fitted with a PTFE high-vacuum stopcock containing NaCl flame-sealed in a 5 mm OD glass tube under high vacuum (1.105 g, 18.92 mmol), D_2SO_4 (6.1835 g, 61.78 mmol) and a PTFE coated

magnetic stirrer bar was evacuated on the high-vacuum line and then sealed under static vacuum. The ampoule was then vigorously shaken to break the sealed tube containing the NaCl whereupon gas evolution was observed. The suspension was stirred for 24 hours before the contents were transferred through condensation on the high-vacuum line to an evacuated ~0.5 L gas storage bulb (0.56 g, 14.95 mmol, 79%).

A solution of DCl in diethyl ether was prepared by vacuum condensation of the contents of the gas bulb (0.56 g, 14.95 mmol) into an ampoule (~50 mL internal volume) followed by diethyl ether (~20 mL) and then cautiously thawing under static vacuum at −78 °C followed by backfilling with argon. This solution concentration was assayed as below.

Titration of Deuterium Chloride Solution

A solution of DCl in diethyl ether (0.2 mL) prepared above was added to a solution of PCy_3 (42 mg, 0.15 mmol) in $CDCl_3$ (0.4 mL) in an NMR tube fitted with a high vacuum PTFE (J. Young) valve, shaken several times to ensure full mixing and then assayed by quantitative $^{31}P\{^1H\}$ NMR spectroscopy at ambient temperature.

Conversion to $[DPCy_3][BAr^F_4]$ (δ 20.7, $^1J_{PD}$ = 75, t) was observed to be 93.6% with the balance (6.4%) unreacted PCy_3 (δ 10.6) and the concentration was calculated to be 0.81 mol L^{-1}.

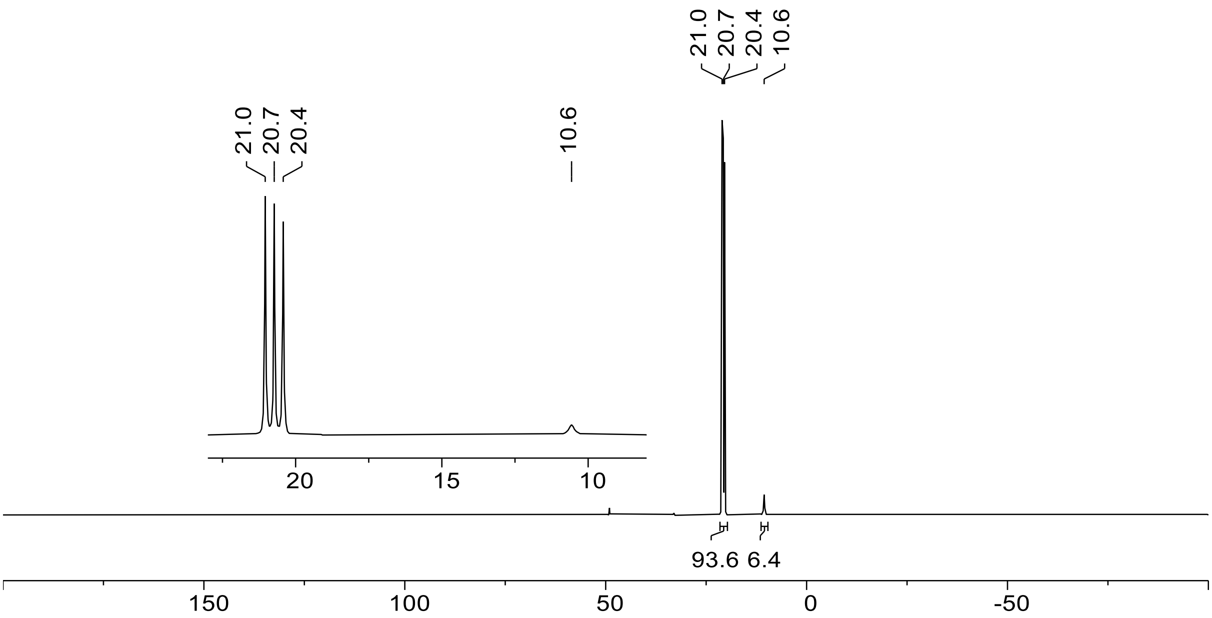

Figure S25. *$^{31}P\{^1H\}$ NMR spectrum of the mixture of $[DPCy_3][BAr^F_4]$ and PCy_3 (242.93 MHz, $CDCl_3$, 298 K).*

Preparation of $[D(OEt_2)_2][BAr^F_4]$

This is a variation of the published procedure.[9] All manipulations involving $[D(OEt_2)_2][BAr^F_4]$ must be conducted in silylated glassware to avoid exchange of ^2H for ^1H from the surface silanols present on borosilicate glass. The procedure of Kraus and Schulz has been found to be the most convenient.[10]

A solution of DCl (5.5 mL, 4.5 mmol, 0.81 M) was added to a solution of Na[BAr$^F{}_4$] (2.00 g, 2.25 mmol) in diethyl ether (50 mL) at −30 °C; the resulting suspension was stirred for 15 minutes before volatiles were removed under reduced pressure at −30 °C. The resulting white oily material was re-suspended in diethyl ether (20 mL), filtered at −30 °C, layered with pentane (20 mL) then stored at −80 °C until large colourless blocks grew (~ 1 week). The mother liquor was decanted via cannula, and the crystals washed with pentane (3 x 10 mL) at −30 °C and dried overnight *in vacuo* (2 x 10^{-2} mbar) to afford the product as a white crystalline powder (1.77 g, 1.75 mmol, 79 %).

Data for [D(OEt$_2$)$_2$][BAr$^F{}_4$]

^{1}H NMR (500 MHz, CD_2Cl_2, 193 K) δ 16.68 (s, 0.15H, Et$_2$O*H*OEt$_2$), 7.72 (s, 8H, Ar$^F{}_4$), 7.52 (s, 4H, Ar$^F{}_4$), 3.96 (q, $^3J_{HH}$ = 7.2, 8H, CH$_3$C*H*$_2$O), 1.31 (t, $^3J_{HH}$ = 7.2, 12H, C*H*$_3$CH$_2$O).

^{2}H NMR (76.79 MHz, CD_2Cl_2, 193 K) δ 16.80 (s, 1D, Et$_2$O*D*OEt$_2$).

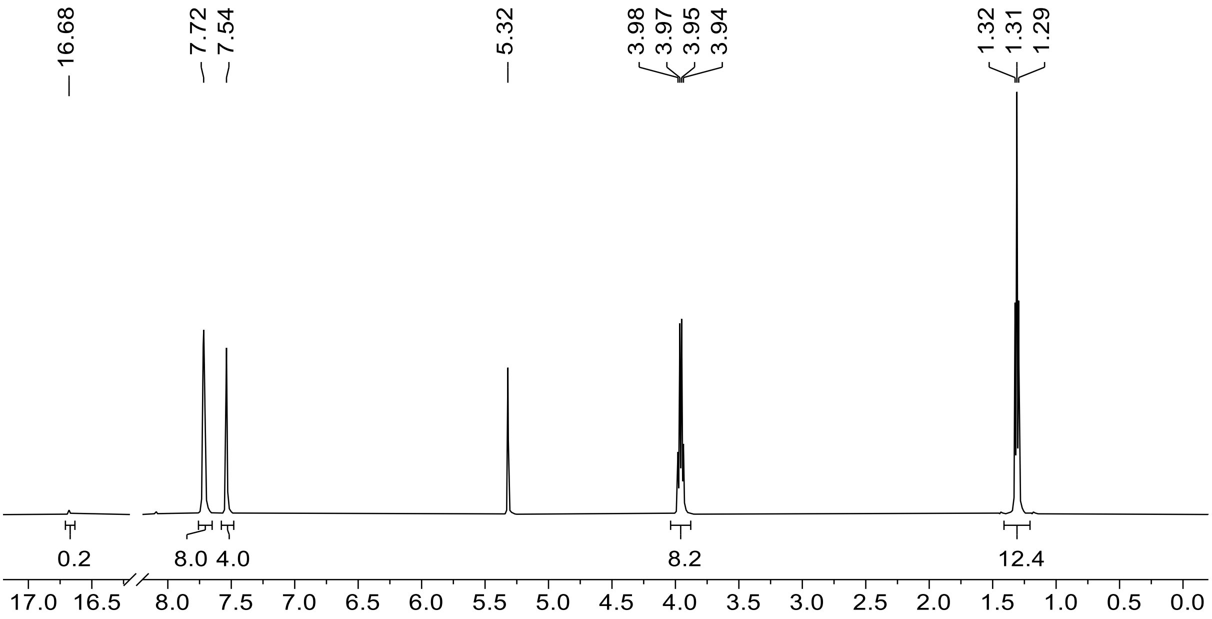

Figure S26. *^{1}H NMR spectrum of [D(OEt$_2$)$_2$][BAr$^F{}_4$] (500.24 MHz, CD_2Cl_2, 193 K).*

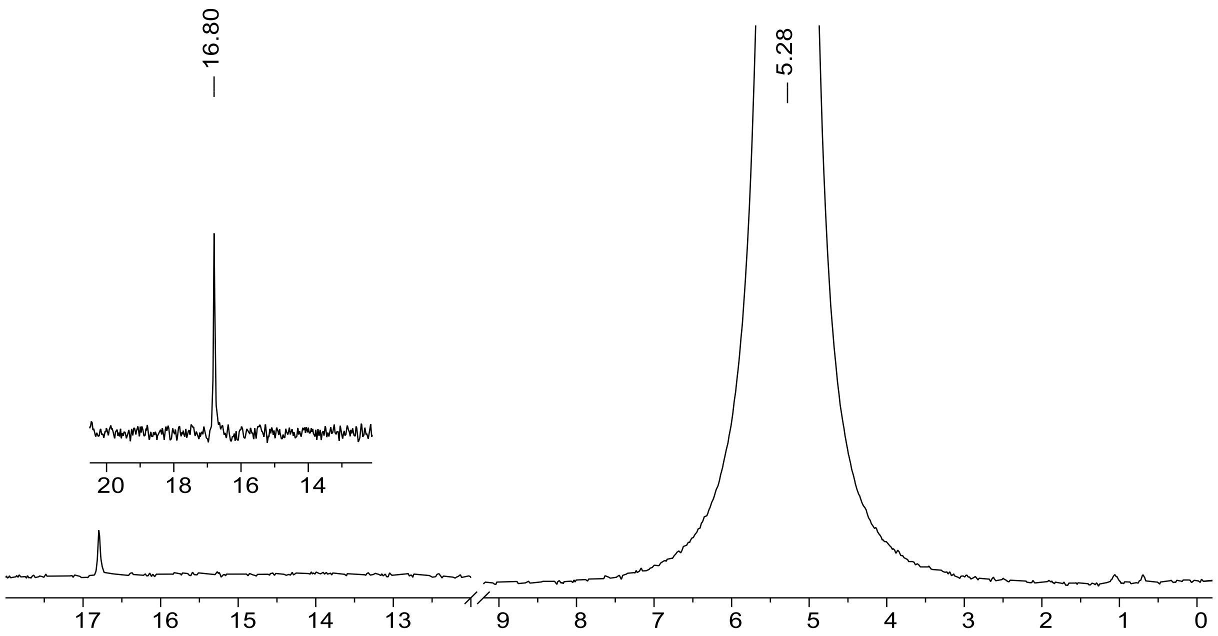

Figure S27. *^{2}H NMR spectrum of $[D(OEt_2)_2][BAr^F{}_4]$ (76.79 MHz, CD_2Cl_2, 193 K).*

Preparation of [Ir(tBu-PONOP)(CD_3)D][$BAr^F{}_4$]

All manipulations involving $[D(OEt_2)_2][BAr^F{}_4]$ must be conducted in silylated glassware to avoid exchange of ^{2}H for ^{1}H from the surface silanols present on borosilicate glass (*vide supra*)

A freshly prepared solution of $[D(OEt_2)_2][BAr^F{}_4]$ (101.5 mg , 100.2 µmol) in 1,2-$C_6H_4F_2$ (*ca.* 5 mL) at −40 °C was added to a solution of [Ir(tBu-PONOP)CD_3] (62.0 mg, 102 µmol) also in 1,2-$C_6H_4F_2$ (*ca.* 5 mL) at −40 °C dropwise over a period of 5 minutes. The resulting bright orange solution was stirred for 15 minutes at −40 °C before volatiles were removed *in vacuo* at this temperature. The remaining materials were re-dissolved in 1,2-$C_6H_4F_2$ (*ca.* 10 mL) at −40 °C, layered with excess heptane (*ca.* 100 mL) and stored at −40 °C to afford [Ir(tBu-PONOP)(CD_3)D][$BAr^F{}_4$] **d_4-1[$BAr^F{}_4$]** in approximately 90% chemical purity and 90-95% ^{2}H content (109.9 mg, 74.70 mmol, 75%); the mass balance consists of [Ir(cyclo-tBu-PONOP')H][$BAr^F{}_4$] **2[$BAr^F{}_4$]** (*ca.* 2%) and the remainder three as yet unidentified iridium hydride species (*ca.* 1%, 5% and 2%).

Data for [Ir(tBu-PONOP)(CD_3)D][$BAr^F{}_4$]

^{1}H NMR (400.11 MHz, CD_2Cl_2, 178 K) δ 7.79 (t, $^3J_{HH}$ = 8, 1H, py), 7.72 (s, 8H, $Ar^F{}_4$), 7.52 (s, 4H, $Ar^F{}_4$), 6.96 (d, $^3J_{HH}$ = 8, 2H, py), 1.76 (s br, 0.1H, IrCH_3), 1.19 (s br, 36H, P^{t}Bu), 41.88 (t, $^3J_{HH}$ = 13.6, 0.1H, IrH).

^{2}H NMR (76.79 MHz, CH_2Cl_2, 178 K) 1.76 (IrCD_3), the resonance for IrD was not observed.

^{31}P{^{1}H} NMR (162.00 MHz, CD_2Cl_2, 178 K) δ 184.7 (major isotopologue), 184.6, 184.5, 184.4 (minor isotopologues).

^{13}C{^{1}H} CP MAS NMR (20 kHz spin rate, 100.66 MHz, 298 K) δ 165.7-162.2 (Ar^F and py *C*), 142.4 (py *C*H), 136.9-116.4 (Ar^F), 104.4-102.7 (py *C*H), 43.0-40.5 (P*C*(CH_3)$_3$), 26.9-25.6 (PC(*C*H_3)$_3$), −23.6 (Ir*C*D_3).

^{31}P{^{1}H} CP MAS NMR (20 kHz spin rate, 162.06 MHz, 298 K) δ 184.7 (d, J_{PP} = 342, P^{t}Bu$_2$), 181.3 (d, J_{PP} = 342, P^{t}Bu$_2$). Resonances for **2[$BAr^F{}_4$]** are also observed (179.0-176.8, P^{t}Bu) and (142.8-138.4, P^{t}Bu)

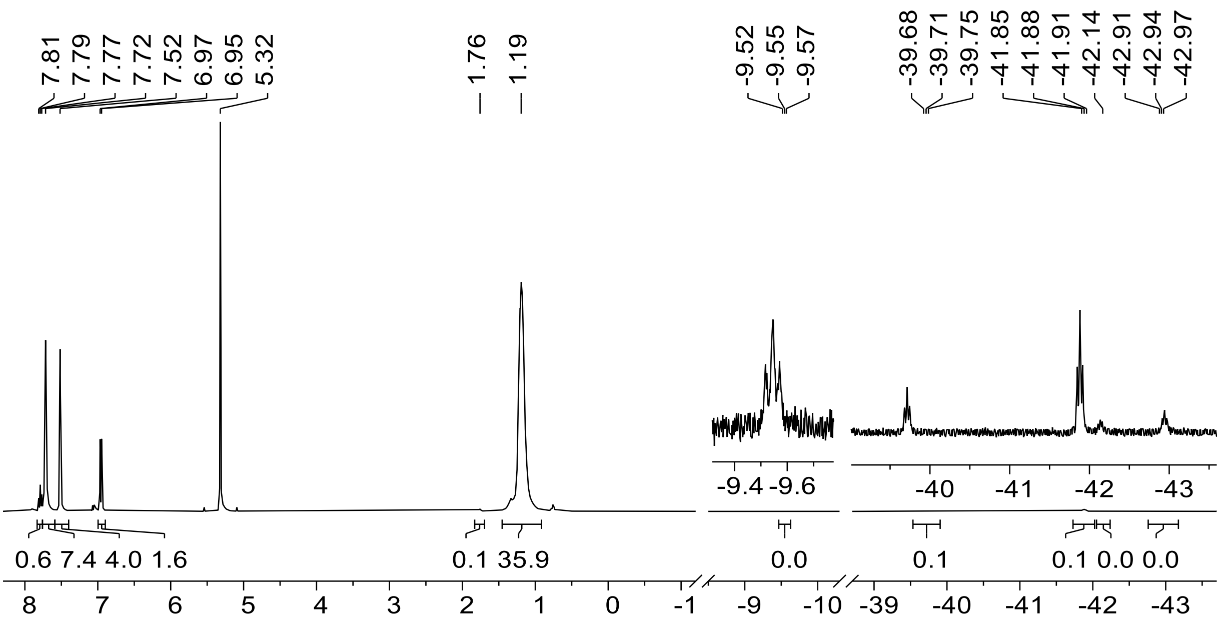

Figure S28. *^{1}H NMR spectrum of [Ir(tBu-PONOP)(CD3)D][BAr$^F_4$]* ***d4-1[BAr$^F_4$]*** *(400.11 MHz, CD_2Cl_2, 178 K).*

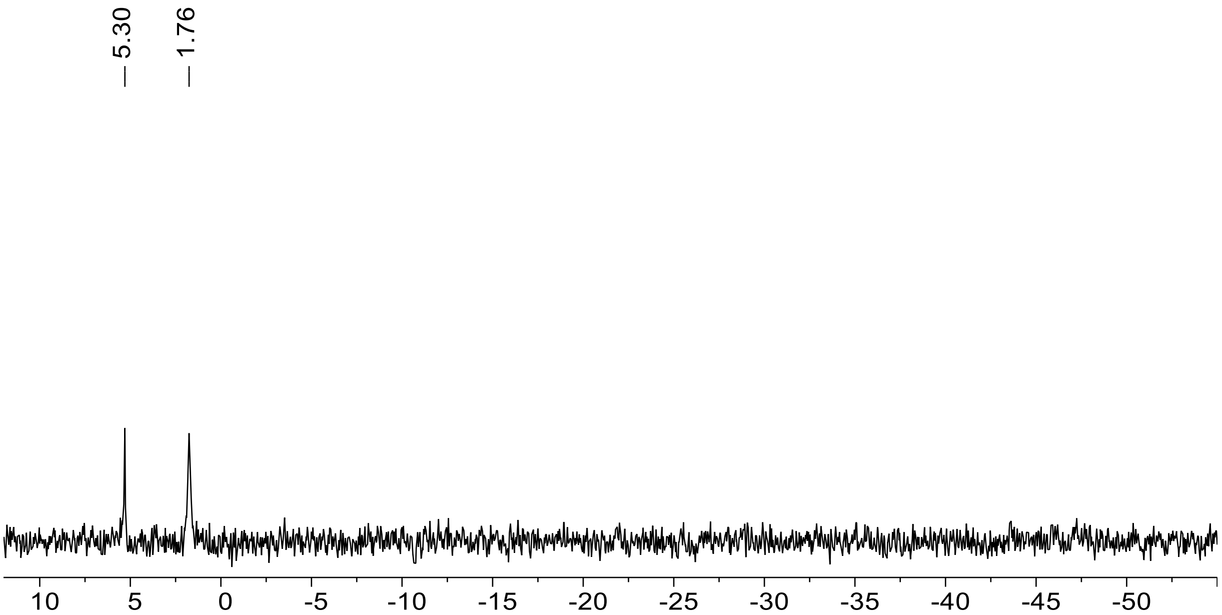

Figure S29. *^{2}H NMR spectrum of [Ir(tBu-PONOP)(CD3)D][BAr$^F_4$]* ***d4-1[BAr$^F_4$]*** *(76.79 MHz, CH_2Cl_2, 178 K).*

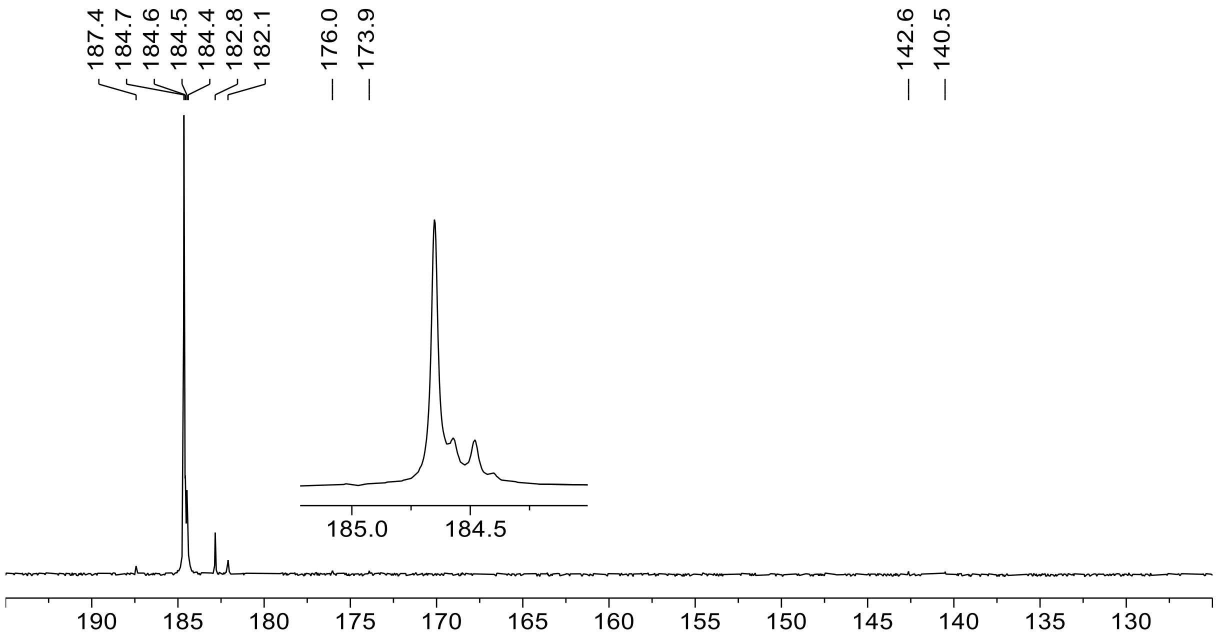

Figure S30. *$^{31}P\{^1H\}$ NMR spectrum of [Ir(^{t}Bu-PONOP)(CD_3)D][BAr$^F{}_4$]* ***d_4-1[BAr$^F{}_4$]*** *(162.00 MHz, CD_2Cl_2, 178 K).*

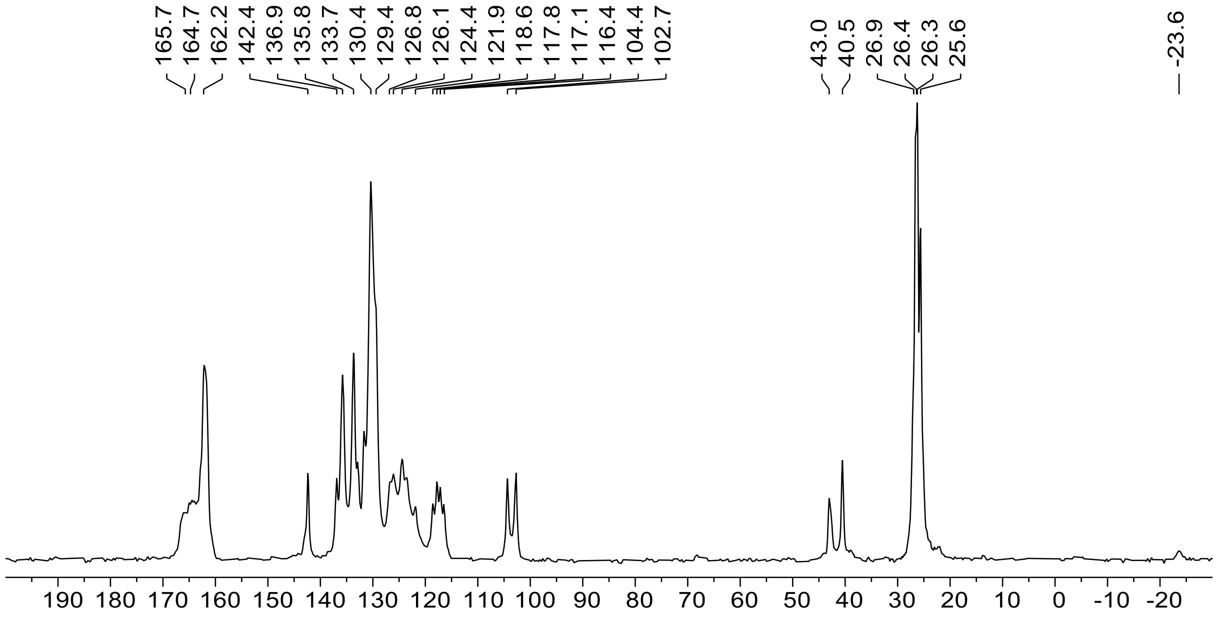

Figure S31. *$^{13}C\{^1H\}$ CP MAS NMR spectrum of [Ir(^{t}Bu-PONOP)(CD_3)D][BAr$^F{}_4$]* ***d_4-1[BAr$^F{}_4$]*** *after sieving to remove material <0.25 mm (20 kHz spin rate, 100.66 MHz, 298 K).*

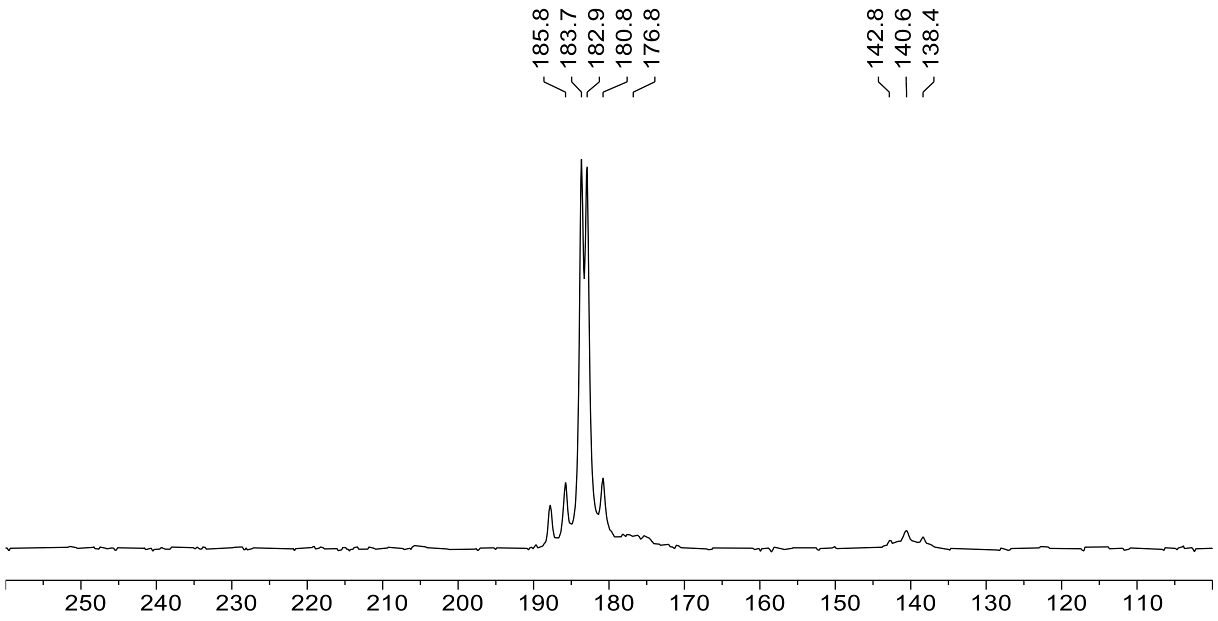

Figure S32. *$^{31}P\{^{1}H\}$ CP MAS NMR spectrum of [Ir(^{t}Bu-PONOP)(CD_3)D][BAr$^{F}_{4}$]* ***d_4-1[BAr$^{F}_{4}$]****, after recrystallisation and sieving to remove material <0.25 mm (20 kHz spin rate, 162.06 MHz, 298 K).*

Preparation of [Ir(cyclo-^{t}Bu-PONOP')H][BAr$^{F}_{4}$]

Solution phase $^{13}C\{^{1}H\}$ spectra were recorded at 253 K due to sample degradation observed over long acquisition in CD_2Cl_2.

Sieved single crystals (0.25-0.5 mm) of [Ir(^{t}Bu-PONOP)MeH][BAr$^{F}_{4}$] **1[BAr$^{F}_{4}$]** (123.6 mg, 84.03 μmol) in an ampoule fitted with a PTFE high-vacuum valve were evacuated (<5 x 10^{-6} mbar) on a greaseless high-vacuum line for 24 hours at ambient temperature. The ampoule was then heated at 80 °C for three days under dynamic high vacuum. An initial pressure surge of non-condensable gas was observed on heating (>1 x 10^{-5} mbar) with the vacuum level (<5 x 10^{-6} mbar) restored over the course of the reaction. The flask was cooled to ambient temperature under vacuum; the ampoule and contents transferred to an argon glovebox, affording [Ir(cyclo-^{t}Bu-PONOP')H][BAr$^{F}_{4}$] **2[BAr$^{F}_{4}$]** as pale orange blocks (120.5 mg, 82.82 μmol, 99%).

Data for [Ir(cyclo-^{t}Bu-PONOP')H][BAr$^{F}_{4}$]

^{1}H NMR (500.22 MHz, CD_2Cl_2, 298 K) δ 8.00 (tt, $^{3}J_{HH}$ = 8.1, $^{5}J_{HP}$ = 0.9, 1H, py), 7.72 (br. m., 8H, Ar$^{F}_{4}$), 7.56 (s, 4H, Ar$^{F}_{4}$), 7.16 (d, $^{3}J_{HH}$ = 8.2, 1H, py), 7.15 (d, $^{3}J_{HH}$ = 8.2, 1H, py), 3.09 (dddd, J_{HP} = 14.5, J_{HH} = 6.5, J_{HP} = 3.3, J_{HH} = 2.0, 1H, PC(CH_3)$_2$(CH_2)), 1.55 (d, $^{3}J_{HP}$ = 18.9, 3H, PC(CH_3)$_2$(CH_2)), 1.45 (d, $^{3}J_{HP}$ = 17.4, 9H, ^{t}Bu), 1.30 (d, $^{3}J_{HP}$ = 16.6, 9H, ^{t}Bu), 1.01 (d, $^{3}J_{HP}$ = 13.8, 3H, PC(CH_3)$_2$(CH_2)), 0.89 (d, $^{3}J_{HP}$ = 15.2, 9H, ^{t}Bu), 0.79 (br. d, $^{3}J_{HP}$ = 6.6, 1H, PC(CH_3)$_2$(CH_2)), −9.24 (app. t, $^{2}J_{HP}$ = 11.3, 1H).

$^{13}C\{^{1}H\}$ NMR (125.80 MHz, CD_2Cl_2, 253 K) δ 162.3 (obscured dd, $^{2}J_{CP}$ = 6, $^{4}J_{CP}$ = 2, py C), 161.8 (q, $^{1}J_{CB}$ = 50 , Ar^{F}), 160.9 (dd, $^{2}J_{CP}$ = 6, $^{4}J_{CP}$ = 3, py C), 144.7 (s, py CH), 134.8 (s br., Ar^{F}), 128.8 (qq, $^{2}J_{CF}$ = 31 , $^{4}J_{CF}$ = 5), 124.6 (q, $^{1}J_{CF}$ = 272, CF_3), 117.6 (sept, $^{4}J_{CF}$ = 5, Ar^{F}), 104.7 (d, $^{3}J_{CP}$ = 5, py CH), 104.2 (d, $^{3}J_{CP}$ = 5, py CH), 68.0 (dd, $^{1}J_{CP}$ = 18, $^{2}J_{CP}$ = 4, P*C*(CH_2)(CH_3)$_2$), 51.3 (dd, $^{1}J_{CP}$ = 17, $^{2}J_{CP}$ = 3, P*C*(CH_3)$_3$), 39.2 (dd, $^{1}J_{CP}$ = 9, $^{2}J_{CP}$ = 6, P*C*(CH_3)$_3$), 38.8 (dd, $^{1}J_{CP}$ = 13, $^{2}J_{CP}$ = 6, P*C*(CH_3)$_3$), 25.9 (d, $^{2}J_{CP}$ = 6, PC(*C*H_3)$_3$), 25.8 (d, $^{2}J_{CP}$ = 5, PC(*C*H_3)$_3$), 25.0 (d, $^{2}J_{CP}$ = 5,

PC(CH_3)$_3$), 24.0 (d, $^2J_{CP}$ = 7, PC(CH_2)(CH_3)$_2$)),), 23.2 (d, $^2J_{CP}$ = 5, PC(CH_2)(CH_3)$_2$)), −2.5 (dd, $^2J_{CP}$ = 26, $^2J_{CP}$ = 2, PC(CH_2)(CH_3)$_2$).

^{31}P NMR (202.50 MHz, CD_2Cl_2, 298 K) δ 177.7 (d, $^2J_{PP}$ = 345, *P*^{t}Bu$_2$), 141.8 (d, $^2J_{PP}$ = 345, *P*C(CH_2)(CH_3)$_2$).

^{13}C{^{1}H} CP MAS NMR (20 kHz spin rate, 162.04 MHz, 298 K) δ 166.4-160.5 (Ar^F and py *C*), 142.8 (py *C*H), 135.9-115.7 (Ar^F), 104.7-103.1 (py *C*H), 68.3-67.0 (^{t}Bu *C*), 51.7-50.9 (^{t}Bu *C*), 39.3-38.7 (^{t}Bu *C*H), 27.4-21.1 (^{t}Bu *C*H), −3.7 (PC(CH_2)(CH_3)$_2$).

^{31}P{^{1}H} CP MAS NMR (20 kHz spin rate, 162.04 MHz, 298 K) δ. 181.3-175.0, 143.1-138.3.

IR (ATR, diamond) 2108 cm^{-1} (Ir-H)

Elemental analysis found (calculated) for $C_{53}H_{51}BF_{24}IrNO_2P_2$: C, 43.62 (43.75); H, 3.53 (3.53); N, 0.95 (0.96).
ESI-MS (CH_2Cl_2) *m/z* found (calculated) for $C_{21}H_{39}IrNO_2P_2$ [M]$^+$: 592.2143 (592.2080).

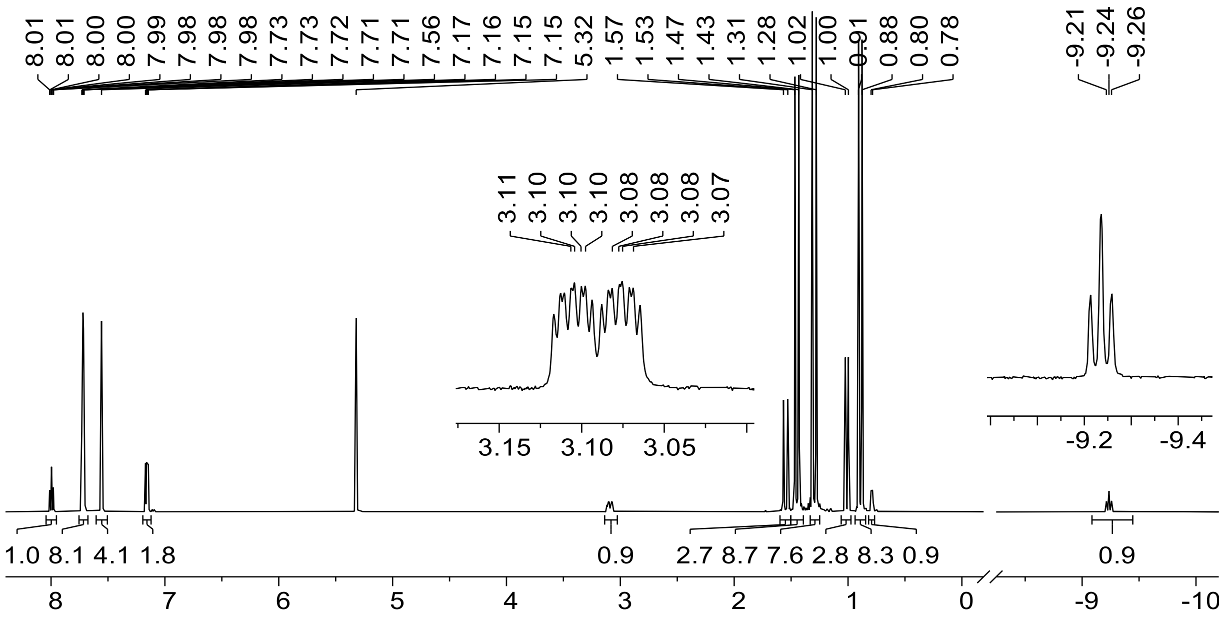

Figure S33. *^{1}H NMR Spectrum of [Ir(cyclo-^{t}Bu-PONOP')H][BAr$^F{}_4$]* ***2[BAr$^F{}_4$]*** *(500.22 MHz, CD_2Cl_2, 298 K).*

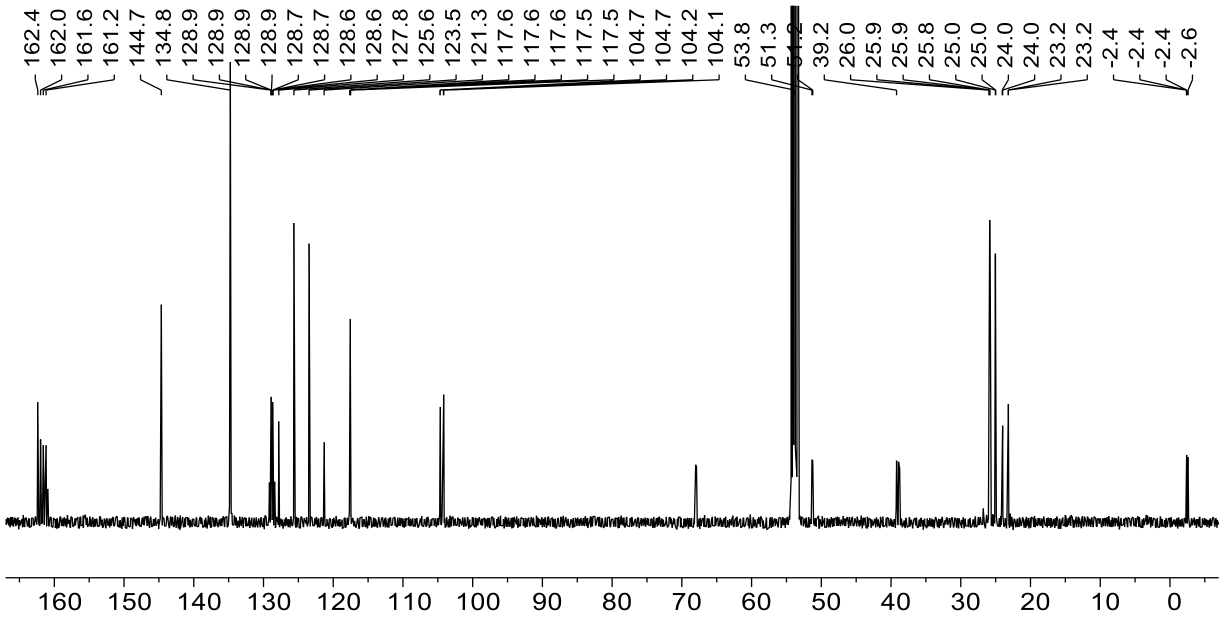

Figure S34. *$^{13}C\{^1H\}$ NMR Spectrum of [Ir(cyclo-^{t}Bu-PONOP')H][BAr$^F{}_4$]* ***2[BAr$^F{}_4$]*** *(125.80 MHz, CD_2Cl_2, 253 K).*

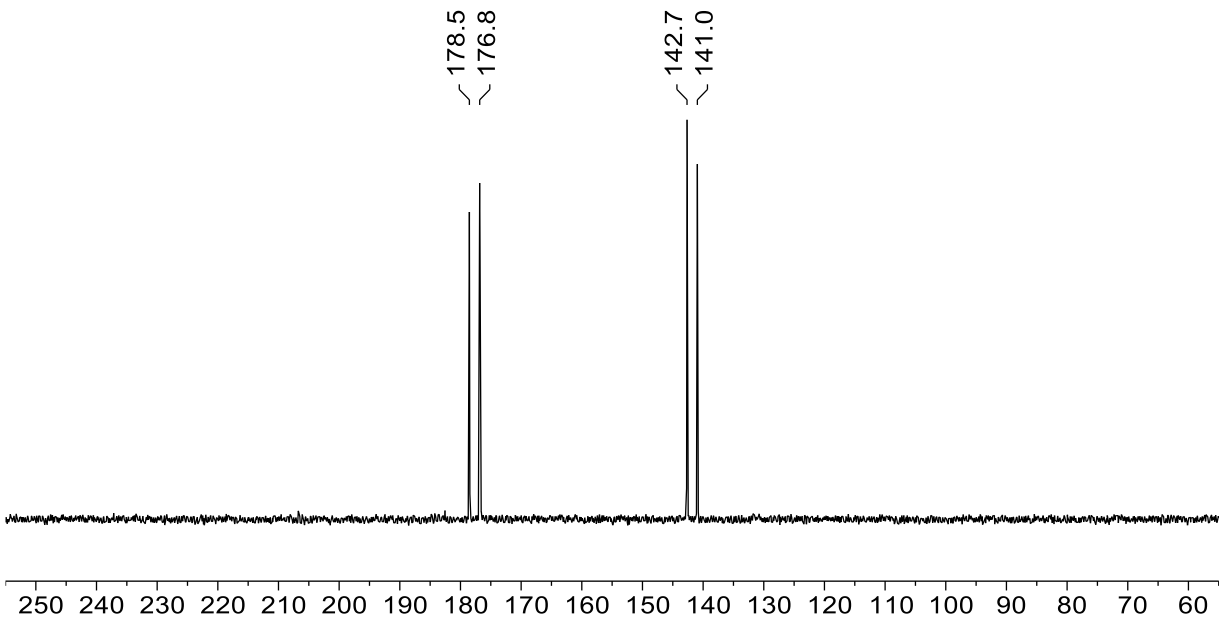

Figure S35. *$^{31}P\{^1H\}$ NMR Spectrum of [Ir(cyclo-^{t}Bu-PONOP')H][BAr$^F{}_4$]* ***2[BAr$^F{}_4$]*** *(202.53 MHz, CD_2Cl_2, 298 K).*

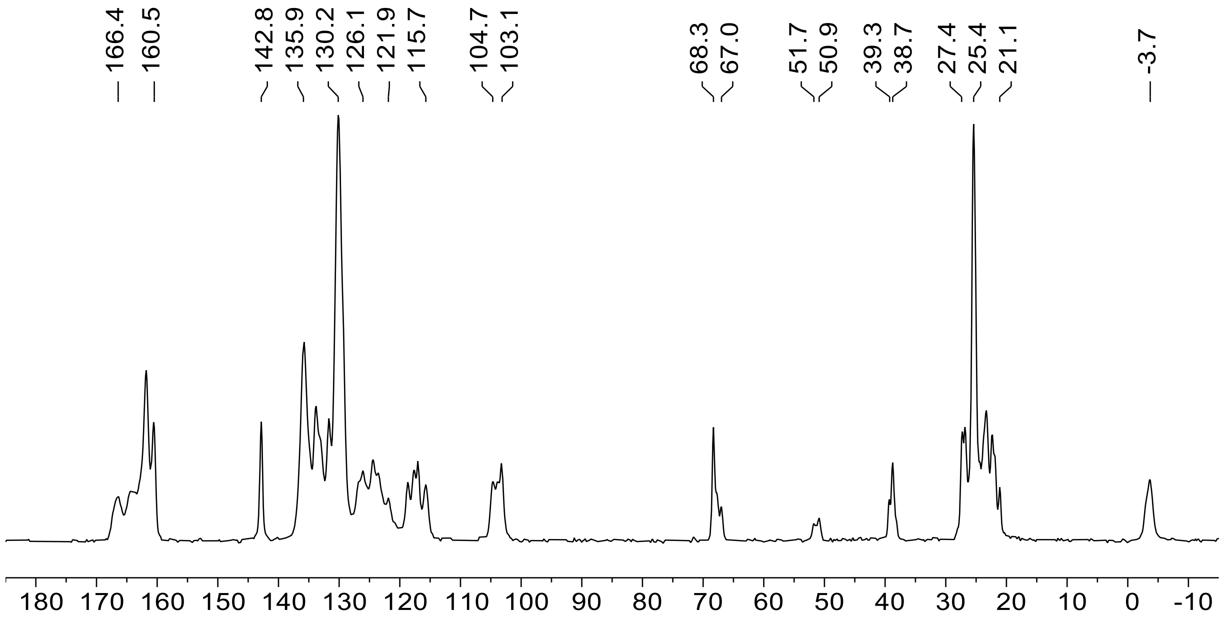

Figure S36. *$^{13}C\{^1H\}$ CP MAS NMR spectrum of [Ir(cyclo-^{t}Bu-PONOP')H][BAr$^F{}_4$]* ***2[BAr$^F{}_4$]***
(20 kHz spin rate, 100.66 MHz, 298 K).

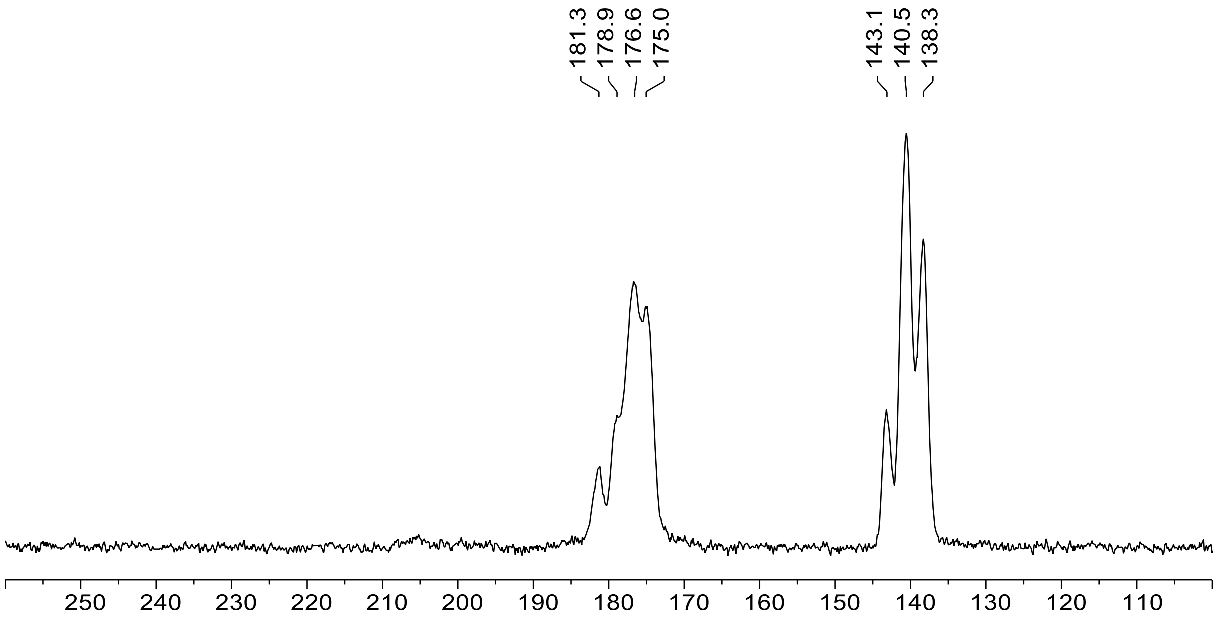

Figure S37. *$^{31}P\{^1H\}$ CP MAS NMR spectrum of [Ir(cyclo-^{t}Bu-PONOP')H][BAr$^F{}_4$]* ***2[BAr$^F{}_4$]***
(20 kHz spin rate, 162.06 MHz, 298 K).

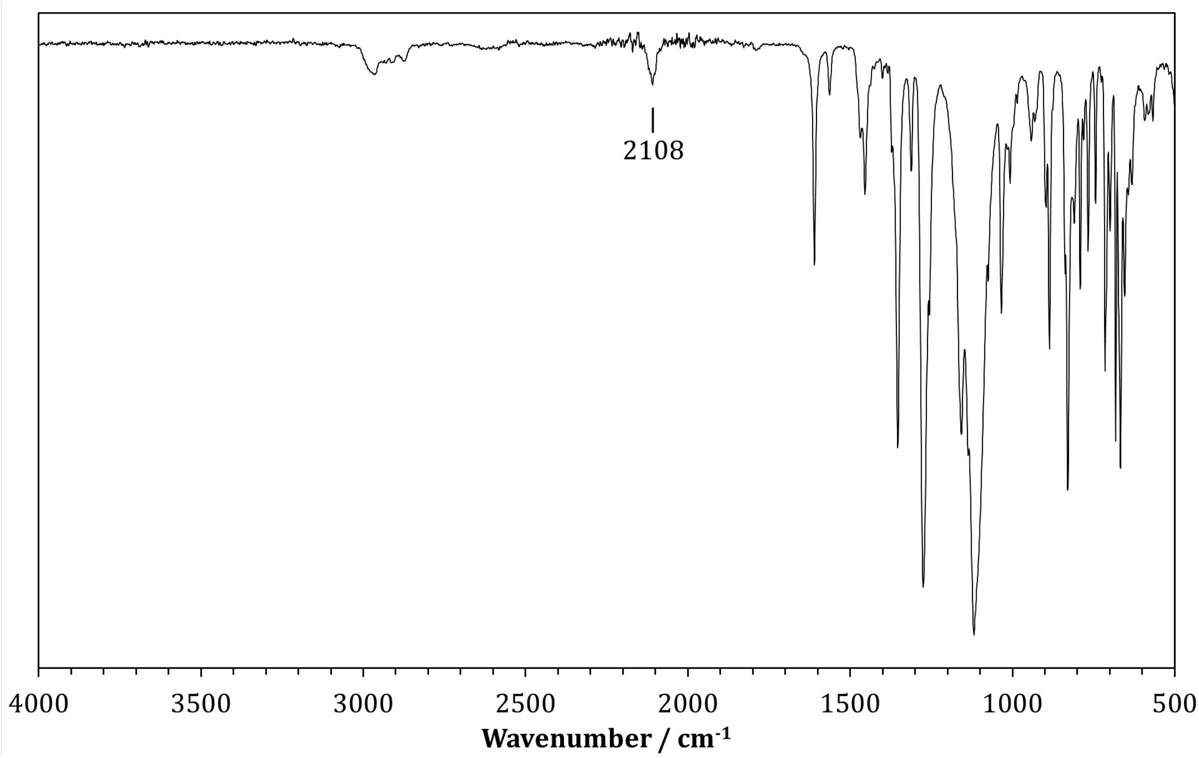

Figure S38. *FT-IR spectrum of [Ir(cyclo-^{t}Bu-PONOP')H][BAr$^F{}_4$]* ***2[BAr$^F{}_4$]****, collected by ATR at ambient temperature.*

Characterisation of [Ir(cyclo-^{t}Bu-PONOP')H(MeCN)][BAr$^F{}_4$]

CD_3CN (*ca*. 0.5 mL) was condensed on the high vacuum line onto crystals of [IrH(cycloPONOP^{t}Bu)][BAr$^F{}_4$] **2[BAr$^F{}_4$]** (6.7 mg, 4.6 μmol) and the resulting solution monitored at room temperature by ^{1}H and ^{31}P{^{1}H} NMR spectroscopy over a 48 hours with no change observed. Volatiles were removed *in vacuo* and the resulting solids redissolved in diethyl ether (*ca*. 5 mL), layered with excess pentane (*ca*. 25 mL) and then stored at −25 °C. The resulting colourless crystals of [Ir(cyclo-^{t}Bu-PONOP')H(MeCN)][BAr$^F{}_4$] **2-MeCN[BAr$^F{}_4$]** that were observed after several days were isolated through decantation of the supernatant and used for single-crystal X-ray diffraction. No further characterisation data was recorded.

Data for [Ir(cyclo-^{t}Bu-PONOP')H(MeCN)][BAr$^F{}_4$]

^{1}H NMR (400.11 MHz, CD_3CN, 298 K) δ 7.93 (t, $^3J_{HH}$ = 8.1, 1H, py), 7.69 (br. m., 8H, Ar$^F{}_4$), 7.67 (s, 4H, Ar$^F{}_4$), 7.06 (d, $^3J_{HH}$ = 8.2, 1H, py), 6.99 (d, $^3J_{HH}$ = 8.2, 1H, py), 1.87 (app. qt, $^2J_{HH}$ = 9.4, $^4J_{HH}$ = 9.2, $^3J_{HP}$ = 1.9, 1H, PC(CH$_3$)$_2$(C*H*$_2$)), 1.50 (d, $^3J_{HP}$ = 20.0, 3H, PC(C*H*$_3$)$_2$(CH$_2$)), 1.42 (d, $^3J_{HP}$ = 16.3, 9H, ^{t}Bu), 1.39 (d, $^3J_{HP}$ = 14.5, 9H, ^{t}Bu), 1.21 (d, $^3J_{HP}$ = 15.1, 9H, ^{t}Bu), 1.02 (d, $^3J_{HP}$ = 15.1, 3H, PC(C*H*$_3$)$_2$(CH$_2$)), 0.17 (dd, $^2J_{HH}$ = 9.2, $^3J_{HP}$ = 2.6, 1H, PC(CH$_3$)$_2$(C*H*$_2$)), −13.99 (app. t, $^2J_{HP}$ = 11.3, 1H).

^{31}P NMR (161.99 MHz, CD_3CN, 298 K) δ 174.8 (d, J_{PP} = 347, *P*^{t}Bu$_2$), 117.1 (d, *J* = 347, *P*C(CH$_2$)(CH$_3$)$_2$).

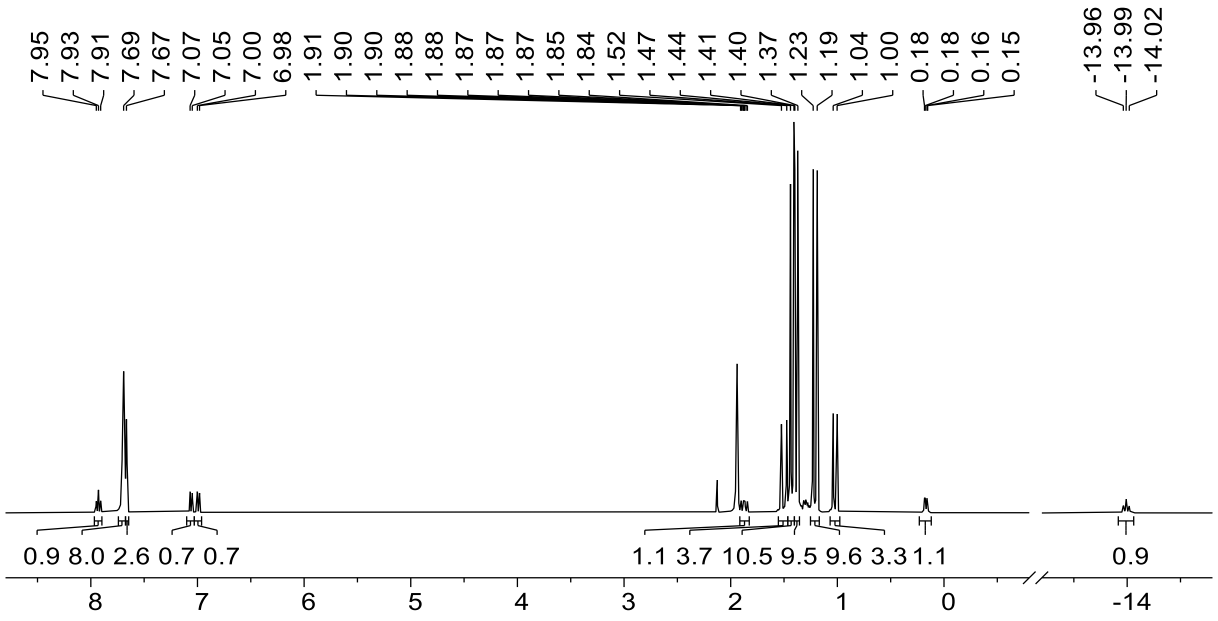

Figure S39. *^{1}H NMR Spectrum of [Ir(cyclo-[t]Bu-PONOP')H(MeCN)][BAr[F]4]* ***2-MeCN[BAr[F]4]*** *(400.11 MHz, CD_3CN, 298 K).*

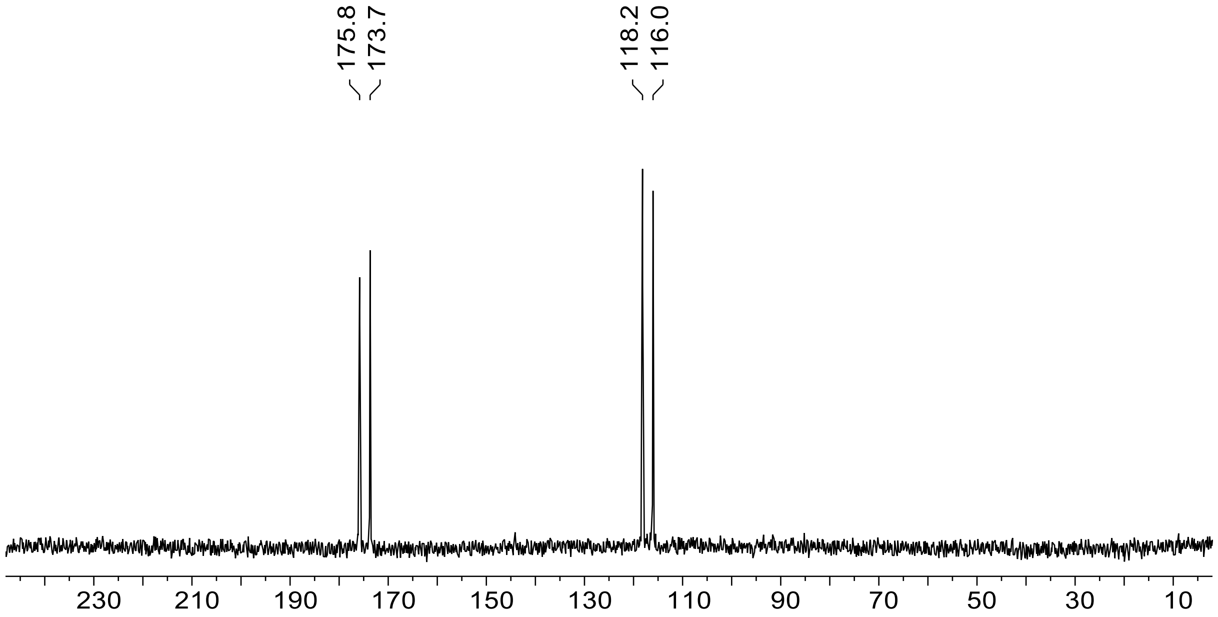

Figure S40. *^{31}P{^{1}H} NMR Spectrum of [Ir(cyclo-[t]Bu-PONOP')H(MeCN)][BAr[F]4]* ***2-MeCN[BAr[F]4]*** *(161.99 MHz, CD_3CN, 298 K).*

Preparation of [Ir(PONOP^{t}Bu)(N_2)][BAr$^F_4$]

Sieved single crystals (0.25-0.5 mm) of [IrH(cycloPONOP^{t}Bu)][BAr$^F_4$] **2[BAr$^F_4$]** (97.4 mg, 66.2 μmol) were heated in an open 25 mL round bottom flask heated in an aluminium block regulated at 80 °C by a fuzzy logic temperature controller inside a dinitrogen filled glovebox (<0.1 ppm H_2O/O_2). Heating was stopped

after 7 days to afford [Ir(^{t}Bu-PONOP)(κ^1-N_2)][BAr$^{F}_4$] **3[BAr$^{F}_4$]** as pale yellow blocks suitable for single crystal x-ray diffraction (98.1 mg, 66.2 μmol, >99%).

Data for [Ir(^{t}Bu-PONOP)(κ^1-N_2)][BAr$^{F}_4$]

Solution phase NMR were recorded at low temperature due to the poor solution phase stability of **3[BAr$^{F}_4$]** at ambient temperature. Complete decomposition to a complicated mixture of products is observed on storage at ambient temperature <24 hours (Figure S44).

^{1}H NMR (500.22 MHz, CD_2Cl_2, 203 K) δ 7.83 (t, $^3J_{HH}$ = 8, 1H, py), 7.71 (br. m., 8H, Ar$^{F}_4$), 7.53 (s, 4H, Ar$^{F}_4$), 7.17 (d, $^3J_{HH}$ = 8, 2H, py), 1.35 (vt, J = 8, 36H, P^{t}Bu$_2$).

^{13}C{^{1}H} NMR (125.80 MHz, CD_2Cl_2, 203 K) δ 164.8 (py C), 161.3 (q, $^1J_{CB}$ = 50 , Ar^F), 144.6 (s, py CH), 134.2 (s br., Ar^F), 128.1 (app q, $^2J_{CF}$ = 31 , Ar^F), 124.0 (q, $^1J_{CF}$ = 272, CF_3), 117.1 (Ar^F), 103.2 (py CH), 41.4 (vt, J = 9, py CH), 26.7 (PC(CH$_3$)$_3$).

^{31}P NMR (202.50 MHz, CD_2Cl_2, 185 K) δ 193.1 (P^tBu$_2$).

^{13}C{^{1}H} CP MAS NMR (20 kHz spin rate, 100.65 MHz, 298 K) δ 166.1-160.9 (Ar^F and py C), 143.8 (py CH), 135.9-116.8 (Ar^F), 103.1-102.3 (py CH), 42.7-41.8 (^{t}Bu C), 27.2-26.1 (^{t}Bu CH).

^{31}P{^{1}H} CP MAS NMR (20 kHz spin rate, 162.06 MHz, 298 K) δ 197.1 (d, $^2J_{PP}$ = 325, P^{t}Bu$_2$), 195.1 (d, $^2J_{PP}$ = 325, P^{t}Bu$_2$).

IR (ATR, diamond) 2158 cm^{-1} (Ir-N≡N)

Elemental analysis found (calculated) for $C_{53}H_{51}BF_{24}IrN_3O_2P_2$: C, 43.19 (42.93); H, 3.48 (3.47); N, 2.77 (2.83).

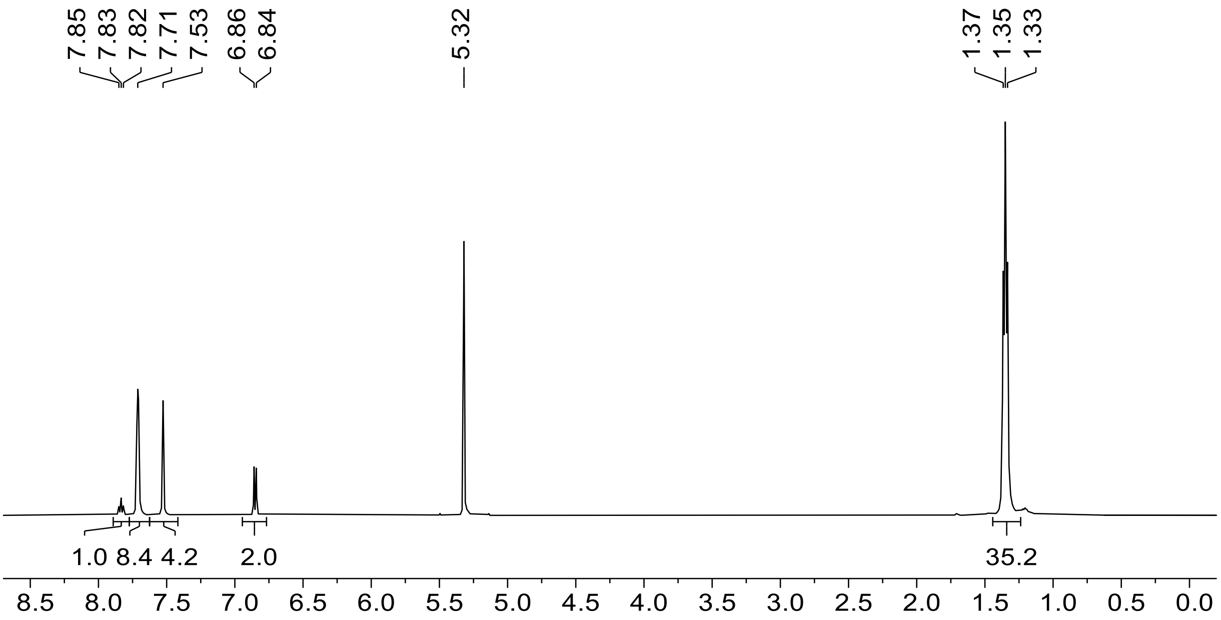

Figure S41. *^{1}H NMR spectrum of [Ir(^{t}Bu-PONOP)(κ^1-N_2)][BAr$^{F}_4$]* ***3[BAr$^{F}_4$]*** *(500.22 MHz, CD_2Cl_2, 203 K).*

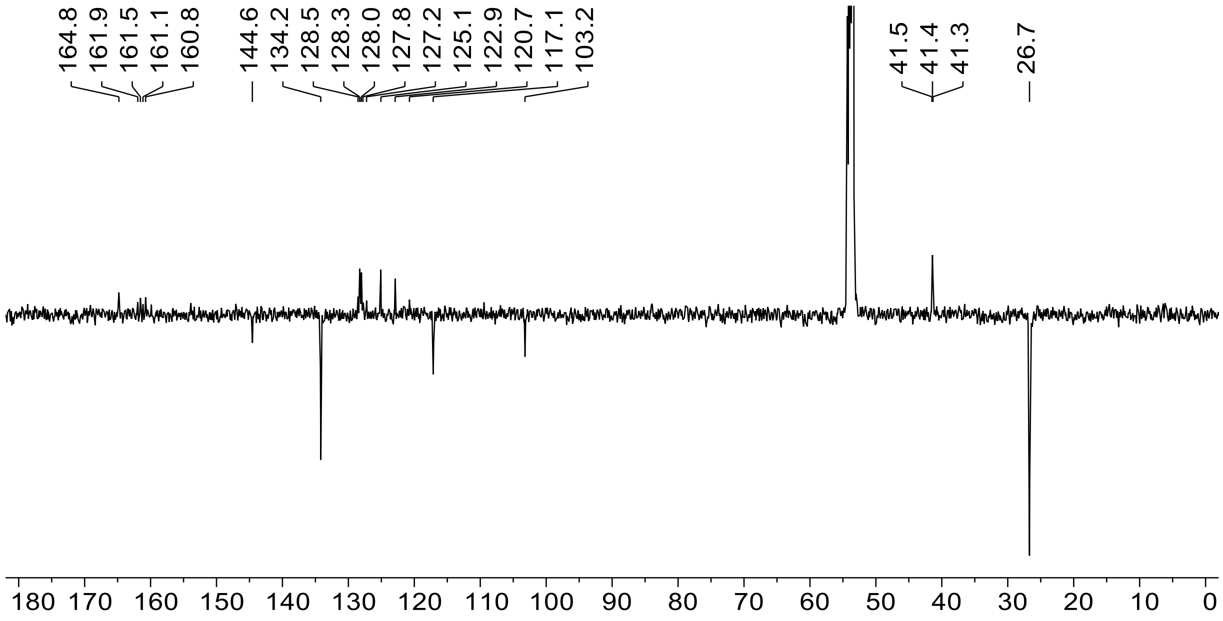

Figure S42. *$^{13}C\{^1H\}$ APT NMR spectrum of [Ir(^{t}Bu-PONOP)(κ^1-N_2)][BAr$^F_4$]* ***3[BAr$^F_4$]*** *(125.80 MHz, CD_2Cl_2, 203 K).*

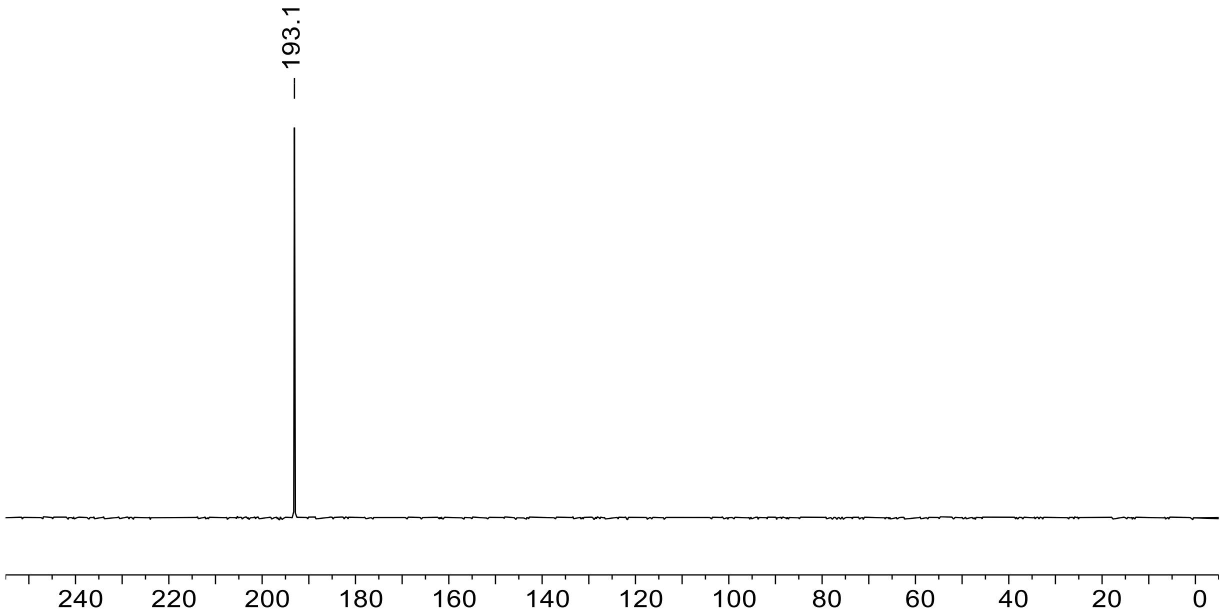

Figure S43. *$^{31}P\{^1H\}$ NMR spectrum of [Ir(^{t}Bu-PONOP)(κ^1-N_2)][BAr$^F_4$]* ***3[BAr$^F_4$]*** *(202.50 MHz, CD_2Cl_2, 185 K).*

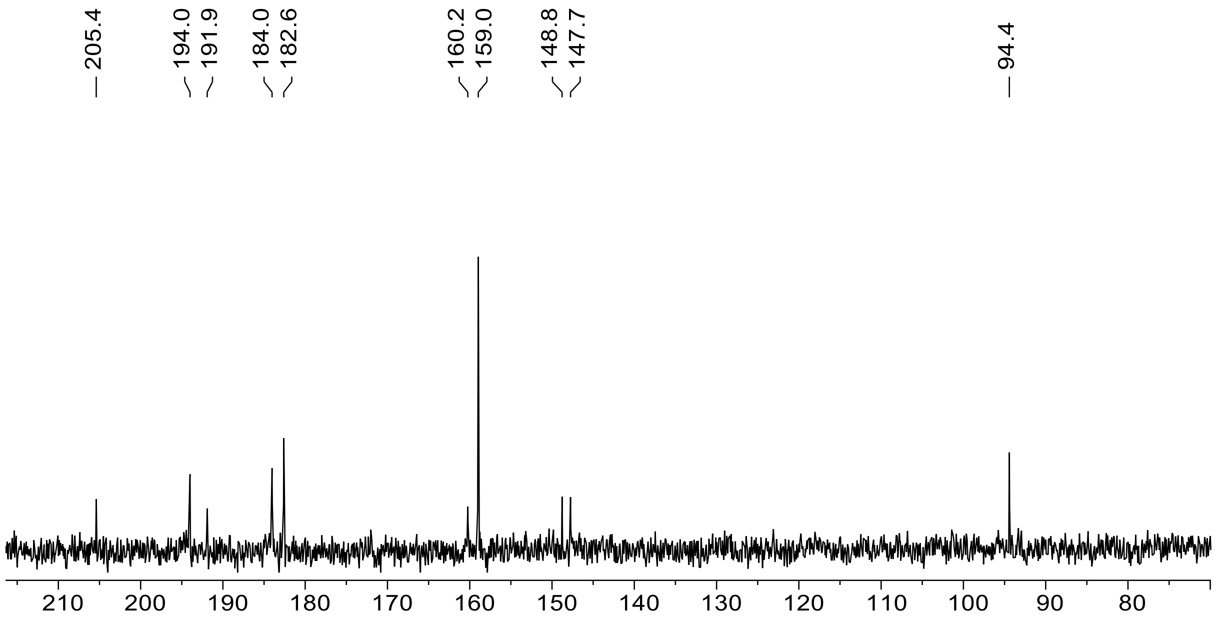

Figure S44. *$^{31}P\{^1H\}$ NMR spectrum of [Ir(^{t}Bu-PONOP)(κ^1-N_2)][BAr$^F_4$]* ***3[BAr$^F_4$]*** *stored in solution at ambient temperature (161.99 MHz, CD_2Cl_2, 298 K).*

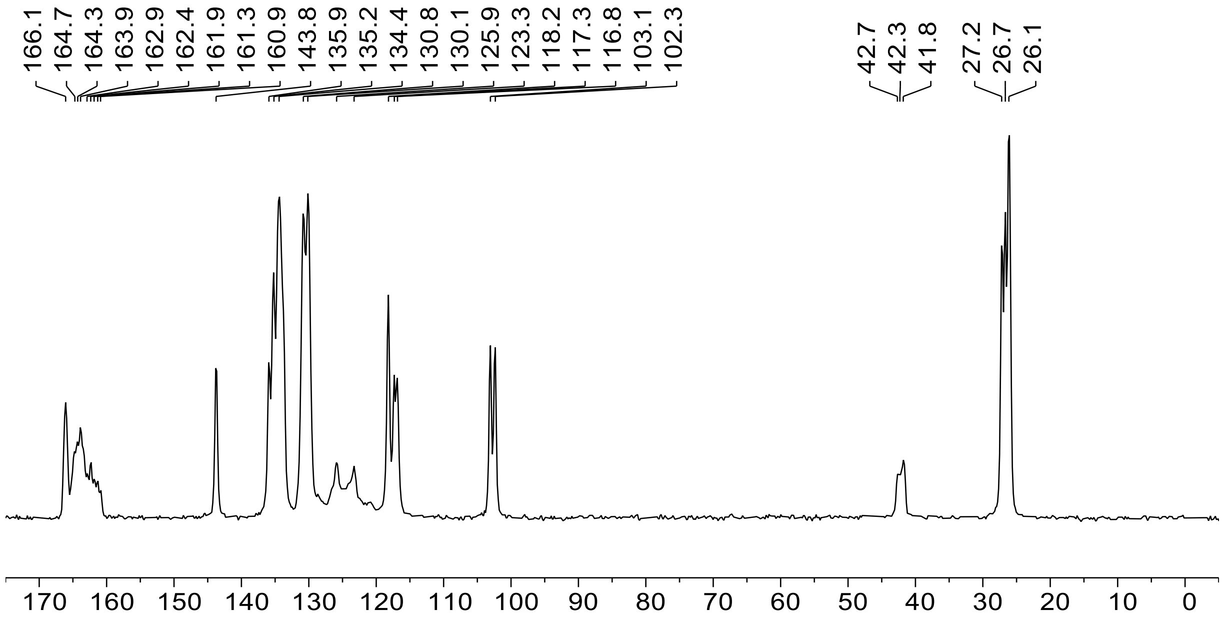

Figure S45. *$^{13}C\{^1H\}$ CP MAS NMR spectrum of [Ir(^{t}Bu-PONOP)(κ^1-N_2)][BAr$^F_4$]* ***3[BAr$^F_4$]****,* *(20 kHz spin rate, 100.65 MHz, 298 K).*

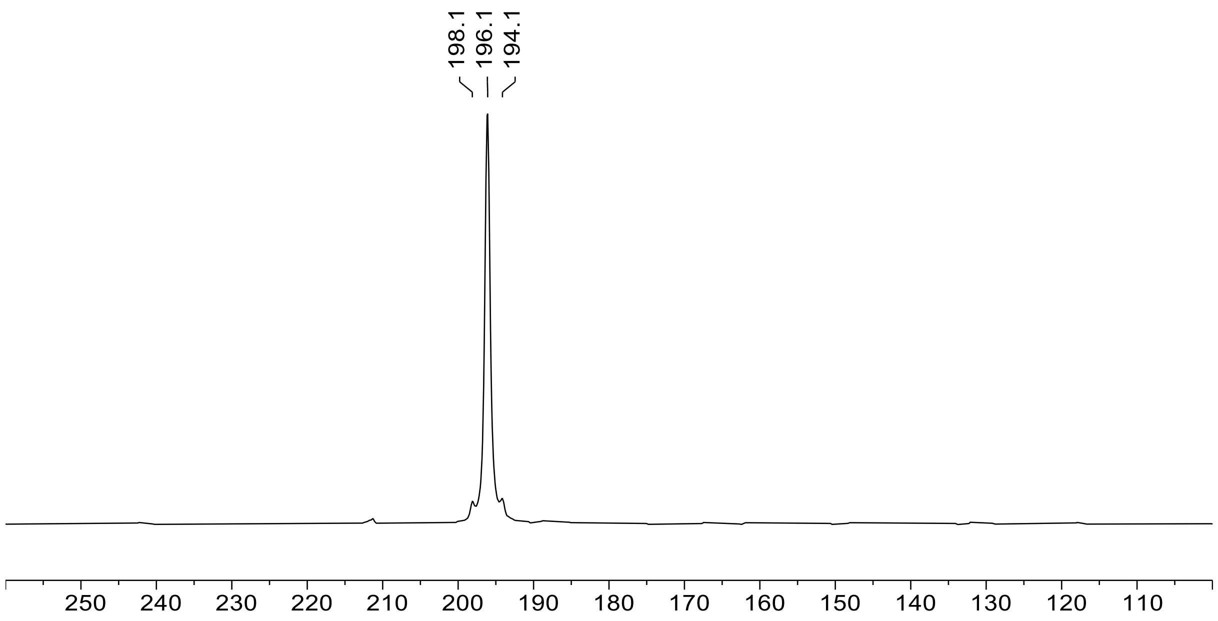

Figure S46. *$^{31}P\{^1H\}$ CP MAS NMR spectrum of [Ir(^{t}Bu-PONOP)(κ^1-N_2)][$BAr^F{}_4$]* ***3[BAr$^F{}_4$]*** *(20 kHz spin rate, 162.06 MHz, 298 K).*

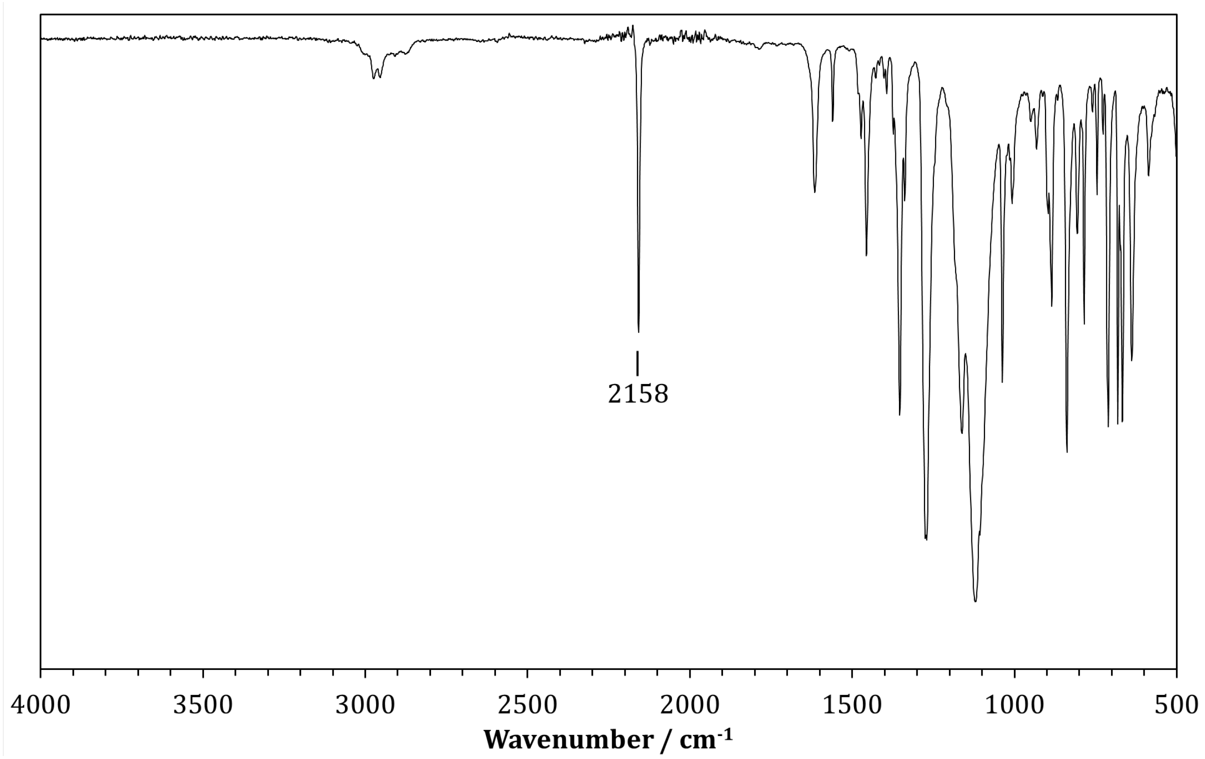

Figure S47. *FT-IR spectrum of [Ir(^{t}Bu-PONOP)(κ^1-N_2)][$BAr^F{}_4$]* ***3[BAr$^F{}_4$]*** *collected by ATR at ambient temperature.*

Solid/Gas Reaction of [Ir(cyclo-^{t}Bu-PONOP')H][$BAr^F{}_4$] with Carbon Monoxide

An NMR tube filled with single crystals of [Ir(cyclo-^{t}Bu-PONOP')H][$BAr^F{}_4$] **2[BAr$^F{}_4$]** (17.0 mg, 11.7 µmol) was evacuated, repressurised with carbon monoxide (1 bar gauge) and stored at room temperature for 24 hours. The carbon monoxide gas was removed *in vacuo*, the crystals were assayed by $^{13}C\{^1H\}$ and $^{31}P\{^1H\}$ SS NMR spectroscopy under argon and then dissolved by condensation of CD_2Cl_2 (*ca.* 0.5 mL) on the high

vacuum line and the contents assayed by NMR spectroscopy at 298 K. Both sets of data are consistent with the formation of [Ir(^{t}Bu-PONOP)(CO)][$BAr^F{}_4$] **4[BAr$^F{}_4$]** as has been previously reported.[11]

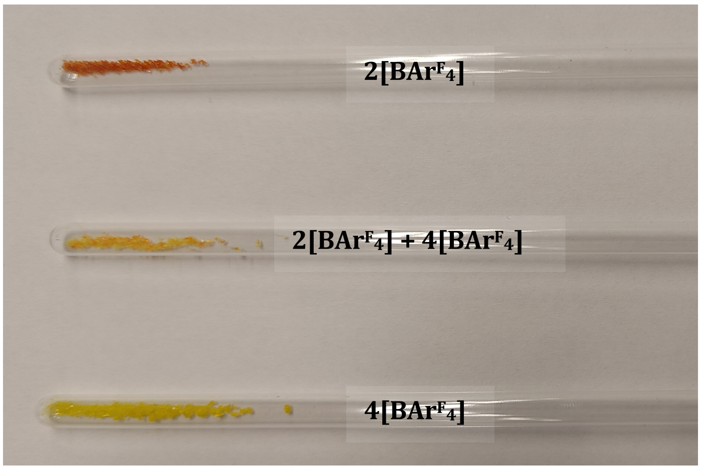

Figure S48. *Photograph showing the colour change observed over 24 hours on addition of carbon monoxide to [Ir(cyclo-^{t}Bu-PONOP')H][$BAr^F{}_4$]* ***2[BAr$^F{}_4$]*** *to form [Ir(^{t}Bu-PONOP)(CO)][$BAr^F{}_4$]* ***4[BAr$^F{}_4$]***.

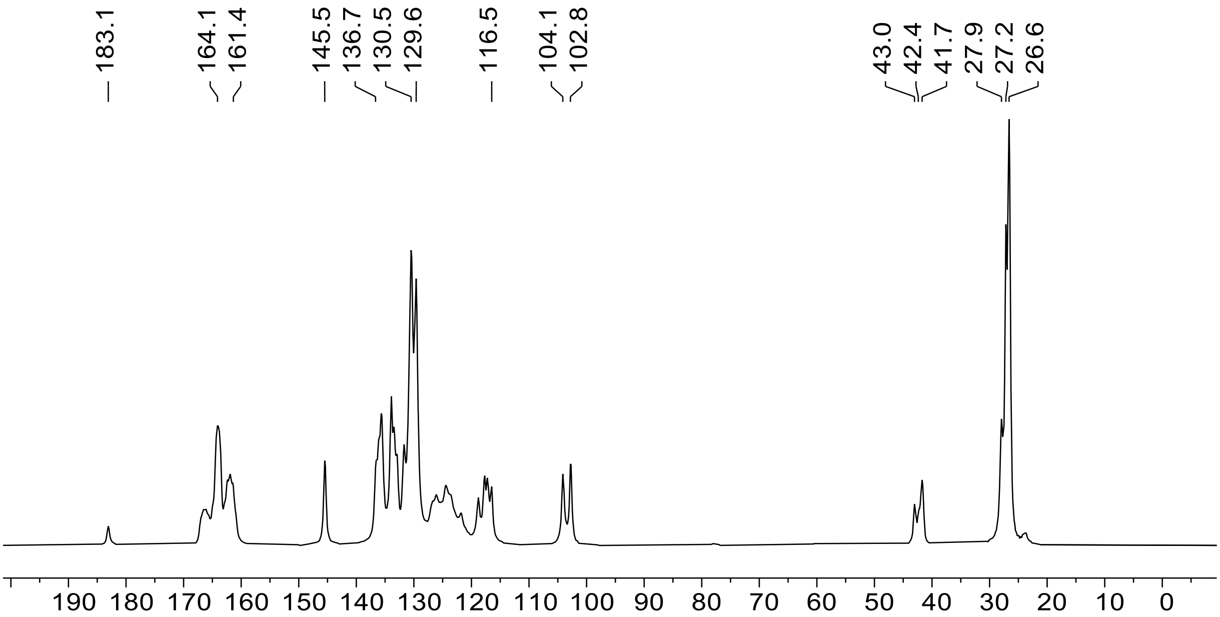

Figure S49. *$^{13}C\{^1H\}$ CP MAS NMR spectrum of [Ir(^{t}Bu-PONOP)(CO)][$BAr^F{}_4$]* ***4[BAr$^F{}_4$]*** *from the solid/gas reaction of [Ir(cyclo-^{t}Bu-PONOP')H][$BAr^F{}_4$]* ***2[BAr$^F{}_4$]*** *and carbon monoxide (20 kHz spin rate, 100.66 MHz, 298 K).*

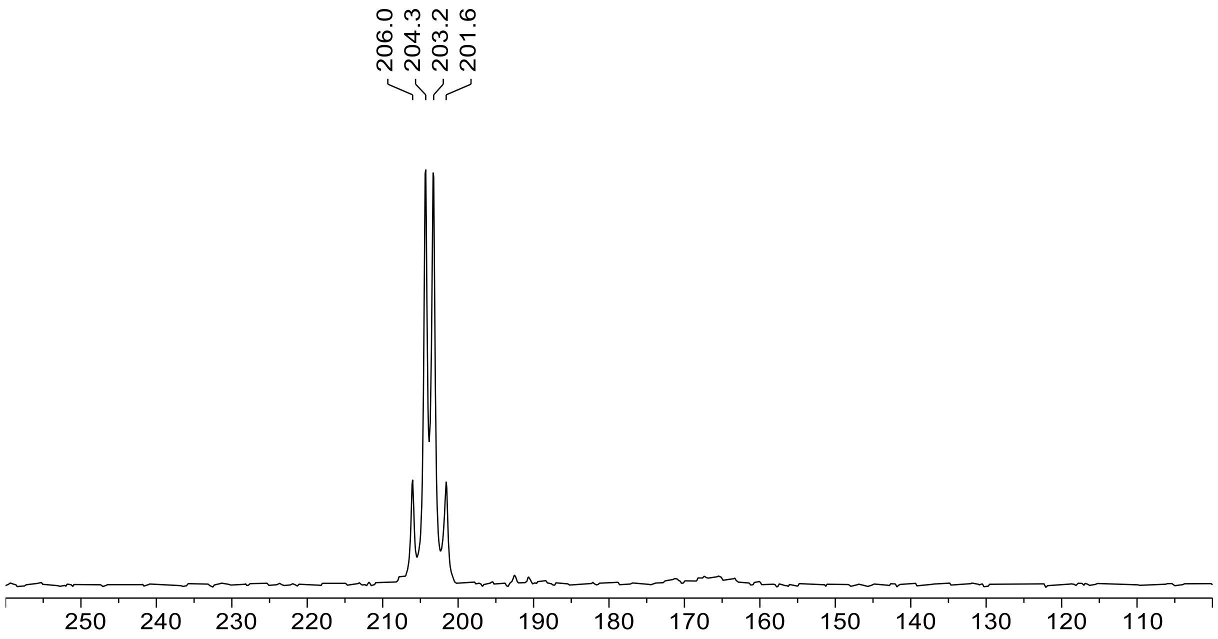

Figure S50. *$^{31}P\{^1H\}$ CP MAS NMR spectrum of [Ir(^{t}Bu-PONOP)(CO)][BAr$^F_4$]* ***4[BAr$^F_4$]*** *from the solid/gas reaction of [Ir(cyclo-^{t}Bu-PONOP')H][BAr$^F_4$]* ***2[BAr$^F_4$]*** *and carbon monoxide (20 kHz spin rate, 162.06 MHz, 298 K).*

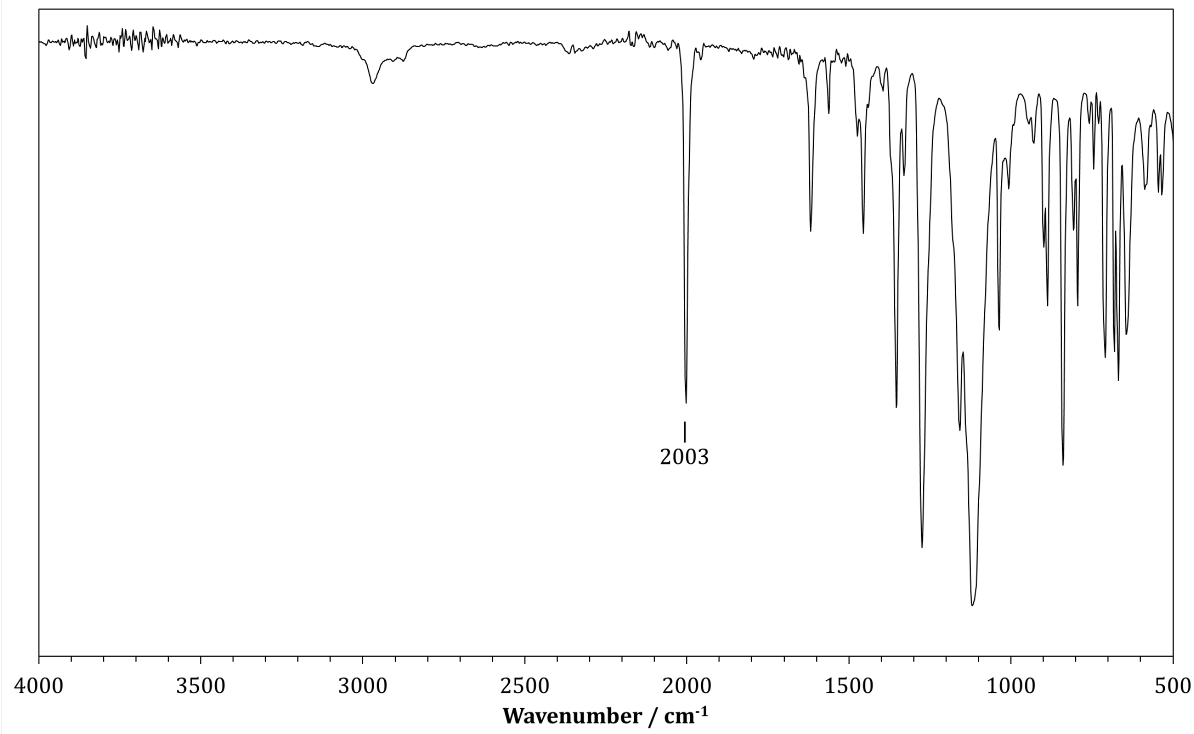

Figure S51. *FT-IR spectrum of [Ir(^{t}Bu-PONOP)(CO)][BAr$^F_4$]* ***4[BAr$^F_4$]*** *from the solid/gas reaction of [Ir(cyclo-^{t}Bu-PONOP')H][BAr$^F_4$]* ***2[BAr$^F_4$]*** *and carbon monoxide collected by ATR at ambient temperature.*

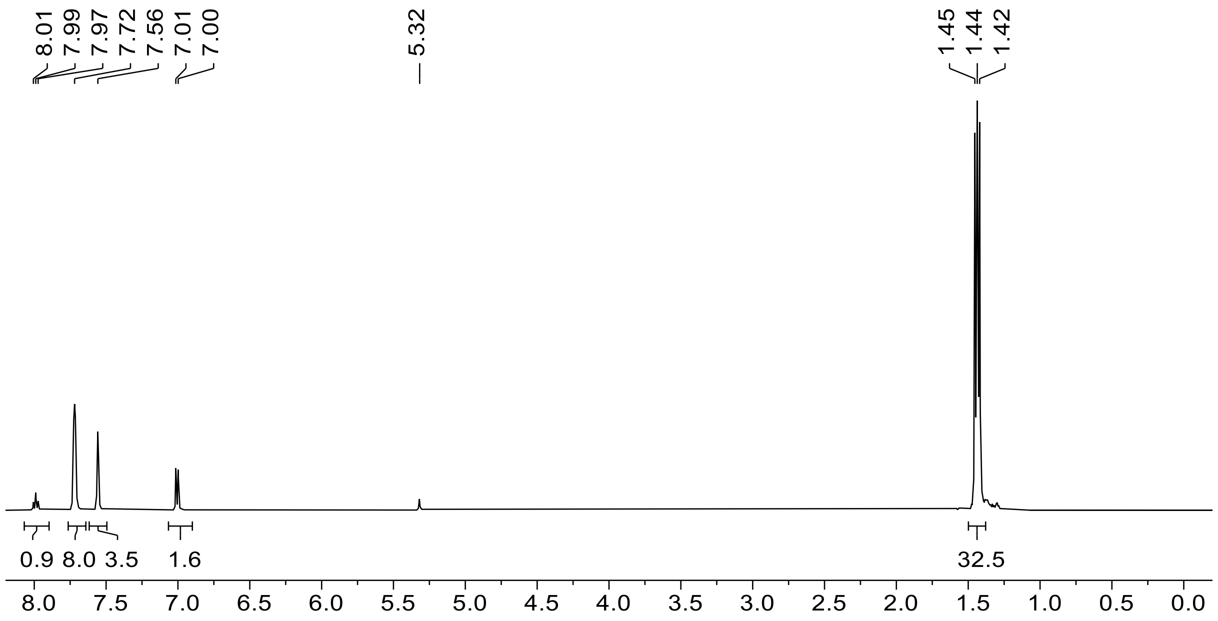

Figure S52. *^{1}H NMR spectrum of [Ir(^{t}Bu-PONOP)(CO)][BAr$^{F}_{4}$]* ***4[BAr$^{F}_{4}$]*** *from the solid/gas reaction of [Ir(cyclo-^{t}Bu-PONOP')H][BAr$^{F}_{4}$]* ***2[BAr$^{F}_{4}$]*** *and carbon monoxide (500.12 MHz, CD_2Cl_2, 298 K).*

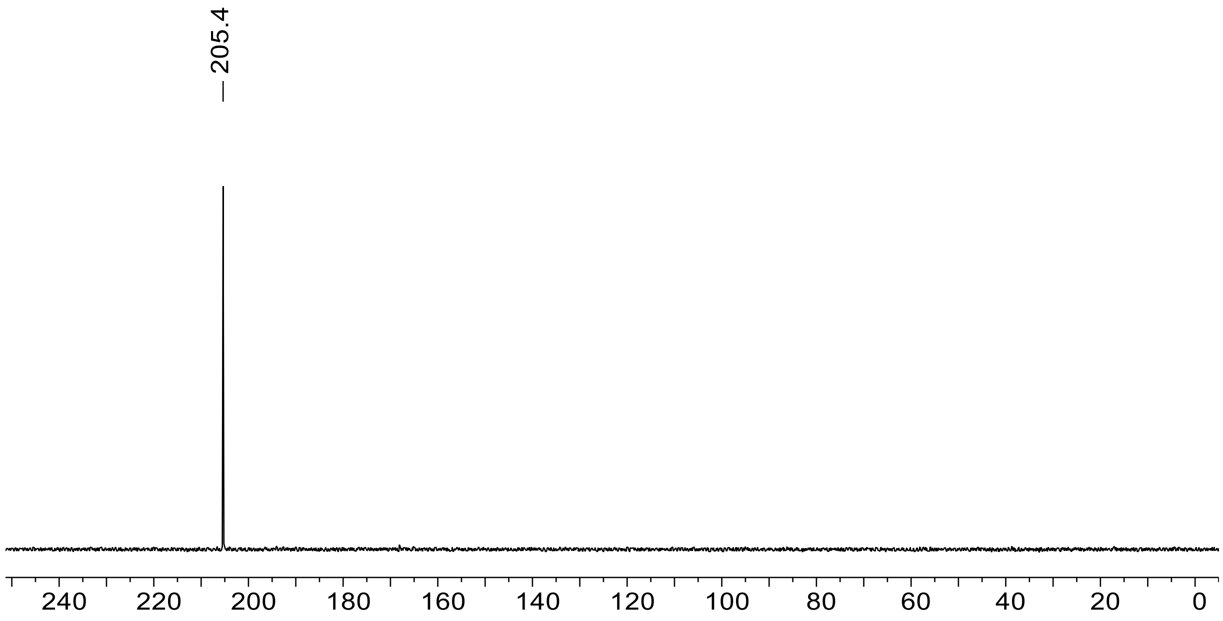

Figure S53. *$^{31}P\{^{1}H\}$ NMR spectrum of [Ir(^{t}Bu-PONOP)(CO)][BAr$^{F}_{4}$]* ***4[BAr$^{F}_{4}$]*** *from the solid/gas reaction of [Ir(cyclo-^{t}Bu-PONOP')H][BAr$^{F}_{4}$]* ***2[BAr$^{F}_{4}$]*** *and carbon monoxide (202.47 MHz, CD_2Cl_2, 298 K).*

Variable Pressure Solid/Gas Reactions of [Ir(cyclo-^{t}Bu-PONOP')H][BAr$^{F}_{4}$] with CH_4

5 mm heavy-wall NMR tubes containing single crystals of [IrH(cycloPONOP^{t}Bu)][BAr$^{F}_{4}$] **2[BAr$^{F}_{4}$]** (3.6-4.7 mg, 2.5-3.2 μmol) were evacuated and pressurised with CH_4 (2, 4, 6 and 8 bar gauge). The tubes were sealed and then heated at 80 °C for 8 or 24 hours, depressurised and the remaining gases removed *in vacuo*. The crystals were dissolved by condensation of CD_2Cl_2 *in vacuo* and assayed by ^{1}H and $^{31}P\{^{1}H\}$ NMR spectroscopy at 183 K. A pressure dependent partial conversion of 2[BAr$^{F}_{4}$] into 1[BAr$^{F}_{4}$] was observed and the relative fraction of each is listed in Table S1-S3

Spectroscopic Data After 8 Hours

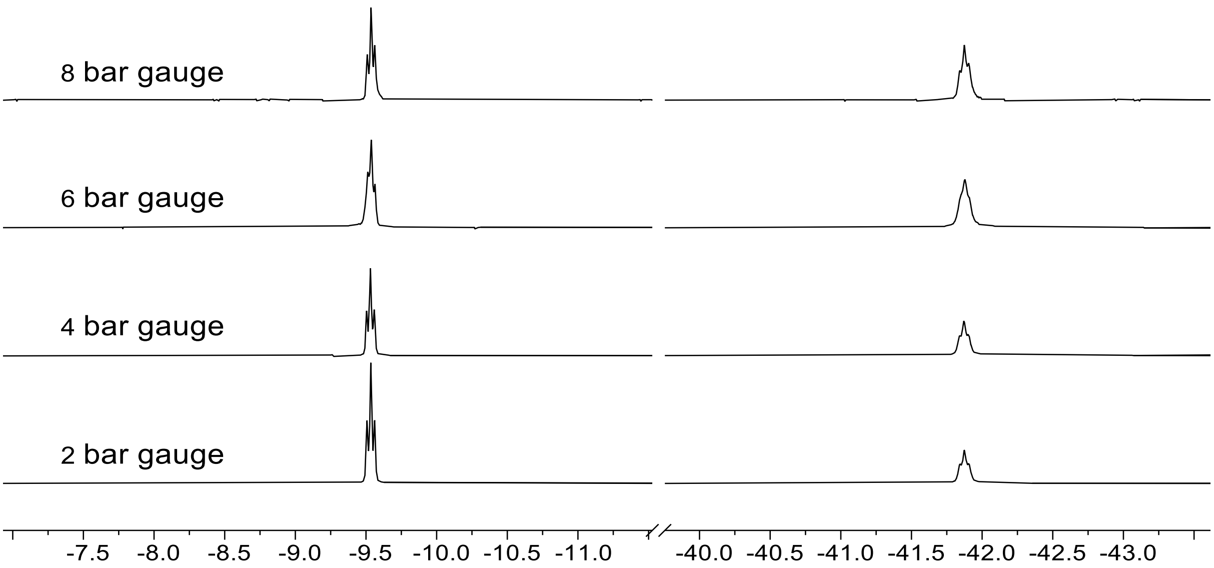

Figure S54. *Stacked ^{1}H NMR spectra of the products of the solid/gas reaction between [Ir(cyclo-^{t}Bu-PONOP')H][BAr$^{F}_{4}$]* ***2[BAr$^{F}_{4}$]*** *and CH_4 after 8 hours (400.11 MHz, CD_2Cl_2, 183 K).*

	2 bar gauge	4 bar gauge	6 bar gauge	8 bar gauge
**1[BAr$^{F}_{4}$]**	35	44	48	51
**2[BAr$^{F}_{4}$]**	65	56	52	49

Table S1*. Relative percentages of [Ir(^{t}Bu-PONOP)MeH][BAr$^{F}_{4}$]* ***1[BAr$^{F}_{4}$]*** *and [Ir(cyclo-^{t}Bu-PONOP')H][BAr$^{F}_{4}$]* ***2[BAr$^{F}_{4}$]*** *after 8 hours under variable CH_4 pressure.*

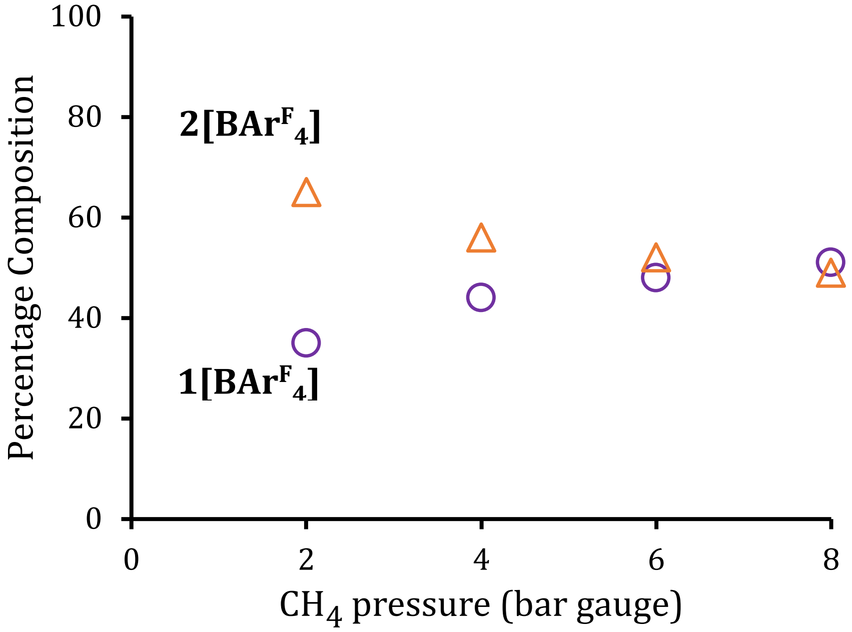

Figure S55. *Plot of CH_4 pressure dependency on conversion of [Ir(cyclo-^{t}Bu-PONOP')H][BAr$^{F}_{4}$]* ***2[BAr$^{F}_{4}$]*** *to [Ir(^{t}Bu-PONOP)MeH][BAr$^{F}_{4}$]* ***1[BAr$^{F}_{4}$]*** *as measured from the ^{1}H NMR spectrum after 8 hours at 80 °C.*

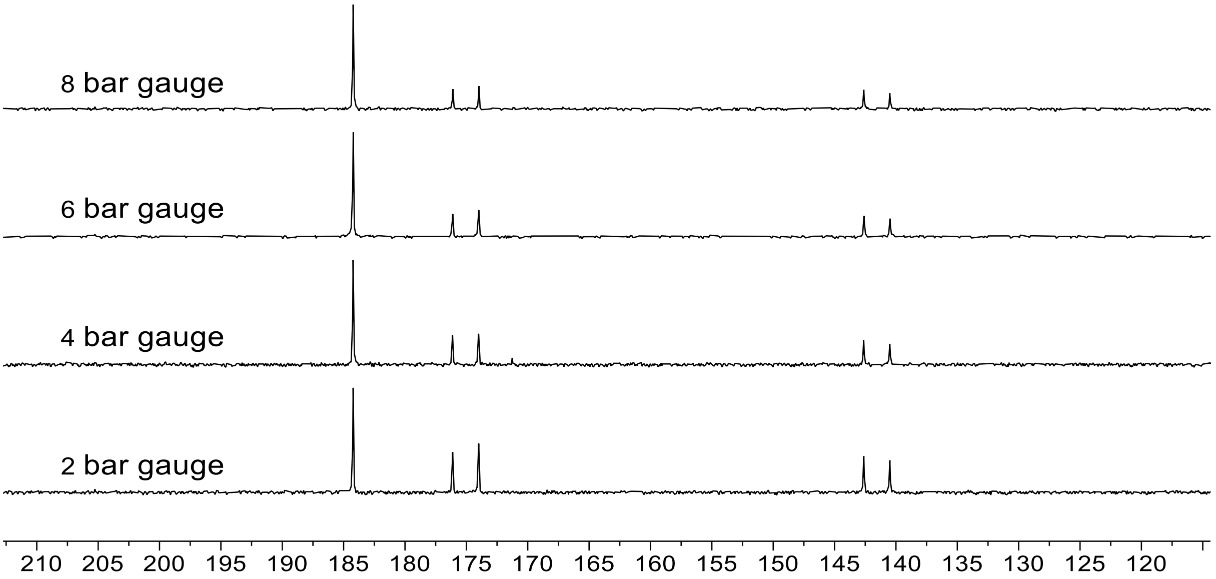

Figure S56. *Stacked $^{31}P\{^1H\}$ NMR spectra of the products of the solid/gas reaction between [Ir(cyclo-^{t}Bu-PONOP')H][BAr$^F{}_4$]* ***2[BAr$^F{}_4$]*** *and CH_4 after 8 hours (161.99 MHz, CD_2Cl_2, 183 K).*

Spectroscopic Data After 24 Hours

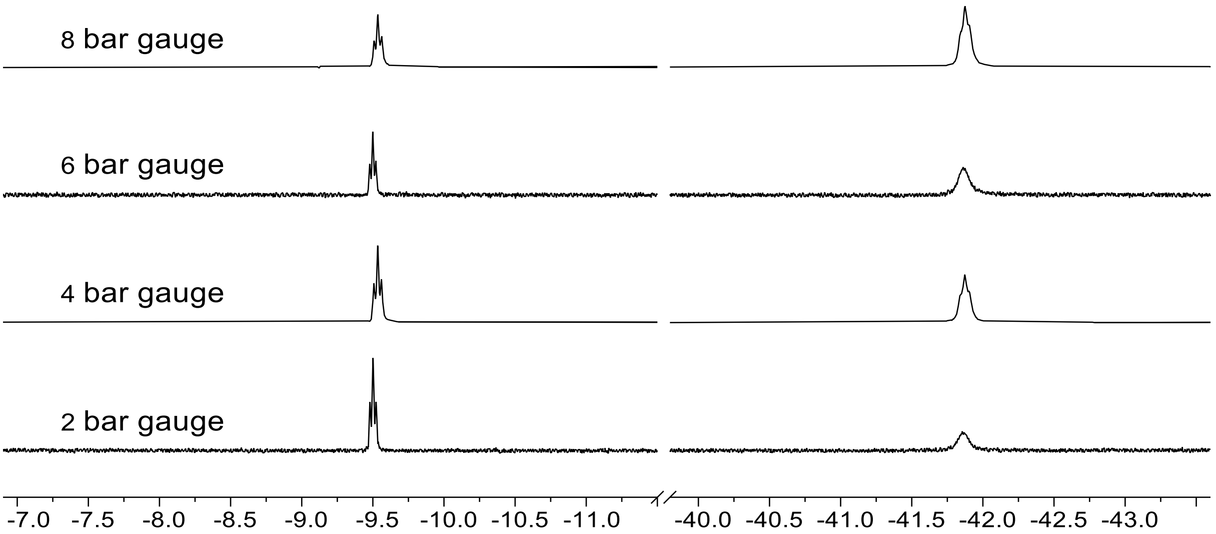

Figure S57. *Stacked ^1H NMR spectra of the products of the solid/gas reaction between [Ir(cyclo-^{t}Bu-PONOP')H][BAr$^F{}_4$]* ***2[BAr$^F{}_4$]*** *and CH_4 after 24 hours (400.11 MHz, CD_2Cl_2, 183 K).*

	2 bar gauge	4 bar gauge	6 bar gauge	8 bar gauge
**1[BAr$^F{}_4$]**	40	55	61	68
**2[BAr$^F{}_4$]**	60	45	39	32

Table S2*. Relative percentages of* ***1[BAr$^F{}_4$]*** *and* ***2[BAr$^F{}_4$]*** *after 24 hours under variable CH_4 pressure.*

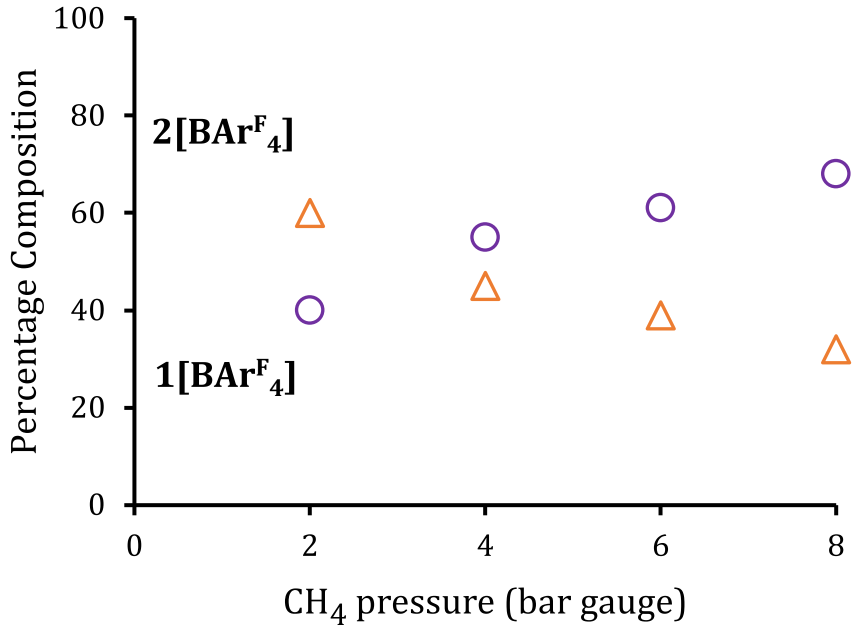

Figure S58. *Plot of CH_4 pressure dependency on conversion of [Ir(cyclo-tBu-PONOP')H][BArF4]* ***2[BArF4]*** *to [Ir(tBu-PONOP)MeH][BArF4]* ***1[BArF4]*** *as measured from the 1H NMR spectrum after 24 hours at 80 °C.*

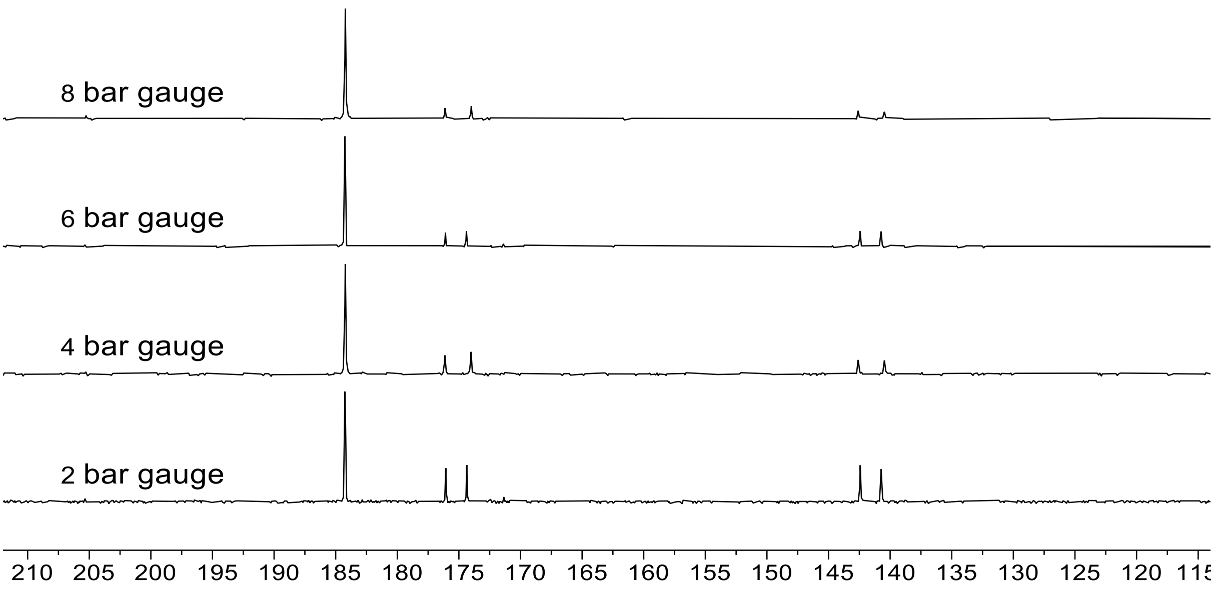

Figure S59. *Stacked $^{31}P\{^1H\}$ NMR spectra of the products of the solid/gas reaction between [Ir(cyclo-tBu-PONOP')H][BArF4]* ***2[BArF4]*** *and CH_4 after 24 hours (161.99 MHz, CD_2Cl_2, 183 K).*

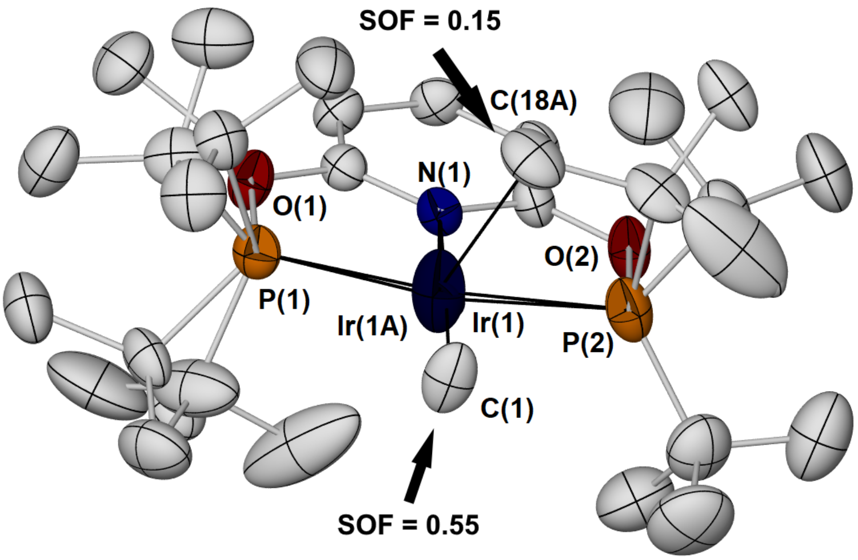

Figure S60. *A representation of the partial model from single-crystal X-ray diffraction experiments conducted on crystals of **2[BAr$^F_4$]** thermolysed under CH_4 (8 bar gauge) for 24 hours. The model is consistent with a superposition of **1[BAr$^F_4$]** and **2[BAr$^F_4$]** structures and refinement statistics are indicative of good quality data (R_1 = 4.04%, R_{int} = 3.30%, GooF = 1.025) however the model is not chemically complete (e.g. C(1) occupancy = 55%, C(18A) occupancy = 15%) and there is insufficient residual electron density (Largest residual diffraction peak = 1.4 e Å^{-3}) localised in a chemically sensible position to include an additional methylene site (C(18B)) to pair with the modelled site (C(18A)).*

	2 bar gauge	4 bar gauge	6 bar gauge	8 bar gauge
1[BAr$^F_4$]	39	57	64	68
2[BAr$^F_4$]	61	43	36	32

Table S3. *Relative percentages of **1[BAr$^F_4$]** and **2[BAr$^F_4$]** after 48 hours under variable CH_4 pressure.*

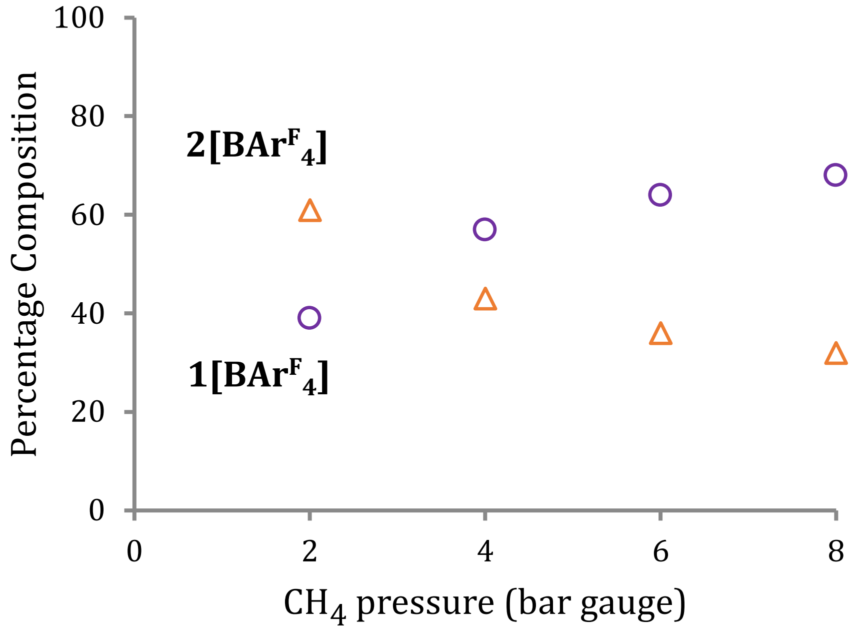

Figure S61. *Plot of CH_4 pressure dependency on conversion of [Ir(cyclo-^{t}Bu-PONOP')H][BAr$^F_4$] **2[BAr$^F_4$]** to [Ir(^{t}Bu-PONOP)MeH][BAr$^F_4$] **1[BAr$^F_4$]** as measured from the ^{1}H NMR spectrum after 48 hours at 80 °C.*

Solid/Gas Reaction of [Ir(^{t}Bu-PONOP)MeH][BAr$^F_4$] with CH_4

A 5 mm heavy-wall NMR tube containing single crystals of [Ir(^{t}Bu-PONOP)MeH][BAr$^F_4$] **1[BAr$^F_4$]** (5.7 mg, 3.9 μmol) was evacuated and pressurised with CH_4 (8 bar gauge). The tube was sealed and heated at 80 °C for 24 hours, depressurised and remaining gases removed *in vacuo*. The crystals were dissolved by condensation of CD_2Cl_2 *in vacuo* and assayed by ^{1}H and ^{31}P{^{1}H} NMR spectroscopy at 193 K. The ratio **1[BAr$^F_4$]**:**2[BAr$^F_4$]** was measured from ^{1}H NMR spectroscopy of the hydride integrals to be 2.2:1.

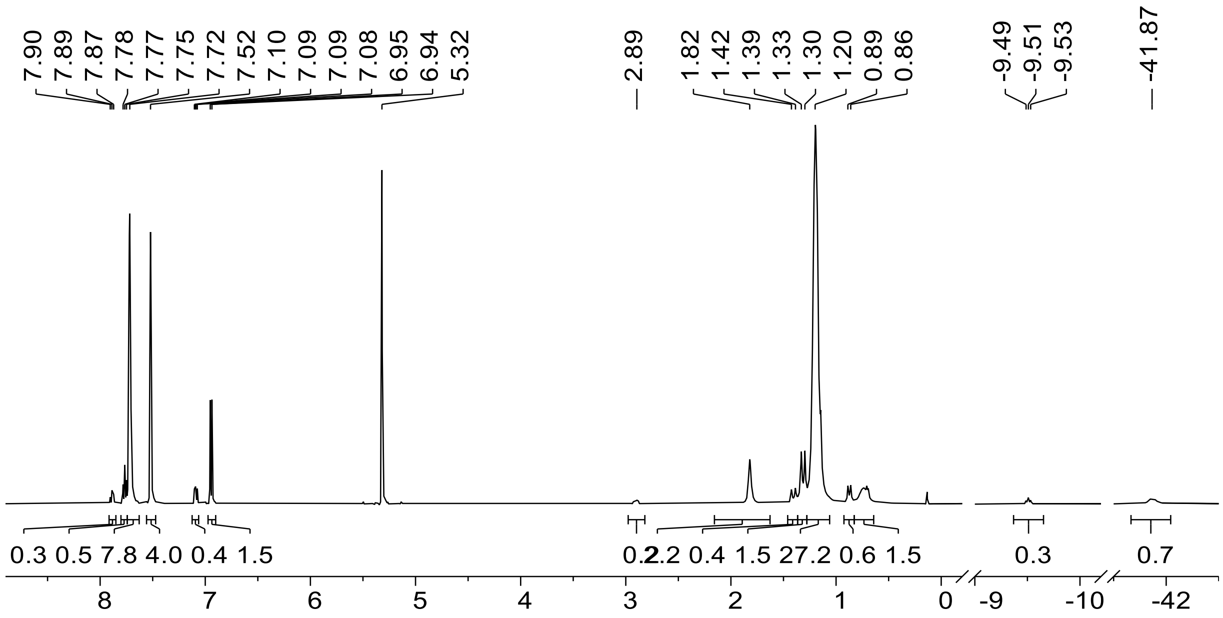

Figure S62. *^{1}H NMR spectrum of the products of the solid/gas reaction between [Ir(^{t}Bu-PONOP)MeH][BAr$^F_4$]* ***1[BAr$^F_4$]*** *and CH_4 (500.22 MHz, CD_2Cl_2, 193 K).*

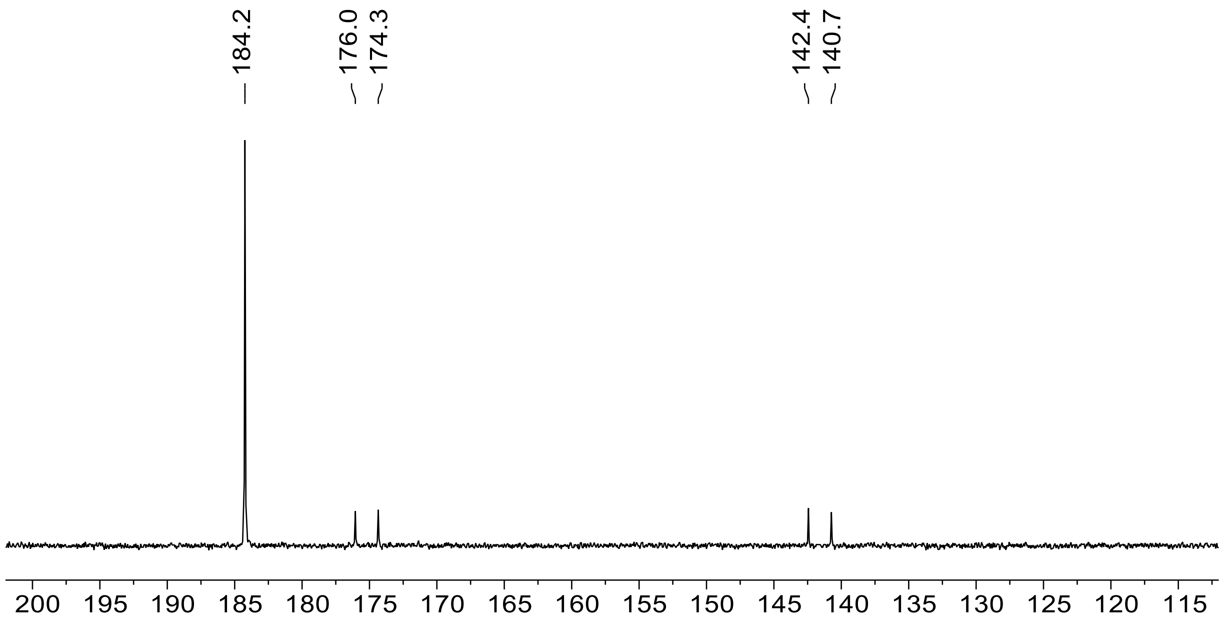

Figure S63. *^{31}P{^{1}H} NMR spectrum of the products of the solid/gas reaction between [Ir(^{t}Bu-PONOP)MeH][BAr$^F_4$]* ***1[BAr$^F_4$]*** *and CH_4 (202.50 MHz, CD_2Cl_2, 193 K).*

Reversibility of Methane Oxidative Addition and Reductive Elimination

A 5 mm heavy-wall NMR tube containing single crystals of $[Ir(cyclo\text{-}^tBu\text{-}PONOP')H][BAr^F_4]$ **2[BAr^F^4]** (18.9 mg, 13.0 µmol) was evacuated and pressurised with CH_4 (8 bar gauge). The tube was sealed and heated at 80 °C for 24 hours, depressurised and the remaining gases removed *in vacuo*. Some crystalline material was removed at this point and assayed by $^{13}C\{^1H\}$ and $^{31}P\{^1H\}$ SS NMR spectroscopy. A mixture of $[Ir(^tBu\text{-}PONOP)MeH][BAr^F_4]$ **1[BAr^F^4]** and **2[BAr^F^4]** (with some decomposition to $[Ir(^tBu\text{-}PONOP)H_2][BAr^F_4]$ **6[BAr^F^4]**[12]) was observed in both spectra (Figure S64 and Figure S65). The remaining crystalline material was evacuated (<5 x 10^{-6} mbar) on a greaseless high-vacuum line for 24 hours before being heated at 80 °C for 24 hours and then assayed by $^{13}C\{^1H\}$ and $^{31}P\{^1H\}$ SS NMR spectroscopy. **1[BAr^F^4]** was observed as the major product (Figure S66 and Figure S67).

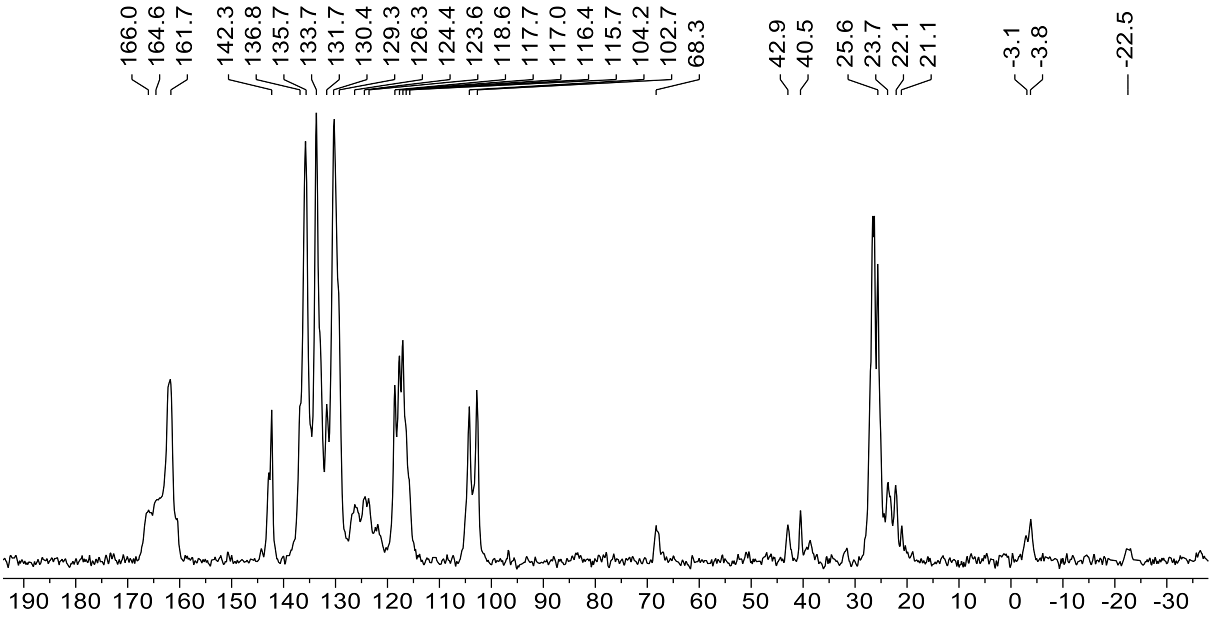

Figure S64. *$^{13}C\{^1H\}$ CP MAS NMR spectrum recorded after heating $[Ir(cyclo\text{-}^tBu\text{-}PONOP')H][BAr^F_4]$* ***2[BAr^F^4]*** *under 8 bar CH_4 for 24 hours (20 kHz spin rate, 100.66 MHz, 298 K).*

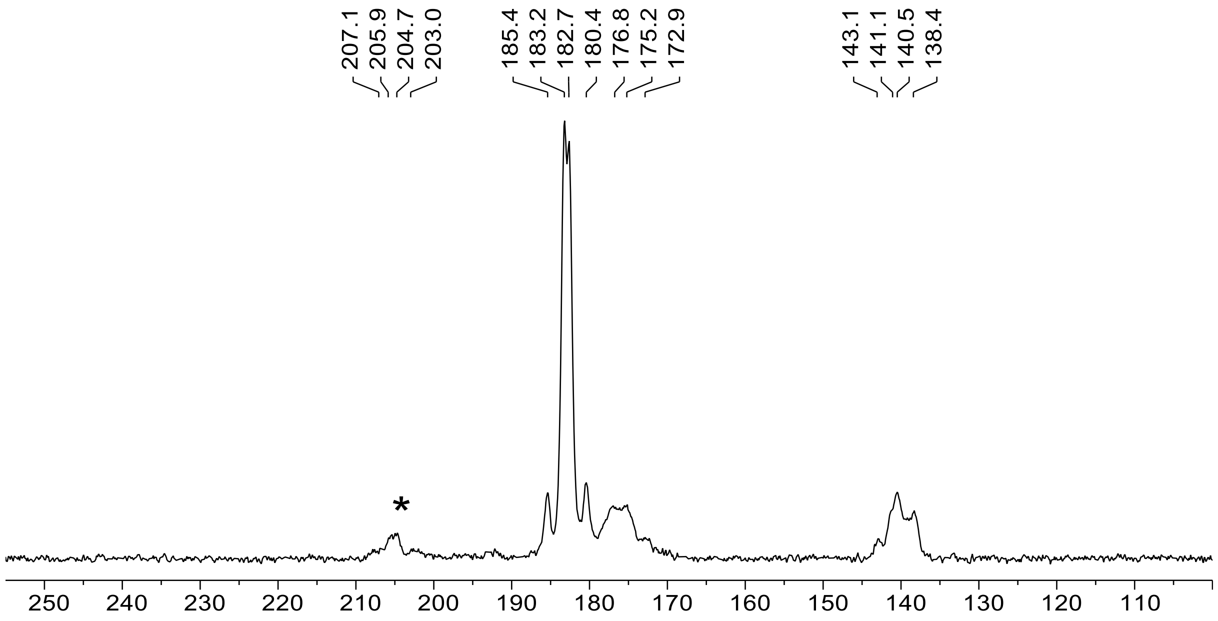

Figure S65. *$^{31}P\{^{1}H\}$ CP MAS NMR spectrum recorded after heating [Ir(cyclo-^{t}Bu-PONOP')H][BAr$^{F}_{4}$]* ***2[BAr$^{F}_{4}$]*** *under 8 bar CH_4 for 24 hours (20 kHz spin rate, 162.06 MHz, 298 K). [Ir(^{t}Bu-PONOP)H_2][BAr$^{F}_{4}$] is indicated by *.*

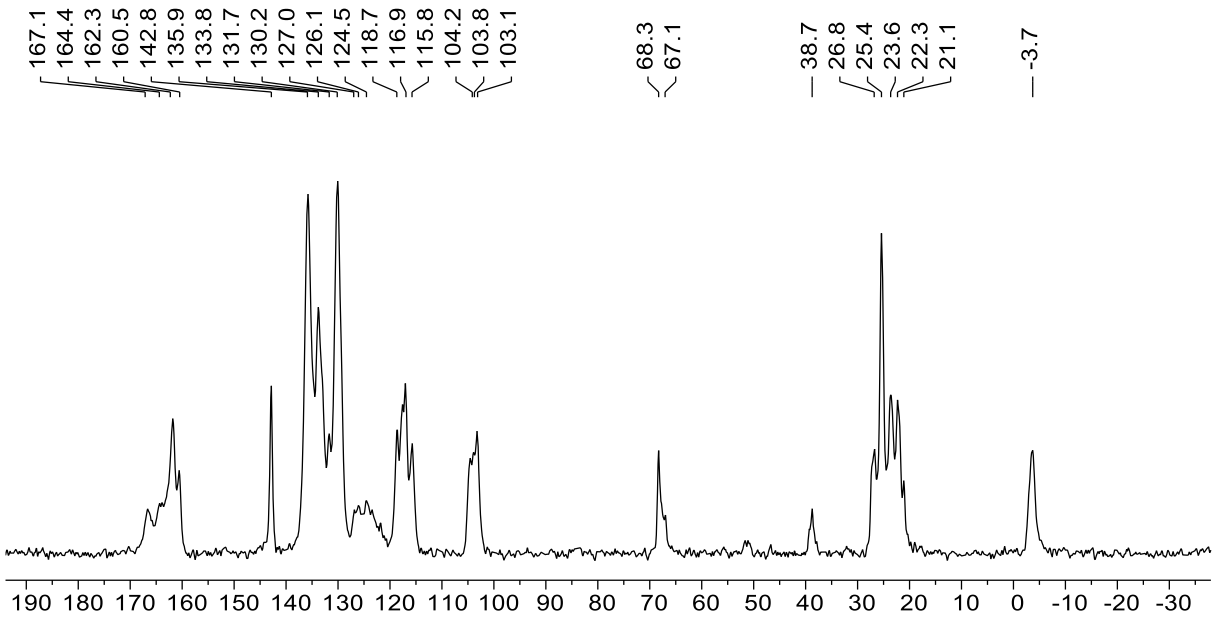

Figure S66. *$^{13}C\{^{1}H\}$ CP MAS NMR spectrum recorded after heating the mixture of [Ir(^{t}Bu-PONOP)MeH][BAr$^{F}_{4}$]* ***1[BAr$^{F}_{4}$]*** *and [Ir(cyclo-^{t}Bu-PONOP')H][BAr$^{F}_{4}$]* ***2[BAr$^{F}_{4}$]*** *under vacuum for 24 hours (20 kHz spin rate, 100.66 MHz, 298 K).*

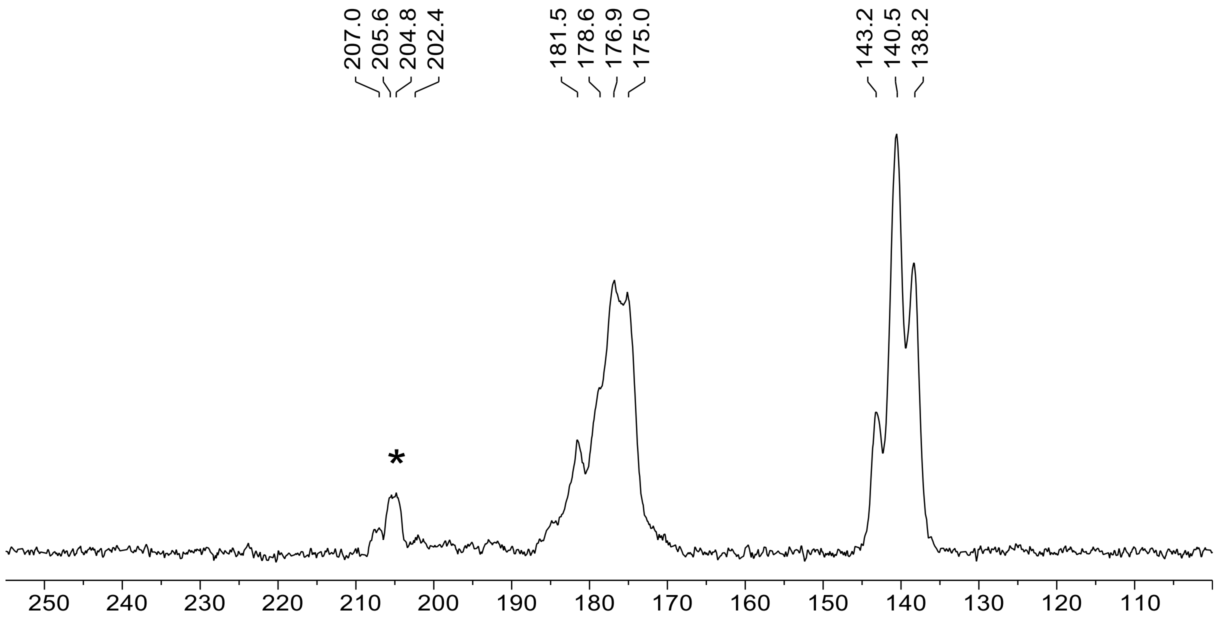

Figure S67. *$^{31}P\{^1H\}$ CP MAS NMR spectrum recorded after heating the mixture of [Ir(^{t}Bu-PONOP)MeH][BAr$^F_4$]* ***1[BAr$^F_4$]*** *and [Ir(cyclo-^{t}Bu-PONOP')H][BAr$^F_4$]* ***2[BAr$^F_4$]*** *under vacuum for 24 hours (20 kHz spin rate, 162.06 MHz, 298 K). [Ir(^{t}Bu-PONOP)H_2][BAr$^F_4$] is indicated by *.*

Solid/Gas Reaction of [Ir(cyclo-^{t}Bu-PONOP')H][BAr$^F_4$] with CD_4

A 5 mm heavy-wall NMR tube containing single crystals of [Ir(cyclo-^{t}Bu-PONOP')H][BAr$^F_4$] **2[BAr$^F_4$]** (5.4 mg, 3.7 µmol) was evacuated on the high vacuum line for three hours at ambient temperature, pressurised with CD_4 (8 bar gauge), then sealed and heated at 80 °C for 24 hours. The tube was then depressurised and remaining gases removed *in vacuo* and the tube back-filled with argon. The crystals were dissolved by condensation of CD_2Cl_2 *in vacuo* and assayed by ^1H and $^{31}P\{^1H\}$ NMR spectroscopy at 183 K (Figure S68 and Figure S69). The solvent was then removed on the high vacuum line while maintaining a sample temperature of −78 °C. Carbon monoxide (1 bar gauge) was then added and the tube stored at ambient temperature for 24 hours. All gases were removed *in vacuo* and the solid materials dissolved by condensation of CD_2Cl_2 *in vacuo* and assayed by ^1H, ^2H and $^{31}P\{^1H\}$ NMR spectroscopy at 298 K (Figure S70, Figure S71 and Figure S72). A mixture of **d_4-1[BAr$^F_4$]**, **2[BAr$^F_4$]** and **3[BAr$^F_4$]** was observed.

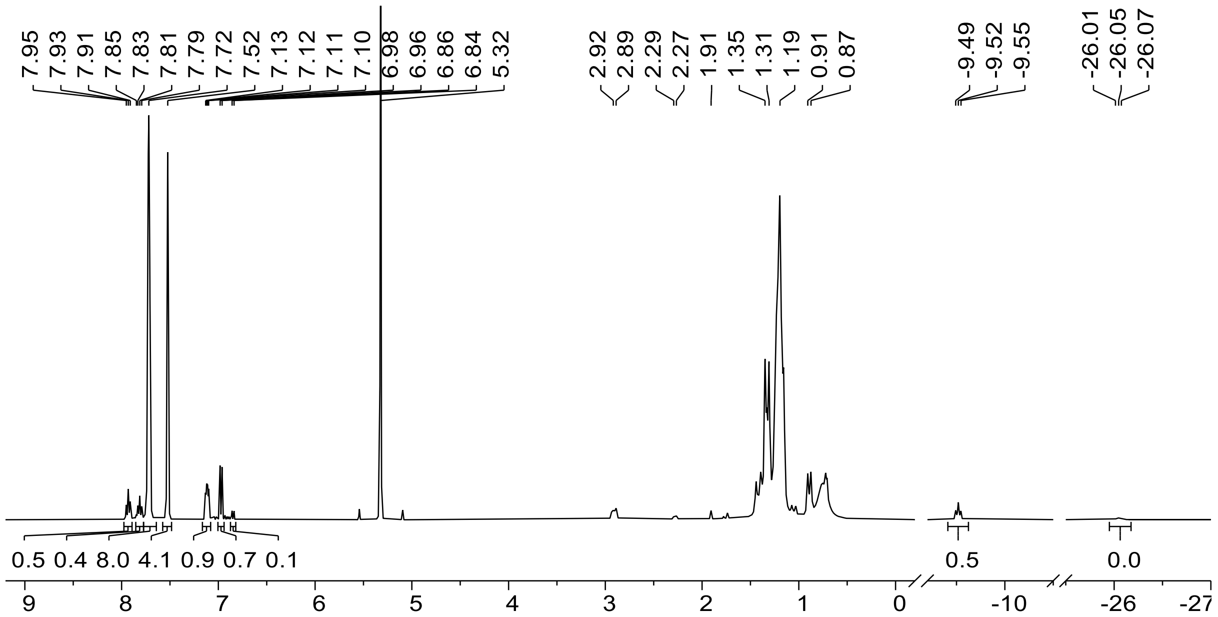

Figure S68. *^{1}H NMR spectrum of the products of the solid/gas reaction between [Ir(cyclo-^{t}Bu-PONOP')H][BAr$^{F}_{4}$]* ***2[BAr$^{F}_{4}$]*** *and CD_4 after 24 hours (400.11 MHz, CD_2Cl_2, 183 K).*

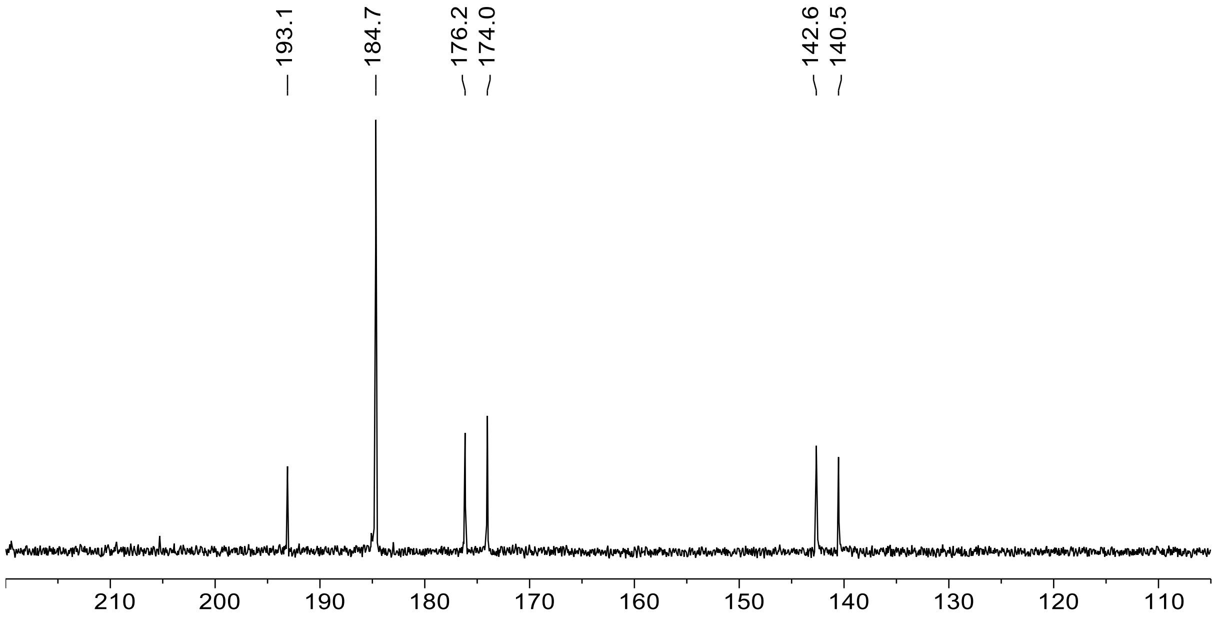

Figure S69. *^{31}P{^{1}H} NMR spectrum of the products of the solid/gas reaction between [Ir(cyclo-^{t}Bu-PONOP')H][BAr$^{F}_{4}$]* ***2[BAr$^{F}_{4}$]*** *and CD_4 after 24 hours (161.99 MHz, CD_2Cl_2, 183 K).*

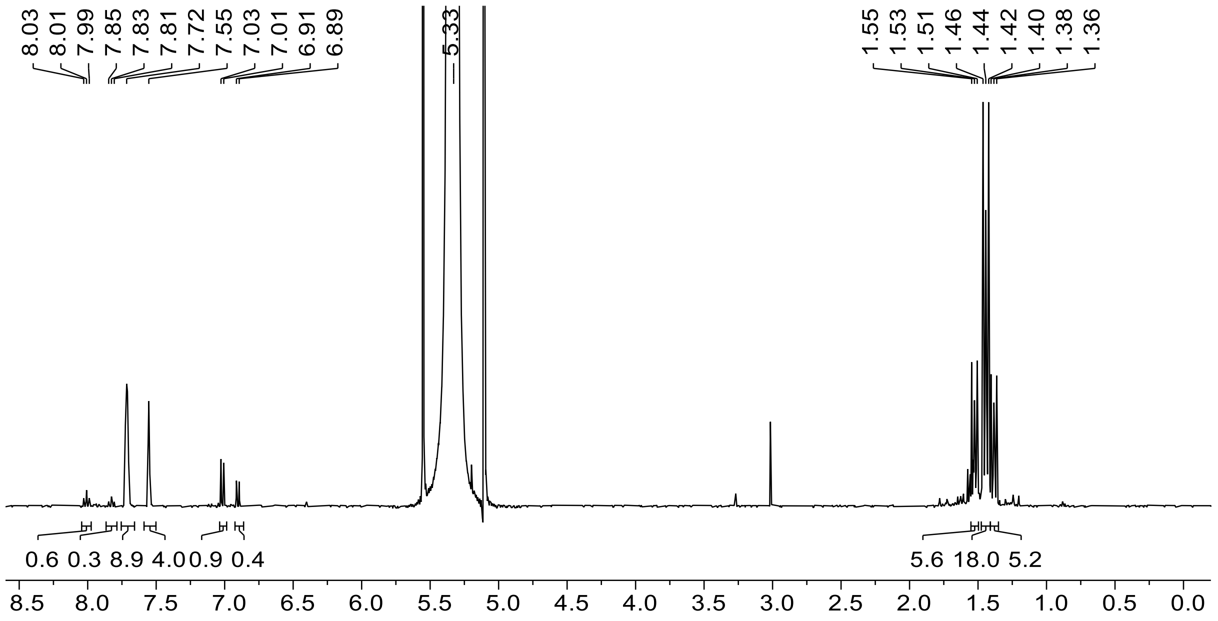

Figure S70. *^{1}H NMR spectrum of the products of the solid/gas reaction between [Ir(cyclo-[t]Bu-PONOP')H][BAr[F]4]* ***2[BAr[F]4]*** *and CD_4 after 24 hours and then treatment with carbon monoxide (400.11 MHz, CH_2Cl_2, 298 K).*

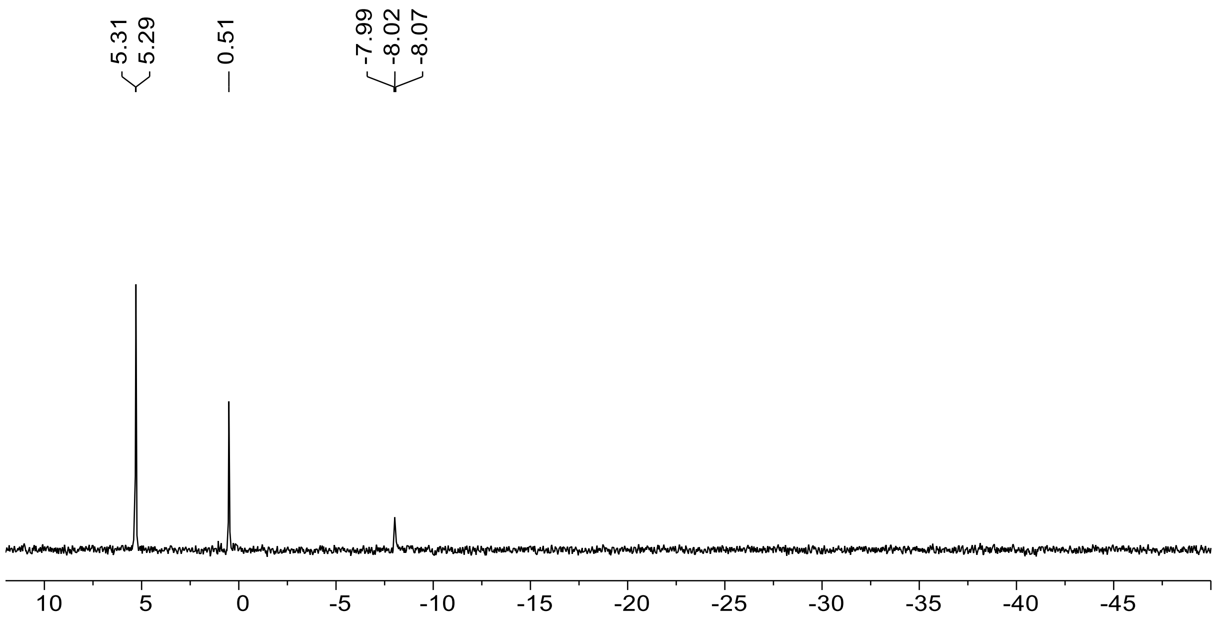

Figure S71. *^{2}H NMR spectrum of the products of the solid/gas reaction between [Ir(cyclo-[t]Bu-PONOP')H][BAr[F]4]* ***2[BAr[F]4]*** *and CD_4 after 24 hours and then treatment with carbon monoxide (61.42 MHz, CH_2Cl_2, 298 K).*

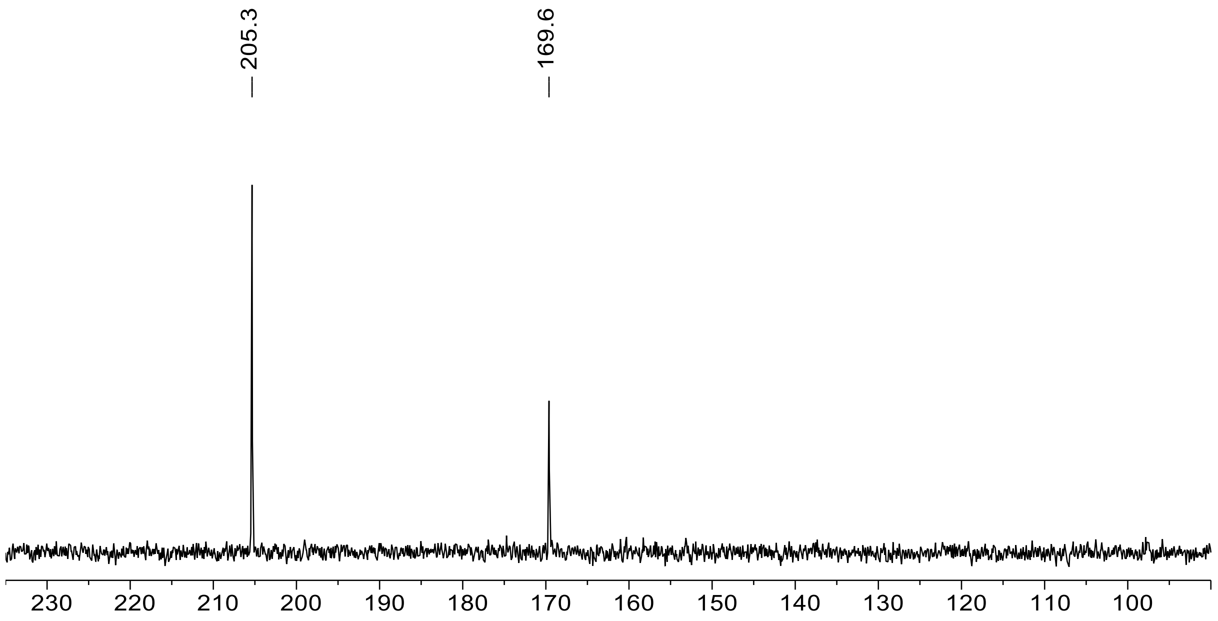

Figure S72. *$^{31}P\{^{1}H\}$ NMR spectrum of the products of the solid/gas reaction between [Ir(cyclo-^{t}Bu-PONOP')H][BAr$^{F}_{4}$]* ***2[BAr$^{F}_{4}$]*** *and CD_4 after 24 hours and then treatment with carbon monoxide (161.99 MHz, CH_2Cl_2, 298 K).*

Preparation of [Ir(cyclo-^{t}Bu-PONOP')H][BAr^F$_4$] from [Ir(^{t}Bu-PONOP)(CD$_3$)D][BAr^F$_4$]

A 5 mm thin-wall NMR tube fitted with high vacuum PTFE (J. Young) valve containing sieved single crystals (0.25-0.50 mm) of [Ir(^{t}Bu-PONOP)(CD$_3$)D][BAr^F$_4$] **α-1[BAr^F$_4$]** (15.0 mg, 10.2 μmol) was evacuated (<5 x 10^{-6} mbar) on a greaseless high-vacuum line for 24 hours before being heated at 80 °C for 24 hours. The tube was cooled to ambient temperature; the crystals were then dissolved by condensation of CD_2Cl_2 *in vacuo* and assayed by ^{1}H, ^{2}H and ^{31}P{^{1}H} NMR spectroscopy at 193 K. No ^{2}H containing species were observed other than the solvent.

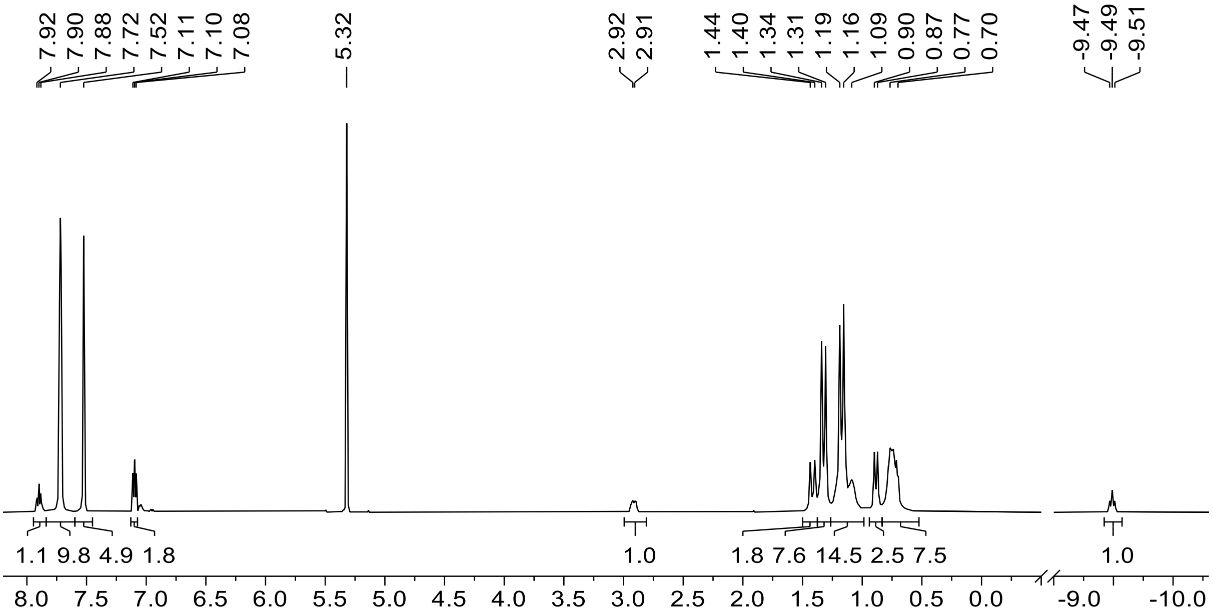

Figure S73. *^{1}H NMR spectrum of [Ir(cyclo-^{t}Bu-PONOP')H][BAr^F$_4$]* ***2[BAr^F$_4$]*** *obtained from the vacuum thermolysis of [Ir(^{t}Bu-PONOP)(CD$_3$)D][BAr^F$_4$]* ***α-1[BAr^F$_4$]*** *after 24 hours (500.22 MHz, CD_2Cl_2, 193 K).*

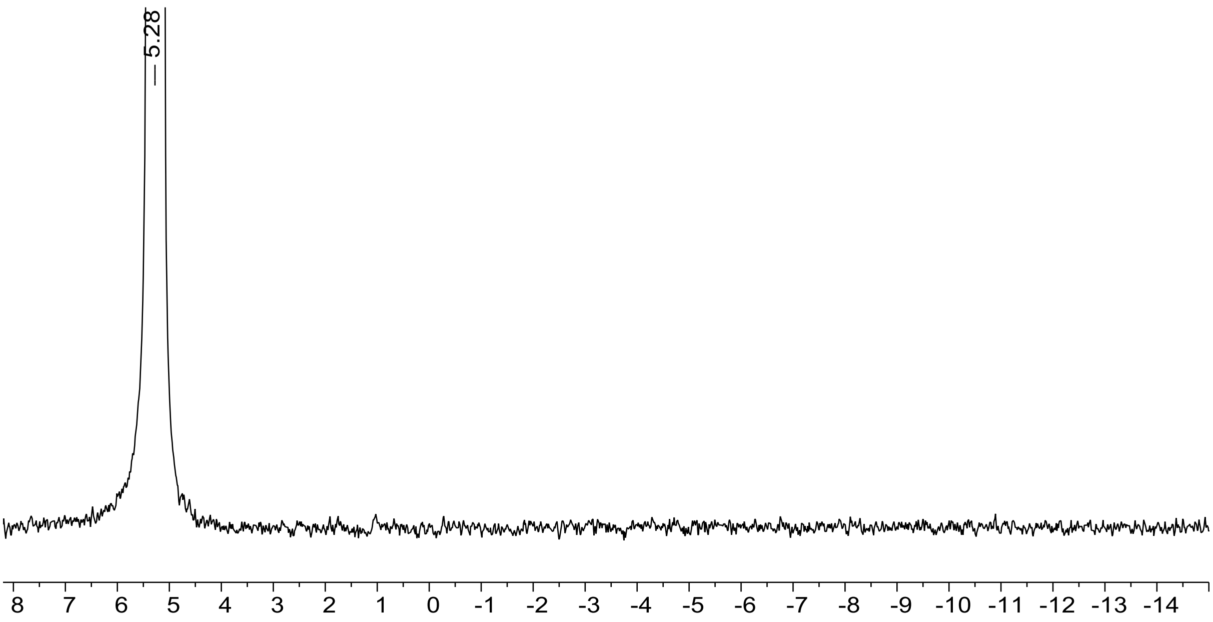

Figure S74. *^{2}H NMR spectrum of [Ir(cyclo-^{t}Bu-PONOP')H][BAr^F$_4$]* ***2[BAr^F$_4$]*** *obtained from the vacuum thermolysis of [Ir(^{t}Bu-PONOP)(CD$_3$)D][BAr^F$_4$]* ***α-1[BAr^F$_4$]*** *after 24 hours (76.79 MHz, CD_2Cl_2, 193 K).*

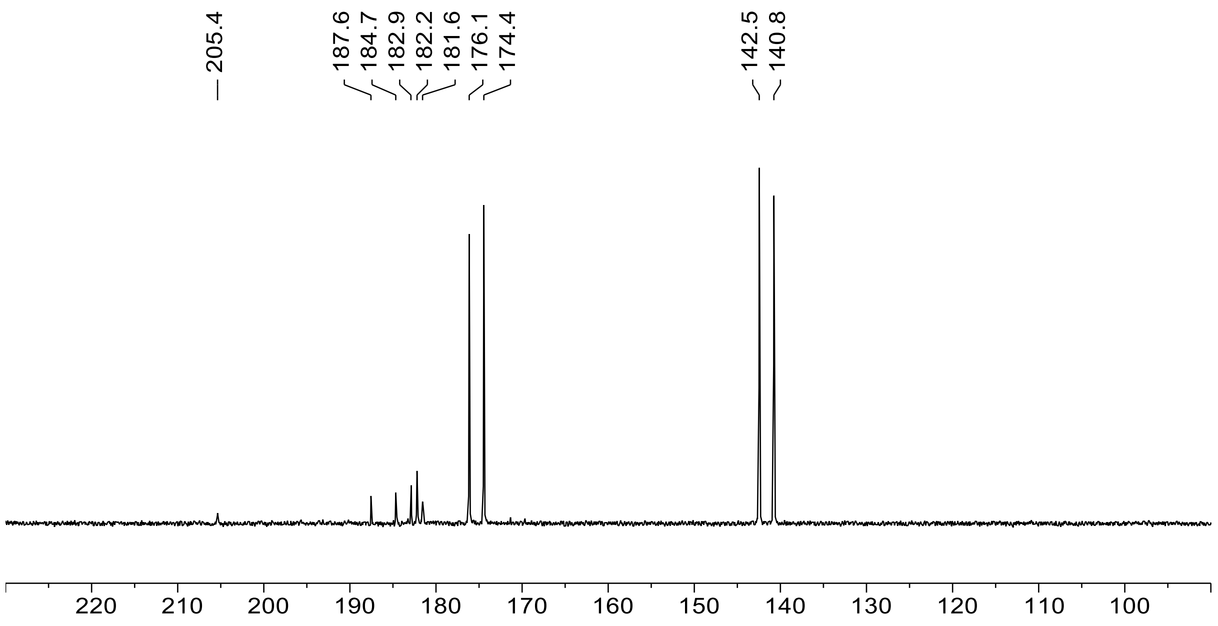

Figure S75*. $^{31}P\{^1H\}$ spectrum of [Ir(cyclo-^{t}Bu-PONOP')H][BAr$^F_4$]* ***2[BAr$^F_4$]*** *obtained from the vacuum thermolysis of [Ir(^{t}Bu-PONOP)(CD_3)D][BAr$^F_4$]* ***α-1[BAr$^F_4$]*** *after 24 hours (202.50 MHz, CD_2Cl_2, 193 K).*

Investigation of Equilibrium Isotope Effects through NMR Scale Vacuum Thermolysis

A 5 mm thin-wall NMR tube fitted with high vacuum PTFE (J. Young) valve containing sieved single crystals (0.25-0.50 mm) of [Ir(^{t}Bu-PONOP)MeH][BAr$^F_4$] **d$_4$-1[BAr$^F_4$]** (14.2 mg, 9.65 μmol) was evacuated (<5 x 10^{-6} mbar) on a greaseless high-vacuum line for 24 hours before being heated at 80 °C for 1 hour. The tube was cooled to ambient temperature; the crystals were then dissolved by condensation of CD_2Cl_2 *in vacuo* and assayed by ^{1}H and $^{31}P\{^1H\}$ NMR spectroscopy at 178 K (Figure S76 to Figure S78).

A separate 5 mm thin-wall NMR tube fitted with high vacuum PTFE (J. Young) valve containing sieved single crystals (0.25-0.5 mm) of [Ir(^{t}Bu-PONOP)(CD_3)D][BAr$^F_4$] **d$_4$-1[BAr$^F_4$]** (13.9 mg, 9.42 μmol) was evacuated (<5 x 10^{-6} mbar) on a greaseless high-vacuum line for 24 hours before being heated at 80 °C for 1 hour. The tube was cooled to ambient temperature; the crystals were then dissolved by condensation of CD_2Cl_2 *in vacuo* and assayed by ^{1}H and $^{31}P\{^1H\}$ NMR spectroscopy at 178 K (Figure S79 to Figure S81).

Solution NMR data were consistent with 85% conversion from **d$_4$-1[BAr$^F_4$]** to **2[BAr$^F_4$]** *vs*. 35% conversion of **α-1[BAr$^F_4$]** to **2[BAr$^F_4$]**.

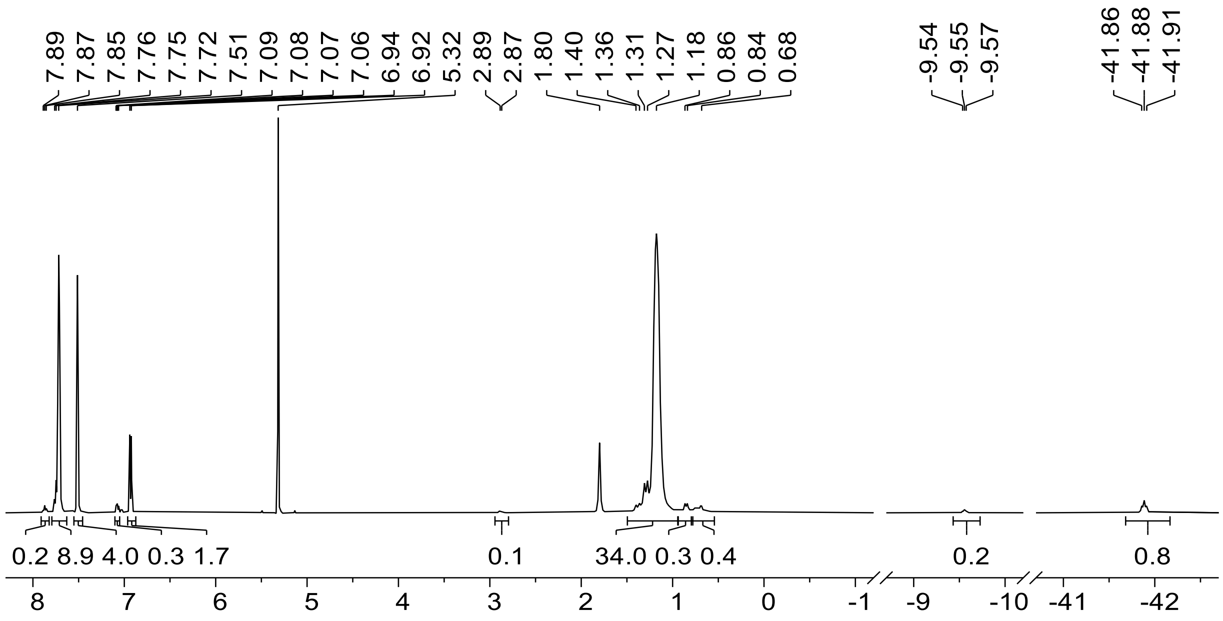

Figure S76. *^{1}H NMR spectrum of the products of the vacuum thermolysis reaction of [Ir(^{t}Bu-PONOP)MeH][BAr$^{F}_{4}$]* ***1[BAr$^{F}_{4}$]*** *after one hour (500.22 MHz, CD_2Cl_2, 178 K).*

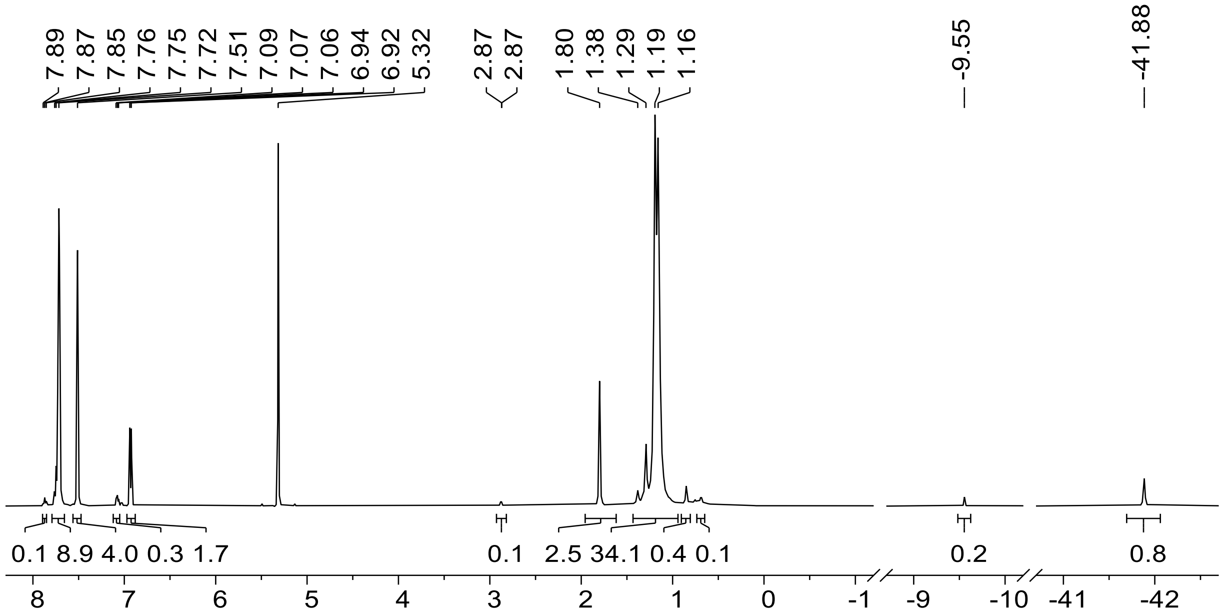

Figure S77. *^{1}H{^{31}P} NMR spectrum of the products of the vacuum thermolysis reaction of [Ir(^{t}Bu-PONOP)MeH][BAr$^{F}_{4}$]* ***1[BAr$^{F}_{4}$]*** *after one hour (500.22 MHz, CD_2Cl_2, 178 K).*

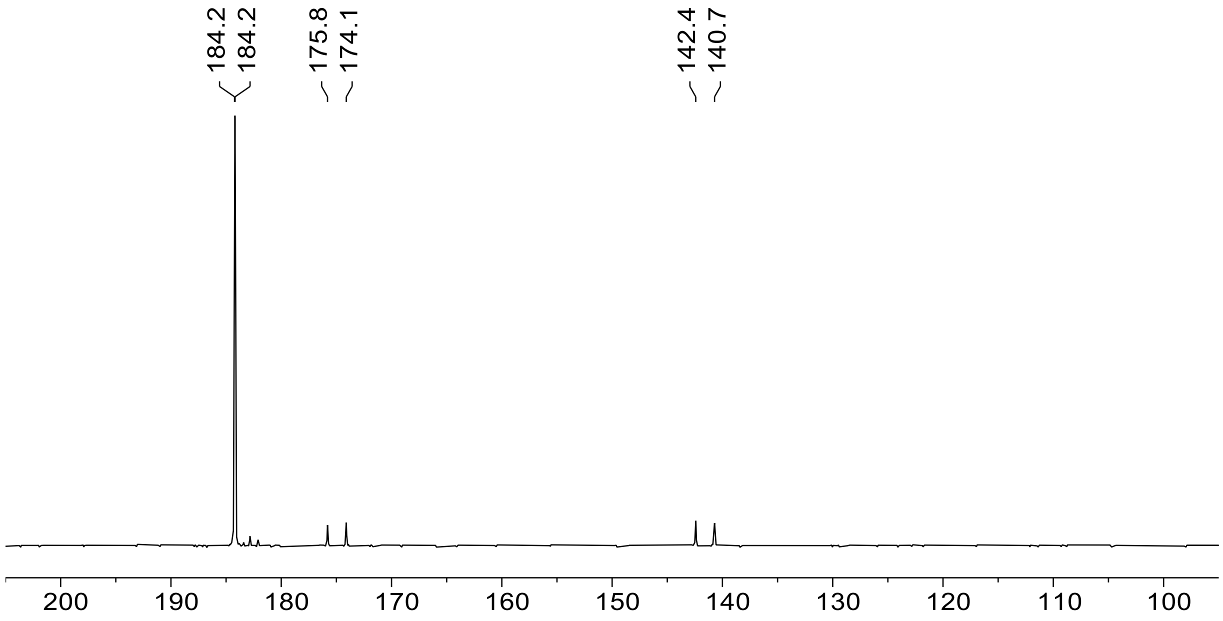

Figure S78. *$^{31}P\{^{1}H\}$ NMR spectrum of the products of the vacuum thermolysis reaction of [Ir(^{t}Bu-PONOP)MeH][BAr$^F{}_4$]* ***1[BAr$^F{}_4$]*** *after one hour (202.50 MHz, CD_2Cl_2, 178 K).*

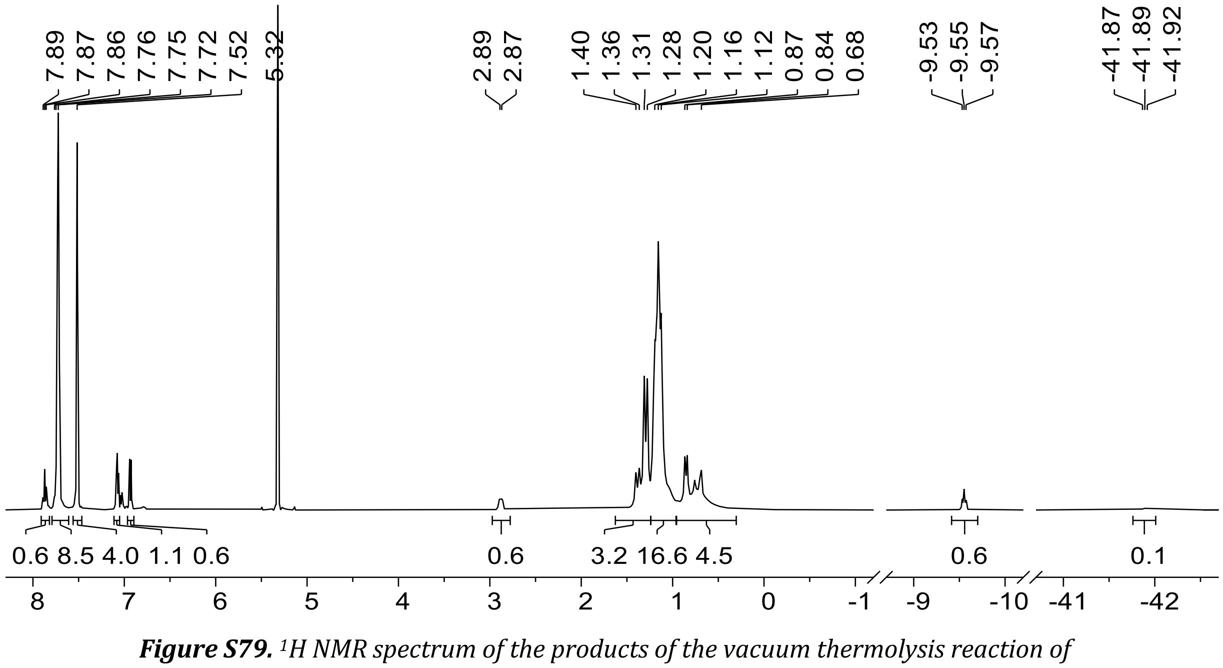

Figure S79. *^{1}H NMR spectrum of the products of the vacuum thermolysis reaction of [Ir(^{t}Bu-PONOP)(CD_3)D][BAr$^F{}_4$]* ***d_4-1[BAr$^F{}_4$]*** *after one hour (500.22 MHz, CD_2Cl_2, 178 K).*

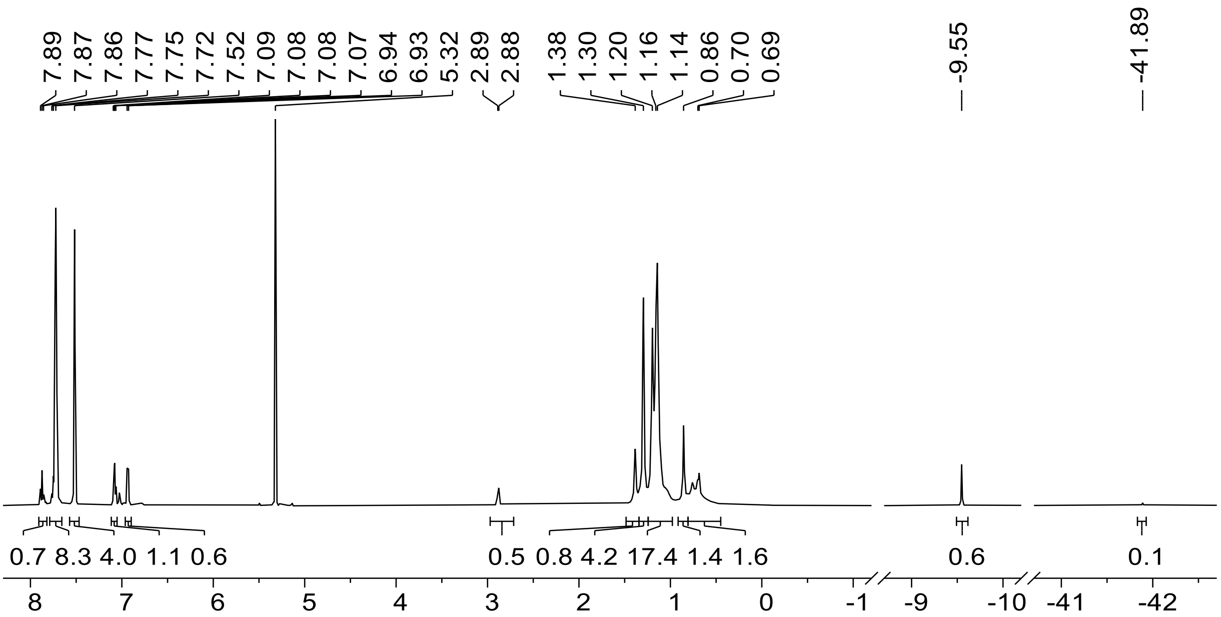

Figure S80. *$^{1}H\{^{31}P\}$ NMR spectrum of the products of the vacuum thermolysis reaction of $[Ir(^{t}Bu\text{-}PONOP)(CD_3)D][BAr^F{}_4]$* ***$d_4$-1[BAr$^F{}_4$]*** *after one hour (500.22 MHz, CD_2Cl_2, 178 K).*

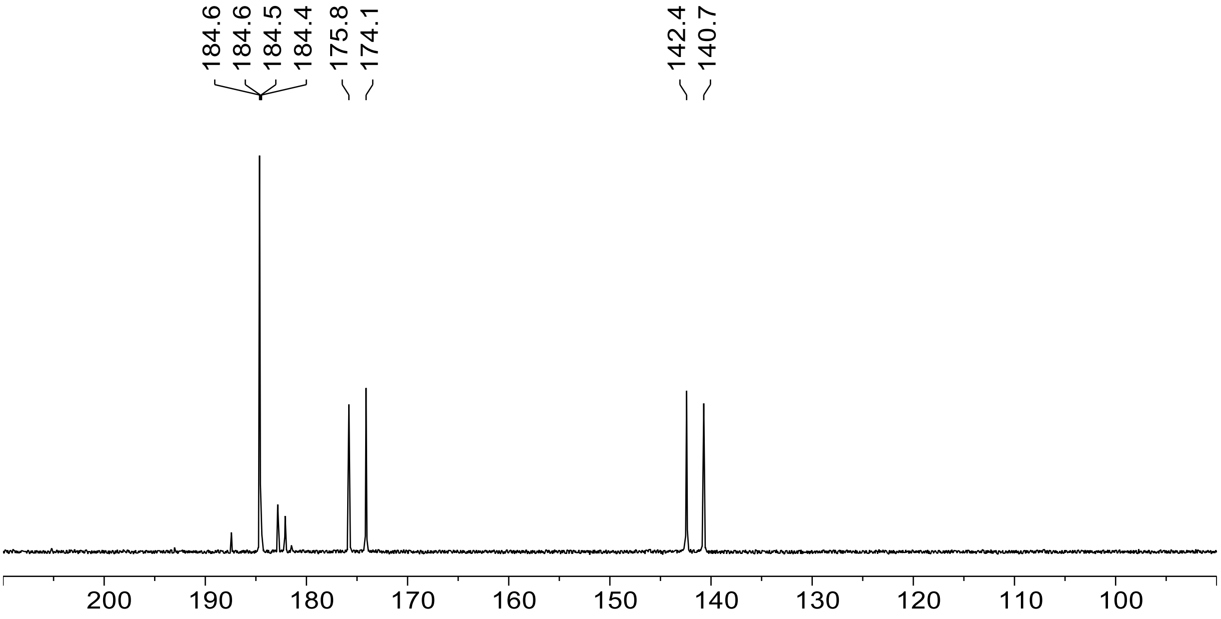

Figure S81. *$^{31}P\{^{1}H\}$ NMR spectrum of the products of the vacuum thermolysis reaction of $[Ir(^{t}Bu\text{-}PONOP)(CD_3)D][BAr^F{}_4]$* ***$d_4$-1[BAr$^F{}_4$]*** *after one hour (202.50 MHz, CD_2Cl_2, 178 K).*

Attempted Solid/Gas CH_4/CD_4 Exchange at Room Temperature

A 5 mm heavy-wall NMR tube containing single crystals of $[Ir(^{t}Bu\text{-}PONOP)MeH][BAr^F{}_4]$ **1[BAr$^F{}_4$]** (9.5 mg, 6.5 μmol) was evacuated and pressurised with CD_4 (10 bar gauge). The tube was sealed and stored at ambient temperature for 48 hours, depressurised and remaining gases removed *in vacuo*. The crystals were dissolved by condensation of CD_2Cl_2 *in vacuo* and assayed by ^{1}H, ^{2}H and $^{31}P\{^{1}H\}$ NMR spectroscopy at 193 K. Spectroscopic data were consistent with no exchange of CH_4 for CD_4 under these conditions.

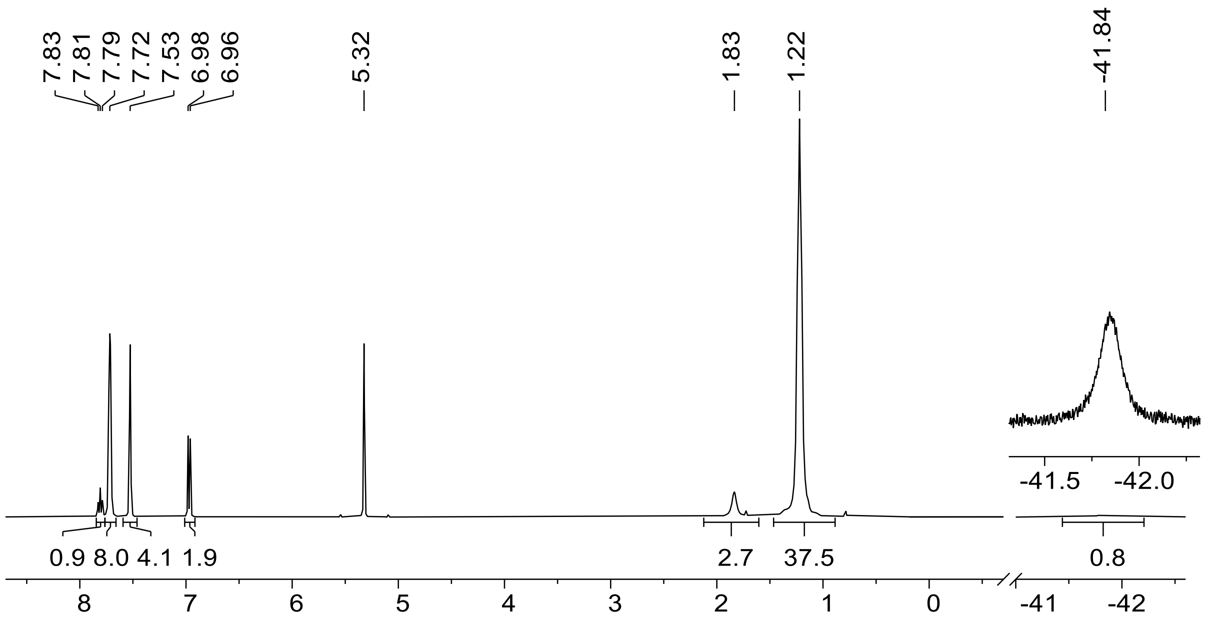

Figure S82. *^{1}H NMR spectrum of the product of the attempted solid/gas reaction between [Ir(^{t}Bu-PONOP)MeH][BAr$^F{}_4$]* ***1[BAr$^F{}_4$]*** *and CD_4 (400.11 MHz, CD_2Cl_2, 193 K).*

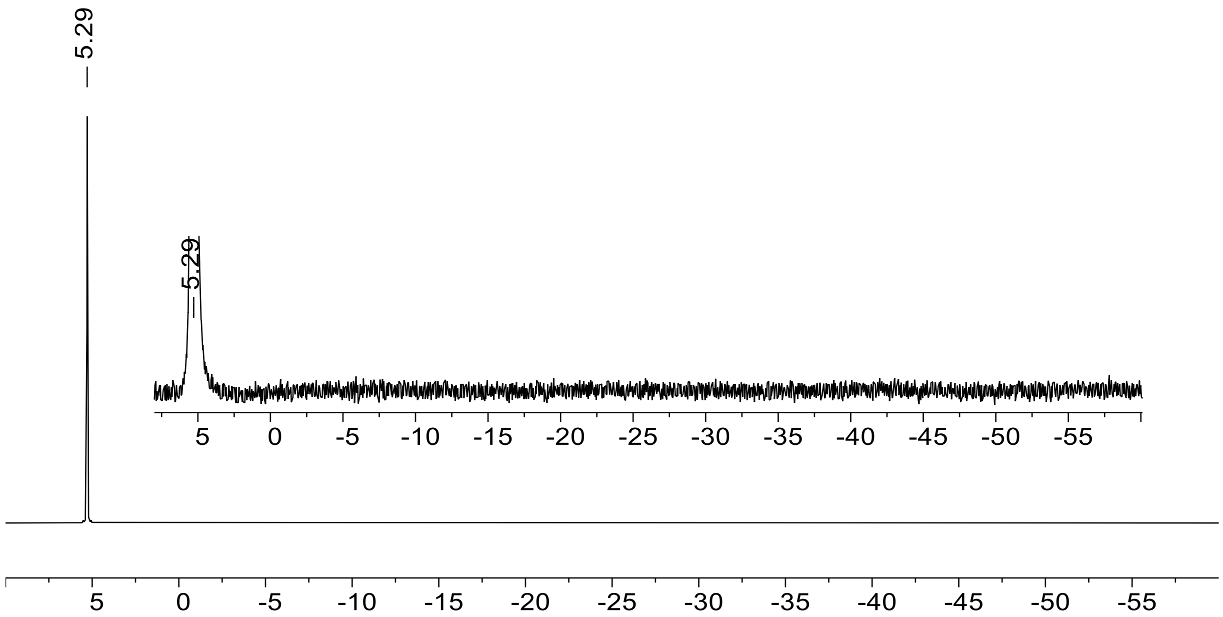

Figure S83. *^{2}H NMR spectrum of the product of the attempted solid/gas reaction between [Ir(^{t}Bu-PONOP)MeH][BAr$^F{}_4$]* ***1[BAr$^F{}_4$]*** *and CD_4 (61.42 MHz, CD_2Cl_2, 203 K).*

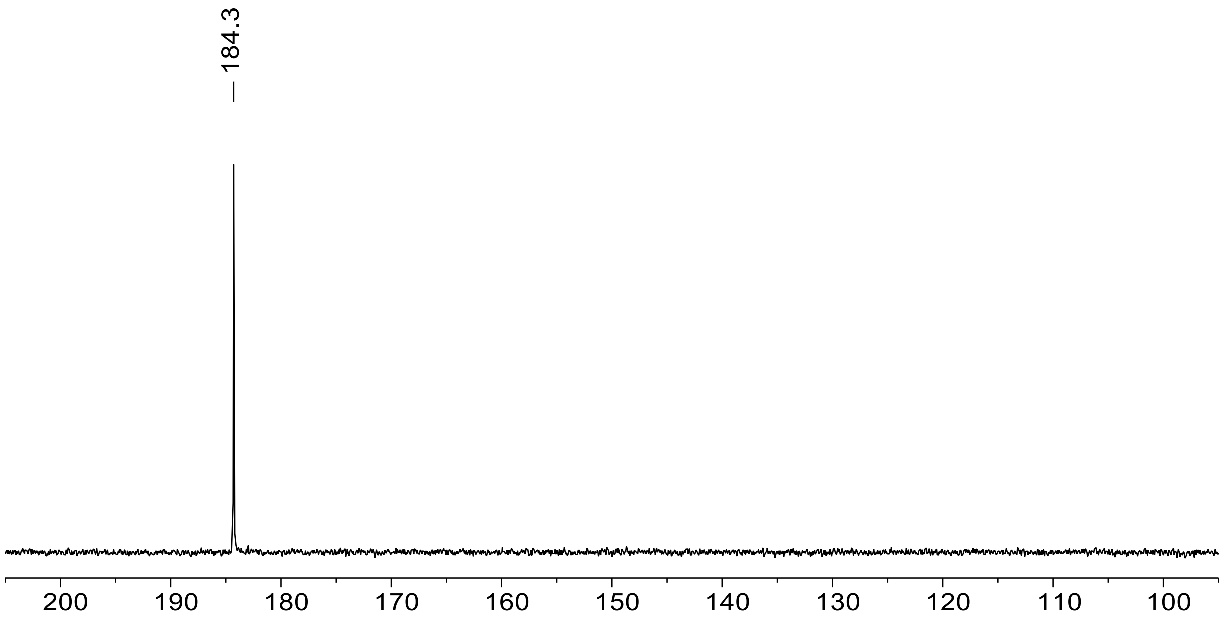

Figure S84. *$^{31}P\{^1H\}$ NMR spectrum of the product of the attempted solid/gas reaction between [Ir(^{t}Bu-PONOP)MeH][BAr$^F_4$]* ***1[BAr$^F_4$]*** *and CD_4 (162.00 MHz, CD_2Cl_2, 203 K).*

Solid/Gas Reaction of [Ir(cyclo-^{t}Bu-PONOP')H][BAr$^F_4$] with C_2H_6

A 5 mm thin-wall valved NMR tube containing single crystals of **2[BAr$^F_4$]** (16.0 mg, 11.0 µmol) was evacuated, re-pressurised with ethane (1 bar gauge) and the contents freeze-thaw degassed on the high vacuum line before being heated at 80 °C for 3 weeks. The tube was cooled to room temperature, de-pressurised and the contents were assayed by $^{13}C\{^1H\}$ and $^{31}P\{^1H\}$ SS NMR under argon and then dissolved by condensation of CD_2Cl_2 *in vacuo* to record ^1H and $^{31}P\{^1H\}$ NMR spectroscopy at 298 K. Both sets of data are consistent with the formation of [Ir(^{t}Bu-PONOP)(η^2-H_2C=CH_2)][BAr$^F_4$] **5[BAr$^F_4$]** and [Ir(^{t}Bu-PONOP)H_2][BAr$^F_4$] **6[BAr$^F_4$]** as the two major products in approximately equal proportions.

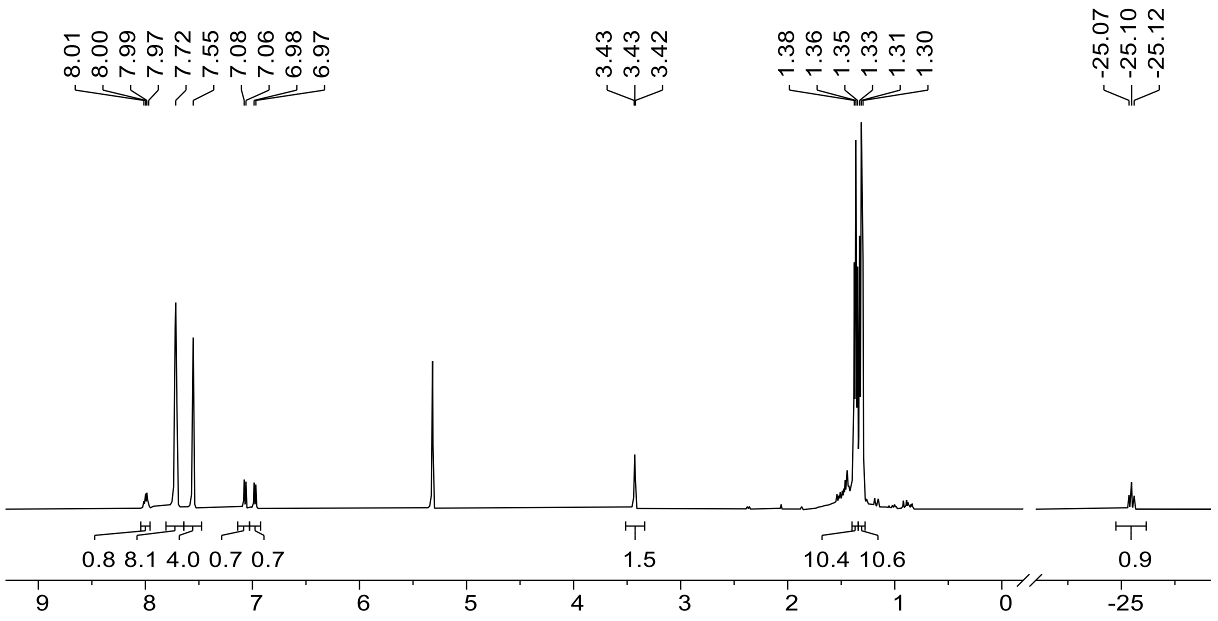

Figure S85. *^{1}H NMR spectrum of the products of the solid/gas reaction between [Ir(cyclo-[t]Bu-PONOP')H][BAr[F]4]* ***2[BAr[F]4]*** *and C_2H_6 after 3 weeks (500.22 MHz, CD_2Cl_2, 298 K).*

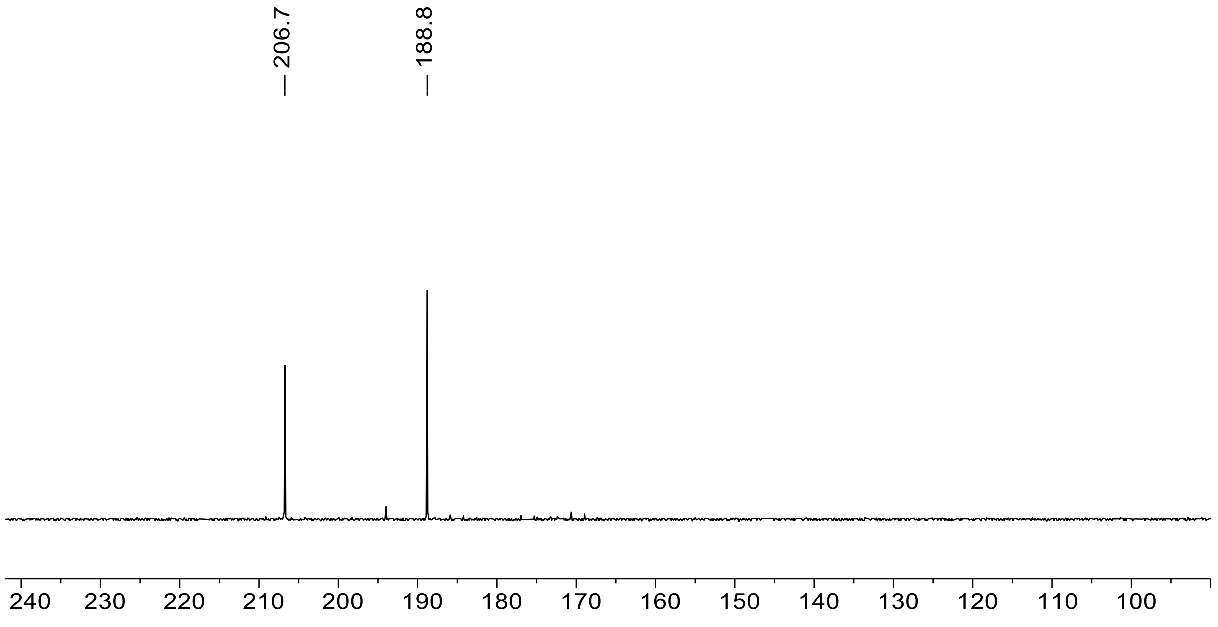

Figure S86. *^{31}P{^{1}H} NMR spectrum of the products of the solid/gas reaction between [Ir(cyclo-[t]Bu-PONOP')H][BAr[F]4]* ***2[BAr[F]4]*** *and C_2H_6 after 3 weeks (202.50 MHz, CD_2Cl_2, 298 K).*

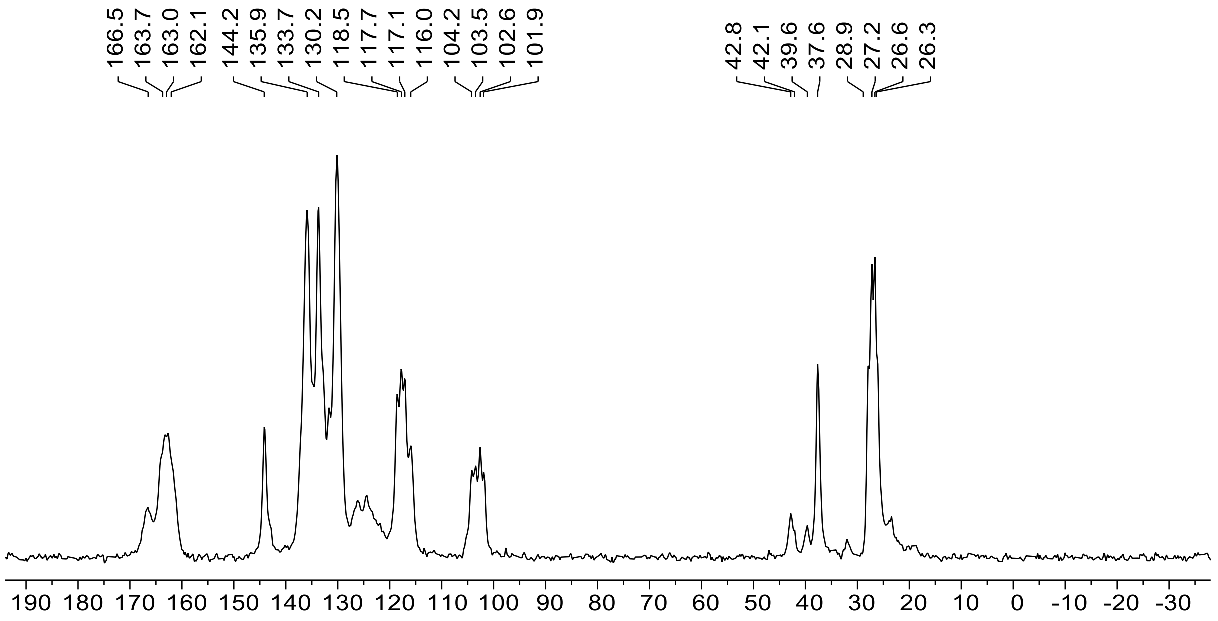

Figure S87. *$^{13}C\{^1H\}$ CP MAS NMR spectrum of the products of the solid/gas reaction between [Ir(cyclo-^{t}Bu-PONOP')H][BAr$^F_4$]* ***2[BAr$^F_4$]*** *and C_2H_6 after 3 weeks (20 kHz spin rate, 100.66 MHz, 298 K).*

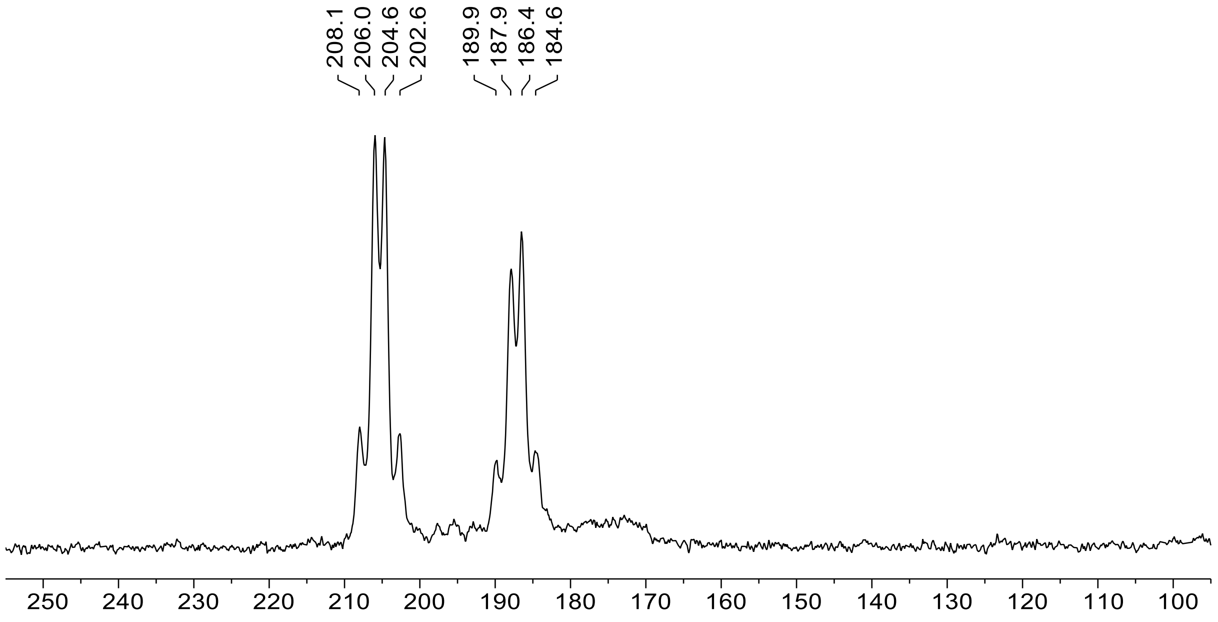

Figure S88. *$^{31}P\{^1H\}$ CP MAS NMR spectrum of the products of the solid/gas reaction between [Ir(cyclo-^{t}Bu-PONOP')H][BAr$^F_4$]* ***2[BAr$^F_4$]*** *and C_2H_6 after 3 weeks (20 kHz spin rate, 162.06 MHz, 298 K)*

Single Crystal Diffraction

X-ray Diffraction

Single-crystal X-ray diffraction data were collected (ω-scans) on a Rigaku SuperNova diffractometer with Cu-K_α (λ = 1.54184 Å) or Mo-K_α (λ = 0.71073 Å) radiation equipped with a dinitrogen gas Oxford Cryosystems Cryostream unit. Diffraction images from raw frame data were reduced using CrysAlis Pro. The structures were solved using SHELXT[15] and refined to convergence on F^2 and against all independent reflections by full-matrix least-squares using SHELXL[13] through the Olex2 GUI.[14] All non-hydrogen atoms were refined anisotropically; hydrogen atoms were geometrically placed and allowed to ride on their parent atoms. Distances and angles were calculated using the full covariance matrix. Disordered CF_3 groups on the [BAr^F_4] anions in all structures were and modelled over two domains with occupancies freely refined using a SHELX free variable and restrained (*e.g*. SADI, SIMU, RIGU) to maintain chemically sensible geometries and anisotropic displacement parameters. No hydride ligands were located against the residual electron density difference maps for any structures determined by single-crystal X-ray diffraction, moiety formulae in the respective *.cifs incorporate these additional hydride ligands. Crystals of **β-1[BAr^F_4]** were invariably found to have a very minor twinned component (BASF = 0.00147, twin law -1 0 1 0 -1 0 0 0 1 2). Disordered *tert*-butyl groups and methylene groups of **2[BAr^F_4]** were modelled over two sites through SHELX free variables and restrained to chemically sensible geometries (*e.g*. SADI, SIMU, RIGU). Crystals of **3[BAr^F_4]** possess two larger than expected residual diffraction peaks around the heavy iridium atom (Ir(1)-Q(1) = 1.2273(4) Å; Ir(1)-Q(2) = 1.2433(4) Å), which lead to a CHECKCIF A alert. An alternative cation site (P-Ir-P, *vide infra*) was considered however the refinement was not chemically sensible based upon comparison to Ir-P bond lengths observed in the main structure component. It was observed that the Q(1)-Ir(1)-Q(2) vector is parallel to the long *c*-axis (39.6346(5) Å) of the cell and thus these residual peaks are believed to be a consequence of uncompensated absorption from the heavy iridium atom in tandem with the expected ripples from Fourier series termination errors. Attempts to apply various different techniques of absorption correction were not successful nor was this compensated for by diffraction with alternative wavelengths (Mo-K_α λ = 0.71073 Å) and thus the residual peaks were left in the structure. A very low-occupancy cation site (SOF ~0.04) is observed in the structure of **5 + 6[BAr^F_4]** for which only Ir(1B), P(1B) and P(2B) could be satisfactorily modelled. This yields a marked improvement in refinement statistics however attempts to model the expected carbon atom sites were non-productive.

Neutron Diffraction

The single-crystal neutron study of **α-1[$BArF_4$]** was the first user experiment undertaken from the first proposal round (2023-2) for which the newly constructed KOALA-2 instrument was available. The new instrument contains significant improvements to the instrument operation, but largely follows the design principles of the KOALA diffractometer. [15] The newly commissioned COBRA wide-bore (9mm diameter inner cold stream) N_2 device built for ANSTO by Oxford Cryosystems delivered excellent temperature control at 110 K with no ice formation about the crystal despite its relatively large dimensions (1.0 × 1.2 × 1.8 mm). The new instrument stands at the same end of guide position on TG-3, a thermal neutron supermirror guide in the neutron guide hall at the OPAL nuclear reactor at ANSTO, Lucas Heights, NSW

Australia. Data were collected in the Laue mode - with the new detector covering a full 360 degrees about the instrument phi axis by means of a neutron sensitive image plate. A total of 17 images across two mountings of the monoclinic crystal relative to the phi axis were measured for 120 minutes neutron beam exposure time with an interframe rotation of 17 degrees within each crystal setting.

A typical image is shown below showing how the program LaueG can be employed to index each frame (using the X-ray determined cell at the same temperature) as well as an expansion of the indicated region shows how indexed spots are treated to facilitate extraction of data for normalization and use in refinement. [16, 17] 141417 data in the wavelength range 0.85 ≤ λ ≤ 1.7 angstrom were extracted R_{int} = 8.2 (6.5)% wR_{int2} =13.2 (9.0)% GOOF = 1.5 (24198 data > 4 sig) of which 13889 were unique. R_{int} =20.9 (19.8)% wR_{int2} =22.8 (21.0)% GOOF = 1.1 (for all data). The structure from the X-ray study was refined against the neutron diffraction data with all H atom sites clearly defined on difference maps using CRYSTALS.[18] A refinement on *F* of all atoms modelled with anisotropic displacement parameters converged to *R* = 0.089, R_w = 0.037 for 1261 parameters, 6609 data I ≥ 3σI and 522 restraints (counterion geometry) with application of a suitable weighting scheme. Final residual densities of -1.69 and 2.2 lie at background levels and are inconsistent with any unmodelled atomic sites.

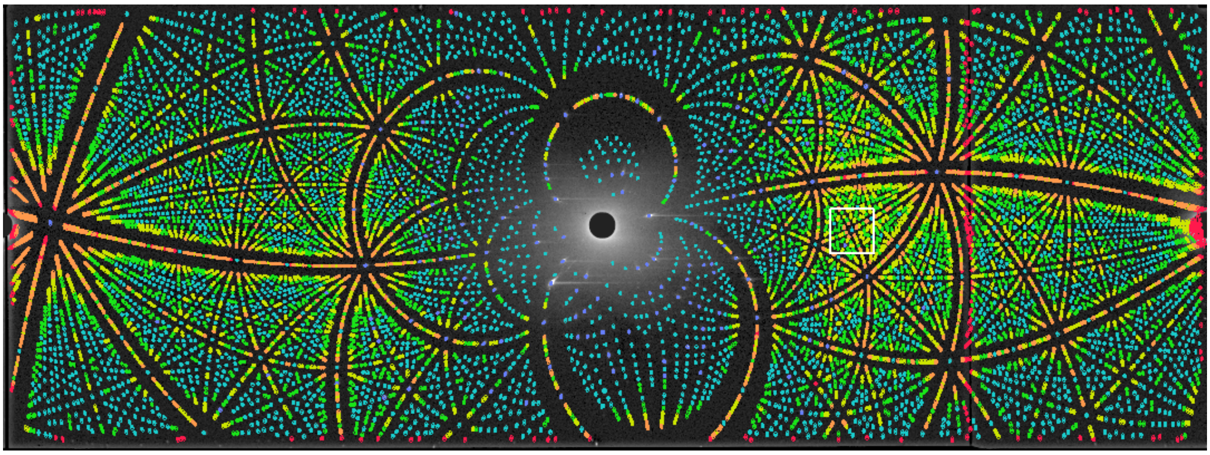

Figure S89. *A selected neutron diffraction image displayed in LaueG.*

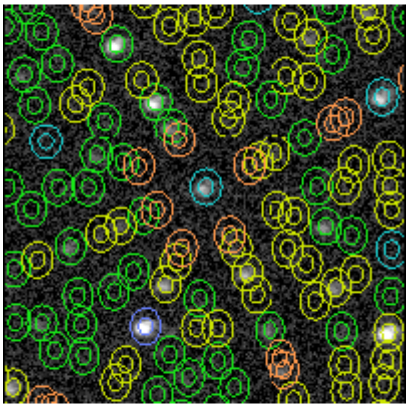

Figure S90. *An expansion of the region indicated in Figure S89.*

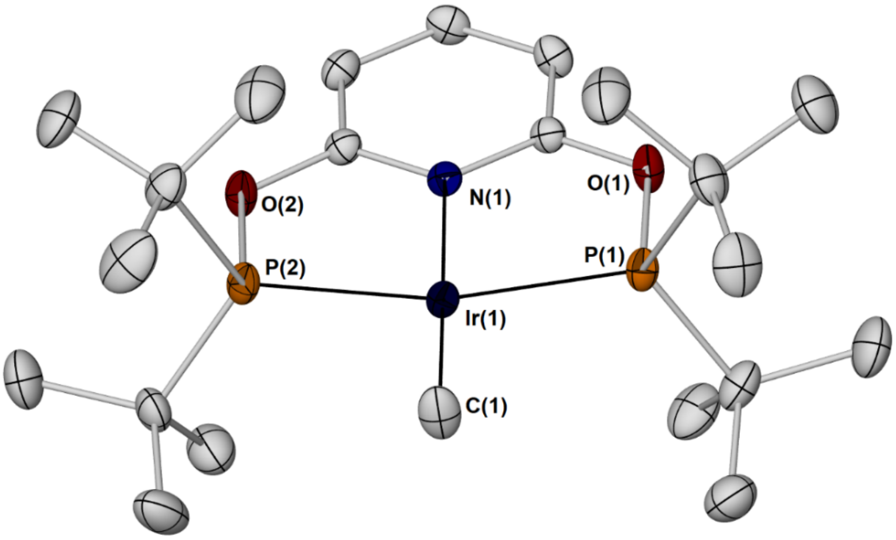

Figure S91. *The cation of the molecular structure of* ***α-1[BAr^F$_4$]*** *as determined by single-crystal X-ray diffraction. Anisotropic displacement parameters are represented as 50% probability ellipsoids. All hydrogen atoms omitted for clarity. Selected bond lengths (Å): Ir(1)-C(1) 2.097(3), Ir(1)-N(1) 2.093(3), Ir(1)-P(1) 2.2781(8), Ir(1)-P(2) 2.2824(8).*

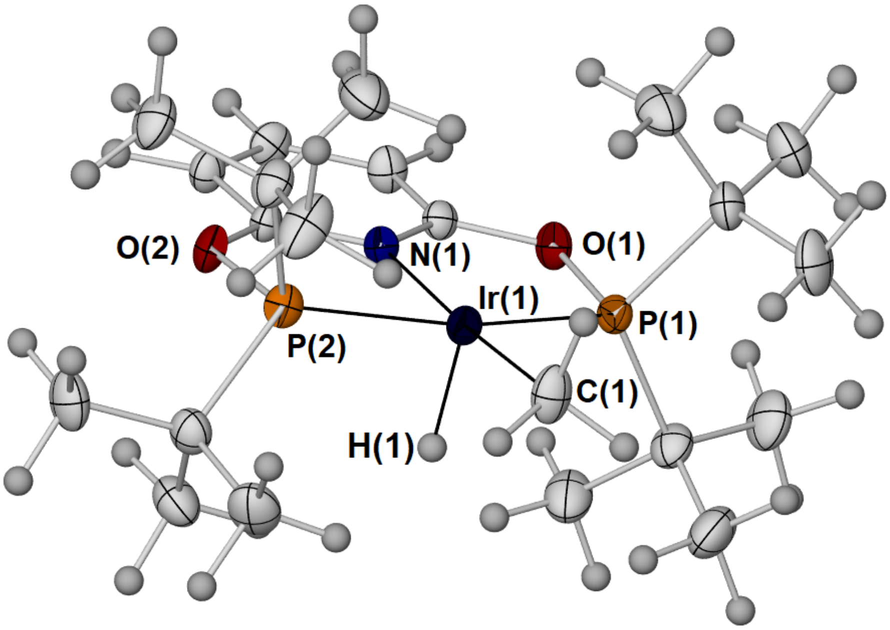

Figure S92. *The cation of the molecular structure of* ***α-1[BAr^F$_4$]*** *as determined by single-crystal neutron diffraction. Anisotropic displacement parameters are represented as 50% probability ellipsoids. All hydrogen atoms are represented as spheres of 20% of van der Waals radius for clarity. Selected bond lengths (Å): Ir(1)-C(1) 2.096(3), Ir(1)-H(1) 1.529(6), Ir(1)-N(1) 2.085(2), Ir(1)-P(1) 2.269(3), Ir(1)-P(2) 2.274(3).*

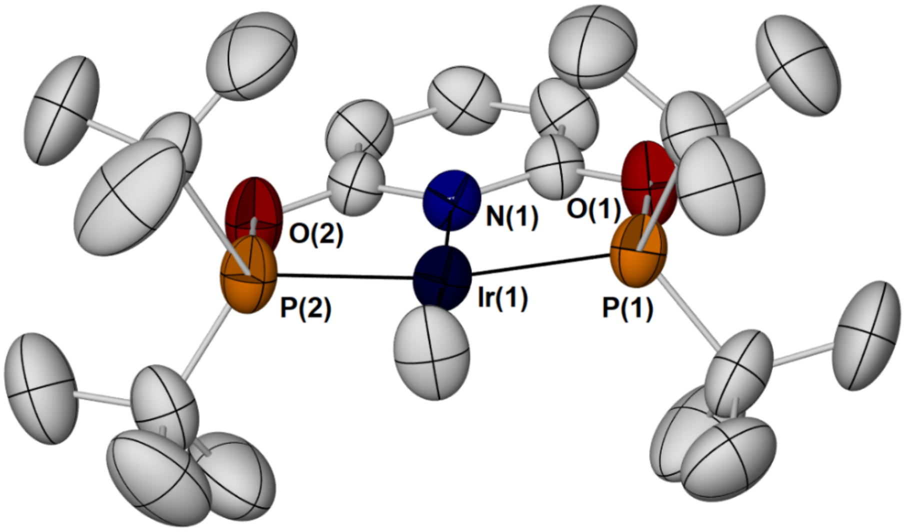

Figure S93. *The cation of the molecular structure of **α-1[BAr$^F_4$]** as determined by single-crystal X-ray diffraction at ambient temperature. Anisotropic displacement parameters are represented as 50% probability ellipsoids. All hydrogen atoms omitted for clarity. Selected bond lengths (Å): Ir(1)-C(1) 2.116(3), Ir(1)-N(1) 2.087(2), Ir(1)-P(1) 2.2823(8), Ir(1)-P(2) 2.2880(9).*

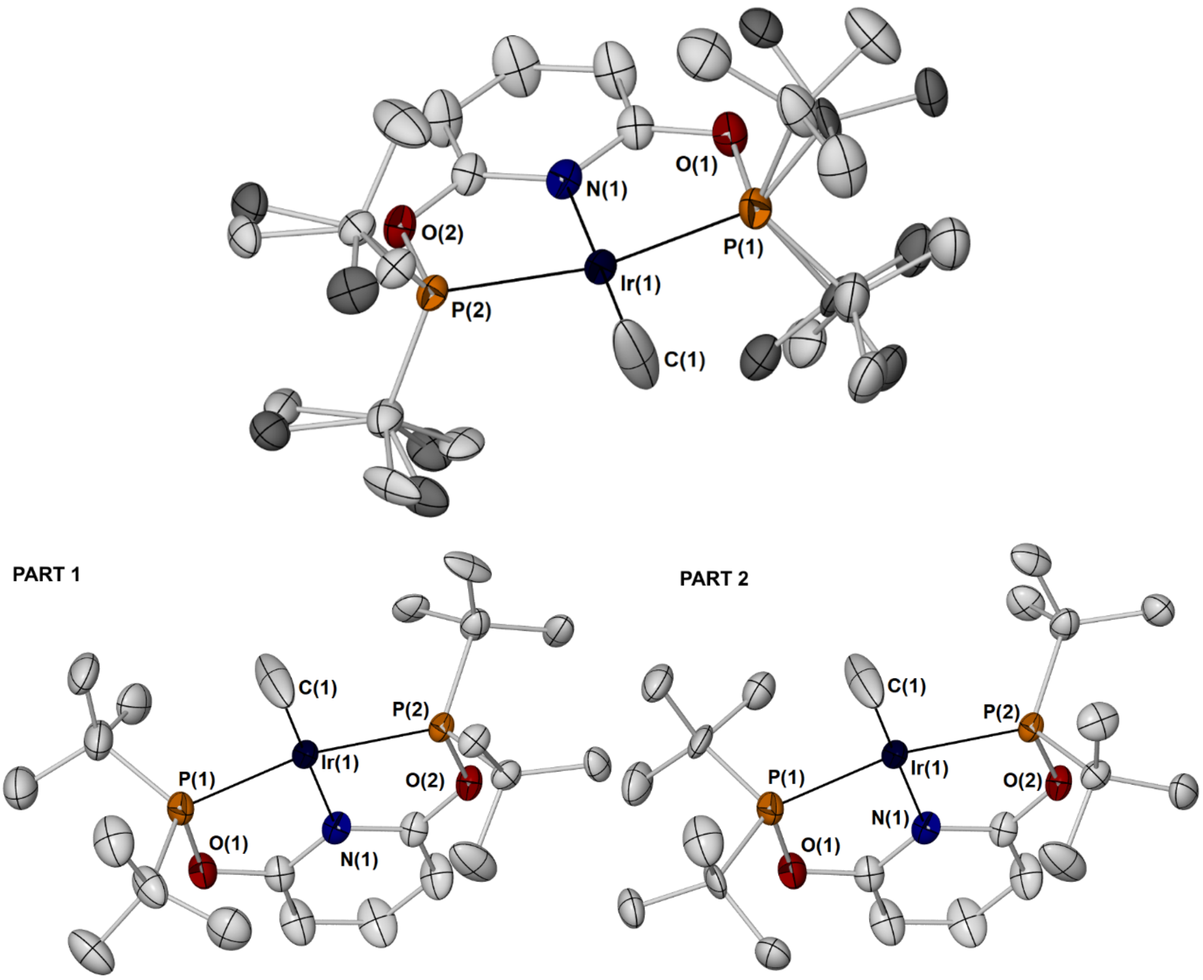

Figure S94. *The cation of the molecular structure of **β-1[BAr$^F_4$]** as determined by single-crystal X-ray diffraction. PART 1 and PART 2 of the complete model are displayed separately. Anisotropic displacement parameters are represented as 50% probability ellipsoids. All hydrogen atoms omitted for clarity. Selected bond lengths (Å): Ir(1)-C(1) 2.088(5), Ir(1)-N(1) 2.094(3), Ir(1)-P(1) 2.2743(8), Ir(1)-P(2) 2.2765(8).*

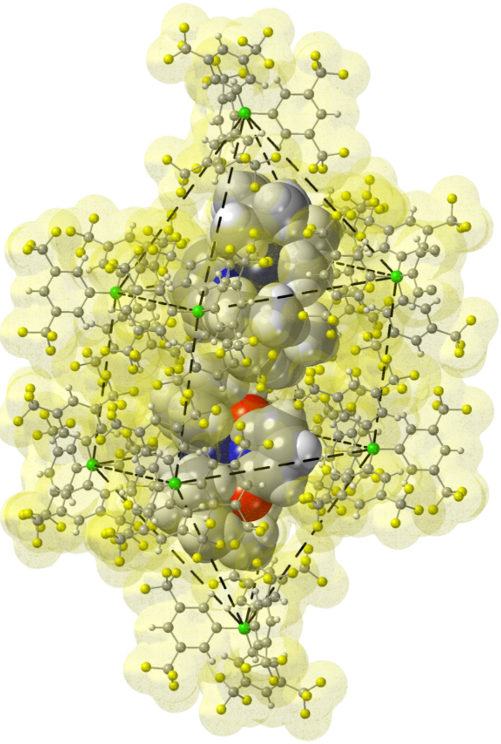

***Figure S95**. Packing arrangement of [BAr$^{F}_{4}$]$^{-}$ anions in **β-1[BAr$^{F}_{4}$]**. The cations and anion surface are shown as van der Waals radii.*

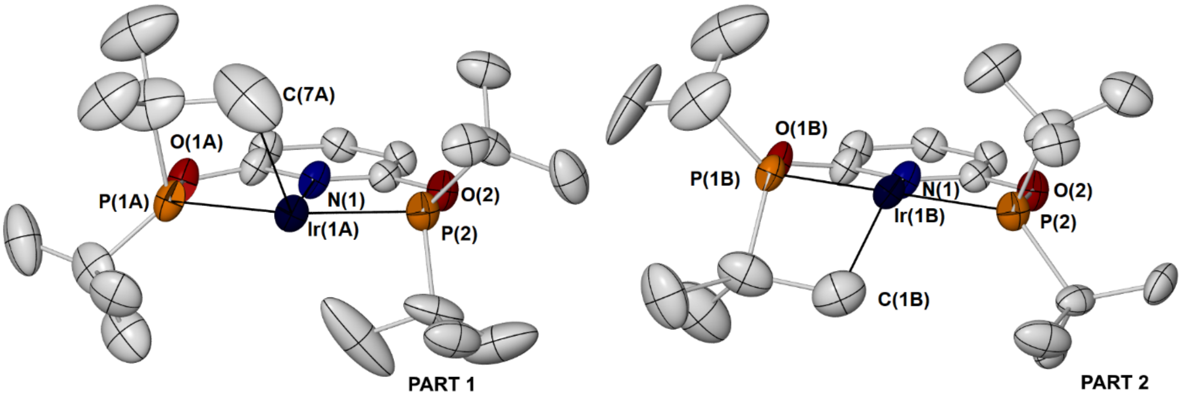

***Figure S96.** The cation of the molecular structure of **2[BAr$^{F}_{4}$]** as determined by single-crystal X-ray diffraction viewed down the* h = *-14,* k = *17,* l = *10 vector. PART 1 and PART 2 of the complete model are displayed separately. Anisotropic displacement parameters are represented as 50% probability ellipsoids. All hydrogen atoms omitted for clarity. Selected bond lengths (Å): Ir(1B)-C(1B) 2.038(15), Ir(1A)-C(7A) 2.176(19), Ir(1A)-N(1) 2.124(3), Ir(1B)-N(1) 2.074(3), Ir(1A)-P(1A) 2.276(4), Ir(1B)-P(1B) 2.204(4), Ir(1A)-P(2) 2.3095(16), Ir(1B)-P(2) 2.2106(15).*

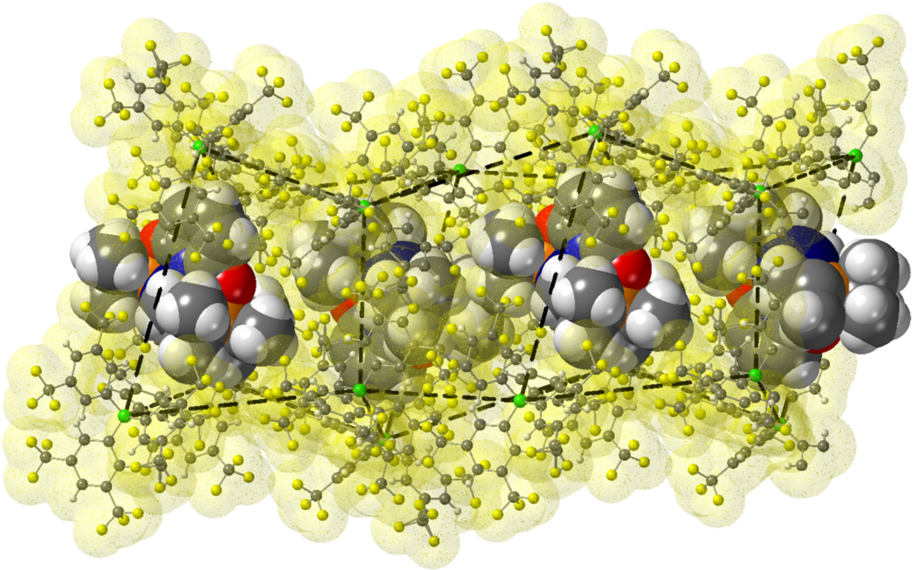

***Figure S97**. Packing arrangement of [BAr^F$_4$]$^-$ anions in **2[BAr^F$_4$]**. The cations and anion surface are shown as van der Waals radii.*

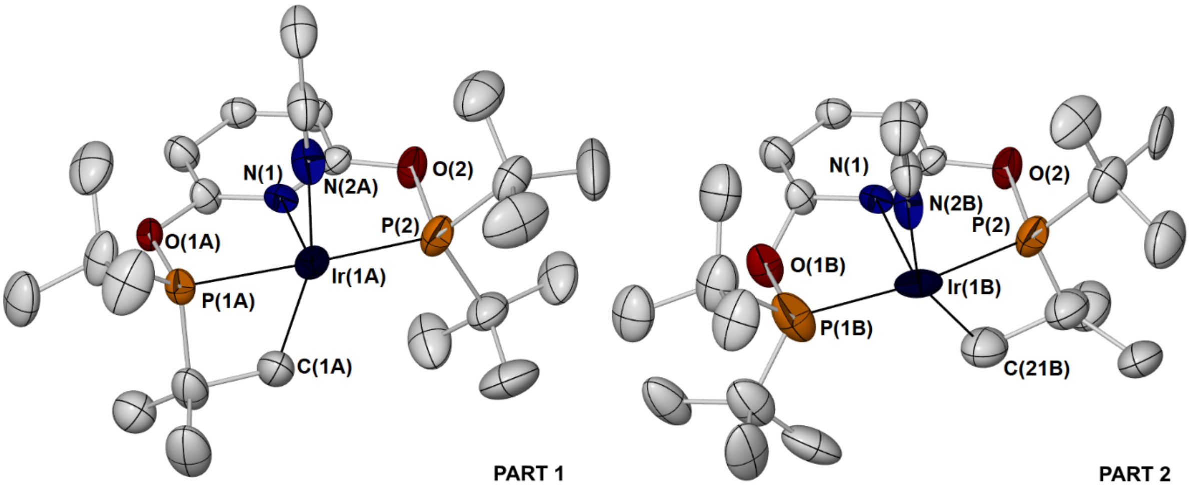

***Figure S98**. The cation of the molecular structure of **2-MeCN[BAr^F$_4$]** as determined by single-crystal X-ray diffraction viewed down the* h = *-1,* k = *-12,* l = *-4 vector. PART 1 and PART 2 of the complete model are displayed separately. Anisotropic displacement parameters are represented as 50% probability ellipsoids. All hydrogen atoms omitted for clarity. Selected bond lengths (Å): Ir(1A)-C(1A) 2.200(8), Ir(1B)-C(21B) 2.104(18), Ir(1A)-N(1) 2.078(4), Ir(1B)-N(1) 2.232(5), Ir(1A)-N(2A) 2.114(7), Ir(1B)-N(2B) 2.084(13), Ir(1A)-P(1A) 2.269(3), Ir(1B)-P(1B) 2.193(12), Ir(1A)-P(2) 2.3320(17), Ir(1B)-P(2) 2.210(4).*

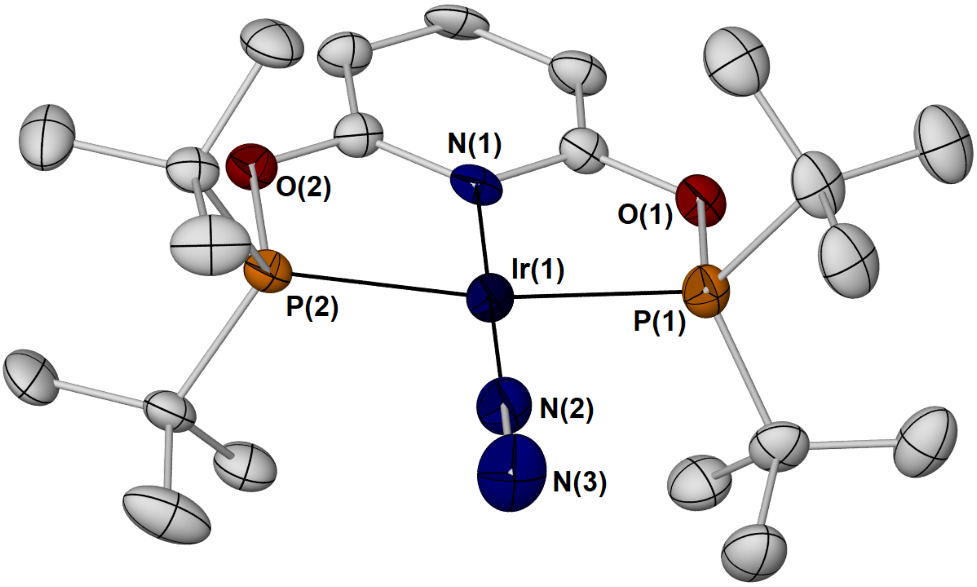

Figure S99. *The cation of the molecular structure of* ***3[BAr^{F}$_4$]*** *as determined by single-crystal X-ray diffraction. Anisotropic displacement parameters are represented as 50% probability ellipsoids. All hydrogen atoms omitted for clarity. Selected bond lengths (Å): Ir(1)-N(1) 2.009(7), Ir(1)-N(2) 1.928(9), Ir(1)-P(1) 2.266(2), Ir(1)-P(2) 2.272(2), N(2)-N(3) 1.120(12).*

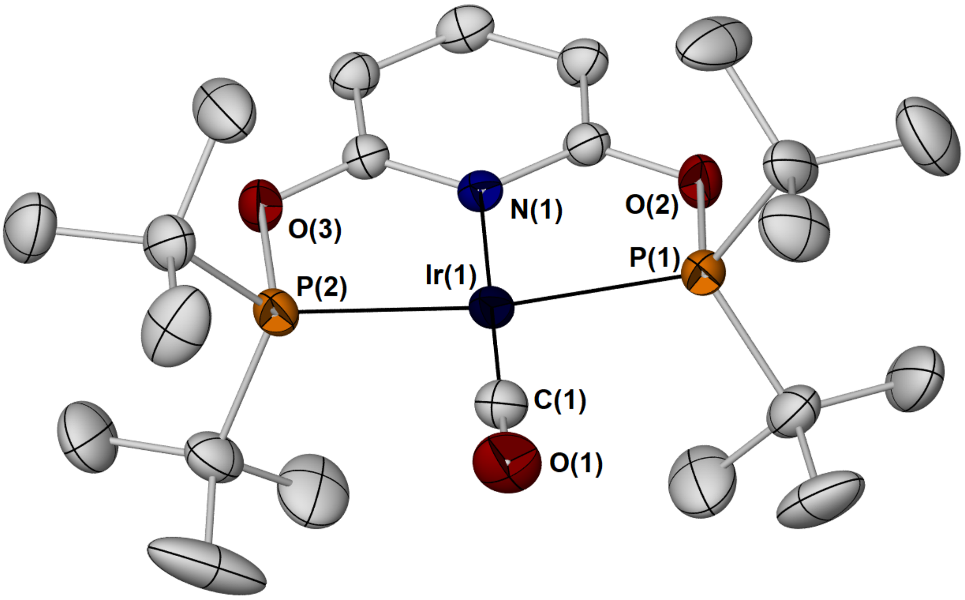

Figure S100. *The cation of the molecular structure of* ***4[BAr^{F}$_4$]*** *as determined by single-crystal X-ray diffraction. Anisotropic displacement parameters are represented as 50% probability ellipsoids. All hydrogen atoms omitted for clarity. Selected bond lengths (Å): Ir(1)-C(1) 1.844(4), Ir(1)-N(1) 2.062(2), Ir(1)-P(1) 2.2768(8), Ir(1)-P(2) 2.2825(8) C(1)-O(1) 1.151(4).*

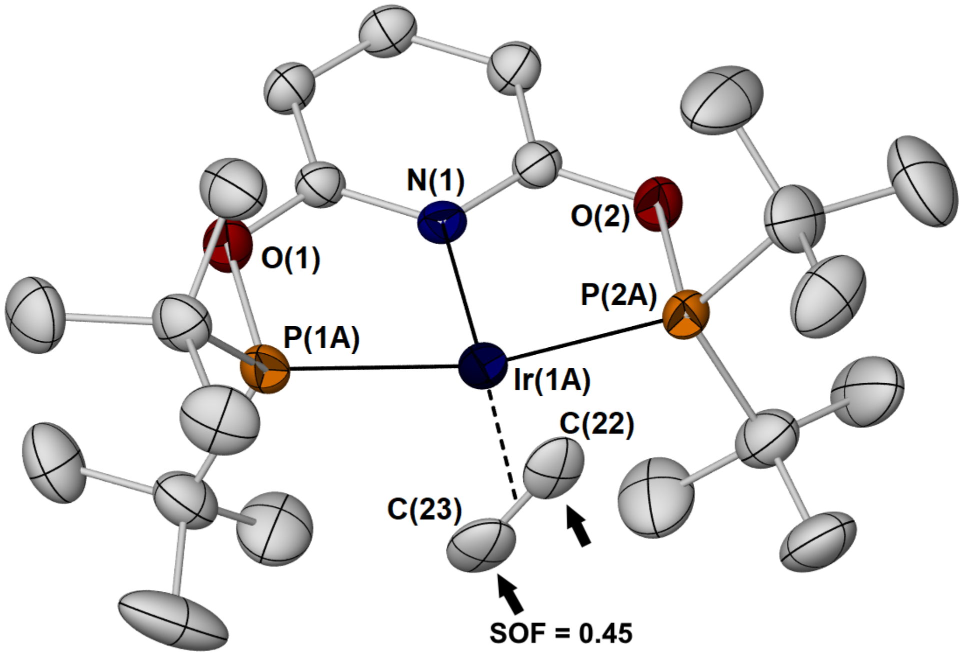

Figure S101. *The cation of the molecular structure of* ***5 + 6[BArF₄]*** *as determined by single-crystal X-ray diffraction. Anisotropic displacement parameters are represented as 50% probability ellipsoids. C(22) and C(23) are modelled as 45% occupancy sites by a SHELX free variable owing to the superposition of* ***5[BArF₄]*** *and* ***6[BArF₄]*** *in the crystal. All hydrogen atoms omitted for clarity. Selected bond lengths (Å): Ir(1A)-N(1) 2.075(2), Ir(1A)-P(1A) 2.2726(8), Ir(1A)-P(2A) 2.2739(8), Ir(1A)-C(22) 2.160(6), Ir(1A)-C(23) 2.139(7), C(22)-C(23) 1.298(13).*

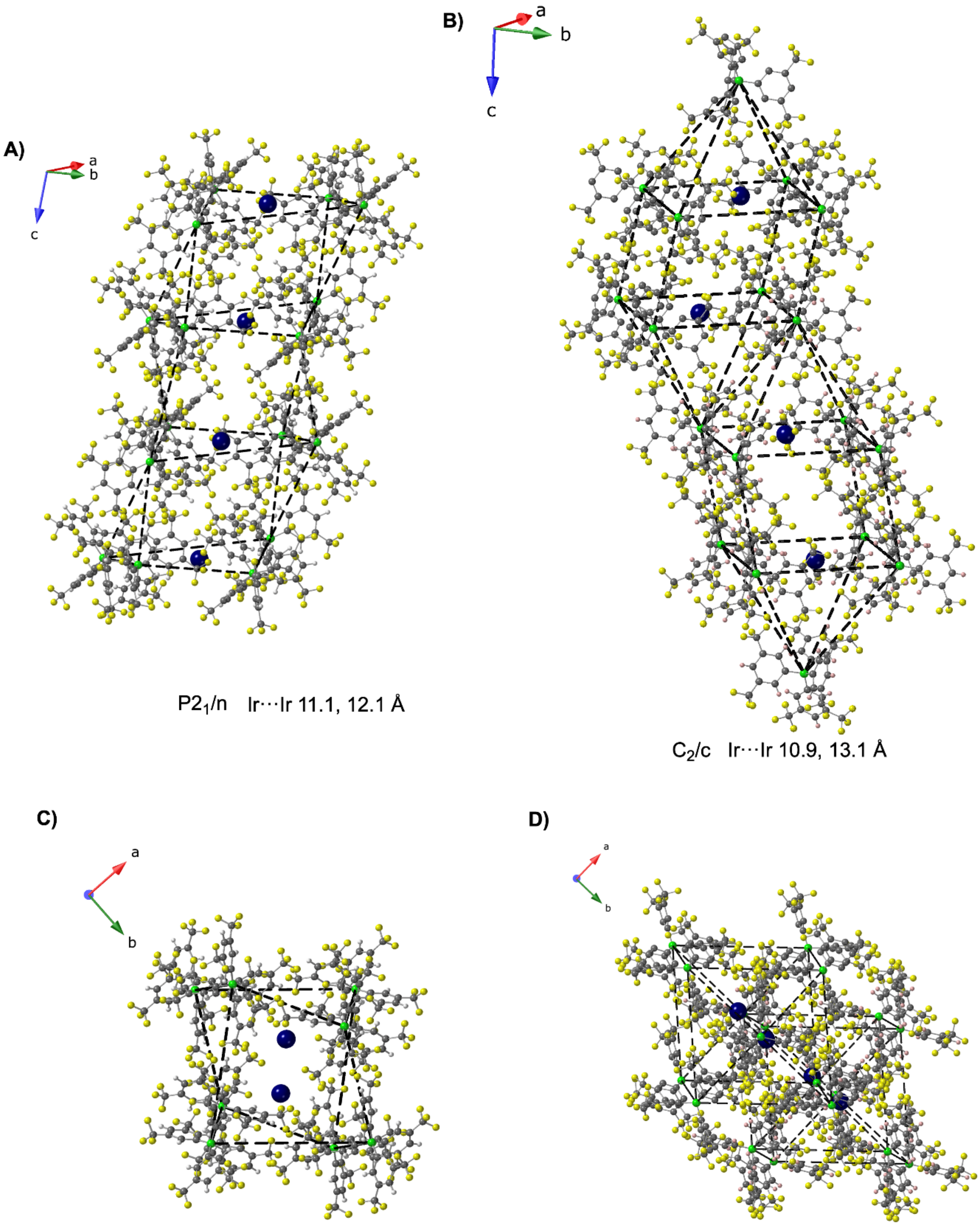

Figure S102. *A) Packing diagram of* ***α-1[BAr$^F{}_4$]****. B) Packing diagram of* ***4[BAr$^F{}_4$]****. C) Packing diagram of* ***α-1[BAr$^F{}_4$]*** *orientated along the c-axis. D) Packing diagram of* ***4[BAr$^F{}_4$]*** *orientated along the c-axis.*

	α-1[BAr^{F}$_4$]	α-1[BAr^{F}$_4$] (Room Temperature)	α-1[BAr^{F}$_4$] (Neutron)	β-1[BAr^{F}$_4$]
**Empirical Formula**	$C_{54}H_{55}BNO_2F_{24}P_2Ir$	$C_{54}H_{55}BNO_2F_{24}P_2Ir$	$C_{54}H_{55}BNO_2F_{24}P_2Ir$	$C_{54}H_{55}BF_{24}IrNO_2P_2$
**Formula Weight**	1470.94	1470.94	1470.97	1471.02
**Temperature/K**	110.0(10)	297.15	110	109.95(10)
**Crystal System**	Monoclinic	Monoclinic	Monoclinic	monoclinic
**Space group**	$P2_1/n$	$P2_1/n$	$P2_1/n$	$C2/c$
**a/Å**	16.478(3)	16.7968(3)	16.4747(2)	16.8801(2)
**b/Å**	18.4008(4)	16.7968(3)	16.4747(2)	18.0466(3)
**c/Å**	21.2079(4)	21.4408(5)	21.2078(3)	40.0439(6)
**α/°**	90	90	90	90
**β/°**	109.679(2)	109.868(2)	109.6740(10)	96.7770(10)
**γ/°**	90	90	90	90
**Volume/Å³**	6055(2)	6308.3(2)	6052.57(14)	12113.3(3)
**Z**	4	4	4	8
**ρ_{calc} g/cm³**	1.612	1.548	1.614	1.612
**μ/mm^{-1}**	2.372	2.276	0	5.800
**F(000)**	2916	2916	1396	5832
**Crystal size/mm³**	0.285 × 0.225 × 0.14	0.229 × 0.165 × 0.133	1.8 × 1.2 × 1.0	0.155 × 0.068 × 0.045
**Radiation**	Mo Kα (λ = 0.71073)	Mo Kα (λ = 0.71073)	N/A (neutron)	Cu Kα (λ = 1.54184)
**2Θ range for data collection/°**	6.87 to 58.158	6.444 to 62.26	5.29 to 176.06	7.198 to 154.066
**Index ranges**	−22 ≤ h ≤ 21, −20 ≤ k ≤ 24, −28 ≤ l ≤ 28	−20 ≤ h ≤ 24, −24 ≤ k ≤ 26, −29 ≤ l ≤ 25	−20 ≤ h ≤ 21, −26 ≤ k ≤ 26, −30 ≤ l ≤ 30	−21 ≤ h ≤ 21, −22 ≤ k ≤ 22, −50 ≤ l ≤ 44
**Reflections collected**	29631	70405	141417	59857
**Independent reflections**	13917 [R_{int} = 0.0304 R_{sigma} = 0.0483]	16504 [R_{int} = 0.0390 R_{sigma} = 0.0344]	13889 [R_{int} = 0.2085 R_{sigma} = 0.2615]	12477 [R_{int} = 0.0404 R_{sigma} = 0.0285]
**Data/restraints/parameters**	13917/381/919	16504/271/928	13889/522/1261	12477/474/1080
**Goodness-of-fit on F²**	1.031	1.010	1.240	1.057
**Final R index [I>=2σ (I)]**	R_1 = 0.0324 wR_2 = 0.0669	R_1 = 0.0380 wR_2 = 0.0840	R_1 = 0.1001 wR_2 = 0.0402	R_1 = 0.0344 wR_2 = 0.0828
**Final R index [all data]**	R_1 = 0.0464 wR_2 = 0.0738	R_1 = 0.0632 wR_2 = 0.0946	R_1 = 0.0892 wR_2 = 0.0372	R_1 = 0.0372, wR_2 = 0.0847
**Largest diff. peak/hole / e Å^{-3}**	0.83/−0.74	0.71/-0.39	2.20/-1.64	1.43/−0.94
**CCDC Deposition Number**	2410458	2410464	2410457	2410462

Table S4. *Selected crystallographic data.*

	2[BAr$^F_4$]	2-MeCN[BAr$^F_4$]·C_5H_{12}	3[BAr$^F_4$]	4[BAr$^F_4$]	5 + 6[BAr$^F_4$]
**Empirical Formula**	$C_{53}H_{51}BF_{24}IrNO_2P_2$	$C_{60}H_{65}BF_{24}IrN_2O_2P_2$	$C_{53}H_{51}BF_{24}IrN_3O_2P_2$	$C_{55}H_{51}BF_{24}IrNO_3P_2$	$C_{53.9}H_{52.8}BF_{23.93}IrNO_2P_2$
**Formula Weight**	1454.89	1567.09	1482.91	1482.90	1467.32
**Temperature/K**	109.95(10)	99(30)	110.05(10)	110.05(10)	110.00(10)
**Crystal System**	monoclinic	monoclinic	monoclinic	monoclinic	monoclinic
**Space group**	$P2_1/n$	$P2_1/c$	$C2/c$	$P2_1/n$	$P2_1/n$
**a/Å**	16.4217(2)	12.7813(3)	16.7836(2)	16.6821(3)	16.48670(2)
**b/Å**	18.3052(3)	14.4855(3)	18.0942(3)	18.1884(3)	18.1940(2)
**c/Å**	21.1070(3)	36.2124(7)	39.6346(5)	21.2173(4)	21.1342(3)
**α/°**	90	90	90	90	90
**β/°**	108.771(2)	97.355(2)	96.1670(10)	110.013(2)	108.8540(17)
**γ/°**	90	90	90	90	90
**Volume/Å^3**	6007.35(16)	6649.3(2)	11966.8(3)	6049.0(2)	5999.38(16)
**Z**	4	4	8	4	4
**ρ_{calc} g/cm^3**	1.608	1.565	1.646	1.628	1.623
**μ/mm^{-1}**	5.842	5.326	5.888	5.827	5.853
**F(000)**	2876	3132.0	5872	2936	2907
**Crystal size/mm^3**	0.197 × 0.123 × 0.097	0.163 × 0.106 × 0.068	0.131 × 0.105 × 0.07	0.119 × 0.112 × 0.071	0.213 × 0.121 × 0.096
**Radiation**	Cu Kα (λ = 1.54184)	Cu Kα (λ = 1.54184)	Cu Kα (λ = 1.54184)	Cu Kα (λ = 1.54184)	Cu Kα (λ = 1.54184)
**2Θ range for data collection/°**	7.46 to 153.736	7.842 to 153.808	7.206 to 133.198	7.446 to 153.986	7.686 to 153.724
**Index ranges**	−20 ≤ h ≤ 15, −22 ≤ k ≤ 22, −26 ≤ l ≤ 26	-16 ≤ h ≤ 15, -18 ≤ k ≤ 13, -44 ≤ l ≤ 45	−16 ≤ h ≤ 19, −21 ≤ k ≤ 19, −47 ≤ l ≤ 47	-18 ≤ h ≤ 21, -22 ≤ k ≤ 17, -26 ≤ l ≤ 26	-20 ≤ h ≤ 20, -13 ≤ k ≤ 22, -20 ≤ l ≤ 26
**Reflections collected**	39497	63383	55051	41405	57123
**Independent reflections**	12194 [R_{int} = 0.0310 R_{sigma} = 0.0282]	13666 [R_{int} = 0.0410 R_{sigma} = 0.0269]	10569 [R_{int} = 0.0545 R_{sigma} = 0.0308]	12279 [R_{int} = 0.0340 R_{sigma} = 0.0323]	12333 [R_{int} = 0.0429 R_{sigma} = 0.0280]
**Data/restraints/parameters**	12194/680/1058	13666/661/1149	10569/178/899	12279/258/955	12333/255/945
**Goodness-of-fit on F^2**	1.016	1.127	1.303	1.019	1.019
**Final R index [I>=2σ (I)]**	R_1 = 0.0384 wR_2 = 0.0973	R_1 = 0.0574 wR_2 = 0.1154	R_1 = 0.0726 wR_2 = 0.1737	R_1 = 0.0308 wR_2 = 0.0698	R_1 = 0.0333 wR_2 = 0.0838
**Final R index [all data]**	R_1 = 0.0421 wR_2 = 0.1002	R_1 = 0.0641 wR_2 = 0.1187	R_1 = 0.0747 wR_2 = 0.1744	R_1 = 0.0375 wR_2 = 0.0731	R_1 = 0.0374 wR_2 = 0.0878
**Largest diff. peak/hole / e Å^{-3}**	1.40/−0.69	0.83/-1.10	3.66/−1.33	1.52/−0.58	1.10/−1.10
**CCDC Deposition Number**	2410465	2410463	2410460	2410459	2410461

Table S5. *Selected crystallographic data.*

Computational Details

Solid-State Calculations

All static Kohn-Sham periodic-DFT calculations were performed employing the Gaussian Plane Wave (GPW) formalism as implemented in the QUICKSTEP[19] module in CP2K (Version 2023.1).[20] Molecularly optimized basis sets of double-ζ quality plus polarization in their short-range variant (DZVP-MOLOPT-SR-GTH)[21] were used for all atomic species. Interactions between the core electrons and the valence shell (Ir: 17, B: 3, C: 4, N: 5, O: 6, P: 5, F: 7, H: 1 electrons) were described by Goedecker-Teter-Hutter (GTH) pseudo potentials.[22-24] The generalized gradient approximation (GGA) to the exchange-correlation functional according to Perdew-Burke-Ernzerhof (PBE)[25] was used in combination with Grimme's D3 correction for dispersion interactions.[26] The auxiliary plane wave basis set was truncated at a cutoff of 700 Ry with initial mechanistic explorations for CH_4 loss from **α-1[BAr$^{F}_{4}$]** carried out with a cutoff of 500 Ry (Figure S108, Table S6 and Table S7). The maximum force convergence criterion was set to 10^{-4} Eh·Bohr^{-1}, whilst default values were used for the remaining criteria. The convergence criterion for the self-consistent field (SCF) accuracy was set to 10^{-7} Eh and 10^{-8} Eh for geometry optimizations. The Brillouin zone was sampled using the Γ-point. Initial coordinates for **α-1[BAr$^{F}_{4}$]**, **2[BAr$^{F}_{4}$]**, **3[BAr$^{F}_{4}$]**, and **4[BAr$^{F}_{4}$]** were obtained from the experimental crystallographic data where the neutron diffraction study was used for **α-1[BAr$^{F}_{4}$]**. Periodic boundary conditions (PBC) were applied throughout in combination with fixed unit cell parameters obtained from experiment.

Reactivity studies in the solid state were centred on one of the cations within the unit cells of either **α-1[BAr$^{F}_{4}$]** or **2[BAr$^{F}_{4}$]**, in which all cations are equivalent under the crystal symmetry (both $P2_1/n$; Z = 4). The 700 Ry cutoff was necessary to provide consistent results when optimizing the structure of **2[BAr$^{F}_{4}$]** from the experimental structure. For the reactions of the **1^{+}** cation within the **α-1[BAr$^{F}_{4}$]** unit cell C–H activation at all four ^{t}Bu groups was modelled (Table S10). The lowest energy pathways occurred at the top left ^{t}Bu group (see Table S6 and Table S8-Table S10) and these are reported in the main text. Transition state calculations were performed using the dimer method with tighter convergence criteria (maximum force 10^{-4} Hartree·Bohr^{-1} and SCF 10^{-7} Eh).[27] Optimized stationary points were characterised by analysis of their numerical second derivatives with a displacement of 0.01 Bohr, using a partial Hessian vibration analysis (PHVA) where only the atoms of the reacting cation were included. Minima and transition states have no or exactly one imaginary eigenvalue, respectively.[28] Transition states were also characterised by displacing the geometry forward and back along the imaginary mode and allowing the resultant geometries relax to the adjacent minima. PHVA were used to compute the thermodynamic data for the reactions using the TAMKIN tool.[29] Selected test calculations using a full vibrational Hessian analysis (FVHA) with all atoms included in the Hessian calculation showed similar results (see Table S6), as also noted by Ghysels and co-workers[30] and in previous studies by our group.[31]

Molecular Calculations

Geometry optimizations of the molecular cations taken from the optimised periodic DFT calculations were run with Gaussian 16 (Revision A.03).[32] These employed the PBE functional with Grimme's D3 dispersion correction.[25, 26] Stuttgart-Dresden (SDD)S17 relativistic effective core potentials (ECP) in combination with

the associated basis sets were used to describe Ir and P, with polarization functions added for P (ζ = 0.387).S18 6-31G(d,p) basis sets were used for all remaining atoms.[33, 34]

Methane Loss from α-1[BAr$^F_4$]

Two general pathways were assessed: Pathway A, CH_4 reductive elimination followed by cyclometallation and Pathway B, cyclometallation followed by CH_4 elimination. These were first explored with the isolated cation model and for Pathway B three variants were characterised: B1, involving an Ir(V) intermediate; B2 via a σ-methane complex and B3, a concerted process.

Isolated Cation Model

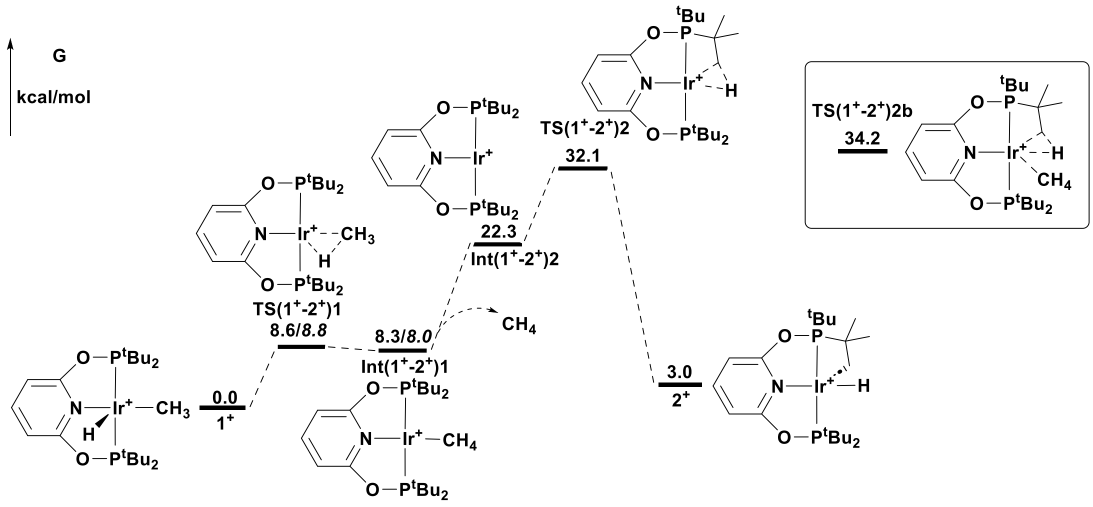

Figure S103. *Computed free energy reaction profile (kcal/mol, 298 K; isolated cation model) for methane loss from 1+ via reductive elimination followed by cyclometallation, Pathway A. For the initial reductive coupling steps values in italics are computed with the d4-[Ir(PONOP)(CD3)(D)]+ isotopologue. Inset: associative C–H activation transition state with CH4 retained in the metal coordination sphere.*

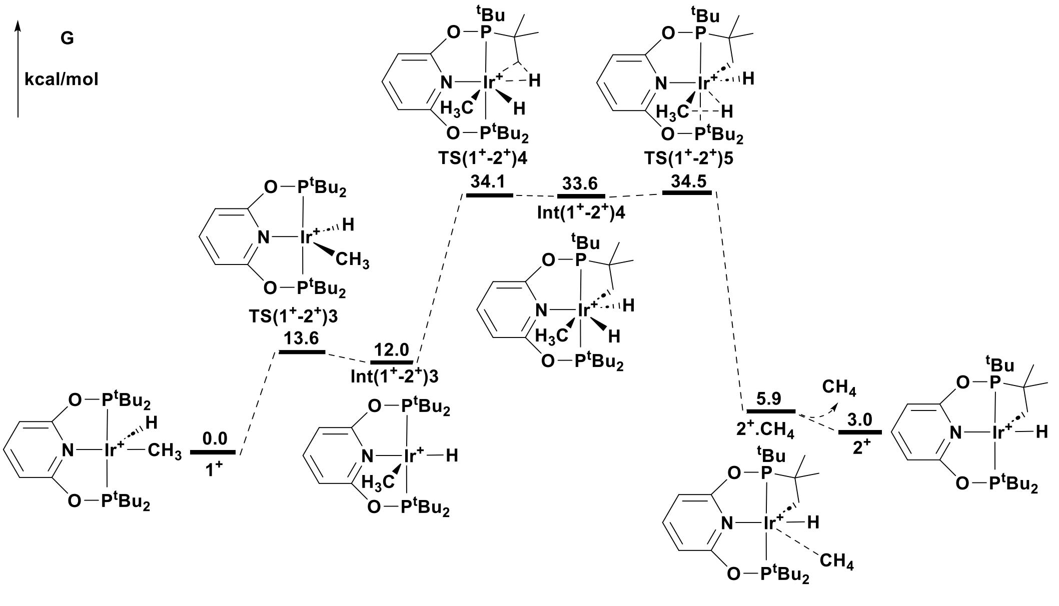

Figure S104. *Computed free energy reaction profile (kcal/mol, 298 K; isolated cation model) for methane loss from 1+ via initial cyclometallation to an Ir(V) intermediate, Pathway B1.*

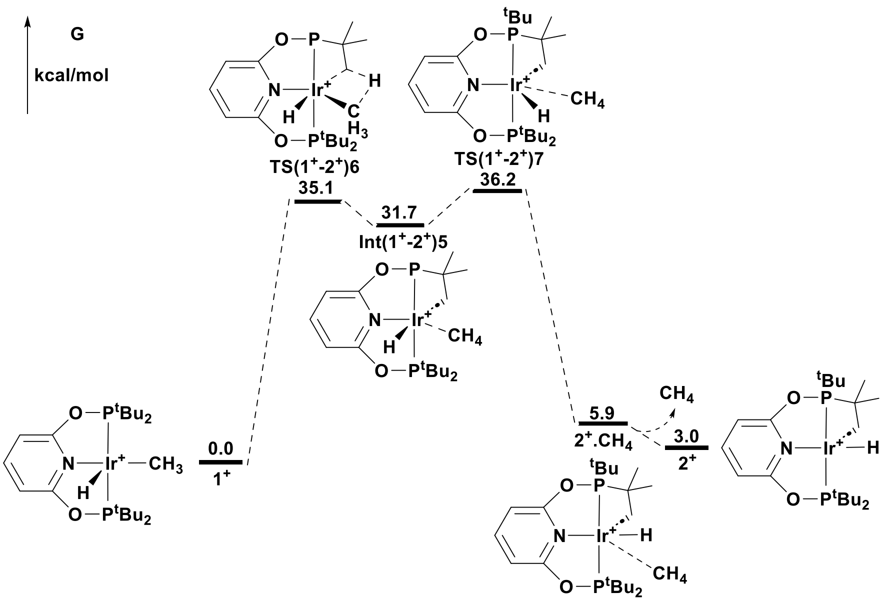

Figure S105. *Computed free energy reaction profile (kcal/mol, 298 K; isolated cation model) for methane loss from 1+ via initial cyclometallation to a σ-CH4 complex, Pathway B2.*

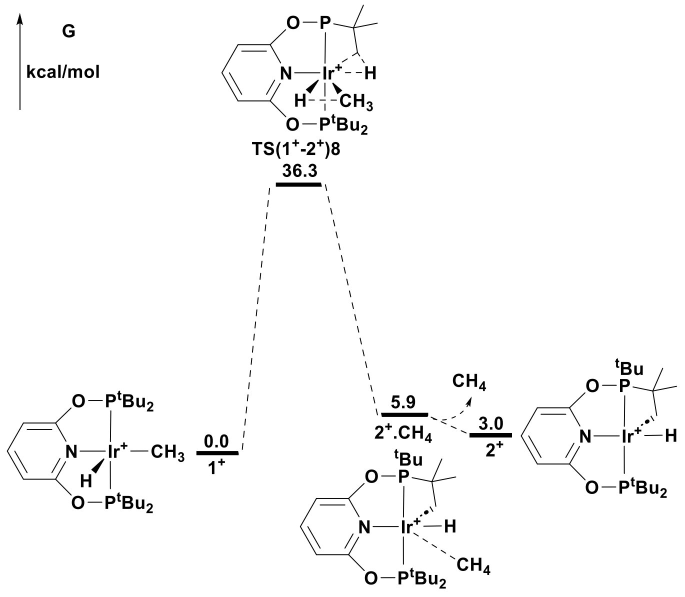

Figure S106. *Computed free energy reaction profile (kcal/mol, 298 K; isolated cation model) for methane loss from 1+ via a concerted process, Pathway B3.*

Solid-State Model

Methane Loss from α-1[BAr^F_4] to form 2^+@α-1[BAr^F_4].

Initial explorations of possible pathways for this process were based on the isolated cation model and performed using a 500 Ry cutoff. Selected stationary points were computed for all four ^{t}Bu substituents. Intermediate and transition state labels omit the unit cell designation for simplicity.

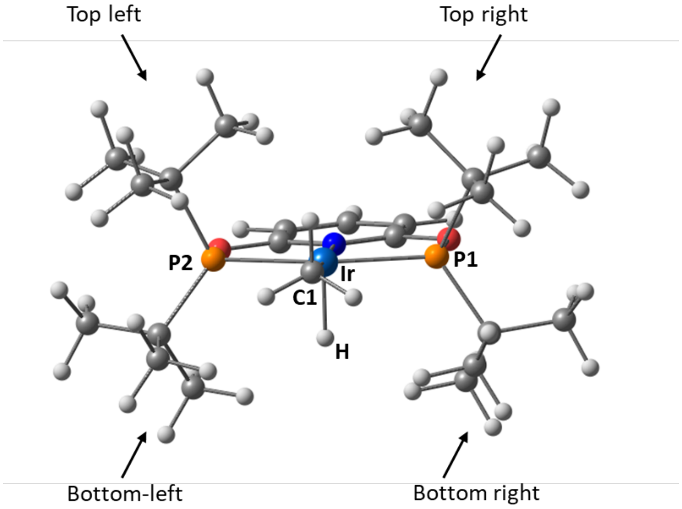

Figure S107. *Labelling system for the four distinct ^{t}Bu groups studied in the solid-state model.*

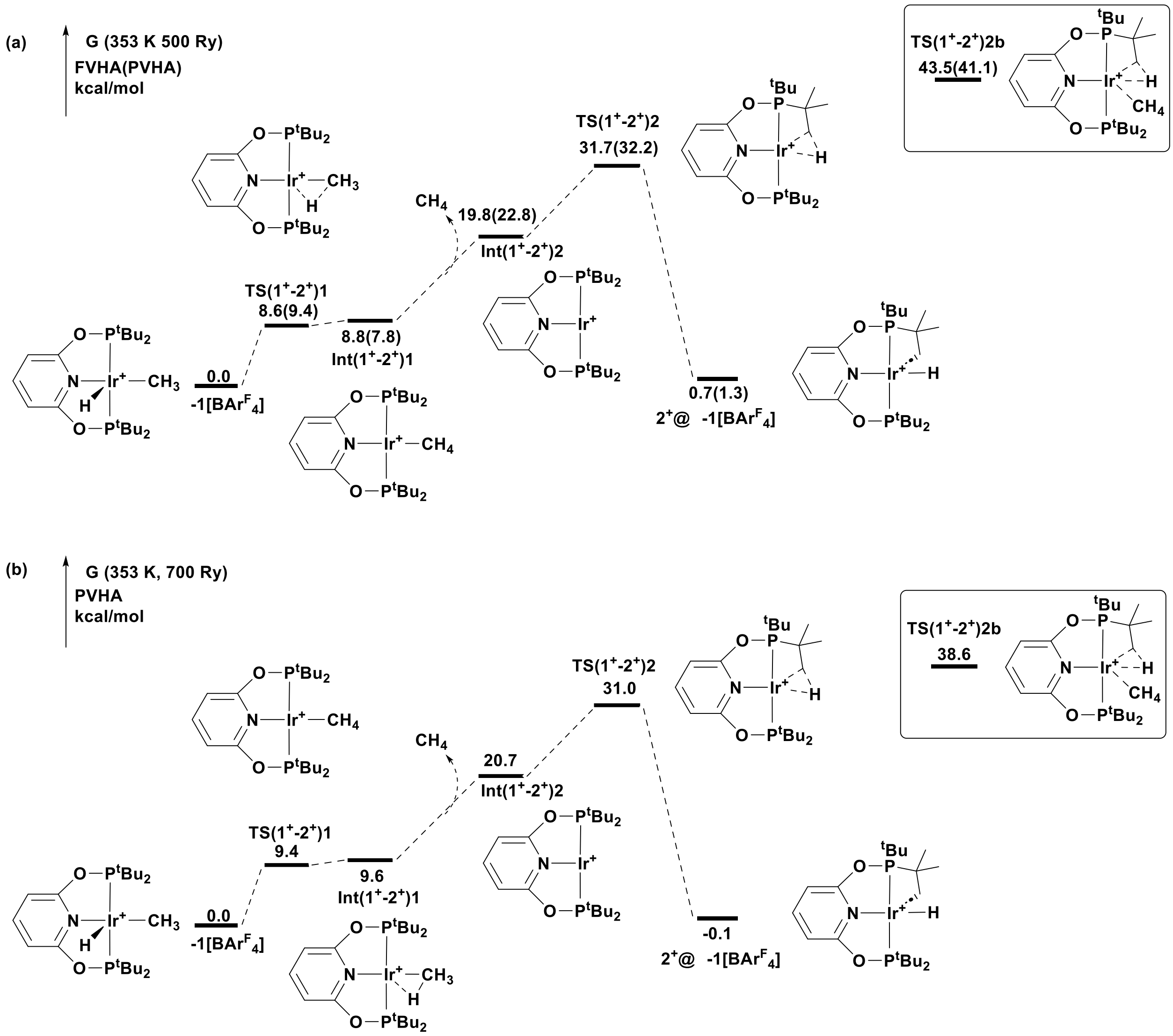

Figure S108. *(a) Lowest free energy reaction profile calculated with the solid-state model (top left ^{t}Bu group; kcal/mol; 353 K; FVHA(PVHA); 500 Ry cutoff) for methane loss from α-1[BAr^{F}$_4$]$^+$ via reductive elimination followed by cyclometalation. Pathway A. (b) As for (a) computed with a 700 Ry cutoff and PVHA. Insets: associative C–H activation transition states with CH_4 retained in the metal coordination sphere.*

^{t}Bu Group	Stationary Point	Absolute E_{SCF}	Relative E_{SCF}	Relative G^{FVHA}	Relative G^{PVHA}
	α-1[BAr$^{F}_{4}$]	-4342.28197	0.0	0.0	0.0
Top left	TS(1^{+}-2^{+})1	-4342.26497	10.7	8.6	9.4
	Int(1^{+}-2^{+})1	-4342.26749	9.1	8.8	7.8
	Int(1^{+}-2^{+})2	-4334.14831	37.0	19.8	22.8
	TS(1^{+}-2^{+})2	-4334.20443	48.7	31.7	32.2
	2^{+}@α-1[BAr$^{F}_{4}$]	-4334.18073	16.4	0.7	1.3
Top right	TS(1^{+}-2^{+})2	-4334.12223	53.4	34.1	37.4
	2^{+}@α-1[BAr$^{F}_{4}$]	-4334.17875	17.9	0.0	3.4
Bottom left	TS(1^{+}-2^{+})2	-4334.12067	54.3	39.8	38.8
	2^{+}@α-1[BAr$^{F}_{4}$]	-4334.17871	17.9	4.0	3.4
Bottom right	TS(1^{+}-2^{+})2	-4334.12856	49.4	33.7	33.9
	2^{+}@α-1[BAr$^{F}_{4}$]	-4334.18099	16.5	2.0	1.7
	CH_4	-8.07471	-	-	-

Table S6. *Absolute (au) and relative electronic energies (E_{SCF}) and relative free energies (kcal/mol; 353 K; 500 Ry cutoff) for methane loss from α-1[BAr$^{F}_{4}$]$^{+}$ via Pathway A. Data for all stationary points for the top left ^{t}Bu group are provided, along with those for the rate-limiting TS(1^{+}-2^{+})2 and 2^{+}@α-1[BAr$^{F}_{4}$] for reaction at the top right, bottom left and bottom right ^{t}Bu groups. Free energies are computed via a FVHA and PVHA for comparison.*

^{t}Bu Group	Stationary Point	Absolute E_{SCF}	Relative E_{SCF}	^{b}Relative G^{PVHA}
	α-1[BAr$^{F}_{4}$]	-4342.25433	0.0	0.0
Top left	TS(1^{+}-2^{+})1	-4342.23757	10.5	9.4
/	Int(1^{+}-2^{+})1	-4342.23984	9.1	9.6
	Int(1^{+}-2^{+})2	-4334.12223	36.0	20.7
	TS(1^{+}-2^{+})2	-4334.10438	47.2	31.0
	2^{+}@α-1[BAr$^{F}_{4}$]	-4334.15456	15.7	-0.1
	CH_4	-8.07471	-	-

Table S7. *Absolute (au) and relative electronic energies (E_{SCF}) and relative free energies (kcal/mol; 353 K; 700 Ry cutoff; PHVA) for methane loss from α-1[BAr$^{F}_{4}$]$^{+}$ via reaction of the top left ^{t}Bu group via Pathway A.*

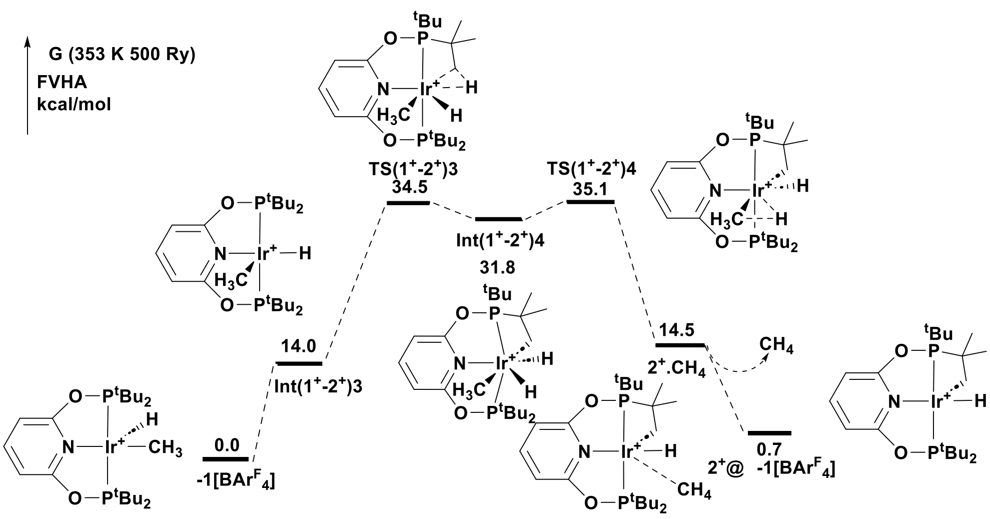

Figure S109. *Computed free energy reaction profile (top left ${}^{t}Bu$ group; kcal/mol, 353 K; solid-state model; FVHA; 500 Ry cutoff) for methane loss from α-1[BAr$^{F}_{4}$]$^{+}$ via an Ir(V) intermediate, Pathway B1.*

^{t}Bu Group	Stationary Point	Absolute E_{SCF}	Relative E_{SCF}	^{b}Relative G^{FVHA}
	α-1[BAr$^{F}_{4}$]	-4342.28197	0.0	0.0
Top left	Int(1^{+}-2^{+})3	-4342.26202	12.5	14.0
	TS(1^{+}-2^{+})3	-4342.22847	33.6	34.5
	Int(1^{+}-2^{+})4	-4342.22946	32.9	31.8
	TS(1^{+}-2^{+})4	-4342.22670	34.7	35.1
	2^{+}.CH_4	-4342.25512	16.8	14.5
Top right	TS(1^{+}-2^{+})3	-4342.22232	37.4	36.8
	Int(1^{+}-2^{+})4	-4342.22355	36.7	34.5
	TS(1^{+}-2^{+})4	-4342.22095	38.3	36.3
Bottom left	TS(1^{+}-2^{+})3	-4342.22581	35.2	40.2
	Int(1^{+}-2^{+})4	-4342.22712	34.4	39.6
	TS(1^{+}-2^{+})4	-4342.22304	37.0	41.8
Bottom right	TS(1^{+}-2^{+})3	-4342.22931	33.0	41.1
	Int(1^{+}-2^{+})4	-4342.23102	32.0	39.7
	TS(1^{+}-2^{+})4	-4342.22707	34.4	41.7

Table S8. *Absolute (au) and relative electronic energies (E_{SCF}) and relative free energies (kcal/mol; 353 K; 500 Ry cutoff; FVHA) for methane loss from α-1[BAr$^{F}_{4}$]$^{+}$ via an Ir(V) intermediate, Pathway B1. Data for all stationary points for the top left ${}^{t}Bu$ group are provided along with those for the high energy stationary points associated with reaction of the top right, bottom left and bottom right ${}^{t}Bu$ groups.*

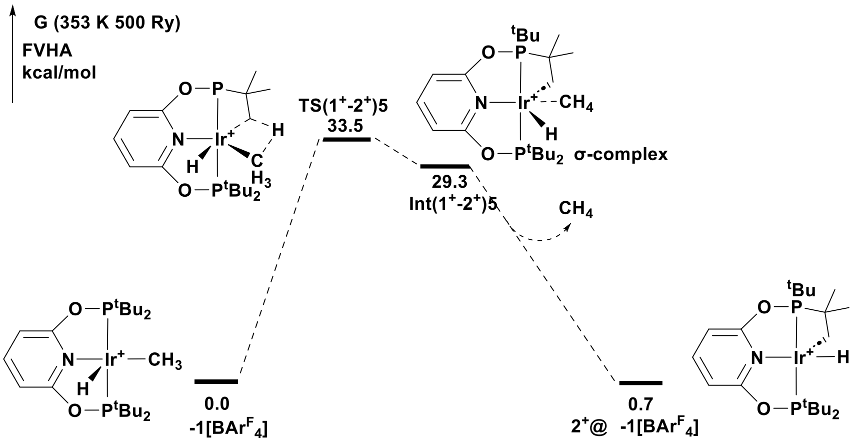

Figure S110. *Computed free energy reaction profile (kcal/mol, 353 K; solid-state model; PVHA; 500 Ry cutoff) for methane loss from α-1[BAr^F$_4$]$^+$ via a σ-CH_4 complex, Pathway B2.*

^{t}Bu Group	Stationary Point	Absolute E$_{SCF}$	Relative E$_{SCF}$	^{b}Relative G^{FVHA}
	α-1[BAr^F$_4$]	-4342.28197	0.0	0.0
Top left	TS(1$^+$-2$^+$)5	-4342.22260	37.3	33.5
	Int(1$^+$-2$^+$)5	-4342.22867	33.4	29.3
Top right	TS(1$^+$-2$^+$)5	-4342.21450	42.3	36.3
	Int(1$^+$-2$^+$)5	-4342.22772	34.0	31.2
Bottom left	TS(1$^+$-2$^+$)5	-4342.21537	41.8	37.2
	Int(1$^+$-2$^+$)5	-4342.22136	38.0	36.2
Bottom right	TS(1$^+$-2$^+$)5	-4342.21973	39.1	39.1
	Int(1$^+$-2$^+$)5	-4342.22531	35.6	35.7

Table S9. *Absolute (au) and relative electronic energies (E$_{SCF}$) and relative free energies (kcal/mol; 353 K; 500 Ry cutoff; FVHA) for methane loss from α-1[BAr^F$_4$]$^+$ via a σ-CH_4 complex, Pathway B2. Data for all stationary points for the top left ^{t}Bu group are provided along with those for the high energy stationary points associated with reaction at the top right, bottom left and bottom right ^{t}Bu groups.*

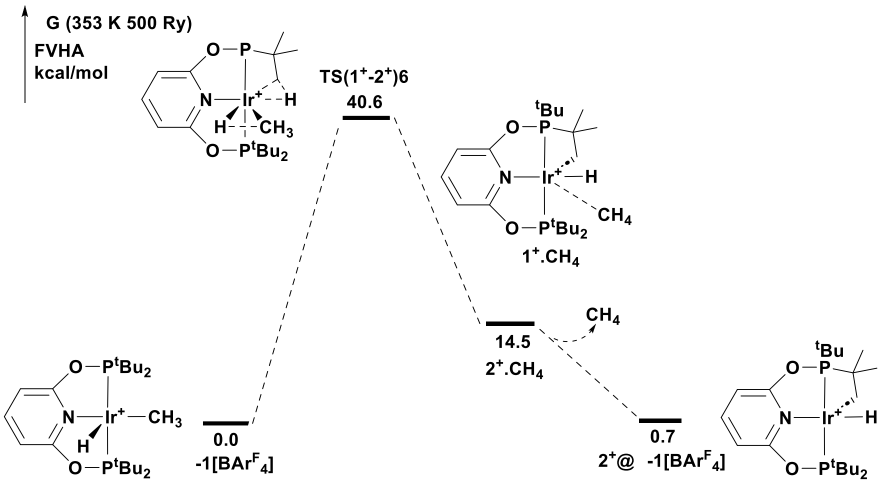

Figure S111. *Computed free energy reaction profile (kcal/mol, 353 K; solid-state model; PVHA; 500 Ry cutoff) for methane loss from α-1[BAr$^F{}_4$]$^+$ via a concerted process, Pathway B3.*

^{t}Bu Group	Stationary Point	Absolute E_{SCF}	Relative E_{SCF}	^{b}Relative G^{FVHA}
	α-1[BAr$^F{}_4$]	-4342.28197	0.0	0.0
Top left	TS(1$^+$-2$^+$)6	-4342.21879	39.6	40.6
	2$^+$.CH$_4$	-4342.25512	16.8	14.5
Top right	TS(1$^+$-2$^+$)6	-4342.21505	42.0	37.8
Bottom left	TS(1$^+$-2$^+$)6	-4342.21328	43.1	37.8
Bottom right	TS(1$^+$-2$^+$)6	-4342.21388	42.7	42.2

Table S10. *Absolute (au) and relative electronic energies (E_{SCF}) and relative free energies (kcal/mol; 353 K; 500 Ry cutoff; FVHA) for methane loss from α-1[BAr$^F{}_4$] via a concerted process, Pathway B3. Data for all stationary points for the top left ^{t}Bu group are provided along with those for TS(1$^+$-2$^+$)8 associated with reaction of the top right, bottom left and bottom right ^{t}Bu groups.*

Reaction of $2[BAr^F_4]$ with CH_4 to form $1^+@2[BAr^F_4]$.

The reverse processes of Pathways A (reductive coupling followed by oxidative addition) and Pathways B1-3 (via an Ir(V) intermediate, a σ-CH_4 complex and a concerted process) were again assessed. All results are computed with a 700 Ry cutoff. Intermediate and transition state labels omit the unit cell designation for simplicity.

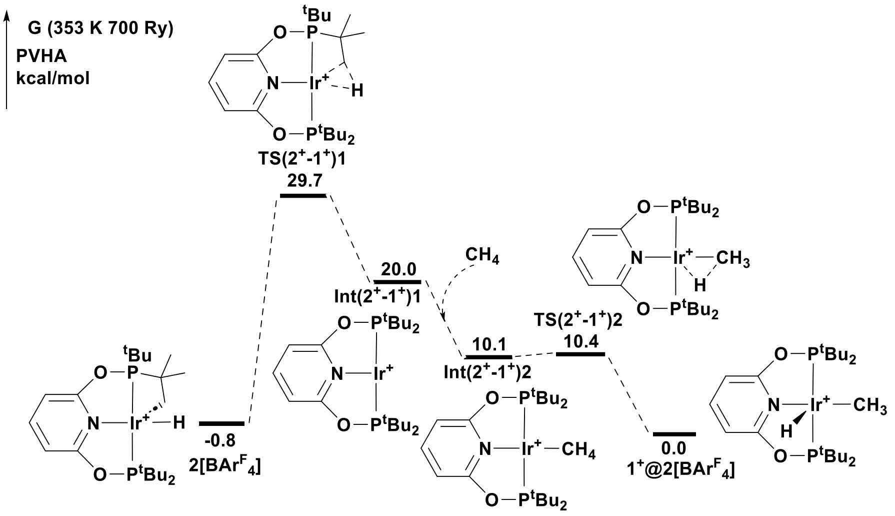

Figure S112. *Computed free energy reaction profile (kcal/mol, 353 K; solid-state model; PVHA) for methane addition to $2[BAr^F_4]^+$ via reductive coupling followed by oxidative addition, Pathway A.*

Stationary Point	Absolute E_{SCF}	Relative E_{SCF}	[b]Relative G^{PVHA}
$1^+@2[BAr^F_4]$	-4317.95994	0.0	0.0
$TS(2^+-1^+)2$	-4317.94317	10.5	10.4
$Int(2^+-1^+)2$	-4317.94527	9.2	10.1
$Int(2^+-1^+)1$	-4309.83114	33.9	20.0
$TS(2^+-1^+)1$	-4309.81326	45.2	29.7
$2[BAr^F_4]$	-4309.86465	12.9	-0.8

Table S11. *Absolute (au) and relative electronic energies (E_{SCF}) and relative free energies (kcal/mol; 353 K; 700 Ry cutoff; PVHA) for the reaction of $2[BAr^F_4]$ with methane via reductive coupling followed by oxidative addition, Pathway A.*

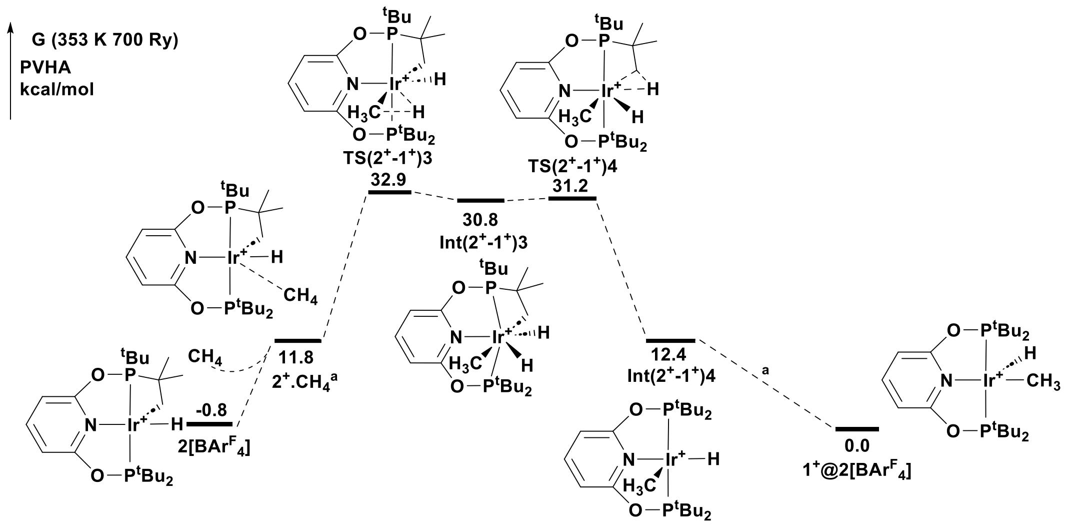

Figure S113. *Computed free energy reaction profile (kcal/mol, 353 K; solid-state model; PVHA; 700 Ry cutoff) for methane addition to 2[BAr$^F_4$]$^+$ via an Ir(V) intermediate, Pathway B1. [a]A transition state for isomerisation from Int(1$^+$-2$^+$)4 to 1@2[BAr$^F_4$] could not be located in the solid state. [a]The structures of 2$^+$.CH$_4$ derive from relaxation of the adjacent TS geometries and so may differ in each mechanism.*

Stationary Point	Absolute E_{SCF}	Relative E_{SCF}	[b]Relative G^{PVHA}
1$^+$@2[BAr$^F_4$]	-4317.95994	0.0	0.0
Int(2$^+$-1$^+$)4	-4317.94123	11.7	12.4
TS(2$^+$-1$^+$)4	-4317.91060	31.0	31.2
Int(2$^+$-1$^+$)3	-4317.90895	29.6	30.8
TS(2$^+$-1$^+$)3	-4317.90895	32.0	32.9
2$^+$.CH$_4$	-4317.93935	12.9	11.8
2[BAr$^F_4$]	-4309.86465	12.9	-0.8

Table S12. *Absolute (au) and relative electronic energies (E_{SCF}) and relative free energies (kcal/mol; 353 K; 700 Ry cutoff; PVHA) for the reaction of 2[BAr$^F_4$] with methane via an Ir(V) intermediate, Pathway B1.*

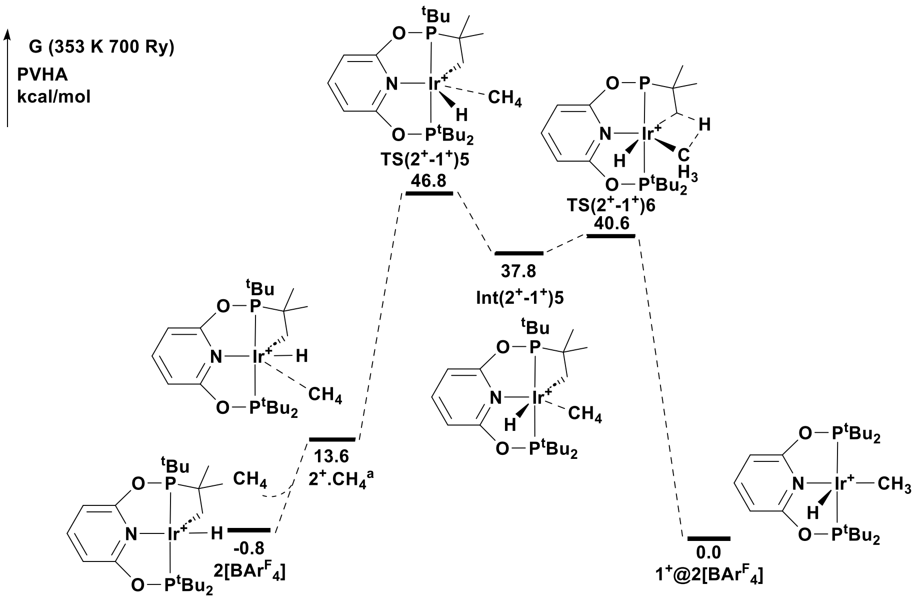

Figure S114. *Computed free energy reaction profile (kcal/mol, 353 K; solid-state model; PVHA; 700 Ry cutoff) for methane addition to 2[BAr^F$_4$]$^+$ via a σ-CH$_4$ complex, Pathway B2. [a]The structures of 2$^+$.CH$_4$ derive from relaxation of the adjacent TS geometry and so may differ in each mechanism.*

Stationary Point	Absolute E_{SCF}	Relative E_{SCF}	[b]Relative G^{PVHA}
1$^+$@2[BAr^F$_4$]	-4317.95994	0.0	0.0
TS(2$^+$-1$^+$)6	-4317.89410	41.3	40.6
Int(2$^+$-1$^+$)5	-4317.90228	36.2	37.8
TS(2$^+$-1$^+$)5	-4317.88455	47.3	46.8
2$^+$.CH$_4$	-4317.93756	14.0	13.6
2[BAr^F$_4$]	-4309.86465	12.9	-0.8

Table S13. *Absolute (au) and relative electronic energies (E_{SCF}) and relative free energies (kcal/mol; 353 K; 700 Ry cutoff; PVHA) for the reaction of 2[BAr^F$_4$] with methane via a σ-CH$_4$ complex, Pathway B2.*

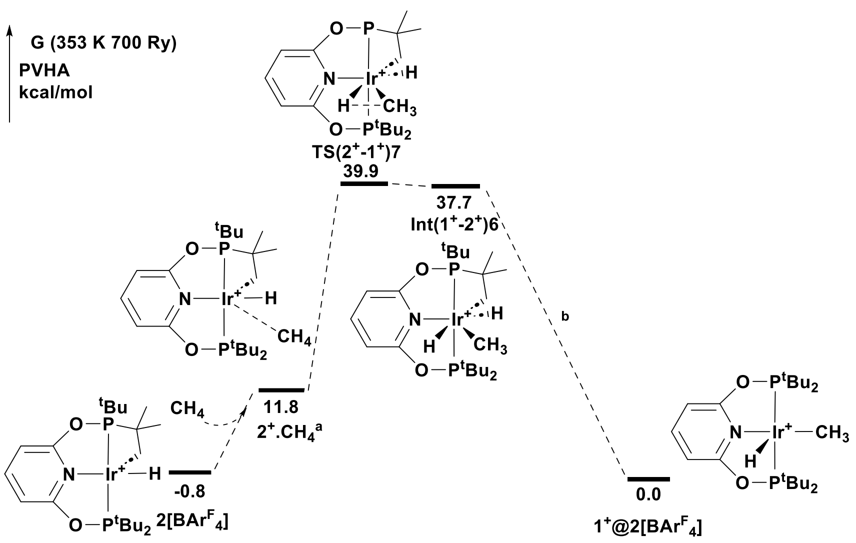

Figure S115. *Computed free energy reaction profile (kcal/mol, 353 K; solid-state model; PVHA; 700 Ry cutoff) for the reaction of 2[BAr^F$_4$] with methane targeting a concerted process, Pathway B3. Contrary to the equivalent processes computed with the isolated cation model and the reverse process within α-1[BAr^F$_4$], TS(2$^+$-1$^+$)7 does not correspond to a concerted process, but leads to a new Ir(V) intermediate, Int(1$^+$-2$^+$)6. [a]The structures of 2$^+$.CH$_4$ derive from relaxation of the adjacent TS geometry and so may differ in each mechanism. [b]A transition state for reductive coupling in Int(1$^+$-2$^+$)6 could not be located.*

Stationary Point	Absolute E$_{SCF}$	Relative E$_{SCF}$	[b]Relative G^{PVHA}
1$^+$@2[BAr^F$_4$]	-4317.95994	0.0	0.0
Int(2$^+$-1$^+$)6	-4317.93935	35.6	37.7
TS(2$^+$-1$^+$)7	-4317.89914	38.2	39.9
2$^+$.CH$_4$	-4317.93935	12.9	11.8
2[BAr^F$_4$]	-4309.86465	12.9	-0.8

Table S14. *Absolute (au) and relative electronic energies (E$_{SCF}$) and relative free energies (kcal/mol; 353 K; 700 Ry cutoff; PVHA) for the reaction of 2[BAr^F$_4$] with methane targeting a concerted process, Pathway B3.*

Reactions of 2[BAr^F$_4$] with N_2 and CO.

Isolated Cation Model

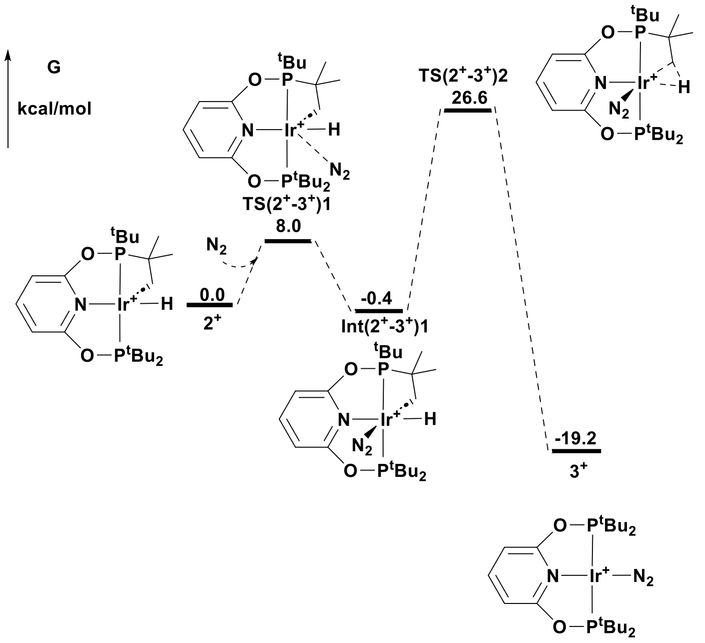

Figure S116. *Computed free energy reaction profile (kcal/mol, 298 K; isolated cation model) for the reaction 2^+ with N_2.*

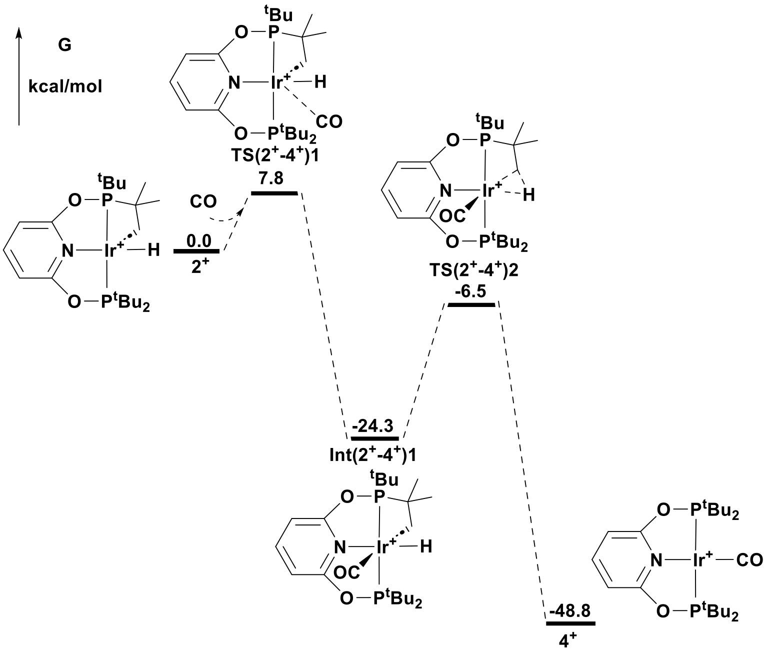

Figure S117. *Computed free energy reaction profile (kcal/mol, 298 K; isolated cation model) for the reaction 2^+ with CO.*

Solid-State Model

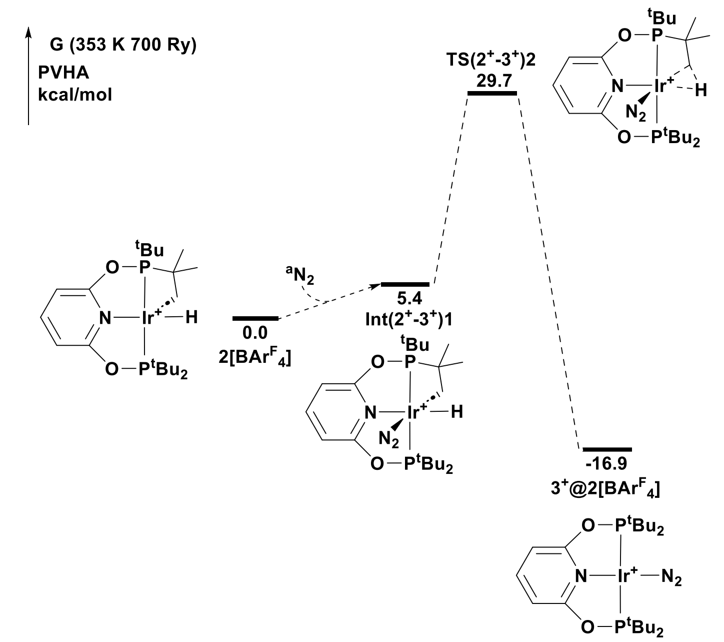

Figure S118. *Computed free energy reaction profile (kcal/mol, 353 K; solid-state model; PVHA; 700 Ry cutoff) for the reaction of 2[BAr$^F_4$] with N_2 to form 3^+@2[BAr$^F_4$]. [a]A transition state for N_2 addition to the 2^+ cation in 2[BAr$^F_4$] could not be located in the solid state.*

Stationary Point	Absolute E_{SCF}	Relative E_{SCF}	[b]Relative G^{PVHA}
2[BAr$^F_4$]	-4309.86465	0.0	0.0
Int(2^+-3^+)1	-4329.77663	-10.6	5.4
TS(2^+-3^+)2	-4329.73197	17.4	29.7
3^+@2[BAr$^F_4$]	-4329.80907	-31.0	-16.9

Table S15. *Absolute (au) and relative electronic energies (E_{SCF}) and relative free energies (kcal/mol; 353 K; 700 Ry cutoff; PVHA) for the reaction of 2[BArF4] with N_2.*

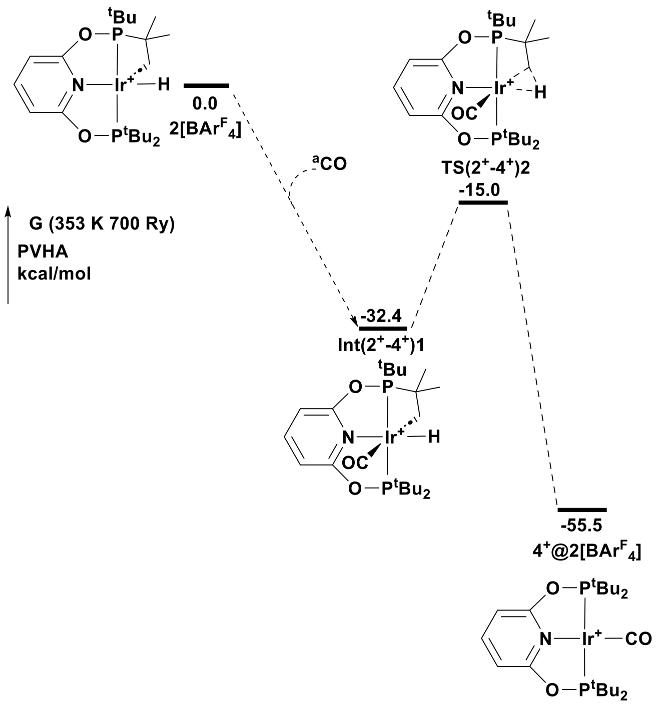

Figure S119. *Computed free energy reaction profile (kcal/mol, 353 K; solid-state model; PVHA; 700 Ry cutoff) for the reaction of 2[BAr^F$_4$] with CO to form 4$^+$@2[BAr^F$_4$]. [a]A transition state for CO addition to the 2$^+$ cation in 2[BAr^F$_4$] could not be located in the solid state.*

Stationary Point	Absolute E$_{SCF}$	Relative E$_{SCF}$	[b]Relative G^{PVHA}
2[BAr^F$_4$]	-4309.86465	0.0	0.0
Int(2$^+$-4$^+$)1	-4331.60801	-47.7	-32.4
TS(2$^+$-4$^+$)2	-4331.57450	-26.7	-15.0
4$^+$@2[BAr^F$_4$]	-4331.64532	-71.2	-55.5

Table S16. *Absolute (au) and relative electronic energies (E$_{SCF}$) and relative free energies (kcal/mol; 353 K; 700 Ry cutoff; PVHA) for the reaction of 2[BArF4] with CO.*

References

(1) Kundu, S.; Brennessel, W. W.; Jones, W. D. Synthesis and reactivity of new Ni, Pd, and Pt 2,6-bis(di-*tert*-butylphosphinito)pyridine pincer complexes. *Inorg. Chem.* **2011**, *50* (19), 9443-9453.
(2) Bernskoetter, W. H.; Hanson, S. K.; Buzak, S. K.; Davis, Z.; White, P. S.; Swartz, R.; Goldberg, K. I.; Brookhart, M. Investigations of iridium-mediated reversible C-H bond cleavage: characterization of a 16-electron iridium(III) methyl hydride complex. *J. Am. Chem. Soc.* **2009**, *131* (24), 8603-8613.
(3) Martinez-Martinez, A. J.; Weller, A. S. Solvent-free anhydrous Li^+, Na^+ and K^+ salts of $[B(3,5-(CF_3)_2C_6H_3)_4]^-$, $[BArF_4]^-$. Improved synthesis and solid-state structures. *Dalton Trans.* **2019**, *48* (11), 3551-3554.
(4) Brookhart, M.; Grant, B.; Volpe, A. F. $[(3,5-(CF_3)_2C_6H_3)_4B]^-[H(OEt_2)_2]^+$: a convenient reagent for generation and stabilization of cationic, highly electrophilic organometallic complexes. *Organometallics* **1992**, *11* (11), 3920-3922.
(5) Fulmer, G. R.; Miller, A. J. M.; Sherden, N. H.; Gottlieb, H. E.; Nudelman, A.; Stoltz, B. M.; Bercaw, J. E.; Goldberg, K. I. NMR Chemical Shifts of Trace Impurities: Common Laboratory Solvents, Organics, and Gases in Deuterated Solvents Relevant to the Organometallic Chemist. *Organometallics* **2010**, *29* (9), 2176-2179.
(6) Harris, R. K.; Becker, E. D.; Cabral De Menezes, S. M.; Granger, P.; Hoffman, R. E.; Zilm, K. W. Further conventions for NMR shielding and chemical shifts IUPAC recommendations 2008. *Solid State Nucl. Magn. Reson.* **2008**, *33* (3), 41-56.
(7) Morcombe, C. R.; Zilm, K. W. Chemical shift referencing in MAS solid state NMR. *J. Magn. Reson.* **2003**, *162* (2), 479-486.
(8) Hu, B.; Gay, I. D. Probing Surface Acidity by ^{31}P Nuclear Magnetic Resonance Spectroscopy of Arylphosphines. *Langmuir* **1999**, *15* (2), 477-481.
(9) Reid, S. M.; Neuner, B.; Schrock, R. R.; Davis, W. M. Synthesis of Rhenium Complexes That Contain the $[(C_6F_5NCH_2CH_2)_3N]^{3-}$ Ligand. *Organometallics* **1998**, *17* (18), 4077-4089.
(10) Naglav, D.; Buchner, M. R.; Bendt, G.; Kraus, F.; Schulz, S. Off the Beaten Track-A Hitchhiker's Guide to Beryllium Chemistry. *Angew Chem Int Ed* **2016**, *55* (36), 10562-10576.
(11) Campos, J.; Kundu, S.; Pahls, D. R.; Brookhart, M.; Carmona, E.; Cundari, T. R. Mechanism of hydrogenolysis of an iridium-methyl bond: evidence for a methane complex intermediate. *J. Am. Chem. Soc.* **2013**, *135* (4), 1217-1220.
(12) Findlater, M.; Schultz, K. M.; Bernskoetter, W. H.; Cartwright-Sykes, A.; Heinekey, D. M.; Brookhart, M. Dihydrogen complexes of iridium and rhodium. *Inorg. Chem.* **2012**, *51* (8), 4672-4678.
(13) Sheldrick, G. M. Crystal structure refinement with SHELXL. *Acta Crystallogr. C* **2015**, *71* (Pt 1), 3-8.
(14) Dolomanov, O. V.; Bourhis, L. J.; Gildea, R. J.; Howard, J. A. K.; Puschmann, H. *OLEX2*: a complete structure solution, refinement and analysis program. *J. Appl. Crystallogr.* **2009**, *42* (2), 339-341.
(15) Edwards, A. J. Neutron Diffraction – Recent Applications to Chemical Structure Determination. *Aust. J. Chem.* **2011**, *64* (7).
(16) Piltz, R. O. Accurate data processing for neutron Laue diffractometers. *J. Appl. Crystallogr.* **2018**, *51* (3), 635-645.
(17) Piltz, R. LaueG software for displaying and processing neutron Laue images. *J. Appl. Crystallogr.* **2018**, *51* (3), 963-965.
(18) Betteridge, P. W.; Carruthers, J. R.; Cooper, R. I.; Prout, K.; Watkin, D. J. CRYSTALS version 12: software for guided crystal structure analysis. *J. Appl. Crystallogr.* **2003**, *36* (6), 1487-1487.
(19) VandeVondele, J.; Krack, M.; Mohamed, F.; Parrinello, M.; Chassaing, T.; Hutter, J. Quickstep: Fast and accurate density functional calculations using a mixed Gaussian and plane waves approach. *Comput. Phys. Commun.* **2005**, *167* (2), 103-128.
(20) Hutter, J.; Iannuzzi, M.; Schiffmann, F.; VandeVondele, J. cp2k: atomistic simulations of condensed matter systems. *Wires Comput. Mol. Sci.* **2013**, *4* (1), 15-25.
(21) VandeVondele, J.; Hutter, J. Gaussian basis sets for accurate calculations on molecular systems in gas and condensed phases. *J. Chem. Phys.* **2007**, *127* (11), 114105.
(22) Goedecker, S.; Teter, M.; Hutter, J. Separable dual-space Gaussian pseudopotentials. *Phys. Rev. B.* **1996**, *54* (3), 1703-1710.
(23) Hartwigsen, C.; Goedecker, S.; Hutter, J. Relativistic separable dual-space Gaussian pseudopotentials from H to Rn. *Phys. Rev. B.* **1998**, *58* (7), 3641-3662.
(24) Krack, M. Pseudopotentials for H to Kr optimized for gradient-corrected exchange-correlation functionals. *Theor. Chem. Acc.* **2005**, *114* (1), 145-152.
(25) Perdew, J. P.; Burke, K.; Ernzerhof, M. Generalized Gradient Approximation Made Simple. *Phys. Rev. Lett.* **1996**, *77* (18), 3865-3868.

(26) Grimme, S.; Antony, J.; Ehrlich, S.; Krieg, H. A consistent and accurate ab initio parametrization of density functional dispersion correction (DFT-D) for the 94 elements H-Pu. *J. Chem. Phys.* **2010**, *132* (15), 154104.
(27) Henkelman, G.; Jónsson, H. A dimer method for finding saddle points on high dimensional potential surfaces using only first derivatives. *J. Chem. Phys.* **1999**, *111* (15), 7010-7022.
(28) Chadwick, F. M.; Kramer, T.; Gutmann, T.; Rees, N. H.; Thompson, A. L.; Edwards, A. J.; Buntkowsky, G.; Macgregor, S. A.; Weller, A. S. Selective C-H Activation at a Molecular Rhodium Sigma-Alkane Complex by Solid/Gas Single-Crystal to Single-Crystal H/D Exchange. *J. Am. Chem. Soc.* **2016**, *138* (40), 13369-13378.
(29) Ghysels, A.; Verstraelen, T.; Hemelsoet, K.; Waroquier, M.; Van Speybroeck, V. TAMkin: A Versatile Package for Vibrational Analysis and Chemical Kinetics. *J. Chem. Inf. Model.* **2010**, *50* (9), 1736-1750.
(30) Ghysels, A.; Van Speybroeck, V.; Pauwels, E.; Catak, S.; Brooks, B. R.; Van Neck, D.; Waroquier, M. Comparative study of various normal mode analysis techniques based on partial Hessians. *J. Comput. Chem.* **2010**, *31* (5), 994-1007.
(31) Altus, K. M.; Sajjad, M. A.; Gyton, M. R.; Whitwood, A. C.; Page, S. J.; Macgregor, S. A.; Weller, A. S. Solid/Gas In Crystallo Reactivity of an Ir(I) Methylidene Complex. *Organometallics* **2024**.
(32) *Gaussian 16 Rev. C.01*; Frisch, M. J.; Trucks, G. W.; Schlegel, H. B.; Scuseria, G. E.; Robb, M. A.; Cheeseman, J. R.; Scalmani, G.; Barone, V.; Petersson, G. A.; Nakatsuji, H.; et al. Gaussian, Inc.: Wallingford, CT, 2016.
(33) Hehre, W. J.; Ditchfield, R.; Pople, J. A. Self—Consistent Molecular Orbital Methods. XII. Further Extensions of Gaussian—Type Basis Sets for Use in Molecular Orbital Studies of Organic Molecules. *J. Chem. Phys.* **1972**, *56* (5), 2257-2261.
(34) Hariharan, P. C.; Pople, J. A. The influence of polarization functions on molecular orbital hydrogenation energies. *Theoret. Chim. Acta* **1973**, *28* (3), 213-222.
